# Healthcare resilience: a meta-narrative systematic review and synthesis of reviews

**DOI:** 10.1136/bmjopen-2023-072136

**Published:** 2023-09-20

**Authors:** Mark Z Y Tan, Gabrielle Prager, Andrew McClelland, Paul Dark

**Affiliations:** 1 Humanitarian and Conflict Response Institute, The University of Manchester, Manchester, UK; 2 Department of International Health, Johns Hopkins University, Baltimore, Maryland, USA; 3 Alliance Manchester Business School, The University of Manchester, Manchester, UK; 4 Clinical Research Network, National Institute for Health and Care Research, London, UK

**Keywords:** Systematic Review, HEALTH SERVICES ADMINISTRATION & MANAGEMENT, Hospitals, QUALITATIVE RESEARCH, PUBLIC HEALTH

## Abstract

**Objectives:**

The COVID-19 pandemic has tested global healthcare resilience. Many countries previously considered ‘resilient’ have performed poorly. Available organisational and system frameworks tend to be context-dependent and focus heavily on physical capacities. This study aims to explore and synthesise evidence about healthcare resilience and present a unified framework for future resilience-building.

**Design:**

Systematic review and synthesis of reviews using a meta-narrative approach.

**Setting:**

Healthcare organisations and systems.

**Primary and secondary outcome measures:**

Definitions, concepts and measures of healthcare resilience. We used thematic analysis across included reviews to summarise evidence on healthcare resilience.

**Results:**

The main paradigms within healthcare resilience include global health, disaster risk reduction, emergency management, patient safety and public health. Definitions of healthcare resilience recognise various hierarchical levels: individual (micro), facility or organisation (meso), health system (macro) and planetary or international (meta). There has been a shift from a focus on mainly disasters and crises, to an ‘all-hazards’ approach to resilience. Attempts to measure resilience have met with limited success. We analysed key concepts to build a framework for healthcare resilience containing pre-event, intra-event, post-event and trans-event domains. Alongside, we synthesise a definition which dovetails with our framework.

**Conclusion:**

Resilience increasingly takes an all-hazards approach and a process-oriented perspective. There is increasing recognition of the relational aspects of resilience. Few frameworks incorporate these, and they are difficult to capture within measurement systems. We need to understand how resilience works across hierarchical levels, and how competing priorities may affect overall resilience. Understanding these will underpin interdisciplinary, cross-sectoral and multi-level approaches to healthcare resilience for the future.

**PROSPERO registration number:**

CRD42022314729.

**STRENGTHS AND LIMITATIONS OF THIS STUDY**

- This is the first systematic review of reviews done on healthcare resilience, at organisational and system levels.
- A meta-narrative approach is used, which is particularly suitable for complex topics spanning across disciplines and hierarchical levels.
- Our analysis and synthesis allow for an interdisciplinary, cross-sectoral and multi-level framework for healthcare resilience.
- Individual (or micro-level) resilience is not included in this review.
- A review of reviews provides a broad overview but does not encompass all details or knowledge about the topic.

## Background

The COVID-19 pandemic has tested the resilience of healthcare across the globe. Previously thriving healthcare systems have struggled with multiple aspects; from hospital capacity^1^ to infection control measures,^2^ healthcare worker burnout^3^ to degrees of community trust,^4^ and from international research cooperation^5^ to vaccine tribalism and hesitancy.^6^ Our previous understanding of healthcare resilience requires re-evaluation.

To practically respond to the whole-of-society challenges in light of COVID-19, we need evidence that effectively integrates knowledge across these disciplines. This need has been highlighted by several reviews.^7 8^ The meta-narrative approach offers such a method. It has been used to track several disciplines converging within a complex field, and is useful for making sense of such complex, heterogenous, and disparate data across disciplines and sectors.^9 10^ It thus forms an interdisciplinary and cross-sectoral approach to healthcare resilience.

Recognition that resilience at individual, organisational and system levels affect each other has resulted in some early multi-level empirical research protocols.^11 12^ Yet, many studies tend to focus on single hierarchal levels,^13–15^ within set paradigms.^16 17^ For example, literature on psychological resilience tends to focus on acute interventions during or after crisis.^18^ These seldom consider wider determinants at organisational or system level which affect individual resilience.^3^ Similarly, some disaster risk reduction (DRR) frameworks have put little emphasis on recovery after crisis,^19^ which has been highlighted as an important factor to build resilience after COVID-19.^20^ Therefore, in addition to interdisciplinary and cross-sectoral evidence, multi-level knowledge must also be consolidated.

Given the sheer volume of empirical literature available on healthcare resilience, a multi-level review was impractical. Instead, reviews of reviews are particularly useful for gaining a broad overview of complex topics and systems with many dependencies. For example, it has helped to better understand policy and practice priorities in climate sciences,^21^ and to unpick COVID-19 vaccine hesitancy.^22^ Both are highly complex and current topics involving multiple sectors, disciplines and hierarchical levels.

We therefore undertook a systematic review of reviews using a meta-narrative approach to present an overview and critical look at healthcare resilience. It highlights lessons learnt from the COVID-19 pandemic and synthesises evidence towards an overarching framework. The research aims to:

- Explore the definitions, measures and concepts of healthcare resilience.
- Synthesise evidence from the exploration and analysis towards a broad overview of the topic.
- Present an interdisciplinary, cross-sectoral and multi-level framework from analysed data.

## Methods

### Theoretical approach

We undertook a systematic literature review of reviews using a meta-narrative approach.^23 24^ ^25^ We used RAMESES guidelines for reporting meta-narrative reviews.^24^ Preferred Reporting Items for Systematic Reviews and Meta-Analyses (PRISMA) guidelines were followed.^26^

The principles of the meta-narrative review are pragmatism, pluralism, historicity, contestation, reflexivity and peer review. This approach was adopted for several reasons. First, the field has been informed by many research traditions. Second, there exists historical agendas which have shifted the focus of resilience at various hierarchical levels (historicity). Third, there are sometimes contrasting findings about resilience (pluralism and contestation). Fourth, an interdisciplinary overview may be useful for policymakers, practitioners and academics (pragmatism). Such an approach helps to extract seemingly heterogenous data (peer review). Finally, the final search strategy was informed by some initial searches and snowball sampling, reflecting a reflexive protocol which was adapted according to findings.

### Search strategy

**Figure 1:**
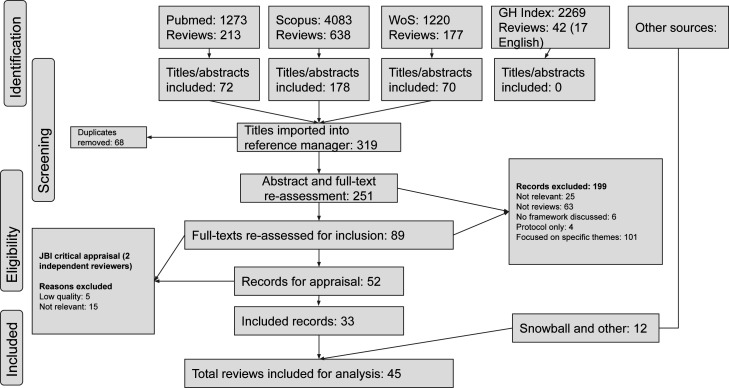
Search strategy.

A systematic keyword search was conducted in Scopus (Elsevier), Web of Science (Clarivate), PubMed (NIH) and Global Index Medicus (WHO)(figure 1). We included alternative spellings and synonyms. Inclusion criteria included reviews related to healthcare organisations and systems. We included records from 2008 to November 2022. The search was performed in January 2022 and repeated in November 2022. 2008 corresponded to the global financial crisis, which resulted in an increase in resilience literature.^27^ Resilience is dynamic.^28^ Concepts from earlier events may not possess the same relevance as more recent articles. This is especially the case since COVID-19. Reviews which were non-healthcare, focused on individual, psychological or community resilience alone, or concerned with specific diseases outside of epidemic contexts were excluded. We also excluded those focused on infection control measures only.^29^ There were many reviews on individual psychological resilience, particularly acute interventions to mental health crises. While we recognised the importance of individuals that make up organisations and systems, we wanted to focus on how resilience is understood at other hierarchical levels, so we excluded these reviews.

### Study selection

**Figure 2:**
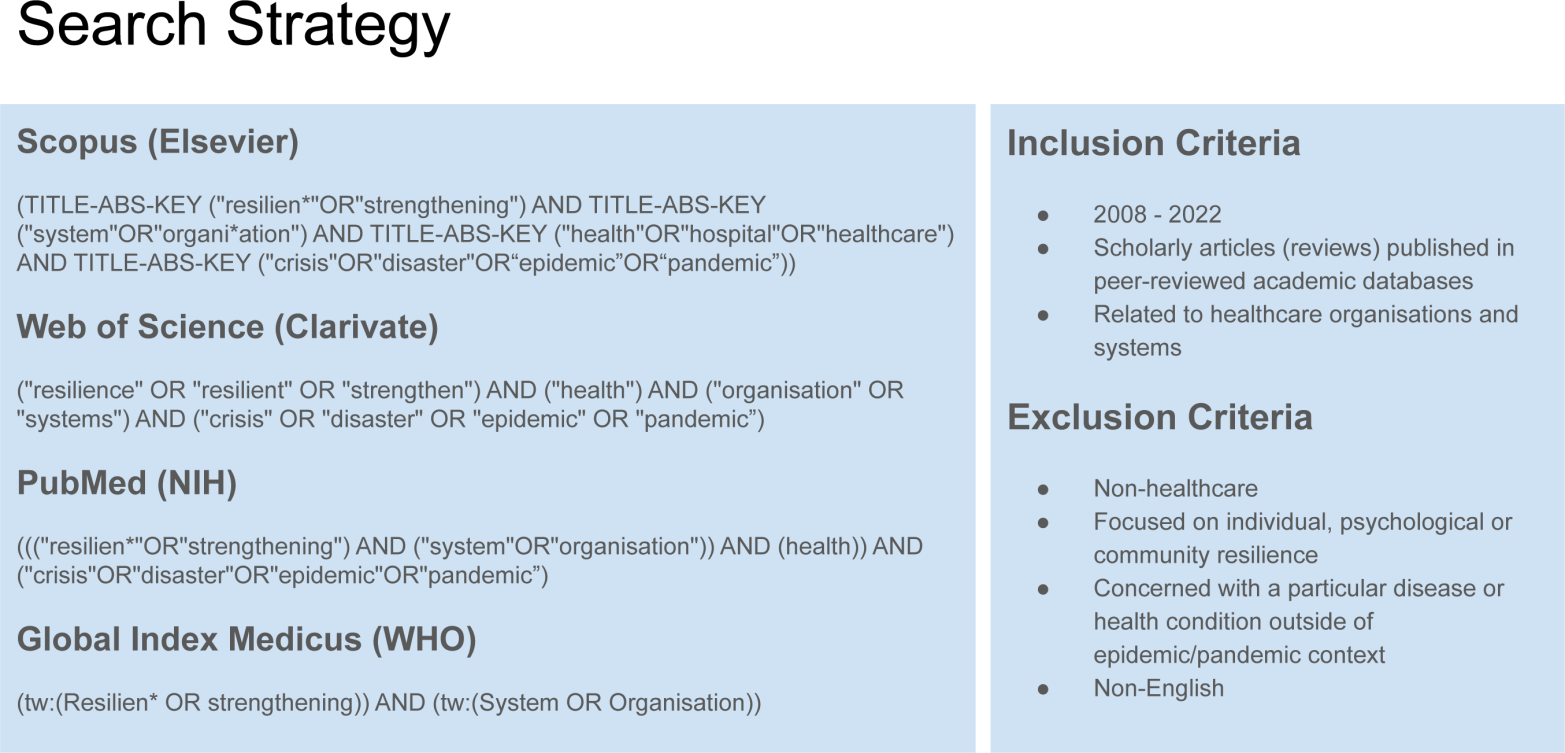
Preferred Reporting Items for Systematic Reviews and Meta-Analyses diagram.

Records were managed with Endnote 20 (Clarivate Analytics). Two reviewers independently selected papers. This was done for several reasons. First, there was a wide heterogeneity of studies and papers. Second, some reviews refer to cross-sectoral interactions but did not deal with healthcare. Third, the quality of reviews varied significantly. As a result, having independent reviewers (peer review) minimises personal bias and ensures there is agreement about selection criteria.^30 31^ Screening was done using a modified two-stage process. Title screening was insufficient as a stage on its own due to lack of information about the actual review, or only using ‘resilience’ as a tangential keyword. Both reviewers thus performed a title/abstract screening independently, followed by abstract/full-text screening and discussion around inclusion and exclusion criteria. Additional reviewers were agreed a priori should disagreements between the two primary reviewers occur. The final list of included papers was supplemented by snowball sampling and hand searching. This was done by searching through relevant references from selected reviews and discussions with other interdisciplinary team members. Because of the volume of results (figure 2), it was impractical to focus on empirical studies, or we would have had to confine the search to a single hierarchical level. Therefore, we included reviews only. This may reduce details and resolution, but it allows a summary of knowledge across several reviews and disciplines.

### Quality appraisal

Quality appraisal of articles was conducted using the Joanna Briggs Institute (JBI) checklist for systematic reviews.^32^ While there are no specific quality appraisal tools for systematic reviews of reviews, the JBI checklist formed a best fit compared with several other tools (online supplemental appendix 1).

10.1136/bmjopen-2023-072136.supp1 Supplementary data

### Data extraction and analysis

Using an inductive approach, three-tiered coding of the text was conducted around the three foci of definition, measures and concepts^33^ (online supplemental appendix 1). We used Atlas.ti qualitative software and data extraction tables. We examined prominent definitions and how they have been informed by different research disciplines. We then collated definitions from the reviews and analysed the most frequently occurring words (online supplemental appendix 2). These results were categorised and synthesised with the concepts of resilience to produce a definition which acknowledges past research, links current knowledge and spans hierarchical levels. Concepts were coded from reviews as well as from the frameworks presented in papers (online supplemental appendix 3).

### Patient and public involvement

There was no patient or public involvement in this research.

## Results

Four databases yielded 8845 records, of which 1070 were reviews. First-stage screening yielded 319 records, of which 68 were duplicates. Two hundred and fifty-one records were re-screened and discussed. The remaining 52 records underwent quality appraisal. This resulted in 33 records from the search which were analysed. Five reviews were excluded for low quality. They lacked details about search strategy, inclusion criteria, data analyses, or indeed clear research questions. Other reviews were not relevant according to inclusion and exclusion criteria. Snowball sampling yielded 12 records for analysis. These include reviews which discussed resilience frameworks. The PRISMA diagram summarises the selection process (figure 2).

### Definitions of healthcare resilience

Most reviews took their definition of healthcare resilience from previous papers. Three reviews contained lists of definitions from other studies.^17 34 35^ Another three reviews presented narrative collections and analyses of definitions.^15 36 37^

Resilience tends to be defined based on paradigms within which it operates.^35–37^ For example, within the engineering paradigm it is concerned with the ability of a structure or material to return to its original state.^16^ However, within business and ecological systems, it includes the ability for an organisation or system to capitalise or improve after a shock.^37–39^ A prominent definition of healthcare resilience is from Hollnagel *et al* as ‘the ability of the health care system (a clinic, a ward, a hospital, a county) to adjust its functioning prior to, during, or following events, and thereby sustain required operations under both expected and unexpected conditions’.^40 41^ While this definition continues to be refined, it provides a frame to highlight several aspects which emerged from our word occurrence analysis too.

**Figure 3:**
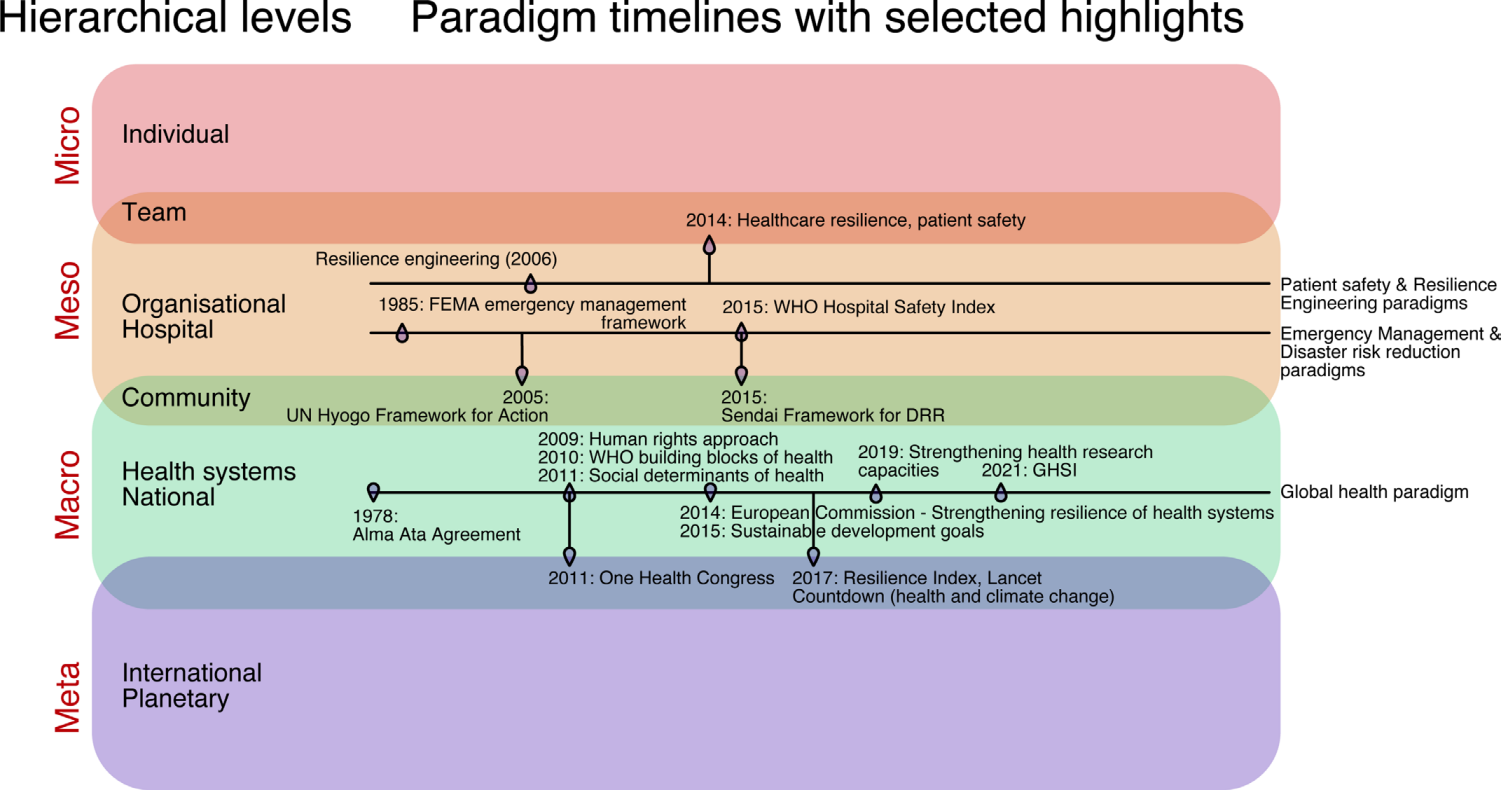
Healthcare resilience: hierarchical levels and selected paradigm timelines with historical highlights. DRR, disaster risk reduction; FEMA, Federal Emergency Management Agency; GHSI, Global Health Security Index.

First is the recognition of different hierarchical levels of the healthcare system^41 42^ (figure 3). There is consensus of these as micro-level (referring to individual), meso-level (hospital or organisation), macro-level (region or country).^41 42^ Several nuances continue to evade neat classification. For example, the team level does not neatly fit into micro-level or meso-level,^43^ and a community has been considered both meso-level and macro-level.^36 37^ There is also emergence of a meta-level of healthcare resilience. This level focuses mainly on the climate crisis as a determinant of health and how it threatens healthcare resilience and exacerbates inequalities.^8 44–46^ This transcends national boundaries and so must be considered on a meta-level.

Second, resilience spans pre-event, intra-event and post-event. This appreciates contributions from different paradigms. DRR studies are mostly concerned with pre-event aspects, such as preparing for shocks thereby minimising disturbance to a system.^13 47–51^ Emergency management (EM) studies examine aspects of how organisations adapt during a crisis (intra-event), and how they may learn from it.^17 52^ Some definitions do not include recovery, while others emphasise that rapidity of recovery back to steady state is a key feature of resilience.^16 36 49 53^

Third, both expected and unexpected events are considered. DRR and EM paradigms are concerned with disasters and unexpected crises, which suggest that resilience is only manifest when a sufficiently large shock is applied to the system.^34^ Patient safety paradigms seek to minimise unintended harm to patients, whether in crisis situations, or within normal operational stressors in healthcare.^7 35 37 54^ Recently, even DRR and EM reviews have adopted such an ‘all-hazards’ approach to resilience.^1 46^

Hollnagel *et al*’s definition of healthcare resilience, though widely used, does not specifically include recovery or review.^40^ Instead, he refers to these concepts as ‘learn’ within his related Resilience Analysis Grid (RAG).^55^ The resilience potentials in RAG are anticipate, respond, monitor and learn. Many other definitions map well onto Hollnagel’s definition and his four potentials. There are benefits in linking up definitions with ‘potentials’ or concepts of resilience. It provides convergence in a complex field, which helps to develop a consistent approach across hierarchical levels. This may promote understanding between policymakers and healthcare practitioners (including leaders and managers). In turn, it may lead to more unified approaches across discipline and hierarchical boundaries.

**Figure 4:**
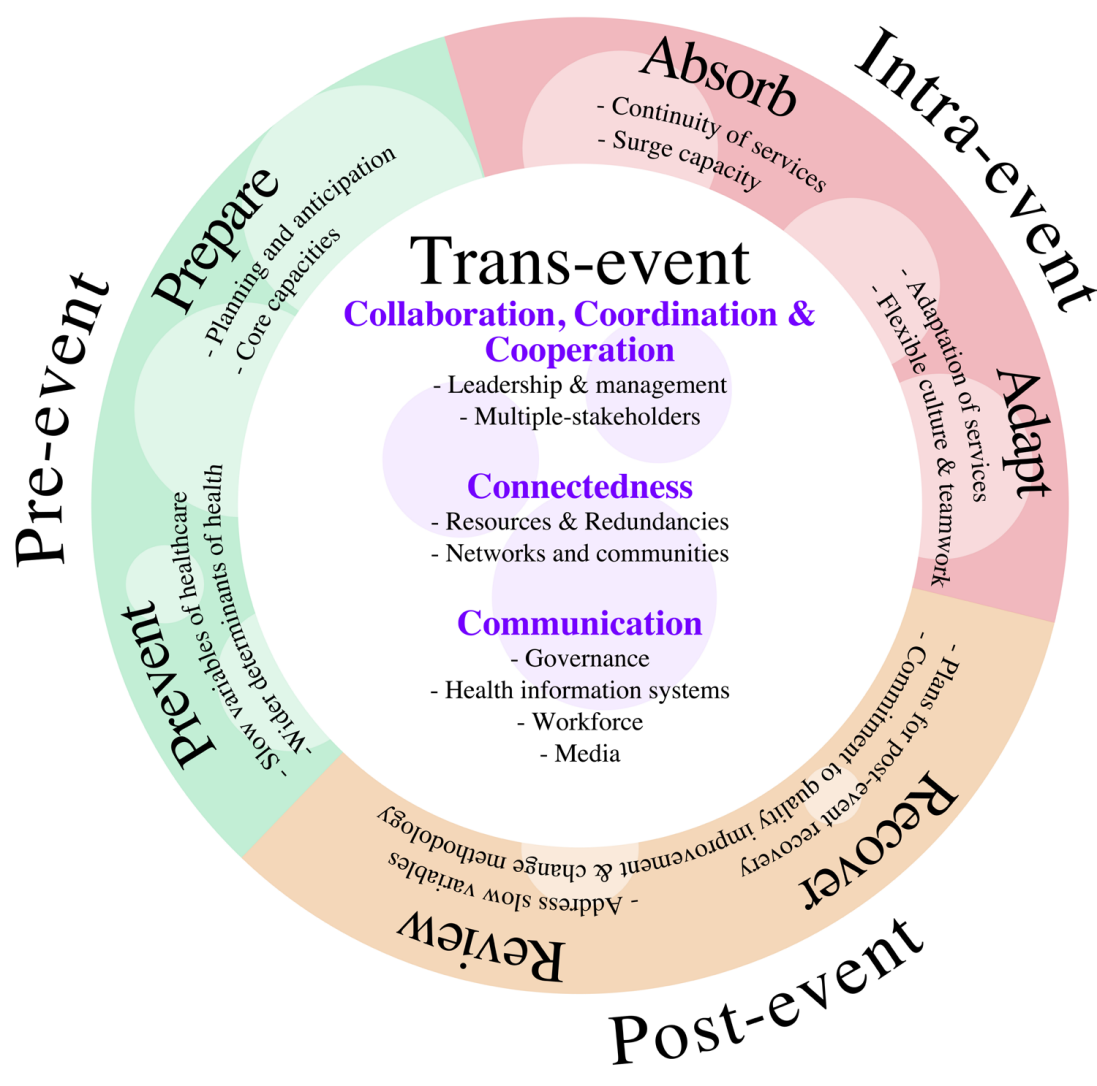
An interdisciplinary, cross-sectoral, and multi-level definition and conceptual framework for healthcare resilience. Defining features are listed as headings within the pre-event, intra-event, post-event and trans-event time domains.

We performed word occurrence analysis from the included reviews, followed by tiered coding into themes, with iterative refinement based on the analysis of concepts of resilience (online supplemental appendix 2). The result is a synthesised definition which complements previous work, spans across disciplines and dovetails with concepts explored in the next section. Our definition of healthcare resilience is ‘the ability of healthcare workers, organisations or systems to (a) prepare for and prevent, (b) absorb and adapt to maintain structure and essential functions, (c) recover and review from crises, shocks or stressors’ (figure 4).

### Concepts of healthcare resilience

**Figure 5:**
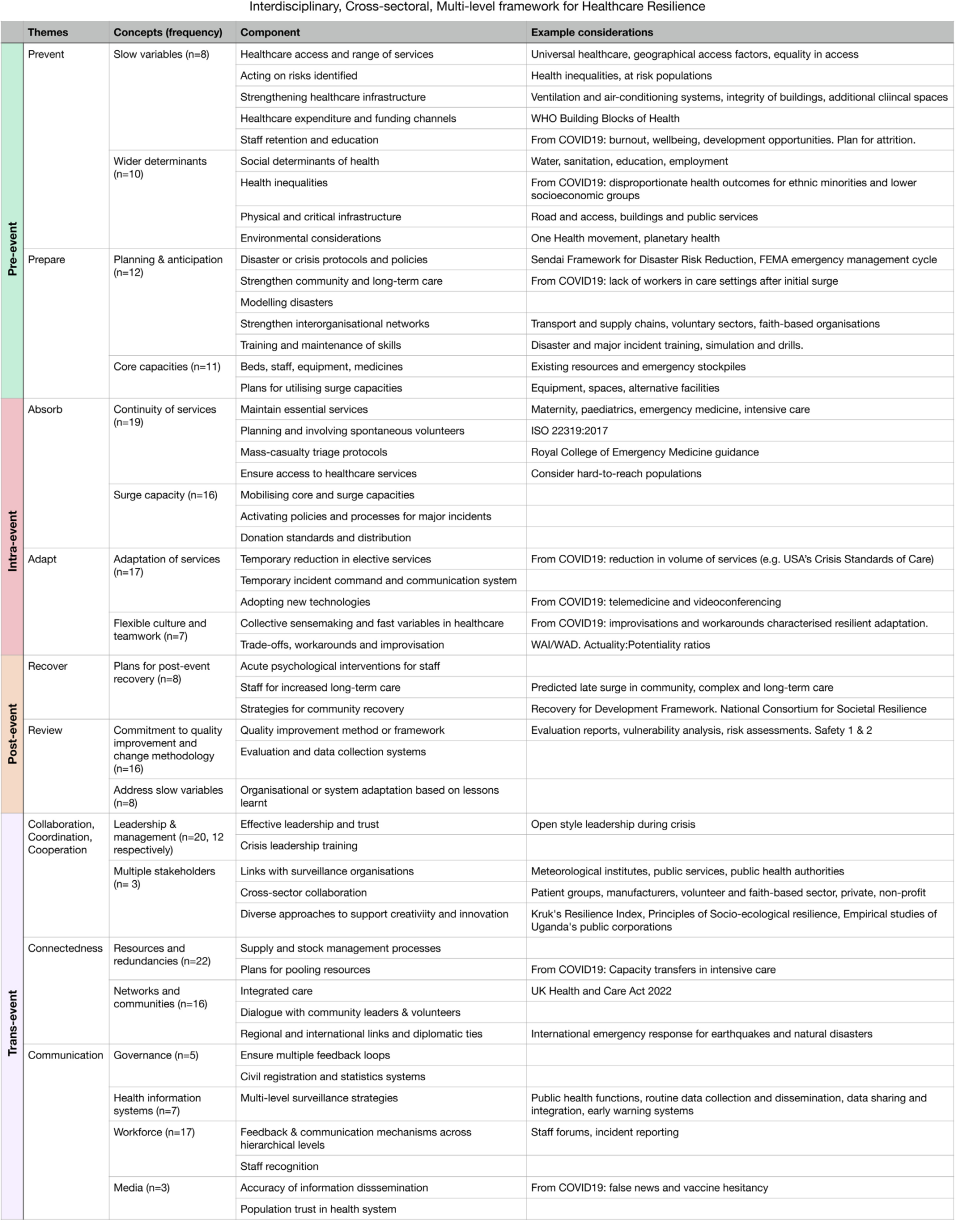
Details of the interdisciplinary, cross-sectoral and multi-level framework for healthcare resilience. Concepts are presented with frequency of occurrences as n-numbers. They are further divided into components, along with example considerations extracted from reviews. FEMA, Federal Emergency Management Agency.

Concepts may refer to practices or features which promote or hinder resilience, or indicators of resilience. Groups of concepts are often collated in frameworks, or set as best fit into established frameworks (eg, WHO building blocks of health). Frameworks are useful to systematically consider various aspects of healthcare resilience.^46 56^ We highlight key concepts at macro-level and meso-level, paying attention to lessons learnt from the COVID-19 pandemic. We then present our framework of concepts learnt from this review (figure 5), which dovetail with our definition (figure 4).

### Physical capacities

Within the DRR and EM paradigms, strengthening of existing capacities feature most prominently. This includes adequate workforce,^57^ equipment,^42 58^ financial resources^4^ and services^4 57^ to ensure maintenance of essential functions during a crisis. From the COVID-19 pandemic, appropriate distribution of physical capacities during various stages of a crisis are equally important. For example, changing shift patterns of emergency staff for increased demand intra-event, but redistributing staff to address longer-term care needs post-event.^59^ This adopts a longitudinal approach to resilience past the acute crisis and helps to address predicted shortages. As such, it may then prevent mere shifting of crisis from one organisation to another.

### Multi-level approaches

Several reviews have highlighted the multi-level approaches required to build resilience.^12 41 60–63^ A small number of empirical studies are currently underway to understand how this works in practice.^11 64^ Multi-level approaches recognise that while previous boundaries help understand specific details about how individual hierarchical levels pursue resilience, they are insufficient in representing the interconnections between levels. As an example, while improvisation and coping were seen as resilient concepts at the micro-level, healthcare workers’ ability to continue working was underpinned by the availability of resources and protective equipment provided by the organisation and their leaders (meso-level), as well as recovery efforts introduced after the crisis at meso-level and macro-level.^4 42 58^ Further work is needed to better understand the interplay and dynamics between the hierarchical levels.

### Recovery and review

The most appropriate method of quality improvement to ensure learning after a crisis depends on context. It is more important for organisations to demonstrate consistency and commitment to change methodology, rather than any specific tool or method.^65^ This is termed ‘review’ in our framework. Within the patient safety paradigm, two prominent and complementary concepts are safety1 and safety2 approaches. The safety1 approach attempts to get to the root cause of adverse events in healthcare, while the safety2 approach recognises that success occurs more frequently than adverse events and therefore lessons can be learnt from such practices too.^40^

Recovery is mentioned in DRR/EM literature,^16 53 66^ but less so in patient safety literature. This could be contextual. In DRR/EM, crises tend to be larger in scale and affect more people, organisations and sectors outside of healthcare. Patient safety tends to focus on teams within clinical emergency scenarios or normal operational circumstances. DRR/EM paradigms therefore require a more concerted recovery effort. Rapidity of recovery has been cited as a feature of resilience, but there is no benchmark. Extent of recovery to previous function has been explored, but this does not appreciate the occasional change in function of a system following a large crisis.^14 36^ Several reviews have emphasised the importance of having plans for recovery within resilience frameworks, but do not elaborate on details.^42 57 58^ Therefore, there is still much to understand about recovery, and some recent frameworks summarise key lessons learnt from COVID-19.^20^

### Relational aspects

The COVID-19 pandemic has highlighted the importance of relational aspects of resilience. A key aspect is effective leadership.^1 13 17 36 46 48 49 52 54 59–61 63 66–71^ Collaborative and authentic styles which were visible and approachable seemed most favoured during crises.^1 2 4 12 17 53 54 66 69 70 72 73^ Command-and-control structures have been cited, but these refer more to the flow of information rather than organisation of authority. Consequently, recent DRR reviews advocate decisions being pushed out towards the periphery, enabling more rapid adaptation to change.^16 49 52 57^

Networks beyond formal healthcare facilities bolsters resilience through the availability of physical capacities (eg, financing, staff, equipment and volunteers).^1 13 16 36 59 60 66 71 72 74^ It also facilitates adaptation and planning post-event. It also facilitates post-event concepts. For example, COVID-19 clinical and vaccine trials depended on international collaboration between many disciplines and industries.^5^ Recognising the importance of this concept, the recent UK Health and Care Act 2022 specifically focuses on interorganisational relationships towards the provision of integrated care. Further research will therefore be required to better understand how these relationships work in practice.^75–77^

### Interdisciplinary, cross-sectoral and multi-level framework for healthcare resilience

These concepts, along with others which emerged from analysis of the reviews, have been compiled into an interdisciplinary, cross-sectoral and multi-level framework (figures 4 and 5, online supplemental appendix 3). It spans meso-level and macro-level, which few existing frameworks do. It balances the historical focus on physical capacities with relational aspects of resilience. It incorporates lessons learnt from the COVID-19 pandemic. The framework may be used as tool to systematically consider various aspects of organisational and systems resilience.

The framework is divided into timeframes (pre-event, intra-event, post-event and trans-event). Within timeframes are themes (prepare and prevent, etc). Themes encompass several concepts, and finally, these concepts are further broken down into components (figures 4 and 5). In figure 4, the size of faint circles indicates the frequency of concepts extracted from the reviews, which are also represented as n-numbers in figure 5.

### Measures of resilience

Due to the considerable global interest in healthcare resilience, there have been attempts to develop measures or indicators for resilience. The process of pursuing resilience requires ways to determine progression. Attempts to measure resilience therefore allow comparisons and identification of potential areas for improvement. However, none of the current measures have been validated, and some widely used indices have been shown to be inaccurate during the COVID-19 pandemic.^78^

Two examples at the macro-level are the Global Health Security Index and Epidemic Preparedness Index. They both focus on infectious diseases outbreaks and contain several domains including public health and healthcare infrastructure (eg, surveillance capabilities, medical workforce), economic resources, and risk assessment and communication strategies.^2 79^ As such, they are focused on the pre-event domain of crises. However, many high-income countries that scored well on these indices performed very poorly during the COVID-19 pandemic.^78^ Several reasons have been cited, including lack of consideration of existing health inequalities, globalisation, societal connectedness and a bias towards physical capacities (eg, number of hospitals or beds).^78^ Of note, increased globalisation suggests that ‘disease control may be only as effective as practices within the poorest performing countries’.^78^ This supports the need for a meta-level of resilience where global health inequalities affect the healthcare resilience of other nations.

Some reviews attempt to evaluate a country’s resilience according to one or more domains of the WHO building blocks of health framework.^14 57 63 67 80^ However, the framework was designed as a systematic approach to the funding decisions within healthcare, rather than to evaluate resilience.^35^

Meso-level measures tend to stem from DRR/EM paradigms, focused on healthcare facilities. Several reviews have collated evidence from empirical papers centred around earthquakes and natural disasters. Quantitative measures include physical capacities (eg, number of beds, staff, equipment), physical infrastructure (eg, structural integrity of buildings)^1^ and actuality:potentiality ratios (eg, adherence to operating protocols).^36 81^ Qualitative indicators include checklists containing several components important during disaster situations (eg, pre-emptive protocols and procedures, command structures, compliance to structural regulations).^16 66^ These qualitative components address the intra-event domain of the resilience framework. By providing measurement tools and checklists, pre-emptive strengthening of intra-event components can help to reduce the impact of crises when they do occur.

In the patient safety paradigm, the Checklist for Assessing Institutional Resilience contains broad domains to help hospitals consider several concepts of resilience.^82^ It was not designed as a set of measures but may be considered a tool for systematic thinking in patient safety. Similarly, while some have suggested that the work-as-imagined versus work-as-done (WAI/WAD) ratio (or the more general actuality:potentiality ratio) may be used as a way to improve patient safety (by minimising performance variability),^17^ others highlighted that this approach may work for some simple processes, but is unlikely to result in favourable patient outcomes in unexpected or complex scenarios.^41^ For example, adherence to sepsis guidelines (WAI) is audited and associated with overall better outcomes,^83^ but depends on accurate diagnosis, which is itself difficult. Sepsis is frequently misdiagnosed (WAD), leading to wrong treatments, antimicrobial resistance and poor outcomes.^84 85^ Therefore, WAI/WAD is not an adequate approach to measure resilience. Instead, WAI/WAD should be used as means to understand the balance struck within systems, and a concept with which to frame observations about adaptations in healthcare. This reframes resilience from an outcome-oriented approach to a process-oriented one.

Some multi-level assessments of the resilience of critical infrastructures exist. The Tiered Approach by Linkov *et al* highlights three tiers of assessments focusing on relationships between components of a system.^86^ A key feature of this is the use of multiple tools for assessment at different levels and for different goals. For example, the Functional Resonance Analysis Method^87^ is useful for mapping relationships between components, while indices may help to align proposed changes with the overarching goals of the system. Checklists may be useful during a crisis. Modelling can help to predict consequences, audit progress and address slow variables. As such, our framework (figures 4 and 5, online supplemental appendix 3) and the related data extraction table (online supplemental appendix 4) provide examples of resilience frameworks mapped onto the time domain of crises. There are contemporary studies which seek to better understand multi-level influences specific to healthcare resilience.^76 77^

There seems to be limited focus on the measurement of post-event and trans-event domains. While quality improvement models tend to be embedded within healthcare organisations and systems, assessment of speed of recovery and robustness of interorganisational relationships rarely feature in assessment tools. Similarly, assessment of culture and leadership is challenging.^88^

## Discussion

We have employed a relatively new review methodology well-suited for complex topics informed by various research traditions. There is increasing acknowledgement of the need for interdisciplinary, cross-sectoral and multi-level approaches to healthcare resilience. Yet, its history has been one of multiple research traditions operating within specific paradigms. Global health paradigms dominate the macro-level, while DRR and patient safety frame the meso-level.

Definitions of healthcare resilience are increasingly broad. They have evolved from highly contextualised situations towards appreciation of different hierarchical levels and from an outcome-oriented understanding towards one which is more process-oriented. With this evolution, it has become more difficult to characterise failure of resilience. Even though healthcare systems and organisations are affected by a range of stressors, they are inherently resilient as essential societal services. Recognising the interplay between hierarchical levels is vital for building resilience. For example, the Institute for Healthcare Improvement framework for improving joy in work considers meso-level and macro-level aspects (eg, participative management and an environment of psychological safety), that affect micro-level resilience, and thus, explicitly links previously distinct hierarchical levels.^89^ Such multi-level frameworks rarely feature at the meso-level and macro-levels, despite healthcare workers being arguably the most important resources of healthcare systems. Several recent study protocols have been published to better understand multi-level influences in resilience, particularly in the patient safety paradigm.^11 64^

Put another way, resilience concepts at macro-level have tended to focus on physical capacities prior to COVID-19. Yet, there is increasing recognition of the importance of relational aspects of resilience, particularly across macro-level and meso-level boundaries. Some examples of these within the COVID-19 pandemic include the adoption of open leadership at meso-level and macro-levels,^58^ interorganisational relationships and their realignment,^4 60^ cultivating an environment of trust,^42 59^ and effective communication channels at micro-level, meso-level and macro-level.^90^ Open leadership styles gained more trust and enabled better understanding of WAI/WAD.^42^ Alongside, effective channels of communication between healthcare workers, managers and in turn national bodies helped with rapid adaptation and identification of acute needs, from equipment and supply issues to psychological support services.^58^

While a range of tools and indicators have been developed for physical capacities and infrastructure, it is still not known how best to assess the effectiveness of relational aspects of resilience. In particular, understanding the nature of interorganisational relationships seems to stand out as a research priority.

Our framework for healthcare resilience fulfils several needs in this field. First, it brings together knowledge and observations gained from a range of disciplines, across hierarchical levels, and integrates it with lessons learnt from the COVID-19 pandemic. It acknowledges and builds on historical health agendas across geopolitical and financial contexts (figure 3). Many of these have been centred in low/middle-income countries including WHO’s building blocks of health, health systems strengthening and DRR agendas (online supplemental appendix 3). As such, our framework may be applied to a wide range of health organisations and systems. It suggests that resilience requires simultaneous consideration of a diverse set of components but does not assert a particular component as most important. Second, it emphasises both physical and relational aspects of resilience (figures 4 and 5). Relational aspects such as cross-sector networks and collaborations feature in global health agendas developed primarily for low/middle-income countries but have hitherto received less attention in the indices applied to high income countries. Thus, different components of the framework may take priority for different systems at different points of time. Finally, it dovetails with our definition and achieves some convergence in a highly heterogenous field.

There are several strengths to this study. First, a review of reviews provides a broad overview of the topic across several geopolitical contexts. There is consolidation of knowledge across a wide range of disciplines and paradigms, enabling wider appreciation of the complexities of the topic and the many ways it has been approached. Second, a meta-narrative approach helps to explore historical trends and ongoing debates within a complex field. In addition to the consolidation of knowledge in the previous point, this approach also acknowledges the differences between paradigms and broadens the understanding of resilience (figure 3, online supplemental appendix 4). Third, we have taken a multi-level approach at meso-level and macro-level, which has hitherto been rare. These three points provide a map for healthcare workers and health systems managers to better understand the contexts within which each party operates.^3^ Fourth, we have highlighted several lessons learnt from the COVID-19 pandemic, which was a shock to entire healthcare systems across the world.

Several limitations persist. We do not focus on the micro-level, even though humans are undoubtedly the most important components of healthcare systems, both as staff and service-users. Empirical studies are excluded. This means that while we were able to maintain breadth, we inadvertently lost resolution. In addition, knowledge continues to be discovered and some will not have made it into reviews yet. Therefore, while this review of reviews is timely, we acknowledge that resilience is a dynamic process.^28^ Several important empirical studies are underway to unpick key relational factors between hierarchical levels and how they may support or hinder adaptive capacity.^11 76^ These studies should help to deepen understanding into healthcare resilience. We chose not to include grey literature due to volume of work, but this potentially excludes some important information from this review. Similarly, while the language restriction potentially imposes a selection bias, a significant proportion of included reviewed specifically focused on non-English speaking and/or low/middle-income countries. Frameworks can be considered tools for systematically approaching resilience but require adaptation towards individual situations (online supplemental appendix 4). Our framework does not provide guidance on which components should be prioritised by which health system at which point of time. This requires contextualised analyses and aligns with the related realist approach seeking to understand ‘what works, for whom, under what circumstances and how’.^24^

Future reviews should state clear research questions, adopt systematic search strategies and use robust data analyses techniques. These would maximise the quality of evidence generated. Future priorities arising from this research include the need to examine multi-level resilience with empirical studies,^11^ exploring how each level’s resilience contributes to other levels^3^ and gaining deeper understanding of the nature and extent of interorganisational relationships and how they affect resilience.

## Conclusion

Our review of reviews provides a broad overview of healthcare resilience. The meta-narrative approach adopted recognises the contribution from several disciplines and paradigms within this field. We have highlighted the tendency for studies to focus on individual hierarchical levels and limited timescales, but also acknowledge the need and current desire to develop more interdisciplinary, cross-sectoral and multi-level approaches to healthcare resilience. Our framework for healthcare resilience crosses hierarchical and time boundaries, providing a broad and reflexive overview of healthcare resilience. Understanding the balances between sometimes opposing concepts, and contrasting priorities of different professional groups or hierarchies, is key to building resilience for the future.

## Supplementary Material

Reviewer comments

Author's manuscript

## Data Availability

All data relevant to the study are included in the article or uploaded as supplementary information.

## References

[1] Barasa E, Mbau R, Gilson L. What is resilience and how can it be nurtured? A systematic review of empirical literature on organizational resilience. Int J Health Policy Manag 2018;7:491–503. 10.15171/ijhpm.2018.06 29935126 PMC6015506

[2] Oppenheim B, Gallivan M, Madhav NK, et al. Assessing global preparedness for the next pandemic: development and application of an epidemic preparedness index. BMJ Glob Health 2019;4:e001157. 10.1136/bmjgh-2018-001157 PMC6352812 30775006

[3] Tan MZY. Resilience is a dirty word: misunderstood, and how we can truly build it. Crit Care 2022;26:168. 10.1186/s13054-022-04040-x 35676690 PMC9175167

[4] Meyer D, Bishai D, Ravi SJ, et al. A checklist to improve health system resilience to infectious disease outbreaks and natural hazards. BMJ Glob Health 2020;5:e002429. 10.1136/bmjgh-2020-002429 PMC7409956 32759184

[5] Park JJH, Mogg R, Smith GE, et al. How COVID-19 has fundamentally changed clinical research in global health. Lancet Glob Health 2021;9:e711–20. 10.1016/S2214-109X(20)30542-8 33865476 PMC8049590

[6] Razai MS, Chaudhry UAR, Doerholt K, et al. Covid-19 vaccination hesitancy. BMJ 2021;373:n1138. 10.1136/bmj.n1138 34016653

[7] Pillay M, Morel G. Measuring resilience engineering: an integrative review and framework for bench-marking organisational safety. Safety 2020;6:37. 10.3390/safety6030037

[8] Curtis S, Fair A, Wistow J, et al. Impact of extreme weather events and climate change for health and social care systems. Environ Health 2017;16:128. 10.1186/s12940-017-0324-3 29219105 PMC5773887

[9] Greenhalgh T, Robert G, Macfarlane F, et al. Storylines of research in diffusion of innovation: a meta-narrative approach to systematic review. Soc Sci Med 2005;61:417–30. 10.1016/j.socscimed.2004.12.001 15893056

[10] Mondal S, Van Belle S, Maioni A. Learning from Intersectoral action beyond health: a meta-narrative review. Health Policy Plan 2021;36:552–71. 10.1093/heapol/czaa163 33564855 PMC8128009

[11] Aase K, Guise V, Billett S, et al. Resilience in healthcare (RiH): a longitudinal research programme protocol. BMJ Open 2020;10:e038779. 10.1136/bmjopen-2020-038779 PMC7592282 33109657

[12] on behalf of the RiH-team, Wiig S, Aase K, et al. Defining the boundaries and operational concepts of resilience in the resilience in healthcare research program. BMC Health Serv Res 2020;20. 10.1186/s12913-020-05224-3 PMC7168985 32306981

[13] Li L, Liao S, Yuan J, et al. Analyzing healthcare facility resilience: scientometric review and knowledge map: Front Public Health 2021;9. 10.3389/fpubh.2021.764069 PMC8606559 34820352

[14] Biddle L, Wahedi K, Bozorgmehr K. Health system resilience: a literature review of empirical research. Health Policy Plan 2020;35:1084–109. 10.1093/heapol/czaa032 32529253 PMC7553761

[15] Koeva S, Rohova M, et al, Department of Health Economics and Management, Faculty of Public Health, Medical University-Varna, Bulgaria. Health system resilience: concept development. JofIMAB 2020;26:3251–8. 10.5272/jimab.2020263.3251

[16] Zhong S, Clark M, Hou X-Y, et al. Development of hospital disaster resilience: conceptual framework and potential measurement. Emerg Med J 2014;31:930–8. 10.1136/emermed-2012-202282 24028975

[17] Son C, Sasangohar F, Neville T, et al. Investigating resilience in emergency management: an integrative review of literature. Appl Ergon 2020;87:103114. 10.1016/j.apergo.2020.103114 32501246

[18] Almedom AM. Resilience research and policy/practice discourse in health, social, behavioral, and environmental sciences over the last ten years. Afr Health Sci 2008;8 Suppl 1:S5–13. 21448375 PMC3060725

[19] Calkins J. Moving forward after Sendai: how countries want to use science, evidence and technology for disaster risk reduction. PLoS Curr 2015;7:ecurrents.dis.22247d6293d4109d09794890bcda1878. 10.1371/currents.dis.22247d6293d4109d09794890bcda1878 26463730 PMC4504499

[20] McClelland AG, Shaw D, O’Grady N, et al. Recovery for development: a multi-dimensional, practice-oriented framework for transformative change post-disaster. J Dev Stud 2023;59:1–20. 10.1080/00220388.2022.2130055

[21] Conti A, Valente M, Paganini M, et al. Knowledge gaps and research priorities on the health effects of heatwaves: a systematic review of reviews. Int J Environ Res Public Health 2022;19:5887. 10.3390/ijerph19105887 35627424 PMC9140727

[22] Ries M. The COVID-19 Infodemic: mechanism, impact, and counter-measures—A review of reviews. Sustainability 2022;14:2605. 10.3390/su14052605

[23] Gough D. Meta-narrative and realist reviews: guidance, rules, publication standards and quality appraisal. BMC Med 2013;11:22. 10.1186/1741-7015-11-22 23360691 PMC3606472

[24] Wong G, Greenhalgh T, Westhorp G, et al. RAMESES publication standards: meta-narrative reviews. BMC Med 2013;11:20. 10.1186/1741-7015-11-20 23360661 PMC3558334

[25] Tan M, Prager G. Resilience in Healthcare: a meta-narrative review of reviews. Crd42022314729: PROSPERO; 2022. Available: https://www.crd.york.ac.uk/prospero/display_record.php?ID=CRD42022314729

[26] Page MJ, McKenzie JE, Bossuyt PM, et al. The PRISMA 2020 statement: an updated guideline for reporting systematic reviews. BMJ 2021;372:n71. 10.1136/bmj.n71 33782057 PMC8005924

[27] Gurney A. Resilience in the UK and other OECD economies: Treasury economic working paper No.2. London HM Treasury; 2008. Available: https://webarchive.nationalarchives.gov.uk/ukgwa/20081112160625/http:/www.hm-treasury.gov.uk/d/bud08_workingpaper2_557_.pdf

[28] Bealt J, Powell D, Shaw D. Benefits of involving public involvement in emergency planning. Manchester University of Manchester; 2021. Available: https://committees.parliament.uk/writtenevidence/21957/pdf

[29] Abdullah MA, Shaikh BT. Confusion and denial: need for systems thinking to understand the HIV epidemic in Pakistan. J Ayub Med Coll Abbottabad 2014;26:396–400. 25671957

[30] Butler A, Hall H, Copnell B. A guide to writing a qualitative systematic review protocol to enhance evidence-based practice in nursing and health care. Worldviews Evid Based Nurs 2016;13:241–9. 10.1111/wvn.12134 26790142

[31] Smith V, Devane D, Begley CM, et al. Methodology in conducting a systematic review of systematic reviews of healthcare interventions. BMC Med Res Methodol 2011;11:15. 10.1186/1471-2288-11-15 21291558 PMC3039637

[32] Aromataris E, Fernandez R, Godfrey CM, et al. Summarizing systenatic reviews: methodological development, conduct and reporting of an umbrella review approach. Int J Evid Based Healthc 2015;13:132–40. 10.1097/XEB.0000000000000055 26360830

[33] Noble H, Mitchell G. What is grounded theory. Evid Based Nurs 2016;19:34–5. 10.1136/eb-2016-102306 26872777

[34] Brand FS, Jax K. Focusing the Meaning(S) of resilience: resilience as a descriptive concept and a boundary object. E&S 2007;12. 10.5751/ES-02029-120123

[35] Turenne CP, Gautier L, Degroote S, et al. Conceptual analysis of health systems resilience: a scoping review. Soc Sci Med 2019;232:168–80. 10.1016/j.socscimed.2019.04.020 31100697

[36] Hosseini S, Barker K, Ramirez-Marquez JE. A review of definitions and measures of system resilience. Reliab Eng Syst Saf 2016;145:47–61. 10.1016/j.ress.2015.08.006

[37] Augustynowicz A, Opolski J, Waszkiewicz M. Resilient health and the healthcare system. A few introductory remarks in times of the COVID-19 pandemic. Int J Environ Res Public Health 2022;19:3603. 10.3390/ijerph19063603 35329289 PMC8953726

[38] Biggs R, Schlüter M, Schoon ML. Principles for Building Resilience. Cambridge University Press, 2015. 10.1017/CBO9781316014240

[39] Florez Jiménez MP, Muñoz Villamizar AF, Lleo A. Exploring the relationship between sustainability, resilience, and purpose in the context of corporations: a comprehensive literature review. SSRN Journal 2021. 10.2139/ssrn.3944148

[40] Hollnagel E, Wears R, Braithwaite J. From safety-I to safety-II: A white paper; 2015.

[41] Berg SH, Akerjordet K, Ekstedt M, et al. Methodological strategies in resilient health care studies: an integrative review. Safety Science 2018;110:300–12. 10.1016/j.ssci.2018.08.025

[42] Rangachari P, L Woods J. Preserving organizational resilience, patient safety, and staff retention during COVID-19 requires a holistic consideration of the psychological safety of healthcare workers. Int J Environ Res Public Health 2020;17:4267. 10.3390/ijerph17124267 32549273 PMC7345925

[43] Stoverink AC, Kirkman BL, Mistry S, et al. Bouncing back together: toward a theoretical model of work team resilience. AMR 2020;45:395–422. 10.5465/amr.2017.0005

[44] Ayuningtyas D, Windiarti S, Hadi MS, et al. Disaster preparedness and mitigation in Indonesia: a narrative review. Iran J Public Health 2021;50:1536–46. 10.18502/ijph.v50i8.6799 34917524 PMC8643537

[45] Romanello M, McGushin A, Di Napoli C, et al. The 2021 report of the lancet countdown on health and climate change: code red for a healthy future. Lancet 2021;398:1619–62. 10.1016/S0140-6736(21)01787-6 34687662 PMC7616807

[46] Kruk ME, Ling EJ, Bitton A, et al. Building resilient health systems: a proposal for a resilience index. BMJ 2017;357:j2323. 10.1136/bmj.j2323 28536191

[47] Carrington MA, Ranse J, Hammad K. The impact of disasters on emergency department resources: review against the Sendai framework for disaster risk reduction 2015-2030. Australas Emerg Care 2021;24:55–60. 10.1016/j.auec.2020.09.003 33032978

[48] Cartwright C, Hall M, Lee ACK. The changing health priorities of earthquake response and implications for preparedness: a scoping review. Public Health 2017;150:60–70. 10.1016/j.puhe.2017.04.024 28645042

[49] Challen K, Lee AC, Booth A, et al. Where is the evidence for emergency planning: a scoping review. BMC Public Health 2012;12. 10.1186/1471-2458-12-542 PMC3438123 22823960

[50] Fallah-Aliabadi S, Ostadtaghizadeh A, Ardalan A, et al. Towards developing a model for the evaluation of hospital disaster resilience: a systematic review. BMC Health Serv Res 2020;20:64. 10.1186/s12913-020-4915-2 31996213 PMC6988294

[51] Luke J, Franklin R, Aitken P, et al. Safer hospital infrastructure assessments for socio-natural disaster - A scoping review. Prehosp Disaster Med 2021;36:627–35. 10.1017/S1049023X21000650 34284848

[52] Xin YT, Xu KY. Hospital emergency management research in China: trends and challenges. Emerg Med J 2012;29:353–7. 10.1136/emermed-2011-200512 22052954

[53] Nuzzo JB, Meyer D, Snyder M, et al. What makes health systems resilient against infectious disease outbreaks and natural hazards? Results from a scoping review. BMC Public Health 2019;19. 10.1186/s12889-019-7707-z PMC6798426 31623594

[54] Iflaifel M, Lim RH, Ryan K, et al. Resilient health care: a systematic review of conceptualisations, study methods and factors that develop resilience. BMC Health Serv Res 2020;20:324. 10.1186/s12913-020-05208-3 32303209 PMC7165381

[55] Pariès J, Wreathall J, Hollnagel E, et al. RAG–the resilience analysis grid. Resilience engineering in practice. CRC Press, 2017: 275–96. 10.1201/9781317065265

[56] WHO. Monitoring the building blocks of health systems; 2010.

[57] Tessema GA, Kinfu Y, Dachew BA, et al. The COVID-19 pandemic and healthcare systems in Africa: a scoping review of preparedness, impact and response. BMJ Glob Health 2021;6:e007179. 10.1136/bmjgh-2021-007179 PMC8637314 34853031

[58] Turner S, Botero-Tovar N, Herrera MA, et al. Systematic review of experiences and perceptions of key actors and organisations at multiple levels within health systems internationally in responding to COVID-19. Implement Sci 2021;16:50. 10.1186/s13012-021-01114-2 33962635 PMC8103061

[59] Alami H, Lehoux P, Fleet R, et al. How can health systems better prepare for the next pandemic? Lessons learned from the management of COVID-19 in Quebec (Canada). Front Public Health 2021;9:671833. 10.3389/fpubh.2021.671833 34222176 PMC8249772

[60] Grimm PY, Oliver S, Merten S, et al. Enhancing the understanding of resilience in health systems of low- and middle-income countries: a qualitative evidence synthesis. Int J Health Policy Manag 2022;11:899–911. 10.34172/ijhpm.2020.261 33619924 PMC9808204

[61] Kamara JK, Akombi BJ, Agho K, et al. Resilience to climate-induced disasters and its overall relationship to well-being in Southern Africa: a mixed-methods systematic review. Int J Environ Res Public Health 2018;15:2375. 10.3390/ijerph15112375 30373194 PMC6267582

[62] Tippong D, Petrovic S, Akbari V. A review of applications of operational research in healthcare coordination in disaster management. Eur J Oper Res 2022;301:1–17. 10.1016/j.ejor.2021.10.048 34728892 PMC8552591

[63] Van Ryneveld M, Schneider H, Lehmann U. Looking back to look forward: a review of human resources for health governance in South Africa from 1994 to 2018. Hum Resour Health 2020;18:92. 10.1186/s12960-020-00536-1 33243260 PMC7689387

[64] Anderson JE, Aase K, Bal R, et al. Multilevel influences on resilient Healthcare in six countries: an international comparative study protocol. BMJ Open 2020;10:e039158. 10.1136/bmjopen-2020-039158 PMC7722365 33277279

[65] Health Foundation. Quality improvement made simple: what everyone should know about health care quality improvement [The Health Foundation]. 2021. Available: https://www.health.org.uk/sites/default/files/QualityImprovementMadeSimple.pdf

[66] Rezaei F, Maracy MR, Yarmohammadian MH, et al. Hospitals preparedness using WHO guideline: a systematic review and meta-analysis. Hong Kong J Emerg Med 2018;25:211–22. 10.1177/1024907918760123

[67] Ayanore MA, Amuna N, Aviisah M, et al. Towards resilient health systems in sub-Saharan Africa: a systematic review of the English language literature on health workforce, surveillance, and health governance issues for health systems strengthening. Ann Glob Health 2019;85:113. 10.5334/aogh.2514 31418540 PMC6696789

[68] Hasan MZ, Neill R, Das P, et al. Integrated health service delivery during COVID-19: a scoping review of published evidence from low-income and lower-middle-income countries. BMJ Glob Health 2021;6:e005667. 10.1136/bmjgh-2021-005667 PMC8210663 34135071

[69] Ravi SJ, Meyer D, Cameron E, et al. Establishing a theoretical foundation for measuring global health security: a scoping review. BMC Public Health 2019;19:954. 10.1186/s12889-019-7216-0 31315597 PMC6637489

[70] Fridell M, Edwin S, von Schreeb J, et al. Health system resilience: what are we talking about? A scoping review mapping characteristics and keywords. Int J Health Policy Manag 2020;9:6–16. 10.15171/ijhpm.2019.71 31902190 PMC6943300

[71] Blumenstock J, Bakker G, Jarris PE. Measuring preparedness: the national health security preparedness index. J Public Health Manag Pract 2014;20:361–3. 10.1097/PHH.0000000000000073 24667201

[72] Haldane V, Zhang Z, Abbas RF, et al. National primary care responses to COVID-19: a rapid review of the literature. BMJ Open 2020;10:e041622. 10.1136/bmjopen-2020-041622 PMC7725079 33293398

[73] Lapão LV, Silva A, Pereira N, et al. Ebola impact on African health systems entails a quest for more International and local resilience: the case of African Portuguese speaking countries. Pan Afr Med J 2015;22 Suppl 1:15. 10.11694/pamj.supp.2015.22.1.6653 PMC4695520 26740843

[74] Tin N, Lwin S, Kyaing NN, et al. An approach to health system strengthening in the Union of Myanmar. Health Policy 2010;95:95–102. 10.1016/j.healthpol.2009.11.013 20015569

[75] Sanderson M, Allen P, Osipovic D, et al. The developing architecture of system management: integrated care systems and sustainability and transformation partnerships - Interim report: Prucomm; 2021. Available: https://prucomm.ac.uk/assets/uploads/PRUComm_ICS_study_interim_report_feb_2021.pdf

[76] Anderson JE, Aase K, Bal R, et al. Multilevel influences on resilient healthcare in six countries: an international comparative study protocol. BMJ Open 2020;10:e039158. 10.1136/bmjopen-2020-039158 PMC7722365 33277279

[77] Petersen EE, Lyng HB, Ree E, et al. Relationship between management and resilience in healthcare: a study protocol for a systematic review. BMJ Open 2021;11:e047855. 10.1136/bmjopen-2020-047855 PMC8291312 34281923

[78] Baum F, Freeman T, Musolino C, et al. Explaining COVID-19 performance: what factors might predict national responses BMJ 2021;372:n91. 10.1136/bmj.n91 33509924 PMC7842256

[79] Bell JA, Nuzzo JB. Global health security index: advancing collective action and accountability amid global crisis: NTI/John Hopkins center for health security; 2021. Available: www.GHSIndex.org

[80] Rameshshanker V, Wyngaarden S, Lau LL, et al. Health system resilience to extreme weather events in Asia-Pacific: a scoping review. Clim Dev 2021;13:944–58. 10.1080/17565529.2020.1870425

[81] Peñaloza GA, Saurin TA, Formoso CT, et al. A resilience engineering perspective of safety performance measurement systems: a systematic literature review. Safety Science 2020;130:104864. 10.1016/j.ssci.2020.104864

[82] Carthey J, de Leval MR, Reason JT. Institutional resilience in healthcare systems. Qual Health Care 2001;10:29–32. 10.1136/qhc.10.1.29 11239141 PMC1743425

[83] Evans L, Rhodes A, Alhazzani W, et al. Surviving sepsis campaign: International guidelines for management of sepsis and septic shock 2021. Intensive Care Med 2021;47:1181–247. 10.1007/s00134-021-06506-y 34599691 PMC8486643

[84] Fitzpatrick F, Tarrant C, Hamilton V, et al. Sepsis and antimicrobial stewardship: two sides of the same coin. BMJ Qual Saf 2019;28:758–61. 10.1136/bmjqs-2019-009445 PMC6860726 31018985

[85] Vincent JL. The clinical challenge of sepsis identification and monitoring. PLoS Med 2016;13:e1002022. 10.1371/journal.pmed.1002022 27187803 PMC4871479

[86] Linkov I, Trump BD, Trump J, et al. Resilience stress testing for critical infrastructure. IJDRR 2022;82:103323. 10.1016/j.ijdrr.2022.103323

[87] Patriarca R, Di Gravio G, Woltjer R, et al. Framing the FRAM: a literature review on the functional resonance analysis method. Safety Science 2020;129:104827. 10.1016/j.ssci.2020.104827

[88] Njah J, Hansoti B, Adeyami A, et al. Measuring for success: evaluating leadership training programs for sustainable impact. Ann Glob Health 2021;87:63. 10.5334/aogh.3221 34307066 PMC8284530

[89] Smith AF, Plunkett E. People, systems and safety: resilience and excellence in healthcare practice. Anaesthesia 2019;74:508–17. 10.1111/anae.14519 30585298 PMC6766951

[90] Raymond CB, Ward PR. Community-level experiences, understandings, and responses to COVID-19 in low-and middle-income countries: a systematic review of qualitative and ethnographic studies. Int J Environ Res Public Health 2021;18:22. 10.3390/ijerph182212063 PMC8621360 34831831

