# Supplementary material for: Healthcare resilience: a meta-narrative systematic review and synthesis of reviews
Source: BMJ Open. 2023 Sep 20;13(9):e072136. doi: 10.1136/bmjopen-2023-072136 (PMC10514640; doi:10.1136/bmjopen-2023-072136)
Supplement: Supplementary data [file bmjopen-2023-072136supp001.pdf]

Supplementary file

Healthcare resilience - A meta-narrative systematic review and synthesis of reviews

List of supplementary materials

Appendix 1: Additional details of methodology
Appendix 2: Definition word cloud and word occurrence table
Appendix 3: Data extraction table from reviews
Appendix 4: Healthcare resilience framework summary

Tan MZY, *et al. BMJ Open* 2024; 13:e072136. doi: 10.1136/bmjopen-2023-072136

Appendix 1: Additional methodological details

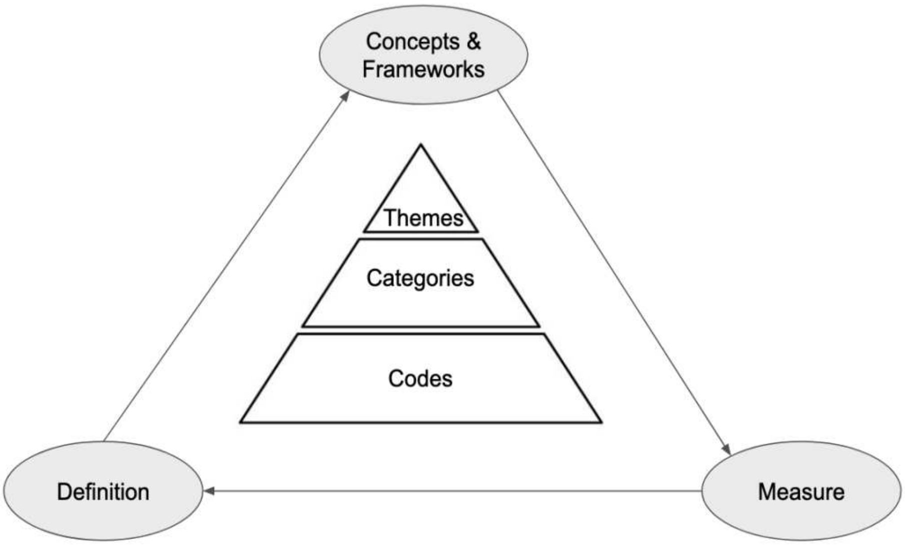

Appendix 1.1: Theoretical framework describing three tiers of analysis within three foci.

In choosing our approach, we referred to Xiao et al.'s typology of reviews [1]. We sought to describe and extend the current understanding of healthcare resilience. Since resilience has been studied within a range of academic disciplines, across multiple sectors and a numerous hierarchies and levels, a meta-narrative was the most appropriate form of review to perform. This aims to consolidate the various research traditions involved in the study of a broad topic, thereby forming an overarching synthesis of the landscape of said topic [2]. A related method is the realist review, which seeks to distil circumstantial conditions conferring success of various interventions [3]. Traditional systematic reviews or meta-analyses were unsuitable methodologies for this study due to several reasons. In particular, studies into resilience include multiple methodologies, both quantitative and qualitative, over a span of disciplines which include evolving terminology and nomenclature. The homogeneity required for a traditional systematic review or quantitative measures which form the basis of a meta-analysis do not feature in resilience studies [4 5].

The key principles of meta-narrative reviews are pragmatism, pluralism,

historicity, contestation, reflexivity and peer review [6]. This review of reviews provides a multidisciplinary and multi-level view of healthcare resilience. It maps current understanding and presents various conceptualisations and frameworks (pluralism). This will thus be useful for a multitude of actors including healthcare leaders, policymakers and academics (pragmatism). Examining the various research traditions which converge in this topic, it will both chart its evolution and explore various perspectives (historicity). Differences in definitions, measures and conceptualisations will be highlighted and discussed (contestation). The protocol is updated according to the findings, and future agendas are proposed in line with emergent data and insights (reflexivity). Finally, independent reviewers are involved throughout the process, while results are shared with a broader research team focusing on whole-of-society resilience (peer review).

Tool or method	Reason for unsuitability
Cochrane protocol [7]	Requires established quantitative metrics
Cochrane qualitative tool [8]	Only applicable for empirical studies. Unsuitable for mixed or multi-method studies
Mixed Methods Assessment Tool (MMAT)[9]	Heavily weighted towards quantitative studies. Many studies in healthcare resilience are qualitative in nature. Sections on randomisation are irrelevant for this topic.
Critical Appraisal Skills Programme (CASP) checklist [10]	Deals with systematic reviews mainly. This precludes many of the reviews within healthcare resilience.
Quality Assessment of Diverse Studies (QuADS)[11]	Targeted at empirical studies. Many components are difficult to apply to reviews.
AMSTAR2 tool [12]	Focused on healthcare interventions, centred around (population, intervention, control group and outcome) PICO components

Appendix 1.2: Table of critical appraisal tool examined and why they were deemed unsuitable for this study.

Appendix 2: Definitions - word cloud and word occurrence table

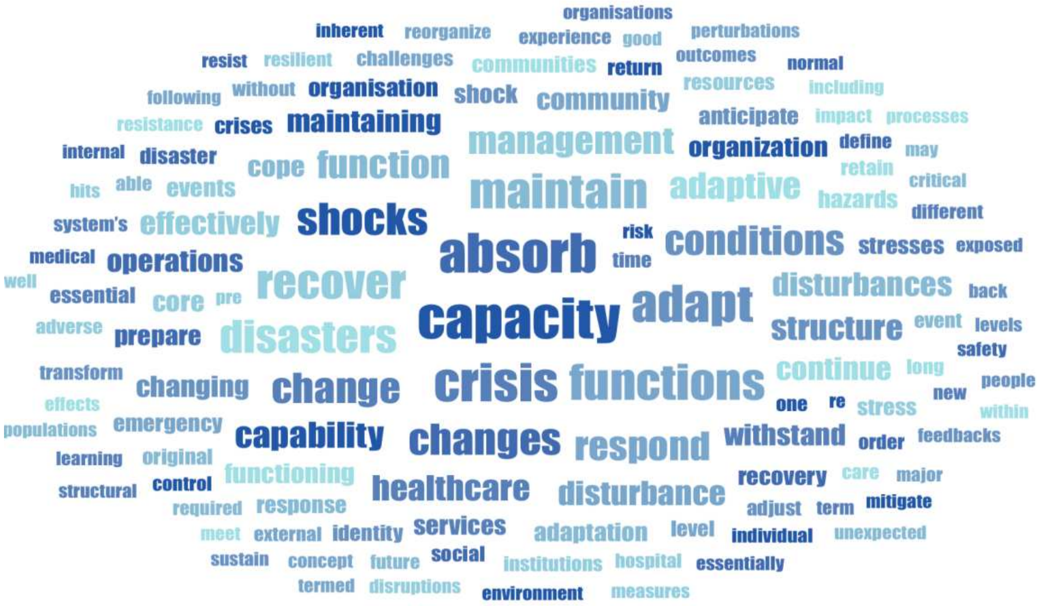

Appendix 2.1: Word occurrence cloud of definitions. Larger words represent increased frequency.

Healthcare Resilience Definition: Word occurrences by timeframe

Pre-event		Intra-event		Post-event		Referent events	
Word/Synonym	Frequency	Word/Synonym	Frequency	Word/Synonym	Frequency	Word/Synonym	Frequency
Prepare	17	Absorb	40	Recovery	42	Crisis	53
Anticipate	10	Adapt	64	Return	15	Shocks/Impacts	41
Expect	5	Capabilities/ Capacities	83	Learning	11	Changes	51
Resource	9	Respond	34	Improvement	9	Disturbance/ Disruption	34
		Maintain	61	Transform	19	Disaster	37
		Essential services	33	Future	7	Events	19
		Essential functions	64			Adversity	12
		Manage	23			Challenges	8
		Mitigate	23			Emergencies	10
		Resist	13			Hazards	11
		Withstand	13				
		Adjust	8				
		Cope	12				

Appendix 2.2: Word occurrence table.

Tan MZY, *et al. BMJ Open* 2024; 13:e072136. doi: 10.1136/bmjopen-2023-072136

Appendix 3: Data extraction table from reviews

Author, Year Country of 1st author	Title of study	Hierarchical level	Research paradigm	Geographic focus	Study design	Brief summary
Alami et al., 2021 [13] Canada	How Can Health Systems Better Prepare for the Next Pandemic? Lessons Learned From the Management of COVID-19 in Quebec (Canada)	Macro	Public health	Quebec, Canada	Narrative review and survey	Took a capacity-focused approach to resilience. Concepts include: Insufficient workforce in several settings, need to ensure training, safety and protection, misaligned policies, shortage of equipment and drugs, ethical dilemmas and utilitarianism, importance of high-level communication, managing media communications
Augustynowicz et al., 2022 [14] Poland	Resilient Health and the Healthcare System. A Few Introductory Remarks in Times of the COVID-19 Pandemic	Macro	Patient safety, theoretical	International	Narrative review	Describes definition and scope issues. Covers definitions from several paradigms. Lists some of the evolution of thinking in resilience. Includes urban and community resilience which is outside the scope of our review
Ayanore et al., 2019 [15] Ghana	Towards Resilient Health Systems in Sub-Saharan Africa: A Systematic Review of the English Language Literature on Health Workforce, Surveillance, and Health Governance Issues for Health Systems Strengthening	Macro	Global health - HSS	Sub-Saharan Africa	Literature review	Assessed 3 out of 6 elements of WHO building blocks of health in Sub-Saharan Africa. Presents lessons learnt from studies focused mainly on infectious diseases outbreaks and epidemics. Key concepts include leadership and physical resource, including equipment, funding and workforce. Some studies highlight the importance of inter-organisational relationships and effective communication
Banwell et al., 2018 [16] Australia	Towards improved linkage of disaster risk reduction and climate change adaptation in health: A review	Meta	Disaster and Climate change adaptation	International	Literature review	Increasing links in literature between DRR and climate change adaptation. Refers to SFDRR. Highlights the need for cross-sectoral work between DRR, climate science and healthcare to build future resilience for health systems
Barasa et al., 2018 [17] UK	What is resilience and how can it be nurtured? A systematic review of empirical literature on organizational resilience	Meso/Macro	Global health	International	Literature review	Key concepts: Material resources, preparedness & planning, information management, collateral pathways and redundancies, governance processes, leadership, organisational culture, human capital, social networks and collaboration
Berg et al., 2018 [18] Norway	Methodological strategies in resilient health care studies: An integrative review	Meso	Resilience engineering	International	Integrative review	All studies included used qualitative methodology to study resilience. Concepts: individual strategies, sense-making, decision-making, performance variability and expertise

Tan MZY, *et al. BMJ Open* 2024; 13:e072136. doi: 10.1136/bmjopen-2023-072136

**Biddle et al., 2020 [19] Germany**	Health system resilience: A literature review of empirical research	Macro	Global health	International	Narrative review	Refers to the balance between an outcome-oriented understanding vs a process-oriented understanding of resilience, taken from Blanchet et al. [20]. Public health studies categorised into three groups: (1) Quantitative studies on service delivery, (2) Qualitative studies focusing on HRH, (3) Broader perspectives: building blocks, etc.<br><br>Macro-level Resilience informed by two discourses: disaster management and healthcare quality (patient safety) and resilience engineering.<br>DRR perspectives have shifted after hurricane Catrina in USA toward community resilience
**Blumenstock et al., 2014 [21] USA**	Measuring Preparedness: The National Health Security Preparedness Index	Macro	Public health	USA	Policy document	Presents the National Health Security Preparedness Index:<br>Health surveillance - surveillance and epidemiology investigation, environmental and biological monitoring, laboratory testing<br>Community planning and engagement - cross-sector collaboration, at-risk populations, spontaneous volunteers, social capital and cohesions<br>Incident and information management - multi-agency coordination, emergency public information and warning<br>Surge management - emergency medical services, acute & primary care, mental healthcare<br>Countermeasure management - medical supply chain and distribution, countermeasure utilisation and effectiveness"
**Brand & Jax, 2007 [22] Germany**	Focusing the Meaning(s) of Resilience: Resilience as a Descriptive Concept and a Boundary Object	Meso/Macro	Theoretical	International	Literature review	Focuses on definitions of resilience. 3 categories (descriptive, hybrid and normative). 10 classes and 10 definitions: Original ecological, extended ecological, systematic-heuristic, operational, sociological, ecological-economic, ecosystem-services-related, social-ecological system, metaphoric, sustainability-related.<br><br>Stresses the importance of determining resilience "of what, to what" to operationalise
**Burke et al., 2021 [23] Ireland**	Building health system resilience through policy development in response to COVID-19 in Ireland: From shock to reform	Macro	Public health - COVID	Ireland	Literature review of health policies	States that the Irish government made several changes to increase healthcare access during the COVID19 pandemic. Argues that existing policies/frameworks should not be abandoned in a pandemic, but authorities should focus on those aspects within the used frameworks that contribute to strengthening and dealing with the acute shock. Views resilience as a transformative process in response to crisis
**Carrington et al., 2021 [24] Australia**	The impact of disasters on emergency department resources: review against the Sendai framework for disaster risk reduction 2015–2030	Meso	Global health - Disaster	International	Integrative review	Uses SFDRR to assess emergency departments. Identified points for improvement based on SFDRR framework

**Cartwright et al., 2017** [25]<br>**UK**	The changing health priorities of earthquake response and implications for preparedness: a scoping review	Macro	Global health - DRR	LMICs	Scoping review	Mapped the needs in physical health, mental health and health care to disaster cycle. Categorised according to time frame after the event.<br>Preparedness: disconnect between planning and policy, importance of leadership. Acute phase: women, children and elderly disproportionately affected, trauma to extremities, spinal cord injuries, wound debridement, perinatal issues, lack of specialists, physical equipment and lack of workforce, transport systems, coordination difficulties, lack of integration of information systems and international staff. Response: general health needs, respiratory, ocular, GI and dermatology problems, wound infections, late presentations, cold supply chain failure, increased suicides, staff mental health, importance of leadership and coordination, utilitarianism, surveillance. Chronic phase: rehabilitation, unwanted pregnancies, worsening burden of chronic diseases, PTSD and mental health, psychological support for victims and staff<br><br>Mapped lessons onto disaster management cycle. Plan: Policy, address inequalities, surveillance and long-term monitoring, current service gaps, maintenance of essential services, surge capacity, record systems, international staff, logistics, changing health priorities. Organise and equip: medication, equipment and blood, supply points, transportation, donations. Train: disaster mangement, leadership. Exercise: regular exercises to validate plans and arrangements. Evaluate and improve
**Challen et al., 2012** [26]<br>**UK**	Where is the evidence for emergency planning: A scoping review	Macro	Emergency management	HICs	Scoping review	Makes use of FEMA emergency cycle. Key aspects: Mitigation, hazard analysis, capability assessment, emergency planning, capability maintenance, emergency response, recovery, development plans, communications/mass media, informatics and intelligence, other organisational issues
**Curtis et al., 2017** [27]<br>**UK**	Impact of extreme weather events and climate change for health and social care systems	Macro	Climate	UK	Literature review	Identified need to improve hospital infrastructure, wider social infrastructure and collective responsibility towards resilience approach, cross-sectoral work and integration of planning and preparedness.<br><br>Concepts categorised into three domains: Physical (built infrastructure: health facilities, utilities), Institutional (policies and practices, human resources, professional practice), Social (community groups and informal networks)
**Fallah-Aliabadi et al., 2020** [28]<br>**Iran**	Towards developing a model for the evaluation of hospital disaster resilience: A systematic review	Meso	Disaster management	International	Literature review	Assessed several frameworks for disaster resilience: Hospital Safety Index (HSI), Dynamic approach to seismic resilience of hospitals, Measuring framework of the hospital resilience, Indicators for assessing hospital disaster preparedness in Japan.<br><br>Concepts categorised into domains (with 27 sub-domains): Constructive (stability, design, architectural, transportation and transition system<br>Infrastructural: power, water and sewage, communication and IT, Heating, ventilation and air conditioning (HVAC), fuel, medical gas, equipment and furniture, hazardous material, fire system), Administrative (disaster plan, risk assessment and reduction, response, command, coordination, evacuation, need assessment, logistic and supplies, safety committee, continuity of services, volunteers, finance, recover, training)

Tan MZY, *et al. BMJ Open* 2024; 13:e072136. doi: 10.1136/bmjopen-2023-072136

**Fridell et al., 2020 [29] Sweden**	Health System Resilience: What Are We Talking About? A Scoping Review Mapping Characteristics and Keywords	Macro	Global health	International	Scoping review + expert consultatio n	Narrative presentation of definitions of resilience.<br><br>Key features of resilience were mapped onto WHO building blocks of health framework: Leadership and governance (ethics, system capacity, operationalisation, engagement, institutional design), information (surveillance, policy making, evaluation), health workforce (diversity and flexibility, limited resources, incentive, community involvement), financing (resource allocation, funding mechanisms, costing), medicines (sustainability), service delivery (response, prevention)
**Grimm et al., 2021 [30] Switzerland**	Enhancing the Understanding of Resilience in Health Systems of Low- and Middle-Income Countries: A Qualitative Evidence Synthesis	Macro	Global health	LMIC	Literature review + interviews	Maps Kruk's Resilience Index [31] onto WHO's building blocks of health [32]. Refined concepts from Resilience Index: Realigned relationships (micro-, meso- and macro-level). Public/private partnerships), foresight and motivation (Investment into slow variables, motivation: political will and individual commitment), change management (integrated change management systems and processes help to confer resilience), emergency preparedness
**Haldane et al., 2020 [33] Canada**	National primary care responses to COVID-19: a rapid review of the literature	Macro	Global health	International	Rapid review	Key lessons from COVID19: Surge capacity should be strengthened. Need to ensure essential services continuity. National guidance needed on biological sampling handling. Most systems increased their telemedicine capabilities
**Hasan et al., 2021 [34] Canada**	Integrated health service delivery during COVID-19: A scoping review of published evidence from low-income and lower-middle-income countries	Macro	Global health (UHC)	LMIC	Scoping review	Assessment of L-LMIC's ability for integrated health service delivery during COVID19 according to WHO building blocks and pandemic continuum.<br><br>Key recommendations: Strengthening of coordination between healthcare bodies at various levels, cross-sectoral collaboration between government, private sector, media, and armed forces. Establishing integrated platforms for testing, health infrastructure to scale-up (redundancies). Empowering communities to engaged in disease prevention and containment. Expansion of digital health technologies. Build resilience by investing in Primary Healthcare and Integrated Health Service Delivery
**Hosseini et al., 2016 [35] USA**	A review of definitions and measures of system resilience	Meso	Engineering	International	Literature review	Presents a narrative of various definitions. Considers differences in organisational, social, economic, and engineering domains. Reliability is often used in engineered systems. There is multidimensionality in some definitions Ongoing issues with definitions include uncertain mechanisms to achieve resilience, the role of recovery, and the need to return to steady-state.<br><br>Presents a summary of how studies measure resilience. Qualitative: conceptual framework, semi-quantitative. Quantitative: generic resilience metrics, structural-based modelling
**Iflaifel et al., 2020 [36] UK**	Resilient Health Care: a systematic review of conceptualisations, study methods and factors that develop resilience	Meso/Macro	Patient safety	International	Literature review	Studies used a mix of qualitative and quantitative methods to study resilience.<br><br>Key concepts: Teamwork, in-situ practical experience, exposure to diverse views and perspectives, trade-offs, protocols & checklists, system design, workarounds

**Jesus et al., 2021 [37]**<br>**Inda**	PREparedness, REsponse and SySTemic transformation (PRE-RE-SyST): a model for disability-inclusive pandemic responses and systemic disparities reduction derived from a scoping review and thematic analysis	Macro	Disability studies	International	Scoping review	Focused on inclusive approach to pandemic responses to reduce health inequalities. Key concepts: transform societies' cultural assumptions about disability. Design systems and policies for structural disability-inclusiveness. Prepare inter-sectoral, disability-inclusive pandemic preparedness. Reduce inequalities. Rapid and compassionate responses in residential and long-term care settings. Provide accessibility
**Koeva & Rohova, 2020 [38]**<br>**Bulgaria**	Health System Resilience: Concept Development	Micro/Meso/ Macro	Theoretical	International	Literature review	Narrative presentation of quotations of definitions. Dictionaries distinguish between physical objects and innate human capacity. Engineering: resist shocks, absorb and hold elastic strain before permanent distortion or failure. Psychology: copes and maintains function in face of adversity, trauma, tragedy or stress. Resilience as an outcome, a process, or even contains both dynamic and static properties (within community resilience).<br><br>Resilience is undergoing evolution from outcome-oriented to process-oriented. There is a dynamically to the scope, breadth and depth of definitions. Conceptualisations are increasingly broad and complex. Existence of dynamism, uncertainty, non-linearity and unpredictability.
**Kruk et al., 2017 [31]**<br>**USA**	Building resilient health systems: a proposal for a resilience index	Macro	Global health	LMICs	Case studies/ review + framework proposal	Defines resilience as "the capacity of health actors, institutions, and populations to prepare for and effectively respond to crises; maintain core functions when a crisis hits; and, informed by lessons learnt during the crisis, reorganise if conditions require it."<br><br>Key domains: aware (capacity, risks and communication), diverse (meets range of needs, adequate financing), self-regulating (maintain core services and leverage collaborations), integrated (coordination and community engagement), adaptive (distributive decision-making, flexible spending and evaluation for improvement)
**Lapão et al., 2015 [39]**<br>**Portugal**	Ebola impact on African health systems entails a quest for more international and local resilience: the case of African Portuguese speaking countries	Meso/Macro	Global health	East Africa	Narrative review and case study	Demonstrated fragility of African health systems during Ebola epidemic: increased demand, inequalities and poor access, infrastructure impact, decreased in standards of care of other areas, themes for learning and improvement, critical services need to continue
**Li et al., 2020 [40]**<br>**China**	Analysing Healthcare Facility Resilience: Scientometric Review and Knowledge Map	Meso	Global health (UHC)	China	Literature review	Measures tend to be indicator based (set of dimensions and indicators), function based (quantitative evaluation of functionality of healthcare facilities), or structure based (modelling of system structures (elements and relationships) and testing failure).<br><br>Concepts: preparedness (risk assessment, strengthen capacities, HRH, HIS, leadership and governance, coordination), monitoring (surveillance, warning systems, monitoring systems), absorb (physical capacities, past experience, flexible approaches, HIS, communication, recovery and learning (safety1 & 2 approaches, organisational quality improvement strategies)

Tan MZY, *et al. BMJ Open* 2024; 13:e072136. doi: 10.1136/bmjopen-2023-072136

**Luke et al., 2021** [41] **Australia**	Safer hospital infrastructure assessments for socio-natural disaster - A scoping review	Meso	Disaster management	International	Scoping review	Uses WHO hospital safety index. Key domains: structural (Previous damage and repair, building integrity, condition of building materials, interaction with other buildings/elements, structural redundancies, architectural design, structure design appropriate to location), non-structural (architectural safety, infrastructure protection, access, and physical security, medical equipment and supplies), critical systems (electrical, telecommunications, water, fire protection, waste management, fuel supplies, medical gases, heating/ventilation)
**Meyer et al., 2020** [42] **USA**	A checklist to improve health system resilience to infectious disease outbreaks and natural hazards	Meso/Macro	Global health , Disaster	International	Scoping review + Interview + Checklist workshop	Proposes checklist as a measurement tool or set of indicators for resilience. Items: maintenance of essential health system services and functions, critical infrastructure and transportation, financing, access, communication, collaboration, coordination and partnerships, leadership and command, surge capacity, risk communications, workforce, infection control
**Nuzzo et al., 2019** [43] **USA**	What makes health systems resilient against infectious disease outbreaks and natural hazards? Results from a scoping review	Macro	Global health	International	Scoping review	Resilience attributes: maintenance of essential functions, access to healthcare, maintaining critical infrastructure and transport, crisis financing, leadership and command, collaboration, coordination and partnerships, communication, flexible plans and management structures, legal preparations, surge capacity, crisis standards of care, human resources for health, infection prevention and control, quality improvement, post-event recovery (psychological health of staff, revision of plans, rebuilding social cohesion and trust).<br><br>Themes are consistent with the Resilience Index. Additional findings: developing crisis standards of care, plan for post-event recovery, commitment to quality improvement to ensure lessons learned.
**Oppenheim et al., 2019** [44] **USA**	Assessing global preparedness for the next pandemic: development and application of an Epidemic Preparedness Index	Macro	Global health	International	Framework proposal and evaluation	Epidemic Preparedness Index: public health infrastructure (surveillance, immunisation, medical workforce, hospital capacity, coordination), physical infrastructure (water and sanitation, roads, phones, internet, logistics), institutional capacity (political stability, corruption, bureaucratic effectiveness, armed conflict, homicide, vital registration), economic resources (government revenue generation, per capita income, gross domestic product, health spending, resource dependency), public health communications (public education, risk communication)
**Penaloza et al., 2020** [45] **Brazil**	A resilience engineering perspective of safety performance measurement systems: A systematic literature review	Meso	Resilience engineering	International	Literature review	Safety performance measurement systems should support real-time monitoring and feedback of everyday performance variability, with the ability to analyse adverse events and excellent practices. Measures should be dynamic and subject to re-evaluation

Tan MZY, *et al. BMJ Open* 2024; 13:e072136. doi: 10.1136/bmjopen-2023-072136

**Pillay & Morel, 2020 [46] Australia**	Measuring Resilience Engineering: An Integrative Review and Framework for Bench-Marking Organisational Safety	Meso	Resilience engineering	International	Integrative review	"Resilience Engineering is a perspective for organisational safety management which enables organisational members to actively anticipate, respond, monitor and learn; by adapting to operate at the boundary of safe operations by narrowing the gap between work as imagined and work as performed; and manifested in an organisation's culture, cognition and behaviours"<br><br>Argues the need to establish boundaries of safe performance, balance trade-offs between production and safety, and dealing with managed vs prescribed safety. Shifts resilience culture towards resilient climate, acknowledging environmental conditions necessary for resilience.<br><br>Key indicators of resilience: learning culture, flexibility, top management commitment, awareness, preparedness, just culture
**Rameshshanker et al., 2021 [47] Canada**	Health system resilience to extreme weather events in Asia-Pacific: a scoping review	Macro/Meta	Disaster, climate change	Asia-Pacific	Literature review	Explored how well health systems were resilient towards adverse weather events. Categorised efforts according to WHO building blocks checklist.<br>Medical workforce was the most frequently mentioned aspect, while health financing was the least.
**Rangachari & Woods, 2020 [48] USA**	Preserving Organizational Resilience, Patient Safety, and Staff Retention during COVID-19 Requires a Holistic Consideration of the Psychological Safety of Healthcare Workers	Micro/Meso	Psychology, Operational research	International	Narrative review	During COVID19, trust and leadership were key aspects to maintain holistic resiliience of staff, which may improve organisational resilience. Advocates for environment of trst, empowerment and safety to enable patient safety concerns to be brought up
**Ravi et al., 2019 [49] USA**	Establishing a theoretical foundation for measuring global health security: A scoping review	Macro	Global health - HSS	International	Scoping review	Focused on health security, centred around performance of critical health security systems, biosafety and biosecurity, and public health preparedness. Underscored importance of real-time health information systems.<br><br>Key concepts: measuring global health security requires analysis of existing prevention, detection, and response capacities, as well as efforts to build core public health, healthcare, and biosecurity capacities. Assessments of national programs and efforts to mitigate a critical subset of priority threats could serve as useful proxies for measuring global health security. There are measurable enabling factors that facilitate global health security-strengthening efforts. Measuring global health security requires consideration of the risk environment from which infectious disease threats might emerge
**Rezaei et al., 2018 [50] Iran**	Hospitals preparedness using WHO guideline: A systematic review and meta-analysis	Meso	Emergency management	International	Systematic review and meta-analysis	Measured compliance to WHO hospital emergency checklist. Domains include command and control, communication, safety and security, triage, surge capacity, continuity of essential services, human resources for health, logistics and supply management, post-disaster recovery

**Son et al., 2020** [51] **USA**	Investigating resilience in emergency management: An integrative review of literature	Meso/Macro	Emergency management	International	Integrative review	Presents a table of collated definitions. Stressed the temporal nature in defining resilience. Some focus on pre-event whilst others focus on intra-event. Expected disruption: some focus on the ability to deal with unexpected events, whilst others do not view the distinction. Means to achieve resilience: allocation of roles and resources, HIS or adjusting procedures.<br><br>Dimensions of resilience in emergency management: collective sense-making (improvisation, honest communication, virtual role systems, attitude of wisdom), team decision making (incident command system, training in generic decision-making protocols), harmonising WAI and WAD, interaction and coordination.<br><br>Tools used to support human operators for resilience: geospatial mapping, event history logs, mobile communication systems, decision support tools
**Tessema et al., 2021** [52] **Australia**	The COVID-19 pandemic and healthcare systems in Africa: a scoping review of preparedness, impact and response	Macro	Global health, COVID	Africa	Scoping review	Used Population, Concept Context (PCC) framework for defining eligibility<br>Measured preparedness, impact and response of health systems<br>Framed within WHO building blocks.<br><br>Key findings: preparedness (limited resources, knowledge gaps, lack of training, limited surge capacity and testing ability, limited health services), Impact (reduced patient flow, miss appointments for chronic services, re-orientation of services deter essential services provision, not all services affected), impact on workforce (stigma, mental health illness), response (provision of guidelines, telehealth, segregation of patient streams)
**Tin et al., 2010** [53] **Myanmar**	An approach to health system strengthening in the Union of Myanmar	Macro	Public health - HSS	Myanmar	Case study/ national review	Prioritised service delivery, programme coordination and human resource management to strengthen Myanmar's health system. Takes a systems thinking approach. Uses Health Systems Strengthening indicators at measures and index to compare several countries.
**Tippong et al., 2021** [54] **UK**	A Review of Applications of Operational Research in Healthcare Coordination in Disaster Management	Meso/Macro	Emergency and Disaster management	International	Literature review	Measured coordination effectiveness in healthcare systems. Various staffing patterns have trade-offs: financial cost vs sustainable pattern. Triage and minimisation of beds needed. Coordination between healthcare-network is important for surge management and adaptive capacity.
**Turenne et al., 2019** [55] **France**	Conceptual analysis of health systems resilience: A scoping review	Macro	Theoretical	International	Scoping review	Presented a collated table of definitions and word frequency table. Concepts are dependent on contexts (different groups and populations)<br>In the face of changes. Argues that resilience thinking may be multi-sectoral, but health systems resilience is primarily concerned with the functions of the health system

Tan MZY, *et al. BMJ Open* 2024; 13:e072136. doi: 10.1136/bmjopen-2023-072136

**Turner et al., 2021 [56] Colombia**	Systematic review of experiences and perceptions of key actors and organisations at multiple levels within health systems internationally in responding to COVID-19	Micro/Meso/ Macro	Global health - COVID	International	Narrative review	Individual level: staff needed to adapt to new working conditions, burnout and psychological distress: work intensity, staff shortages, fear of contagion and transmission, positive adaptation: Telehealth, community healthcare workers, creative approaches<br><br>Organisational level: COVID as catalyst for service change, need to provide psychological support for staff, open management style during crisis<br><br>Local health system level: continuous training to support preparedness for future pandemics, the need to look at working conditions, the need to coordinate diverse actors (community and primary health etc), review and update regulations
**Wiig et al., 2020 [57] Norway**	Defining the boundaries and operational concepts of resilience in the resilience in healthcare research programme	Meso	Patient safety	International	Debate and review	Centred around engineering, psychological and ecological perspectives: Resilience of what, to what, through what. Focused around high quality healthcare, centred around set a capacities, based on events which provide challenges, changes or disruptions. Grounded in multi-level adaptation, enhancement and reorganisation, indicating diversity, coordination and collaboration and is open-ended
**Xin & Xu, 2012 [58] China**	Hospital emergency management research in China: trends and challenges	Macro	Emergency management	China	Narrative review	Defines crisis based on disruptions to hospital systems: patient surge, provision of services, reputation, financial trouble, or major incident. Noted a move from normal vs abnormal crisis to all-hazards. Comprehensive management system may be more useful than temporary command and control, or professional response team. Identified current issues with Chinese medical systems based on WHO building blocks
**Zhong et al., 2014 [59] China**	Development of hospital disaster resilience: conceptual framework and potential measurement	Macro	Global health - Disaster	International	Literature review	Presented a collated list of definitions from different disciplines. Most measures focus on structure, but hardly on process and outcome (based on Donebedian categories of quality of care). Propose measures as a framework: Hospital safety and vulnerability - surveillance, risk & safety. Disaster preparedness and resources - Leadership, cooperation and communication, logistics & stockpiles, workforce, training & drills. Continuity of essential services - surge capacity, emergency medicines. Recovery and adaptation - evaluation, adaptation and recovery capability.<br><br>Factors conferring resilient healthcare: Accessibility, Critical services, Leadership, Integration with community

Legend: WHO = World Health Organisation. DRR = Disaster Risk Reduction. USA = United States of America. HRH = Human Resources for Health. L-LMIC = Low and Low-Middle Income Countries. PTSD = Post-Traumatic Stress Disorder. SFDRR = Sendai Framework for Disaster Risk Reduction. HIS = Health Information Systems

Tan MZY, *et al. BMJ Open* 2024; 13:e072136. doi: 10.1136/bmjopen-2023-072136

Appendix 4: Summary characteristics of selected resilience frameworks

Framework (reference)	Key components or indicators or principles	Hierarchical focus	Paradigm	Pre-event	Intra-event	Post-event
**Resilience Potentials** [60]	Anticipate,<br>Monitor,<br>Respond,<br>Learn	Meso	Engineering			
**Independent EM concepts** [51]	Collective sense-making,<br>Team decision-making,<br>Harmonising WAI/WAD,<br>Interaction and Coordination	Meso	Emergency Management			
**FEMA adapted emergency management cycle** [26]	Mitigation, hazard and capacity assessment<br>Planning<br>Information systems<br>Continuity of services<br>Emergency response<br>Communications<br>Recovery	Meso	Emergency Management			
**WHO Hospital Safety Index (HSI)** [41]	Structural (e.g. building integrity, redundancies, previous damage)<br>Non-structural (e.g. critical systems, medical equipment and supplies)<br>Disaster management system	Meso	Healthcare			
**WHO hospital disaster preparedness** [46 50]	Command and Control<br>Communication<br>Safety and Security<br>Triage<br>Surge capacity<br>Continuity of essential services<br>HRH<br>Logistics and supply management<br>Post-disaster recovery	Meso	Healthcare			
**Hospital disaster resilience model** [28]	3 domains with 27 sub-domains:<br>Constructive (stability, design, architectural, transportation)<br>Infrastructural (power, water/sewage, communication/IT, HVAC, fuel, medical gas, equipment<br>Administrative (disaster plan, command, coordination, needs assessment, logistics and supplies, safety committee, continuity of services, volunteers, finance, recovery, training)	Meso	Healthcare			
**Independent hospital and healthcare resilience** [59]	4 principles:<br>Robustness<br>Redundancy<br>Rapidity<br>Resourcefulness<br>4 cross cutting domains:<br>Vulnerability and safety<br>Recovery and adaptation<br>Continuity of essential services<br>Preparedness and resources	Meso/Macro	Healthcare			
**Independent disaster preparedness cycle** [25]	5 parts of cycle further divided into components, all underpinned by leadership & coordination:<br>Plan<br>Organise & equip<br>Train<br>Exercise	Meso/Macro	Disaster Management			

	Evaluate & improve					
**Concepts conferring resilience [17]**	Material resources<br>Preparedness & planning<br>Information management<br>Collateral pathways and redundancies<br>Governance processes<br>Leadership<br>Organisational culture<br>Human capital<br>Social networks and collaboration	Meso/Macro	Healthcare			
**Primary Care Framework for pandemics (from H1N1) [33]**	Clinical service delivery,<br>Public health functions,<br>Primary care facility operational level<br>Health system level	Meso/Macro	Healthcare			
**NHS Emergency Preparedness, Resilience and Response (EPRR) framework**	Preparedness & anticipation<br>Continuity<br>Subsidiary: decisions at lowest level<br>Communication<br>Cooperation & integration<br>Direction	Macro	Healthcare			
**SFDRR [61]**	Reduce Disaster Mortality<br>Reduce the Number of Affected People<br>Reduce Direct Disaster Economic Loss<br>Reduce Disaster Damage to Critical Infrastructure and Disruption of Basic Services<br>Increase the Number of Countries with National and Sub-National/Local Disaster Risk Reduction Strategies<br>Enhance International Cooperation<br>Increase the Availability of and Access to Multi-Hazard Early Warning Systems	Macro	Disaster Management			
**Global Health Security Index [62]**	Prevent (pathogens)<br>Detect<br>Respond<br>Healthcare (formal)<br>Norms<br>Risk	Macro	Public health			
**Epidemic Preparedness Index [44]**	Public health infrastructure: surveillance, immunisation, medical workforce, hospital capacity, coordination<br>Physical infrastructure: water and sanitation, roads, phones, internet, logistics<br>Institutional capacity: political stability, corruption, bureaucratic effectiveness, armed conflict, homicide, vital registration<br>Economic resources: government revenue generation, per capita income, gross domestic product, health spending, resource dependency<br>Public health communications: public education, risk communication	Macro	Public health			
**European Primary Care Monitoring [63]**	Structure: Governance, economic conditions, workforce<br>Process: access, comprehensiveness, coordination and continuity<br>Outcome: Quality, efficiency, equity	Macro	Healthcare			

**USA Public Health Infrastructure** [64]	Assessment: monitor health, diagnose/investigate<br>Policy development: Inform/educate/empower, community partnerships, develop policies<br>Assurance: Enforce laws, link care, competent workforce, evaluate	Macro	Public health			
**WHO building blocks** [32]	Leadership & governance,<br>Health Information Systems (HIS),<br>Human Resources for Health (HRH),<br>Financing,<br>Service delivery,<br>Essential medicines and technologies	Macro	Healthcare			
**National Health Security Preparedness Index (NHSPI)** [21]	Health surveillance: surveillance and epidemiology investigation, environmental and biological monitoring, laboratory testing<br>Community planning and engagement: cross-sector collaboration, at-risk populations, spontaneous volunteers, social capital and cohesions<br>Incident and information management: multi-agency coordination, emergency public information and warning<br>Surge management: emergency medical services, acute & primary care, mental healthcare<br>Countermeasure management: medical supply chain and distribution, countermeasure utilisation and effectiveness	Macro	Healthcare			
**Integrated Health Service Delivery** [34]	Clinical,<br>Service,<br>Organisational,<br>Functional	Macro	Healthcare			
**Emerging Infectious Diseases Preparedness** [65]	Hardware: Surveillance, infrastructure & medical supplies, workforce, communication mechanisms<br>Software: Governance, trust	Macro	Healthcare			
**Myanmar Health Systems Strengthening Strategy** [53]	Service delivery<br>Programme coordination<br>Human resources	Macro	Healthcare			
**Stockholm Resilience Centre 7 Principles** [66]	Foster complex adaptive systems thinking,<br>Maintain diversity & redundancies,<br>Broaden Participation,<br>Manage connectivity,<br>Manage slow variables and feedback,<br>Encourage learning,<br>Polycentric governance	Macro/Meta	Socioecological systems			
**Refined conceptual models of health system resilience** [30 43]	Foundations of resilience added onto the Resilience Index:<br>Realigned relationships,<br>Foresight and motivation,<br>Change management,<br>Emergency preparedness<br>Crisis standards of care<br>Recovery plans<br>Learning and improvement	Micro/Meso/Macro	Healthcare			
**Health Systems Resilience Index** [31]	Aware: capacity, risks and communication<br>Diverse: meets range of needs, adequate financing<br>Self-regulating: maintain core services and leverage collaborations<br>Integrated: coordination and community engagement<br>Adaptive: distributive decision-making, flexible spending and evaluation for improvement	Meso/Macro/Meta	Healthcare			

Legend: Frameworks are categorised according to predominant hierarchical

focus and paradigm. They are colour-coded according to which time domain they cover.

Abbreviations: EM = Emergency Management. WAI/WAD = Work-As-Imagined vs Work-As-Done. FEMA = Federal Emergency Management Agency. WHO = World Health Organisation. HRH = Human Resources for Health. IT = Information Technology. HVAC = Heating, Ventilation and Air-Conditioning. NHS = UK's National Health Service. SFDRR = Sendai Framework for Disaster Risk Reduction. USA = United States of America.

Tan MZY, *et al. BMJ Open* 2024; 13:e072136. doi: 10.1136/bmjopen-2023-072136

References

1. Xiao Y, Watson M. Guidance on Conducting a Systematic Literature Review. *Journal of Planning Education and Research* 2019;39(1):93-112. doi: 10.1177/0739456x17723971
2. Greenhalgh T, Robert G, Macfarlane F, et al. Storylines of research in diffusion of innovation: a meta-narrative approach to systematic review. *Soc Sci Med* 2005;61(2):417-30. doi: 10.1016/j.socscimed.2004.12.001 [published Online First: 2005/05/17]
3. Gough D. Meta-narrative and realist reviews: guidance, rules, publication standards and quality appraisal. *BMC Medicine* 2013;11(1):22. doi: 10.1186/1741-7015-11-22
4. Smith V, Devane D, Begley CM, et al. Methodology in conducting a systematic review of systematic reviews of healthcare interventions. *BMC Med Res Methodol* 2011;11(1):15. doi: 10.1186/1471-2288-11-15 [published Online First: 20110203]
5. Becker L, Oxman A. Overviews of reviews. In: Higgins J, Green S, eds. Cochrane handbook for systematic reviews of interventions. Oxford: The Cochrane Collaboration 2010.
6. Wong G, Greenhalgh T, Westhorp G, et al. RAMESES publication standards: meta-narrative reviews. *BMC Medicine* 2013;11(1):20. doi: 10.1186/1741-7015-11-20
7. Higgins JP TJ, Chandler J, Cumpston M, Li T, Page MJ, Welch VA, editor(s). Cochrane Handbook for Systematic Reviews of Interventions Version 6.2 (updated February 2021): Cochrane; 2021 [Available from: training.cochrane.org/handbook accessed Feb 2022.
8. Hannes K. Supplementary Guidance for Inclusion of Qualitative Research in Cochrane Systematic Reviews of Interventions. Version 1 (updated August 2011). In: Noyes J BA, Hannes K, Harden A, Harris J, Lewin S, Lockwood C ed.: Cochrane Collaboration Qualitative Methods Group 2011.
9. HONG QN, PLUYE P, FÀBREGUES S, et al. MIXED METHODS APPRAISAL TOOL (MMAT): McGill University; 2018 [Available from: http://mixedmethodsappraisaltoolpublic.pbworks.com/w/file/fetch/127916259/MMAT_2018_criteria-manual_2018-08-01_ENG.pdf accessed Jan 2022.
10. CASP. Critical Appraisal Skills Programme - Qualitative Checklist 2018 [Available from: https://casp-uk.net/wp-content/uploads/2018/01/CASP-Qualitative-Checklist-2018.pdf accessed

Feb 2022.

11. Harrison R, Jones B, Gardner P, et al. Quality assessment with diverse studies (QuADS): an appraisal tool for methodological and reporting quality in systematic reviews of mixed- or multi-method studies. *BMC Health Services Research* 2021;21(1):144. doi: 10.1186/s12913-021-06122-y
12. Shea BJ, Reeves BC, Wells G, et al. AMSTAR 2: a critical appraisal tool for systematic reviews that include randomised or non-randomised studies of healthcare interventions, or both. *BMJ* 2017;358:j4008. doi: 10.1136/bmj.j4008
13. Alami H, Lehoux P, Fleet R, et al. How Can Health Systems Better Prepare for the Next Pandemic? Lessons Learned From the Management of COVID-19 in Quebec (Canada). *Frontiers in public health* 2021;9:671833. doi: 10.3389/fpubh.2021.671833
14. Augustynowicz A, Opolski J, Waszkiewicz M. Resilient Health and the Healthcare System. A Few Introductory Remarks in Times of the COVID-19 Pandemic. *Int J Environ Res Public Health* 2022;19(6) doi: 10.3390/ijerph19063603
15. Ayanore MA, Amuna N, Aviisah M, et al. Towards Resilient Health Systems in Sub-Saharan Africa: A Systematic Review of the English Language Literature on Health Workforce, Surveillance, and Health Governance Issues for Health Systems Strengthening. *Annals of Global Health* 2019;85(1) doi: 10.5334/aogh.2514
16. Banwell N, Rutherford S, Mackey B, et al. Towards improved linkage of disaster risk reduction and climate change adaptation in health: A review. *Int J Environ Res Public Health* 2018;15(4) doi: 10.3390/ijerph15040793
17. Barasa E, Mbau R, Gilson L. What is resilience and how can it be nurtured? A systematic review of empirical literature on organizational resilience. *International Journal of Health Policy and Management* 2018;7(6):491-503. doi: 10.15171/ijhpm.2018.06
18. Berg SH, Akerjordet K, Ekstedt M, et al. Methodological strategies in resilient health care studies: An integrative review. *Safety Science* 2018;110:300-12. doi: 10.1016/j.ssci.2018.08.025
19. Biddle L, Wahedi K, Bozorgmehr K. Health system resilience: A literature review of empirical research. *Health Policy and Planning* 2020;35(8):1084-109. doi: 10.1093/heapol/czaa032
20. Blanchet K, Nam SL, Ramalingam B, et al. Governance and Capacity to Manage Resilience of Health Systems: Towards a New Conceptual Framework. *International Journal of Health Policy and Management*

2017;6(8):431-35. doi: 10.15171/ijhpm.2017.36
21. Blumenstock J, Bakker G, Jarris PE. Measuring Preparedness: The National Health Security Preparedness Index. *Journal of Public Health Management and Practice* 2014;20(3):361-63. doi: 10.1097/phh.0000000000000073
22. Brand FS, Jax K. Focusing the Meaning(s) of Resilience: Resilience as a Descriptive Concept and a Boundary Object. *Ecology and Society* 2007;12(1)
23. Burke S, Parker S, Fleming P, et al. Building health system resilience through policy development in response to COVID-19 in Ireland: From shock to reform. *Lancet Reg Health Eur* 2021;9:100223. doi: 10.1016/j.lanepe.2021.100223 [published Online First: 20211007]
24. Carrington MA, Ranse J, Hammad K. The impact of disasters on emergency department resources: review against the Sendai framework for disaster risk reduction 2015–2030. *Australas Emerg Care* 2021;24(1):55-60. doi: 10.1016/j.auec.2020.09.003
25. Cartwright C, Hall M, Lee ACK. The changing health priorities of earthquake response and implications for preparedness: a scoping review. *Public Health* 2017;150:60-70. doi: 10.1016/j.puhe.2017.04.024
26. Challen K, Lee AC, Booth A, et al. Where is the evidence for emergency planning: A scoping review. *BMC Public Health* 2012;12(1) doi: 10.1186/1471-2458-12-542
27. Curtis S, Fair A, Wistow J, et al. Impact of extreme weather events and climate change for health and social care systems. *Environmental Health: A Global Access Science Source* 2017;16 doi: 10.1186/s12940-017-0324-3
28. Fallah-Aliabadi S, Ostadtaghizadeh A, Ardalan A, et al. Towards developing a model for the evaluation of hospital disaster resilience: A systematic review. *BMC Health Services Research* 2020;20(1) doi: 10.1186/s12913-020-4915-2
29. Fridell M, Edwin S, Schreeb JV, et al. Health system resilience: what are we talking about? A scoping review mapping characteristics and keywords. *International Journal of Health Policy and Management* 2020;9(1):6-16. doi: 10.15171/ijhpm.2019.71
30. Grimm PY, Oliver S, Merten S, et al. Enhancing the Understanding of Resilience in Health Systems of Low- and Middle-Income Countries: A Qualitative Evidence Synthesis. *International Journal of Health Policy and Management* 2021 doi: 10.34172/ijhpm.2020.261
31. Kruk ME, Ling EJ, Bitton A, et al. Building resilient health systems: a proposal for a resilience index. *BMJ* 2017;357:j2323. doi:

10.1136/bmj.j2323
32. WHO. Key components of a well functioning health system 2010 [Available from: https://www.who.int/healthsystems/EN_HSSkeycomponents.pdf accessed Nov 2019.
33. Haldane V, Zhang Z, Abbas RF, et al. National primary care responses to COVID-19: a rapid review of the literature. *BMJ Open* 2020;10(12):e041622. doi: 10.1136/bmjopen-2020-041622 [published Online First: 20201208]
34. Hasan MZ, Neill R, Das P, et al. Integrated health service delivery during COVID-19: A scoping review of published evidence from low-income and lower-middle-income countries. *BMJ Global Health* 2021;6(6) doi: 10.1136/bmjgh-2021-005667
35. Hosseini S, Barker K, Ramirez-Marquez JE. A review of definitions and measures of system resilience. *Reliability Engineering & System Safety* 2016;145:47-61. doi: https://doi.org/10.1016/j.ress.2015.08.006
36. Iflaifel M, Lim RH, Ryan K, et al. Resilient Health Care: a systematic review of conceptualisations, study methods and factors that develop resilience. *BMC Health Serv Res* 2020;20(1):324. doi: 10.1186/s12913-020-05208-3 [published Online First: 2020/04/19]
37. Jesus TS, Kamalakannan S, Bhattacharjya S, et al. PREparedness, REsponse and SySTemic transformation (PRE-RE-SyST): a model for disability-inclusive pandemic responses and systemic disparities reduction derived from a scoping review and thematic analysis. *Int J Equity Health* 2021;20(1):204. doi: 10.1186/s12939-021-01526-y [published Online First: 20210914]
38. Koeva S, Rohova M. Health system resilience: concept development. *Journal of Imab* 2020;26(3):3251-58. doi: 10.5272/jimab.2020263.3251
39. Lapão LV, Silva A, Pereira N, et al. Ebola impact on African health systems entails a quest for more international and local resilience: the case of African Portuguese speaking countries. *Pan Afr Med J* 2015;22 Suppl 1(Suppl 1):15. doi: 10.11694/pamj.supp.2015.22.1.6653 [published Online First: 20151011]
40. Li X, Krumholz HM, Yip W, et al. Quality of primary health care in China: challenges and recommendations. *The Lancet* 2020;395(10239):1802-12. doi: 10.1016/S0140-6736(20)30122-7
41. Luke J, Franklin R, Aitken P, et al. Safer hospital infrastructure assessments for socio-natural disaster - A scoping review. *Prehosp Disaster Med* 2021;36(5):627-35. doi: 10.1017/S1049023X21000650
42. Meyer D, Bishai D, Ravi SJ, et al. A checklist to improve health system resilience to infectious disease outbreaks and natural hazards. *BMJ Glob*

Health 2020;5(8) doi: 10.1136/bmjgh-2020-002429
43. Nuzzo JB, Meyer D, Snyder M, et al. What makes health systems resilient against infectious disease outbreaks and natural hazards? Results from a scoping review. *BMC Public Health* 2019;19(1) doi: 10.1186/s12889-019-7707-z
44. Oppenheim B, Gallivan M, Madhav NK, et al. Assessing global preparedness for the next pandemic: development and application of an Epidemic Preparedness Index. *BMJ Global Health* 2019;4(1):e001157. doi: 10.1136/bmjgh-2018-001157
45. Penaloza GA, Saurin TA, Formoso CT, et al. A resilience engineering perspective of safety performance measurement systems: A systematic literature review. *Safety Science* 2020;130 doi: 10.1016/j.ssci.2020.104864
46. Pillay M, Morel G. Measuring Resilience Engineering: An Integrative Review and Framework for Bench-Marking Organisational Safety. *Safety* 2020;6(3) doi: 10.3390/safety6030037
47. Rameshshanker V, Wyngaarden S, Lau LL, et al. Health system resilience to extreme weather events in Asia-Pacific: a scoping review. *Clim Dev* 2021;13(10):944-58. doi: 10.1080/17565529.2020.1870425
48. Rangachari P, J LW. Preserving Organizational Resilience, Patient Safety, and Staff Retention during COVID-19 Requires a Holistic Consideration of the Psychological Safety of Healthcare Workers. *Int J Environ Res Public Health* 2020;17(12) doi: 10.3390/ijerph17124267 [published Online First: 2020/06/19]
49. Ravi SJ, Meyer D, Cameron E, et al. Establishing a theoretical foundation for measuring global health security: A scoping review. *BMC Public Health* 2019;19(1) doi: 10.1186/s12889-019-7216-0
50. Rezaei F, Maracy MR, Yarmohammadian MH, et al. Hospitals preparedness using WHO guideline: A systematic review and meta-analysis. *Hong Kong Journal of Emergency Medicine* 2018;25(4):211-22. doi: 10.1177/1024907918760123
51. Son C, Sasangohar F, Neville T, et al. Investigating resilience in emergency management: An integrative review of literature. *Appl Ergon* 2020;87 doi: 10.1016/j.apergo.2020.103114
52. Tessema GA, Kinfu Y, Dachew BA, et al. The COVID-19 pandemic and healthcare systems in Africa: a scoping review of preparedness, impact and response. *Bmj Global Health* 2021;6(12) doi: 10.1136/bmjgh-2021-007179
53. Tin N, Lwin S, Kyaing NN, et al. An approach to health system strengthening in the Union of Myanmar. *Health Policy* 2010;95(2-3):95-

102. doi: 10.1016/j.healthpol.2009.11.013

54. Tippong D, Petrovic S, Akbari V. A Review of Applications of Operational Research in Healthcare Coordination in Disaster Management. *Eur J Oper Res* 2021 doi: 10.1016/j.ejor.2021.10.048 [published Online First: 20211028]
55. Turenne CP, Gautier L, Degroote S, et al. Conceptual analysis of health systems resilience: A scoping review. *Soc Sci Med* 2019;232:168-80. doi: 10.1016/j.socscimed.2019.04.020 [published Online First: 20190430]
56. Turner S, Botero-Tovar N, Herrera MA, et al. Systematic review of experiences and perceptions of key actors and organisations at multiple levels within health systems internationally in responding to COVID-19. *Implementation Science* 2021;16(1) doi: 10.1186/s13012-021-01114-2
57. Wiig S, Aase K, Billett S, et al. Defining the boundaries and operational concepts of resilience in the resilience in healthcare research program. *BMC Health Services Research* 2020;20(1) doi: 10.1186/s12913-020-05224-3
58. Xin YT, Xu KY. Hospital emergency management research in China: trends and challenges. *Emerg Med J* 2012;29(5):353-7. doi: 10.1136/emermed-2011-200512 [published Online First: 20111103]
59. Zhong S, Clark M, Hou X-Y, et al. Development of hospital disaster resilience: conceptual framework and potential measurement. *Emergency Medicine Journal* 2014;31(11):930-U8901. doi: 10.1136/emermed-2012-202282
60. Hollnagel E. Epilogue: RAG–the resilience analysis grid. Resilience engineering in practice: CRC Press 2017:275-96.
61. Calkins J. Moving forward after Sendai: How countries want to use science, evidence and technology for disaster risk reduction. *PLoS Currents* 2015;7(DISASTERS) doi: 10.1371/currents.dis.22247d6293d4109d09794890bcda1878
62. Bell JA, Nuzzo JB. Global Health Security Index: Advancing Collective Action and Accountability Amid Global Crisis: NTI/John Hopkins center for health security; 2021 [Available from: www.GHSIndex.org accessed Mar 2022.
63. Kringos DS, Boerma WGW, Bourgueil Y, et al. The european primary care monitor: structure, process and outcome indicators. *BMC Fam Pract* 2010;11(1):81. doi: 10.1186/1471-2296-11-81
64. Benjamin GC. Ensuring health equity during the COVID-19 pandemic: The role of public health infrastructure. *Revista Panamericana de Salud Publica/Pan American Journal of Public Health* 2020;44 doi: 10.26633/RPSP.2020.70

65. Palagyi A, Marais BJ, Abimbola S, et al. Health system preparedness for emerging infectious diseases: A synthesis of the literature. *Glob Public Health* 2019;14(12):1847-68. doi: 10.1080/17441692.2019.1614645 [published Online First: 20190514]
66. Biggs R, Schlüter M, Schoon ML. Principles for Building Resilience: Cambridge University Press 2015.
