# Supplementary material for: Healthcare resilience: a meta-narrative systematic review and synthesis of reviews
Source: BMJ Open. 2023 Sep 20;13(9):e072136. doi: 10.1136/bmjopen-2023-072136 (PMC10514640; doi:10.1136/bmjopen-2023-072136)
Supplement: Author's manuscript [file bmjopen-2023-072136.draft_revisions.pdf]

BMJ Open

BMJ Open is committed to open peer review. As part of this commitment we make the peer review history of every article we publish publicly available.

When an article is published we post the peer reviewers' comments and the authors' responses online. We also post the versions of the paper that were used during peer review. These are the versions that the peer review comments apply to.

The versions of the paper that follow are the versions that were submitted during the peer review process. They are not the versions of record or the final published versions. They should not be cited or distributed as the published version of this manuscript.

BMJ Open is an open access journal and the full, final, typeset and author-corrected version of record of the manuscript is available on our site with no access controls, subscription charges or pay-per-view fees (http://bmjopen.bmj.com).

If you have any questions on BMJ Open's open peer review process please email info.bmjopen@bmj.com

BMJ Open

Healthcare Resilience: A meta-narrative systematic review and synthesis of reviews

Journal:	*BMJ Open*
Manuscript ID	bmjopen-2023-072136
Article Type:	Original research
Date Submitted by the Author:	24-Jan-2023

Complete List of Authors:	Tan, Mark; The University of Manchester,<br>Prager, Gabrielle; Johns Hopkins University<br>McClelland, Andrew; The University of Manchester, Alliance Manchester Business School<br>Dark, Paul; National Institute for Health and Care Research, Clinical Research Network; The University of Manchester, Humanitarian and Conflict Response Institute

Keywords:	Systematic Review, COVID-19, Health policy < HEALTH SERVICES ADMINISTRATION & MANAGEMENT, Organisation of health services < HEALTH SERVICES ADMINISTRATION & MANAGEMENT, Health Services Accessibility

SCHOLARONE™
Manuscripts

I, the Submitting Author has the right to grant and does grant on behalf of all authors of the Work (as defined in the below author licence), an exclusive licence and/or a non-exclusive licence for contributions from authors who are: i) UK Crown employees; ii) where BMJ has agreed a CC-BY licence shall apply, and/or iii) in accordance with the terms applicable for US Federal Government officers or employees acting as part of their official duties; on a worldwide, perpetual, irrevocable, royalty-free basis to BMJ Publishing Group Ltd ("BMJ") its licensees and where the relevant Journal is co-owned by BMJ to the co-owners of the Journal, to publish the Work in this journal and any other BMJ products and to exploit all rights, as set out in our licence.

The Submitting Author accepts and understands that any supply made under these terms is made by BMJ to the Submitting Author unless you are acting as an employee on behalf of your employer or a postgraduate student of an affiliated institution which is paying any applicable article publishing charge ("APC") for Open Access articles. Where the Submitting Author wishes to make the Work available on an Open Access basis (and intends to pay the relevant APC), the terms of reuse of such Open Access shall be governed by a Creative Commons licence – details of these licences and which Creative Commons licence will apply to this Work are set out in our licence referred to above.

Other than as permitted in any relevant BMJ Author's Self Archiving Policies, I confirm this Work has not been accepted for publication elsewhere, is not being considered for publication elsewhere and does not duplicate material already published. I confirm all authors consent to publication of this Work and authorise the granting of this licence.

For peer review only

Healthcare resilience - A meta-narrative systematic review and synthesis of reviews

For peer review only

Abstract

Objectives

The COVID19 pandemic has tested global healthcare resilience. Many countries previously considered "resilient" have performed poorly. Available organisational and system frameworks tend to be context-dependent and focus heavily on physical capacities. This study aims to explore and synthesise evidence about healthcare resilience and present a unified framework for future resilience-building.

Design

Systematic review and synthesis of reviews using a meta-narrative approach.

Setting

Healthcare organisations and systems

Participants

There were no participants in this review of reviews.

Primary and secondary outcome measures

Definitions, concepts, and measures of healthcare resilience. We utilised thematic analysis across included reviews to summarise evidence on healthcare resilience.

Results

The main paradigms within healthcare resilience include global health, disaster risk reduction, emergency management, patient safety and public health. Definitions of healthcare resilience recognise various hierarchical levels: individual (micro), facility or organisation (meso), health system (macro), and planetary or international (meta). There has been a shift from a focus on mainly disasters and crises, to an "all-hazards" approach to resilience. Attempts to measure resilience have met with limited success. We analysed key concepts to build a framework for healthcare resilience containing pre-, intra-, post- and trans-event domains. Alongside, we synthesise a definition which dovetails with our framework.

Conclusion

Resilience increasingly takes an all-hazards approach and a process-oriented perspective. There is increasing recognition of the relational aspects of resilience. Few frameworks incorporate these, and they are difficult to capture within measurement systems. We need to understand how resilience works across hierarchical levels, and how competing priorities may affect overall resilience. Understanding these will underpin interdisciplinary, cross-sectoral and multi-level approaches to healthcare resilience for the future.

Summary statement

What is already known on this topic

- Resilience has been studied within many research traditions and paradigms. Definitions, and concepts continue to be refined.
- Most studies have focused on specific paradigms at single hierarchical levels. There has been recognition of the need for multi-level and interdisciplinary work.
- The COVID19 pandemic has tested the resilience of healthcare systems around the world and has challenged previous understanding of healthcare resilience.

What this study adds

- This is the first systematic review of reviews done on healthcare resilience.
- It consolidates knowledge across disciplines and hierarchical levels via a meta-narrative approach. We highlight international lessons from the COVID19 pandemic.
- We synthesise an evidence-based definition and conceptual framework which dovetail with each other, increasing convergence within this complex topic.

How this study might affect research, practice, or policy

- There is a need to better understand how inter-organisational relationships affect resilience.
- Governments must also consider international (meta-level) aspects of healthcare resilience (e.g. climate crisis and globalisation)
- Interdisciplinary, cross-sectoral and multi-level approaches must be adopted to build healthcare resilience in the future.

For peer review only

For peer review only

Main Manuscript Title

Healthcare resilience - A meta-narrative systematic review and synthesis of reviews

1. Background

The COVID19 pandemic has tested the resilience of healthcare across the globe. Previously thriving healthcare systems have struggled with multiple aspects; from hospital capacity [1] to infection control measures [2], healthcare worker burnout [3] to degrees of community trust [4], and from international research cooperation [5] to vaccine tribalism and hesitancy [6]. Our previous understanding of healthcare resilience requires re-evaluation.

To practically respond to the whole-of-society challenges in light COVID19, we need evidence that effectively integrates knowledge across these disciplines. This need has been highlighted by several reviews [7 8]. The meta-narrative approach offers such a method. It has been used to track several disciplines converging within a complex field, and is useful for making sense of such complex, heterogenous and disparate data across disciplines and sectors [9 10]. It thus forms an interdisciplinary and cross-sectoral approach to healthcare resilience.

Recognition that resilience at individual, organisational and system levels affect each other has resulted in some early multi-level empirical research protocols [11 12]. Yet, many studies tend to focus on single hierarchical levels[13-15], within set paradigms [16 17]. For example, literature on psychological resilience tends to focus on acute interventions during or after crisis [18]. These seldom consider wider determinants at organisational or system level which affect individual resilience [3]. Similarly, some disaster risk reduction (DRR) frameworks have put little emphasis on recovery after crisis [19], which has been highlighted as an important factor to build resilience after COVID19 [20]. Therefore, in addition to interdisciplinary and cross-sectoral evidence, multi-level knowledge must also be consolidated.

Given the sheer volume of empirical literature available on healthcare resilience, it was impractical to review beyond a single hierarchical level. Instead, reviews of reviews are particularly useful for gaining a broad overview of complex topics and systems with many dependencies. For example, it has helped to better understand policy and practice priorities in climate sciences [21], and to unpick COVID19 vaccine hesitancy [22]. Both are highly complex and current topics involving multiple sectors, disciplines, and hierarchical levels.

We therefore undertook a systematic review of reviews using a meta-narrative approach to present an overview and critical look at healthcare resilience. It highlights lessons learnt from the COVID19 pandemic and synthesises evidence towards an

For peer review only

overarching framework. The research aims to:

1. Explore the definitions, measures, and concepts of healthcare resilience.
2. Synthesise evidence from the exploration and analysis towards a broad overview of the topic
3. Present an interdisciplinary, cross-sectoral, and multi-level framework from analysed data

2. Methods

Theoretical approach

We undertook a systematic literature review of reviews using a meta-narrative approach [23 24]. The review was registered with PROSPERO (CRD42022314729)[25]. We utilised RAMESES guidelines for reporting meta-narrative reviews [24]. PRISMA guidelines were followed[27].

The principles of the meta-narrative review are pragmatism, pluralism, historicity, contestation, reflexivity, and peer-review. This approach was adopted for several reasons. Firstly, the field has been informed by many research traditions. Secondly, there exists historical agendas which have shifted the focus of resilience at various hierarchical levels (historicity). Thirdly, there are sometimes contrasting findings about resilience (pluralism and contestation). Fourthly, an interdisciplinary overview may be useful for policymakers, practitioners, and academics (pragmatism). Such an approach helps to extract seemingly heterogenous data (peer review). Finally, the final search strategy was informed by some initial searches and snowball sampling, reflecting a reflexive protocol which was adapted according to findings.

Search strategy

Keywords are presented in Figure 1. We included records from 2008 to November 2022. The search was performed in January 2022. A repeat search was performed in November 2022, which yielded five further records, four of which did not meet selection criteria. 2008 corresponded to the global financial crisis, which resulted in an increase in resilience literature [28]. Resilience is dynamic [29]. Concepts from earlier events may not possess the same relevance as more recent articles. This is especially the case since COVID19. There were reviews focused on other specific diseases and epidemics, but they mainly related to infection control measures [30]. There was a marked focus on individual psychological resilience, focusing on acute interventions to mental health crises. While we recognised the importance of individuals that make up organisations and systems, we wanted to focus on how resilience is understood at other hierarchical levels, so we excluded these reviews.

For peer review only - http://bmjopen.bmj.com/site/about/guidelines.xhtml

Study selection

Records were managed with Endnote 20 (Clarivate analytics). Two reviewers independently selected papers. This was done for several reasons. Firstly, there was wide heterogeneity of studies and papers. Secondly, some reviews refer to cross-sectoral interactions but didn't deal with healthcare. Thirdly, the quality of reviews varied significantly. As a result, having independent reviewers (peer-review) minimises personal bias and ensures there is agreement about selection criteria [26 31]. Screening was done using a modified two-stage process. Title screening was insufficient as a stage on its own due to lack of information about the actual review, or only using "resilience" as a tangential keyword. Both reviewers thus performed a title/abstract screening independently, followed by abstract/full-text screening and discussion around inclusion and exclusion criteria. Additional reviewers were agreed *a priori* should disagreements between the two primary reviewers occur. The final list of included papers was supplemented by snowball sampling and hand searching. This was done by searching through relevant references from selected reviews and discussions with other interdisciplinary team members. Because of the volume of results (Figure 2), it was impractical to focus on empirical studies, or we would have had to confine the search to a single hierarchical level. Therefore, we included reviews only. This may reduce details and resolution, but it allows a summary of knowledge across several reviews and disciplines [32].

Quality appraisal

Quality appraisal of articles was conducted using the Joanna Briggs Institute (JBI) checklist for systematic reviews [33]. While there are no specific quality appraisal tools for systematic reviews of reviews, the JBI checklist formed a best fit compared to several other tools (Appendix 1).

Data extraction and analysis

Using an inductive approach, three-tiered coding of the text was conducted around the three foci of definition, measures, and concepts [34](Appendix 1). We used Atlas.ti qualitative software and data extraction tables. We examined prominent definitions and how they have been informed by different research disciplines. We then collated definitions from the reviews and analysed the most frequently occurring words (Appendix 2). These results were categorised and synthesised with the concepts of resilience to produce a definition which acknowledges past research, links current knowledge, and spans hierarchical levels. Concepts were coded from reviews as well as from the frameworks presented in papers (Appendix 3).

3. Results

Four databases yielded 8845 records, of which 1070 were reviews. First-stage screening yielded 319 records, of which 68 were duplicates. 251 records were re-screened and discussed. The remaining 52 records underwent quality appraisal. This

resulted in 33 records from the search which were analysed. Alongside, snowball sampling was carried out and this yielded 12 records for analysis. The PRISMA diagram summarises the selection process (Figure 2).

3.1 Definitions of healthcare resilience

Most reviews took their definition of healthcare resilience from previous papers. Three reviews contained lists of definitions from other studies [17 35 36]. Another three reviews presented narrative collections and analyses of definitions [15 37 38]. We performed word occurrence analysis on all the definitions in the included reviews (Appendix 2).

Resilience tends to be defined based on paradigms within which it operates [36-38]. For example, within the engineering paradigm it is concerned with the ability of a structure or material to return to its original state [39]. However, within business and ecological systems, it includes the ability for an organisation or system to capitalise or improve after a shock [38 40 41]. A prominent definition of healthcare resilience is from Hollnagel as “the ability of the health care system (a clinic, a ward, a hospital, a county) to adjust its functioning prior to, during, or following events, and thereby sustain required operations under both expected and unexpected conditions” [42 43]. While this definition continues to be refined, it provides a frame to highlight several aspects which emerged from our word occurrence analysis too.

First is the recognition of different hierarchical levels of the healthcare system [43 44](Figure 3). There is consensus of these as micro-level (referring to individual), meso-level (hospital or organisation), macro-level (region or country)[43 44]. Several nuances continue to evade neat classification. For example, the team level does not neatly fit into micro- or meso-levels [45], and a community has been considered both meso- and macro-levels [37 38]. There is also emergence of a meta-level of healthcare resilience. This level focuses mainly on the climate crisis as a determinant of health and how it threatens healthcare resilience and exacerbates inequalities [8 46-48]. This transcends national boundaries and so must be considered on a meta-level.

Second, resilience spans pre-event, intra-event, and post-event. This appreciates contributions from different paradigms. DRR studies are mostly concerned with pre-event aspects, such as preparing for shocks thereby minimising disturbance to a system [13 49-53]. Emergency Management (EM) studies examine aspects of how organisations adapt during a crisis (intra-event), and how they may learn from it [17 54]. Some definitions do not include recovery, while others emphasise that rapidity of recovery back to steady state is a key feature of resilience [16 37 51 55].

For peer review only

Third, both expected and unexpected events are considered. DRR and EM paradigms are concerned with disasters and unexpected crises, which suggest that resilience is only manifest when a sufficiently large shock is applied to the system [35]. Patient safety paradigms seek to minimise unintended harm to patients, whether in crisis situations, or within normal operational stressors in healthcare [7 36 38 56]. Recently, even DRR and EM reviews have adopted such an "all-hazards" approach to resilience [1 48].

Hollnagel's definition of healthcare resilience, though widely used, does not specifically include recovery or review [42]. Instead, he refers to these concepts as "learn" within his related Resilience Analysis Grid (RAG) [57]. The resilience potentials in RAG are (1) anticipate, (2) respond, (3) monitor, and (4) learn. Many other definitions map well onto Hollnagel's definition and his four potentials. There are benefits in linking up definitions with "potentials" or concepts of resilience. It provides convergence in a complex field, which helps to develop a consistent approach across hierarchical levels. This may promote understanding between policymakers and healthcare practitioners (including leaders and managers). In turn, it may lead to more unified approaches across discipline and hierarchical boundaries.

We have therefore synthesised a definition from this analysis which complements previous work, spans across disciplines, and dovetails with concepts explored in the next section. Our definition of healthcare resilience is *the ability of healthcare workers, organisations or systems to (a) prepare for and prevent, (b) absorb and adapt to maintain structure and function, (c) recover and review from crises, shocks or stressors* (Figure 4).

3.2 Concepts of healthcare resilience

Concepts may refer to practices or features which promote or hinder resilience, or indicators of resilience. Groups of concepts are often collated in frameworks, or set as best fit into established frameworks (e.g. WHO building blocks of health). Frameworks are useful to systematically consider various aspects of healthcare resilience [48 58]. We highlight key concepts at macro- and meso-levels, paying attention to lessons learnt from the COVID19 pandemic. We then present our framework of concepts learnt from this review (Figure 5), which dovetail with our definition (Figure 4).

Physical capacities

Within the DRR and EM paradigms, strengthening of existing capacities feature most prominently. This includes adequate workforce [59], equipment [44 60], financial resources [4], and services [4 59] to ensure maintenance of function during a crisis. From

the COVID19 pandemic, appropriate distribution of physical capacities during various stages of a crisis are equally important. For example, changing shift patterns of emergency staff for increased demand intra-event, but redistributing staff to address longer-term care needs post-event [61]. This adopts a longitudinal approach to resilience past the acute crisis and helps to address predicted shortages. As such, it may then prevent mere shifting of crisis from one organisation to another.

Multi-level approaches

Several reviews have highlighted the multi-level approaches required to build resilience [12 43 62-65]. A small number of empirical studies are currently underway to understand how this works in practice [11 66]. Multi-level approaches recognise that while previous boundaries help understand specific details about how individual hierarchical levels pursue resilience, they are insufficient in representing the interconnections between levels. As an example, while improvisation and coping were seen as resilient concepts at the micro-level, healthcare workers' ability to continue working was underpinned by the availability of resources and protective equipment provided by the organisation and their leaders (meso-level), as well as recovery efforts introduced after the crisis at meso- and macro-level [4 44 60]. Further work is needed to better understand the interplay and dynamics between the hierarchical levels.

Recovery and Review

The most appropriate method of quality improvement to ensure learning after a crisis depends on context. It is more important for organisations to demonstrate consistency and commitment to change methodology, rather than any specific tool or method [67]. This is termed "review" in our framework. Within the patient safety paradigm, two prominent and complementary concepts are safety1 and safety2 approaches. The safety1 approach attempts to get to the root cause of adverse events in healthcare, while the safety2 approach recognises that success occurs more frequently than adverse events and therefore lessons can be learnt from such practices too [42].

Recovery is mentioned in DRR/EM literature [16 55 68], but less so in patient safety literature. This could be contextual. In DRR/EM, crises tend to be larger in scale and affect more people, organisations, and sectors outside of healthcare. Patient safety tends to focus on teams within clinical emergency scenarios or normal operational circumstances. DRR/EM paradigms therefore require a more concerted recovery effort. Rapidity of recovery has been cited as a feature of resilience, but there is no benchmark. Extent of recovery to previous function has been explored, but this does not appreciate the occasional change in function of a system following a large crisis [14 37]. Several reviews have emphasised the importance of having plans for recover within resilience frameworks, but do not elaborate on details [44 59 60]. Therefore, there is still much to understand about recovery, and some recent frameworks summarise key lessons learnt from COVID19 [20].

For peer review only

Relational aspects

The COVID19 pandemic has highlighted the importance of relational aspects of resilience. A key aspect is effective leadership [1 13 17 37 48 50 51 54 56 61-63 65 68-73]. Collaborative and authentic styles which were visible and approachable seemed most favoured during crises[1 2 4 12 17 55 56 68 71 72 74 75]. Command-and-control structures have been cited, but these refer more to the flow of information rather than organisation of authority. Consequently, more recent DRR reviews advocate for decisions to be pushed out towards the periphery, enabling more rapid adaptation to change [16 51 54 59].

Networks beyond formal healthcare facilities bolsters resilience through the availability of physical capacities (e.g. financing, staff, equipment and volunteers) [1 13 16 37 61 62 68 73 74 76]. It also facilitates adaptation and planning post-event. It also facilitates post-event concepts. For example, COVID19 clinical and vaccine trials depended on international collaboration between many disciplines and industries [5]. Recognising the importance of this concept, the recent UK Health and Care Act 2022 specifically focuses on inter-organisational relationships towards the provision of integrated care. Further research will therefore be required to better understand how these relationships work in practice [77].

An interdisciplinary, cross-sectoral, and multi-level framework for healthcare resilience

These concepts, along with others which emerged from analysis of the reviews, have been compiled into an interdisciplinary, cross-sectoral, and multi-level framework (Figure 4 & 5, Appendix 3). It spans meso- and macro-levels, which few existing frameworks do. It balances the historical focus on physical capacities with relational aspects of resilience. It incorporates lessons learnt from the COVID19 pandemic. The framework may be used as tool to systematically consider various aspects of organisational and systems resilience.

The framework is divided into timeframes (pre-, intra-, post-, and trans-event). Within timeframes are themes (prepare and prevent etc). Themes encompass several concepts, and finally, these concepts are further broken down into components (Figure 4 & 5). In Figure 4, the size of faint circles indicates the frequency of concepts extracted from the reviews, which are also represented as n-numbers in Figure 5.

3.3 Measures of resilience

Due to the considerable global interest in healthcare resilience, there have been attempts to develop measures or indicators for resilience. The process of pursuing resilience requires ways to determine progression. Attempts to measure resilience therefore allow comparisons and identification of potential areas for improvement. However, none of the current measures have been validated, and some widely used

For peer review only

indices have been shown to be inaccurate during the COVID19 pandemic.

Two examples at the Macro-level are the Global Health Security Index (GHSI) and Epidemic Preparedness Index (EPI). They both focus on infectious diseases outbreaks and contain several domains including public health and healthcare infrastructure (e.g. surveillance capabilities, medical workforce), economic resources, and risk assessment and communication strategies [2 78]. However, many High-Income Countries (HICs) that scored well on these indices performed very poorly during the COVID19 pandemic [79]. Several reasons have been cited, including lack of consideration of existing health inequalities, globalisation, societal connectedness, and a bias towards physical capacities (e.g. number of hospitals or beds)[79]. Of note, increased globalisation suggests that ”disease control may be only as effective as practices within the poorest performing countries” [79]. This supports the need for a meta-level of resilience where global health inequalities affect the healthcare resilience of other nations.

Some reviews attempt to evaluate a country’s resilience according to one or more domains of the WHO building blocks of health framework [14 59 65 69 80]. However, the framework was designed as a systematic approach to the funding decisions within healthcare, rather than to evaluate resilience [36].

Meso-level measures tend to stem from DRR/EM paradigms, focused on healthcare facilities. Several reviews have collated evidence from empirical papers centred around earthquakes and natural disasters. Quantitative measures include physical capacities (e.g. number of beds, staff, equipment), physical infrastructure (e.g. structural integrity of buildings)[1], and actuality:potentiality ratios (e.g. adherence to operating protocols)[37 81]. Qualitative indicators include checklists containing several components important during disaster situations (e.g. pre-emptive protocols and procedures, command structures, compliance to structural regulations)[16 68].

In the patient safety paradigm, the Checklist for Assessing Institutional Resilience (CAIR) contains broad domains to help hospitals consider several concepts of resilience [82]. It was not designed as a set of measures but may be considered a tool for systematic thinking in patient safety. Similarly, while some have suggested that the work-as-imagined vs work-as-done (WAI/WAD) ratio (or the more general actuality:potentiality ratio) may be used as a way to improve patient safety (by minimising performance variability)[17], others highlighted that this approach may work for some simple processes, but is unlikely to result in favourable patient outcomes in unexpected or complex scenarios [43]. For example, adherence to sepsis guidelines (WAI) is audited and associated with overall better outcomes [83], but depends on accurate diagnosis, which is itself difficult. Sepsis is frequently misdiagnosed (WAD), leading to wrong treatments, antimicrobial resistance, and poor outcomes [84 85].

For peer review only

Therefore, WAI/WAD is not an adequate approach to measure resilience. Instead, WAI/WAD should be used as means to understand the balance struck within systems, and a concept with which to frame observations about adaptations in healthcare. This reframes resilience from an outcome-oriented approach to a process-oriented one.

4. Discussion

We have employed a relatively new review methodology well-suited for complex topics informed by various research traditions. There is increasing acknowledgement of the need for interdisciplinary, cross-sectoral, and multi-level approaches to healthcare resilience. Yet, its history has been one of multiple research traditions operating within specific paradigms. Global health paradigms dominate the macro-level, while disaster risk reduction and patient safety frame the meso-level.

Definitions of healthcare resilience are increasingly broad. They have evolved from highly contextualised situations towards appreciation of different hierarchical levels and from an outcome-oriented understanding towards one which is more process-oriented. With this evolution, it has become more difficult to characterise failure of resilience. Even though healthcare systems and organisations are affected by a range of stressors, they are inherently resilient as essential societal services. Recognising the interplay between hierarchical levels is vital for building resilience. For example, the Institute for Healthcare Improvement framework for improving joy in work considers meso- and macro-level aspects (e.g. participative management and an environment of psychological safety), that affect micro-level resilience, and thus, explicitly links previously distinct hierarchical levels [86]. Such inter-level frameworks rarely feature at the meso- and macro-levels, despite healthcare workers being arguably the most important resources of healthcare systems. Several recent study protocols have been published to better understand multi-level influences in resilience, particularly in the patient safety paradigm [11 66].

Put another way, resilience concepts at macro-level have tended to focus on physical capacities prior to COVID19. Yet, there is increasing recognition of the importance of relational aspects of resilience, particularly across macro- and meso-level boundaries. Some examples of these within the COVID19 pandemic include the adoption of more open leadership at meso- and macro-levels [60], interorganisational relationships and their realignment [4 62], cultivating an environment of trust [44 61], and open communication channels at micro-, meso- and macro-levels [87]. Open leadership styles gained more trust and enabled better understanding of WAI/WAD [44]. Alongside, the open channels of communication between healthcare workers, managers and in turn national bodies helped with rapid adaptation and identification of acute needs, from equipment and supply issues to psychological support services [60].

While a range of tools and indicators have been developed for physical capacities and infrastructure, it is still not known how best to assess the effectiveness of relational aspects of resilience. In particular, understanding the nature of interorganisational relationships seems to stand out as a research priority.

Our framework for healthcare resilience fulfils several needs in this field. First, it brings together knowledge and observations gained from a range of disciplines, across hierarchical levels, and integrates it with lessons learnt from the COVID19 pandemic. Second, it emphasises both physical and relational aspects of resilience, the latter of which has received less attention in the past. Finally, it dovetails with our definition and achieves some convergence in a highly heterogenous field.

There are several strengths to this study. Firstly, a review of reviews provides a broad overview of the topic across several geopolitical contexts. Reviews included countries and hospitals with different health financing models, health inequalities, geographies, and range of services (Appendix 3). There is consolidation of knowledge across a wide range of disciplines and paradigms, enabling wider appreciation of the complexities of the topic and the many ways it has been approached. Secondly, a meta-narrative approach helps to explore historical trends and ongoing debates within a complex field. In addition to the consolidation of knowledge in the previous point, this approach also acknowledges the differences between paradigms and broadens the understanding of resilience (Figure 3, Appendix 4). Thirdly, we have taken a multi-level approach at meso- and macro-levels, which has hitherto been rare. These three points provide a map for healthcare workers and health systems managers to better understand the contexts within which each party operates [3]. Fourthly, we have highlighted several lessons learn from the COVID19 pandemic, which was a shock to entire healthcare systems across the world. Because of the range of geopolitical contexts covered, extracted lessons may be applied across different healthcare systems and organisations. Our framework increases the focus on relational aspects of resilience, particularly in the trans-event domains (Figures 4 & 5). The importance of which is also reflected within the recently renewed interest into integrated care in the UK.

Several limitations persist. We do not focus on the micro-level, even though humans are undoubtedly the most important components of healthcare systems, both as staff and service-users. Empirical studies are excluded. This means that while we were able to maintain breadth, we inadvertently lose resolution. Nevertheless, frameworks can be considered tools for systematically approaching resilience, but require adaptation towards other more contextualised situations (Appendix 4). Knowledge continues to be discovered and some will not have made it into reviews yet. Therefore, while this review of reviews is timely, we acknowledge that resilience is a dynamic process [29]. We have provided only limited guidance in individual aspects

For peer review only - http://bmjopen.bmj.com/site/about/guidelines.xhtml

such as health financing, infrastructure, or leadership styles. There is a wealth of literature for these purposes. We instead focus on a broad overview across disciplines and hierarchical levels.

Future priorities arising from this research include the need to examine multi-level resilience with empirical studies [11], exploring how each level's resilience contributes to other levels [3], and gaining deeper understanding of the nature and extent of interorganisational relationships and how they affect resilience.

5. Conclusion

Our review of reviews provides a broad overview of healthcare resilience. The meta-narrative approach adopted recognises the contribution from several disciplines and paradigms within this field. We have highlighted the tendency for studies to focus on individual hierarchical levels and limited timescales, but also acknowledge the need and current desire to develop more interdisciplinary, cross-sectoral, and multi-level approaches to healthcare resilience. Our framework for healthcare resilience crosses hierarchical and time boundaries, providing a broad and reflexive overview of healthcare resilience. Understanding the balances between sometimes opposing concepts, and contrasting priorities of different professional groups or hierarchies, is key to building resilience for the future.

For peer review only

Contributorship statement

Concept – MT, AMcC, PD. Planning and design – MT. Screening – MT, GP. Additional reviewers – AMcC, PD. Data collection, analysis, and interpretation – MT, GP. Writing – MT. Editing – all authors

Competing interests

None declared.

Funding

There is no funding associated with this research. MT and GP are academic clinical fellows. This programme allows clinicians protected time to undertake and develop their research skills alongside clinical practice.

Data sharing statement

All data relevant to the study are included in the article or uploaded as supplementary information

Ethics Approval

This study does not involve human participants and ethical approval was not required

Patient and Public Involvement

There was no patient or public involvement in this research.

For peer review only

References

1. Barasa E, Mbau R, Gilson L. What Is Resilience and How Can It Be Nurtured? A Systematic Review of Empirical Literature on Organizational Resilience. *International Journal of Health Policy and Management* 2018;7(6):491-503. doi: 10.15171/ijhpm.2018.06
2. Oppenheim B, Gallivan M, Madhav NK, et al. Assessing global preparedness for the next pandemic: development and application of an Epidemic Preparedness Index. *BMJ Global Health* 2019;4(1):e001157. doi: 10.1136/bmjgh-2018-001157
3. Tan MZY. Resilience is a dirty word: misunderstood, and how we can truly build it. *Critical Care* 2022;26(1):168. doi: 10.1186/s13054-022-04040-x
4. Meyer D, Bishai D, Ravi SJ, et al. A checklist to improve health system resilience to infectious disease outbreaks and natural hazards. *BMJ Glob Health* 2020;5(8) doi: 10.1136/bmjgh-2020-002429
5. Park JJH, Mogg R, Smith GE, et al. How COVID-19 has fundamentally changed clinical research in global health. *The Lancet Global Health* 2021;9(5):e711-e20. doi: https://doi.org/10.1016/S2214-109X(20)30542-8
6. Razai MS, Chaudhry UAR, Doerholt K, et al. Covid-19 vaccination hesitancy. *BMJ* 2021;373:n1138. doi: 10.1136/bmj.n1138
7. Pillay M, Morel G. Measuring Resilience Engineering: An Integrative Review and Framework for Bench-Marking Organisational Safety. *Safety* 2020;6(3) doi: 10.3390/safety6030037
8. Curtis S, Fair A, Wistow J, et al. Impact of extreme weather events and climate change for health and social care systems. *Environmental Health: A Global Access Science Source* 2017;16 doi: 10.1186/s12940-017-0324-3
9. Greenhalgh T, Robert G, Macfarlane F, et al. Storylines of research in diffusion of innovation: a meta-narrative approach to systematic review. *Soc Sci Med* 2005;61(2):417-30. doi: 10.1016/j.socscimed.2004.12.001 [published Online First: 2005/05/17]
10. Mondal S, Van Belle S, Maioni A. Learning from intersectoral action beyond health: a meta-narrative review. *Health Policy Plan* 2021;36(4):552-71. doi: 10.1093/heapol/czaa163
11. Aase K, Guise V, Billett S, et al. Resilience in Healthcare (RiH): A longitudinal research programme protocol. *BMJ Open* 2020;10(10) doi: 10.1136/bmjopen-2020-038779
12. Wiig S, Aase K, Billett S, et al. Defining the boundaries and operational concepts of resilience in the resilience in healthcare research program. *BMC Health Services Research* 2020;20(1) doi: 10.1186/s12913-020-05224-3
13. Li L, Liao S, Yuan J, et al. Analyzing Healthcare Facility Resilience: Scientometric Review and Knowledge Map. *Frontiers in Public Health* 2021;9 doi: 10.3389/fpubh.2021.764069
14. Biddle L, Wahedi K, Bozorgmehr K. Health system resilience: A literature review of empirical research. *Health Policy and Planning* 2020;35(8):1084-109. doi: 10.1093/heapol/czaa032

For peer review only - http://bmjopen.bmj.com/site/about/guidelines.xhtml

15. Koeva S, Rohova M. Health system resilience: concept development. *Journal of Imab* 2020;26(3):3251-58. doi: 10.5272/jimab.2020263.3251
16. Zhong S, Clark M, Hou X-Y, et al. Development of hospital disaster resilience: conceptual framework and potential measurement. *Emergency Medicine Journal* 2014;31(11):930-U8901. doi: 10.1136/emermed-2012-202282
17. Son C, Sasangohar F, Neville T, et al. Investigating resilience in emergency management: An integrative review of literature. *Appl Ergon* 2020;87 doi: 10.1016/j.apergo.2020.103114
18. Almedom AM. Resilience research and policy/practice discourse in health, social, behavioral, and environmental sciences over the last ten years. *African health sciences* 2008;8 Suppl 1:S5-13.
19. Calkins J. Moving forward after Sendai: How countries want to use science, evidence and technology for disaster risk reduction. *PLoS Currents* 2015;7(DISASTERS) doi: 10.1371/currents.dis.22247d6293d4109d09794890bcda1878
20. McClelland AG, Shaw D, O'Grady N, et al. Recovery for Development: A Multi-Dimensional, Practice-Oriented Framework for Transformative Change Post-Disaster. *The Journal of Development Studies* 2022:1-20. doi: 10.1080/00220388.2022.2130055
21. Conti A, Valente M, Paganini M, et al. Knowledge Gaps and Research Priorities on the Health Effects of Heatwaves: A Systematic Review of Reviews. *Int J Environ Res Public Health* 2022;19(10) doi: 10.3390/ijerph19105887 [published Online First: 20220512]
22. Ries M. The COVID-19 Infodemic: Mechanism, Impact, and Counter-Measures—A Review of Reviews. *Sustainability* 2022;14(5):2605.
23. Gough D. Meta-narrative and realist reviews: guidance, rules, publication standards and quality appraisal. *BMC Medicine* 2013;11(1):22. doi: 10.1186/1741-7015-11-22
24. Wong G, Greenhalgh T, Westhorp G, et al. RAMESES publication standards: meta-narrative reviews. *BMC Medicine* 2013;11(1):20. doi: 10.1186/1741-7015-11-20
25. Tan M, Prager G. Resilience in healthcare: a meta-narrative review of reviews. CRD42022314729: PROSPERO; 2022 [Available from: https://www.crd.york.ac.uk/prospero/display_record.php?ID=CRD42022314729 accessed Mar 2022.
26. Butler A, Hall H, Copnell B. A Guide to Writing a Qualitative Systematic Review Protocol to Enhance Evidence-Based Practice in Nursing and Health Care. *Worldviews Evid Based Nurs* 2016;13(3):241-9. doi: 10.1111/wvn.12134 [published Online First: 20160120]
27. Page MJ, McKenzie JE, Bossuyt PM, et al. The PRISMA 2020 statement: an updated guideline for reporting systematic reviews. *BMJ* 2021;372:n71. doi: 10.1136/bmj.n71
28. Gurney A. Resilience in the UK and other OECD economies: Treasury Economic Working Paper No.2 London: HM Treasury; 2008 [Available from: https://webarchive.nationalarchives.gov.uk/ukgwa/20081112160625/http:/www.hm-treasury.gov.uk/d/bud08_workingpaper2_557_.pdf.

For peer review only

29. Bealt J, Powell D, Shaw D. Benefits of involving public involvement in emergency planning Manchester: University of Manchester; 2021 [Available from: https://committees.parliament.uk/writtenevidence/21957/pdf/ accessed Aug 2021.
30. Abdullah MA, Shaikh BT. Confusion and denial: need for systems thinking to understand the HIV epidemic in Pakistan. *J Ayub Med Coll Abbottabad* 2014;26(3):396-400.
31. Smith V, Devane D, Begley CM, et al. Methodology in conducting a systematic review of systematic reviews of healthcare interventions. *BMC Med Res Methodol* 2011;11(1):15. doi: 10.1186/1471-2288-11-15 [published Online First: 20110203]
32. Smith V, Devane D, Begley CM, et al. Methodology in conducting a systematic review of systematic reviews of healthcare interventions. *BMC Medical Research Methodology* 2011;11(1):15. doi: 10.1186/1471-2288-11-15
33. Aromataris E, Fernandez R, Godfrey C, et al. Summarizing systenatic reviews: methodological development, conduct and reporting of an Umbrella review approach. . *Int J Evid Based Healthc* 2015;13:132-40.
34. Noble H, Mitchell G. What is grounded theory? *Evidence Based Nursing* 2016;19(2):34-35. doi: 10.1136/eb-2016-102306
35. Brand FS, Jax K. Focusing the Meaning(s) of Resilience: Resilience as a Descriptive Concept and a Boundary Object. *Ecology and Society* 2007;12(1)
36. Turenne CP, Gautier L, Degroote S, et al. Conceptual analysis of health systems resilience: A scoping review. *Soc Sci Med* 2019;232:168-80. doi: 10.1016/j.socscimed.2019.04.020 [published Online First: 20190430]
37. Hosseini S, Barker K, Ramirez-Marquez JE. A review of definitions and measures of system resilience. *Reliability Engineering & System Safety* 2016;145:47-61. doi: https://doi.org/10.1016/j.ress.2015.08.006
38. Augustynowicz A, Opolski J, Waszkiewicz M. Resilient Health and the Healthcare System. A Few Introductory Remarks in Times of the COVID-19 Pandemic. *Int J Environ Res Public Health* 2022;19(6) doi: 10.3390/ijerph19063603
39. Zhong S, Clark M, Hou X-Y, et al. Proposing and developing a definition and conceptual framework for health care resilience to cope with disasters. *Emergencias* 2014;26(1):69-77.
40. Biggs R, Schlüter M, Schoon ML. Principles for Building Resilience: Cambridge University Press 2015.
41. Florez-Jimenez M, Muñoz-Villamizar A, Lleo A. Exploring the relationship between sustainability, resilience, and purpose in the context of corporations: a comprehensive literature review SSRN; 2021 [Available from: https://papers.ssrn.com/sol3/papers.cfm?abstract_id=3944148 accessed Nov 2021.
42. Hollnagel E, Wears R, Braithwaite J. From Safety-I to Safety-II: A White Paper. 2015.
43. Berg SH, Akerjordet K, Ekstedt M, et al. Methodological strategies in resilient health care studies: An integrative review. *Safety Science* 2018;110:300-12. doi: 10.1016/j.ssci.2018.08.025

For peer review only

44. Rangachari P, J LW. Preserving Organizational Resilience, Patient Safety, and Staff Retention during COVID-19 Requires a Holistic Consideration of the Psychological Safety of Healthcare Workers. *Int J Environ Res Public Health* 2020;17(12) doi: 10.3390/ijerph17124267 [published Online First: 2020/06/19]
45. Stoverink AC, Kirkman BL, Mistry S, et al. Bouncing Back Together: Toward a Theoretical Model of Work Team Resilience. *Academy of Management Review* 2020;45(2):395-422. doi: 10.5465/amr.2017.0005
46. Ayuningtyas D, Windiarti S, Sapoan Hadi M, et al. Disaster preparedness and mitigation in indonesia: A narrative review. *Iranian Journal of Public Health* 2021;50(8):1536-46.
47. Romanello M, McGushin A, Di Napoli C, et al. The 2021 report of the Lancet Countdown on health and climate change: code red for a healthy future. *The Lancet* 2021;398(10311):1619-62. doi: 10.1016/S0140-6736(21)01787-6
48. Kruk ME, Ling EJ, Bitton A, et al. Building resilient health systems: a proposal for a resilience index. *BMJ* 2017;357:j2323. doi: 10.1136/bmj.j2323
49. Carrington MA, Ranse J, Hammad K. The impact of disasters on emergency department resources: review against the Sendai framework for disaster risk reduction 2015–2030. *Australas Emerg Care* 2021;24(1):55-60. doi: 10.1016/j.auec.2020.09.003
50. Cartwright C, Hall M, Lee ACK. The changing health priorities of earthquake response and implications for preparedness: a scoping review. *Public Health* 2017;150:60-70. doi: 10.1016/j.puhe.2017.04.024
51. Challen K, Lee AC, Booth A, et al. Where is the evidence for emergency planning: A scoping review. *BMC Public Health* 2012;12(1) doi: 10.1186/1471-2458-12-542
52. Fallah-Aliabadi S, Ostadtaghizadeh A, Ardalan A, et al. Towards developing a model for the evaluation of hospital disaster resilience: A systematic review. *BMC Health Services Research* 2020;20(1) doi: 10.1186/s12913-020-4915-2
53. Luke J, Franklin R, Aitken P, et al. Safer hospital infrastructure assessments for socio-natural disaster - A scoping review. *Prehosp Disaster Med* 2021;36(5):627-35. doi: 10.1017/S1049023X21000650
54. Xin YT, Xu KY. Hospital emergency management research in China: trends and challenges. *Emerg Med J* 2012;29(5):353-7. doi: 10.1136/emermed-2011-200512 [published Online First: 20111103]
55. Nuzzo JB, Meyer D, Snyder M, et al. What makes health systems resilient against infectious disease outbreaks and natural hazards? Results from a scoping review. *BMC Public Health* 2019;19(1) doi: 10.1186/s12889-019-7707-z
56. Iflaifel M, Lim RH, Ryan K, et al. Resilient Health Care: a systematic review of conceptualisations, study methods and factors that develop resilience. *BMC Health Serv Res* 2020;20(1):324. doi: 10.1186/s12913-020-05208-3 [published Online First: 2020/04/19]
57. Hollnagel E. Epilogue: RAG–the resilience analysis grid. Resilience engineering in practice: CRC Press 2017:275-96.
58. WHO. Monitoring the building blocks of health systems 2010 [accessed Nov 2019.
59. Tessema GA, Kinfu Y, Dachew BA, et al. The COVID-19 pandemic and

healthcare systems in Africa: a scoping review of preparedness, impact and response. *Bmj Global Health* 2021;6(12) doi: 10.1136/bmjgh-2021-007179
60. Turner S, Botero-Tovar N, Herrera MA, et al. Systematic review of experiences and perceptions of key actors and organisations at multiple levels within health systems internationally in responding to COVID-19. *Implementation Science* 2021;16(1) doi: 10.1186/s13012-021-01114-2
61. Alami H, Lehoux P, Fleet R, et al. How Can Health Systems Better Prepare for the Next Pandemic? Lessons Learned From the Management of COVID-19 in Quebec (Canada). *Frontiers in public health* 2021;9:671833. doi: 10.3389/fpubh.2021.671833
62. Grimm PY, Oliver S, Merten S, et al. Enhancing the Understanding of Resilience in Health Systems of Low- and Middle-Income Countries: A Qualitative Evidence Synthesis. *International Journal of Health Policy and Management* 2021 doi: 10.34172/ijhpm.2020.261
63. Kamara JK, Akombi BJ, Agho K, et al. Resilience to climate-induced disasters and its overall relationship to well-being in southern africa: A mixed-methods systematic review. *Int J Environ Res Public Health* 2018;15(11) doi: 10.3390/ijerph15112375
64. Tippong D, Petrovic S, Akbari V. A Review of Applications of Operational Research in Healthcare Coordination in Disaster Management. *Eur J Oper Res* 2021 doi: 10.1016/j.ejor.2021.10.048 [published Online First: 20211028]
65. Van Ryneveld M, Schneider H, Lehmann U. Looking back to look forward: a review of human resources for health governance in South Africa from 1994 to 2018. *Human Resources for Health* 2020;18(1) doi: 10.1186/s12960-020-00536-1
66. Anderson JE, Aase K, Bal R, et al. Multilevel influences on resilient healthcare in six countries: An international comparative study protocol. *BMJ Open* 2020;10(12) doi: 10.1136/bmjopen-2020-039158
67. HealthFoundation. Quality improvement made simple: What everyone should know about health care quality improvement: The Health Foundation; 2021 [Available from: https://www.health.org.uk/sites/default/files/QualityImprovementMadeSimple.pdfOct 2022.
68. Rezaei F, Maracy MR, Yarmohammadian MH, et al. Hospitals preparedness using WHO guideline: A systematic review and meta-analysis. *Hong Kong Journal of Emergency Medicine* 2018;25(4):211-22. doi: 10.1177/1024907918760123
69. Ayanore MA, Amuna N, Aviisah M, et al. Towards Resilient Health Systems in Sub-Saharan Africa: A Systematic Review of the English Language Literature on Health Workforce, Surveillance, and Health Governance Issues for Health Systems Strengthening. *Annals of Global Health* 2019;85(1) doi: 10.5334/aogh.2514
70. Hasan MZ, Neill R, Das P, et al. Integrated health service delivery during COVID-19: A scoping review of published evidence from low-income and lower-middle-income countries. *BMJ Global Health* 2021;6(6) doi: 10.1136/bmjgh-2021-005667
71. Ravi SJ, Meyer D, Cameron E, et al. Establishing a theoretical foundation for

For peer review only

measuring global health security: A scoping review. *BMC Public Health* 2019;19(1) doi: 10.1186/s12889-019-7216-0
72. Fridell M, Edwin S, Schreeb JV, et al. Health system resilience: what are we talking about? A scoping review mapping characteristics and keywords. *International Journal of Health Policy and Management* 2020;9(1):6-16. doi: 10.15171/ijhpm.2019.71
73. Blumenstock J, Bakker G, Jarris PE. Measuring Preparedness: The National Health Security Preparedness Index. *Journal of Public Health Management and Practice* 2014;20(3):361-63. doi: 10.1097/phh.0000000000000073
74. Haldane V, Zhang Z, Abbas RF, et al. National primary care responses to COVID-19: a rapid review of the literature. *BMJ Open* 2020;10(12):e041622. doi: 10.1136/bmjopen-2020-041622 [published Online First: 20201208]
75. Lapão LV, Silva A, Pereira N, et al. Ebola impact on African health systems entails a quest for more international and local resilience: the case of African Portuguese speaking countries. *Pan Afr Med J* 2015;22 Suppl 1(Suppl 1):15. doi: 10.11694/pamj.supp.2015.22.1.6653 [published Online First: 20151011]
76. Tin N, Lwin S, Kyaing NN, et al. An approach to health system strengthening in the Union of Myanmar. *Health Policy* 2010;95(2-3):95-102. doi: 10.1016/j.healthpol.2009.11.013
77. Sanderson M, Allen P, Osipovic D, et al. The Developing Architecture of System Management: Integrated Care Systems and Sustainability and Transformation Partnerships - Interim report: PRUComm; 2021 [Available from: https://prucomm.ac.uk/assets/uploads/PRUComm_ICS_study_interim_report_feb_2021.pdf accessed Jul 2022.
78. Bell JA, Nuzzo JB. Global Health Security Index: Advancing Collective Action and Accountability Amid Global Crisis: NTI/John Hopkins center for health security; 2021 [Available from: www.GHSIndex.org accessed Mar 2022.
79. Baum F, Freeman T, Musolino C, et al. Explaining covid-19 performance: what factors might predict national responses? *BMJ* 2021;372:n91. doi: 10.1136/bmj.n91
80. Rameshshanker V, Wyngaarden S, Lau LL, et al. Health system resilience to extreme weather events in Asia-Pacific: a scoping review. *Clim Dev* 2021;13(10):944-58. doi: 10.1080/17565529.2020.1870425
81. Penaloza GA, Saurin TA, Formoso CT, et al. A resilience engineering perspective of safety performance measurement systems: A systematic literature review. *Safety Science* 2020;130 doi: 10.1016/j.ssci.2020.104864
82. Carthey J, de Leval MR, Reason JT. Institutional resilience in healthcare systems. *Quality in Health Care* 2001;10(1):29-32. doi: 10.1136/qhc.10.1.29
83. Evans L, Rhodes A, Alhazzani W, et al. Surviving sepsis campaign: international guidelines for management of sepsis and septic shock 2021. *Intensive Care Med* 2021;47(11):1181-247. doi: 10.1007/s00134-021-06506-y [published Online First: 20211002]
84. Fitzpatrick F, Tarrant C, Hamilton V, et al. Sepsis and antimicrobial stewardship: two sides of the same coin. *BMJ Quality & Safety* 2019;28(9):758-61. doi: 10.1136/bmjqs-2019-009445
85. Vincent JL. The Clinical Challenge of Sepsis Identification and Monitoring. *PLoS*

For peer review only

Med 2016;13(5):e1002022. doi: 10.1371/journal.pmed.1002022 [published Online First: 20160517]
86. Smith AF, Plunkett E. People, systems and safety: resilience and excellence in healthcare practice. *Anaesthesia* 2019;74(4):508-17. doi: 10.1111/anae.14519
87. Raymond CB, Ward PR. Community-level experiences, understandings, and responses to COVID-19 in low-and middle-income countries: A systematic review of qualitative and ethnographic studies. *Int J Environ Res Public Health* 2021;18(22) doi: 10.3390/ijerph182212063

For peer review only

Figure captions

Figure 1: Search strategy

Figure 2: PRISMA diagram

Figure 3: Healthcare resilience: hierarchical levels and selected paradigm timelines with historical highlights. *Legend: WHO = World Health Organisation. DRR: Disaster Risk Reduction. FEMA: Federal Emergency Management Agency. GHSI: Global Health Security Index.*

Figure 4: An interdisciplinary, cross-sectoral, and multi-level definition and conceptual framework for healthcare resilience. *Legend: Defining features are listed as headings within the pre-, intra-, post- and trans-event time domains.*

Figure 5: Details of the interdisciplinary, cross-sectoral, and multi-level framework for healthcare resilience. Concepts are presented with frequency of occurrences as n-numbers. They are further divided into components, along with example considerations extracted from reviews.

For peer review only

Search Strategy

Scopus (Elsevier)

(TITLE-ABS-KEY ("resilien*"OR"strengthening") AND TITLE-ABS-KEY ("system"OR"organisation") AND TITLE-ABS-KEY ("health"OR"hospital"OR"healthcare") AND TITLE-ABS-KEY ("crisis"OR"disaster"OR“epidemic”OR“pandemic”))

Web of Science (Clarivate)

("resilience" OR "resilient" OR "strengthen") AND ("health") AND ("organisation" OR "systems") AND ("crisis" OR "disaster" OR "epidemic" OR "pandemic”)

PubMed (NIH)

((("resilien*"OR"strengthening") AND ("system"OR"organisation")) AND (health)) AND ("crisis"OR"disaster"OR"epidemic"OR"pandemic”)

Global Health Index (WHO)

(tw:(Resilien* OR strengthening)) AND (tw:(System OR Organisation))

Inclusion Criteria

- 2008 - 2022
- Scholarly articles (reviews) published in peer-reviewed academic databases
- Related to healthcare organisations and systems

Exclusion Criteria

- Non-healthcare
- Focused on individual, psychological or community resilience
- Concerned with a particular disease or health condition outside of epidemic/pandemic context
- Non-English

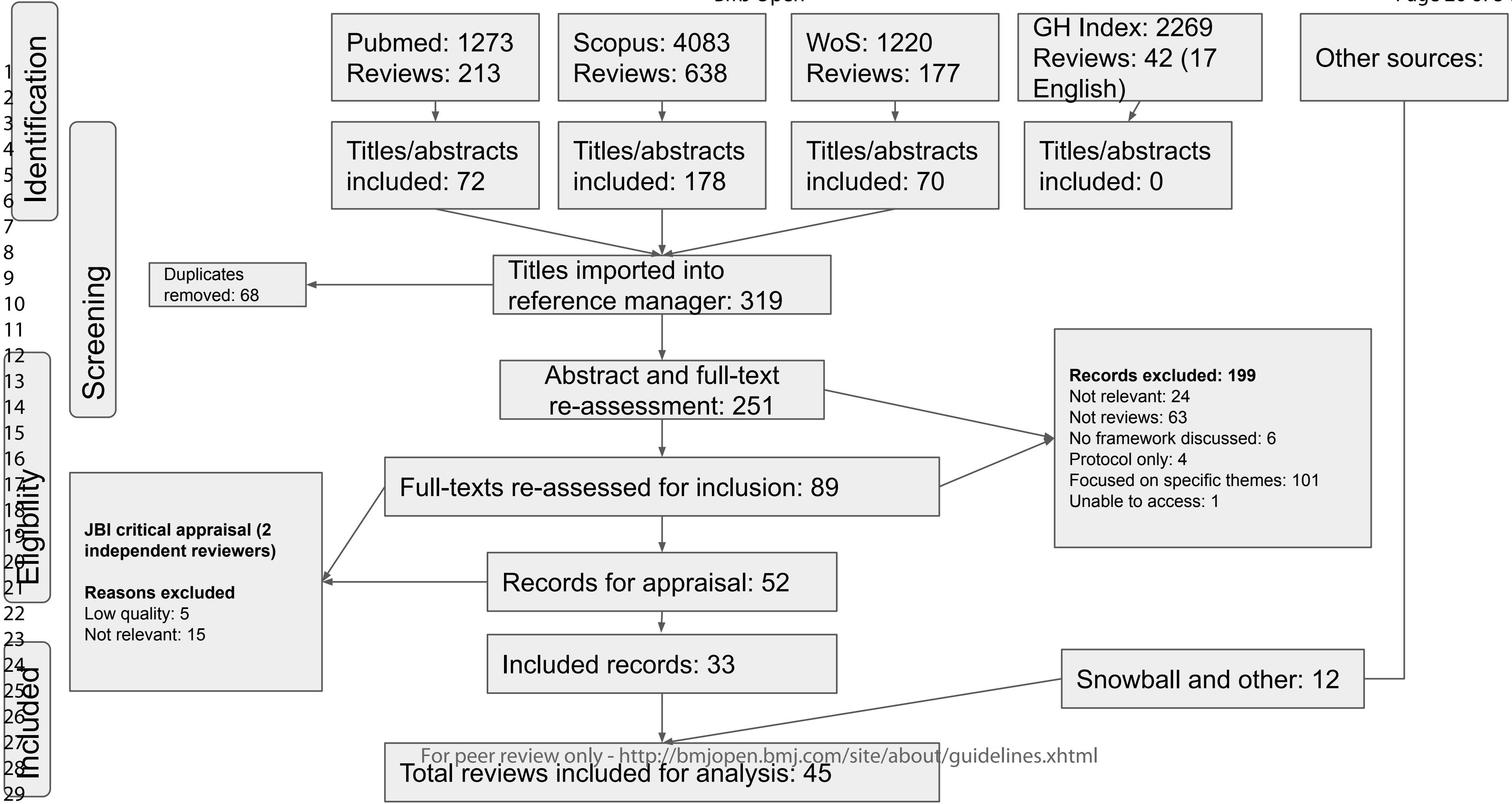

For peer review only - http://bmjopen.bmj.com/site/about/guidelines.xhtml

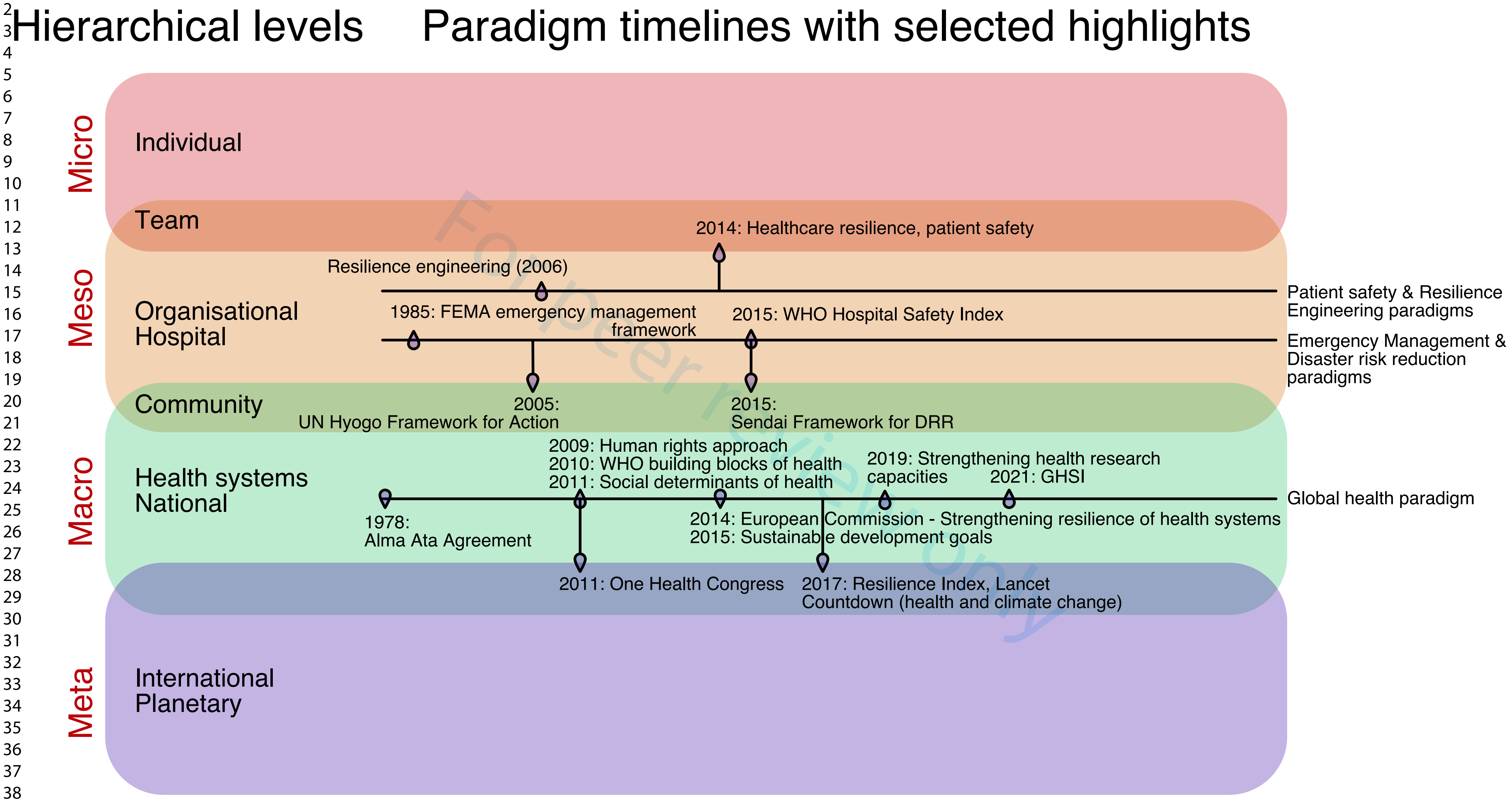
Hierarchical levels
Paradigm timelines with selected highlights
Micro
Individual
Team
Meso
Organisational
Hospital
Community
Macro
Health systems
National
Meta
International
Planetary
2014: Healthcare resilience, patient safety
Resilience engineering (2006)
Patient safety & Resilience Engineering paradigms
1985: FEMA emergency management framework
2015: WHO Hospital Safety Index
Emergency Management & Disaster risk reduction paradigms
2005: UN Hyogo Framework for Action
2015: Sendai Framework for DRR
2009: Human rights approach
2010: WHO building blocks of health
2011: Social determinants of health
2019: Strengthening health research capacities
2021: GHSI
Global health paradigm
1978: Alma Ata Agreement
2014: European Commission - Strengthening resilience of health systems
2015: Sustainable development goals
2011: One Health Congress
2017: Resilience Index, Lancet Countdown (health and climate change)

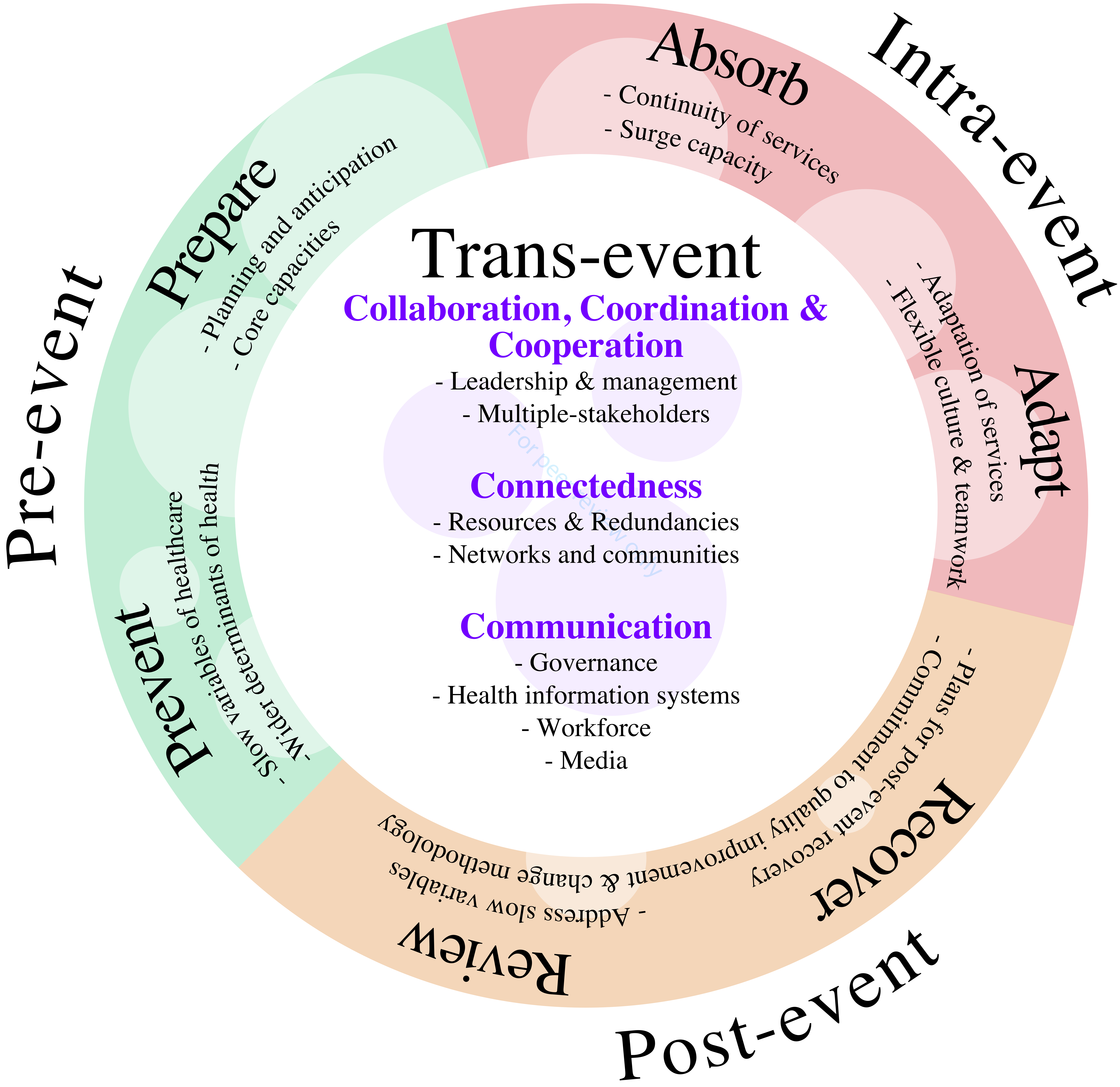
Trans-event
Collaboration, Coordination & Cooperation
- Leadership & management
- Multiple-stakeholders
Connectedness
- Resources & Redundancies
- Networks and communities
Communication
- Governance
- Health information systems
- Workforce
- Media
Pre-event
Prepare
- Planning and anticipation
- Core capacities
Prevent
- Slow variables of healthcare
- Wider determinants of health
Intra-event
Absorb
- Continuity of services
- Surge capacity
Adapt
- Adaptation of services
- Flexible culture & teamwork
Post-event
Recover
- Plans for post-event recovery
- Commitment to quality improvement & change methodology
Review
- Address slow variables

Interdisciplinary, Cross-sectoral, Multi-level framework for Healthcare Resilience

	Themes	Concepts (frequency)	Component	Example considerations
Pre-event	Prevent	Slow variables (n=8)	Healthcare access and range of services	Universal healthcare, geographical access factors, equality in access
			Acting on risks identified	Health inequalities, at risk sub-populations
			Strengthening healthcare infrastructure	Ventilation and air-conditioning systems, integrity of buildings, additional cliincal spaces
			Healthcare expenditure and funding channels	WHO Building Blocks of Health
			Staff retention and education	From COVID19: burnout, wellbeing, development opportunities. Plan for attrition.
		Wider determinants (n=10)	Social determinants of health	Water, sanitation, education, employment
			Health inequalities	From COVID19: disproportionate health outcomes for ethnic minorities and lower socioeconomic groups
			Physical and critical infrastructure	Road and access, buildings and public services
			Environmental considerations	One Health movement, planetary health
	Prepare	Planning & anticipation (n=12)	Disaster or crisis protocols and policies	Sendai Framework for Disaster Risk Reduction, FEMA emergency management cycle
			Strengthen community and long-term care	From COVID19: lack of workers in care settings after initial surge
			Modelling disasters	
			Strengthen interorganisational networks	Transport and supply chains, voluntary sectors, faith-based organisations
			Training and maintenance of skills	Disaster and major incident training, simulation and drills.
		Core capacities (n=11)	Beds, staff, equipment, medicines	Existing resources and emergency stockpiles
			Plans for utilising surge capacities	Equipment, spaces, alternative facilities
Intra-event	Absorb	Continuity of services (n=19)	Maintain essential services	Maternity, paediatrics, emergency medicine, intensive care
			Planning and involving spontaneous volunteers	ISO 22319:2017
			Mass-casualty triage protocols	Royal College of Emergency Medicine guidance
			Ensure access to healthcare services	Consider hard-to-reach populations
		Surge capacity (n=16)	Mobilising core and surge capacities	
			Activating policies and processes for major incidents	
			Donation standards and distribution	
	Adapt	Adaptation of services (n=17)	Temporary reduction in elective services	From COVID19: Crisis standards of care
			Temporary incident command and communication system	
			Adopting new technologies	From COVID19: telemedicine and videoconferencing
		Flexible culture and teamwork (n=7)	Collective sensemaking and fast variables in healthcare	From COVID19: improvisations and workarounds characterised resilient adaptation.
			Trade-offs, workarounds and improvisation	WAI/WAD. Actuality:Potentiality ratios
Post-event	Recover	Plans for post-event recovery (n=8)	Acute psychological interventions for staff	
			Staff for increased long-term care	Predicted late surge in community, complex and long-term care
			Strategies for community recovery	Recovery for Development Framework. National Consortium for Societal Resilience
	Review	Commitment to quality improvement and change methodology (n=16)	Quality improvement method or framework	Evaluation reports, vulnerability analysis, risk assessments. Safety 1 & 2
			Evaluation and data collection systems	
		Address slow variables (n=8)	Organisational or system adaptation based on lessons learnt	
Trans-event	Collaboration, Coordination, Cooperation	Leadership & management (n=20, 12 respectively)	Effective leadership and trust	Open style leadership during crisis
			Crisis leadership training	
		Multiple stakeholders (n= 3)	Links with surveillance organisations	Meteorological institutes, public services, public health authorities
			Cross-sector collaboration	Patient groups, manufacturers, volunteer and faith-based sector, private, non-profit
			Diverse approaches to support creativiity and innovation	Kruk's Resilience Index, Principles of Socio-ecological resilience, Empirical studies of Uganda's public corporations
	Connectedness	Resources and redundancies (n=22)	Supply and stock management processes	
			Plans for pooling resources	From COVID19: Capacity transfers in intensive care
		Networks and communities (n=16)	Integrated care	
			Dialogue with community leaders & volunteers	
			Regional and international links and diplomatic ties	International emergency response for earthquakes and natural disasters
	Communication	Governance (n=5)	Ensure multiple feedback loops	
			Civil registration and statistics systems	
		Health information systems (n=7)	Multi-level surveillance strategies	Public health functions, routine data collection and dissemination, data sharing and integration, early warning systems
		Workforce (n=17)	Feedback & communication mechanisms across hierarchical levels	Staff forums, incident reporting
			Staff recognition	
		Media (n=3)	Accuracy of information disssemination	From COVID19: false news and vaccine hesitancy
			Population trust in health system	

Supplementary file

Healthcare resilience - A meta-narrative systematic review and synthesis of reviews

For peer review only

List of supplementary materials

Appendix 1: Additional details of methodology
Appendix 2: Definition word cloud and word occurrence table
Appendix 3: Data extraction table from reviews
Appendix 4: Healthcare resilience framework summary

Appendix 1: Additional methodological details

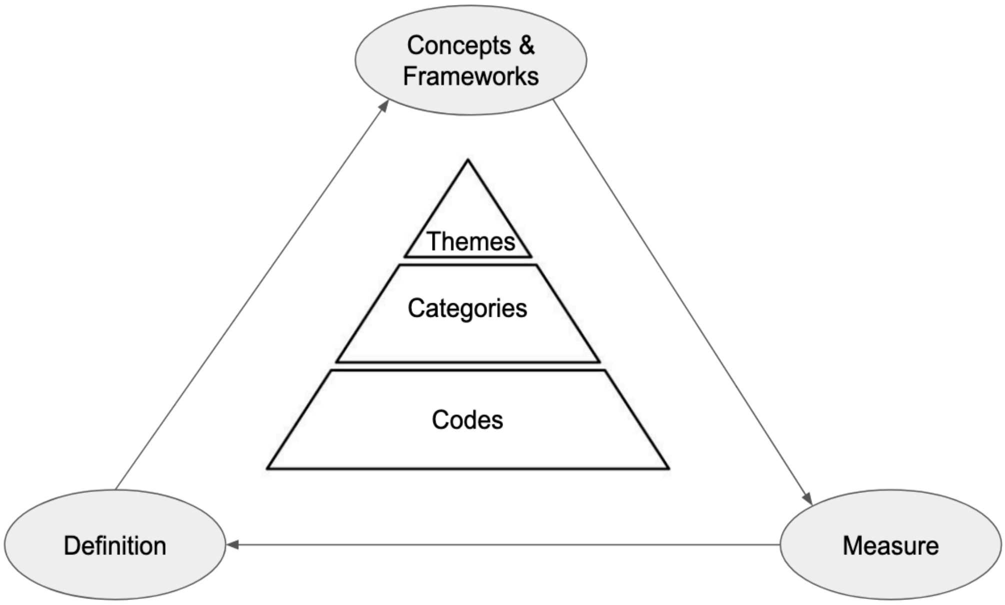

Appendix 1.1: Theoretical framework describing three tiers of analysis within three foci.

In choosing our approach, we referred to Xiao et al.'s typology of reviews [1]. We sought to describe and extend the current understanding of healthcare resilience. Since resilience has been studied within a range of academic disciplines, across multiple sectors and a numerous hierarchies and levels, a meta-narrative was the most appropriate form of review to perform. This aims to consolidate the various research traditions involved in the study of a broad topic, thereby forming an overarching synthesis of the landscape of said topic [2]. A related method is the realist review, which seeks to distil circumstantial conditions conferring success of various interventions [3]. Traditional systematic reviews or meta-analyses were unsuitable methodologies for this study due to several reasons. In particular, studies into resilience include multiple methodologies, both quantitative and qualitative, over a span of disciplines which include evolving terminology and nomenclature. The homogeneity required for a traditional systematic review or quantitative measures which form the basis of a meta-analysis do not feature in resilience studies [4 5].

The key principles of meta-narrative reviews are pragmatism, pluralism, historicity, contestation, reflexivity and peer review [6]. This review of reviews provides a multidisciplinary and multi-level view of healthcare resilience. It maps current understanding and presents various conceptualisations and frameworks (pluralism). This will thus be useful for a multitude of actors including healthcare leaders, policymakers and academics (pragmatism).

Examining the various research traditions which converge in this topic, it will both chart its evolution and explore various perspectives (historicity). Differences in definitions, measures and conceptualisations will be highlighted and discussed (contestation). The protocol is updated according to the findings, and future agendas are proposed in line with emergent data and insights (reflexivity). Finally, independent reviewers are involved throughout the process, while results are shared with a broader research team focusing on whole-of-society resilience (peer review).

Tool or method	Reason for unsuitability
**Cochrane protocol** [7]	Requires established quantitative metrics
**Cochrane qualitative tool** [8]	Only applicable for empirical studies. Unsuitable for mixed or multi-method studies
**Mixed Methods Assessment Tool (MMAT)**[9]	Heavily weighted towards quantitative studies. Many studies in healthcare resilience are qualitative in nature. Sections on randomisation are irrelevant for this topic.
**Critical Appraisal Skills Programme (CASP) checklist** [10]	Deals with systematic reviews mainly. This precludes many of the reviews within healthcare resilience.
**Quality Assessment of Diverse Studies (QuADS)**[11]	Targeted at empirical studies. Many components are difficult to apply to reviews.
**AMSTAR2 tool** [12]	Focused on healthcare interventions, centred around (population, intervention, control group and outcome) PICO components

Appendix 1.2: Table of critical appraisal tool examined and why they were deemed unsuitable for this study.

For peer review only

Appendix 2: Definitions - word cloud and word occurrence table

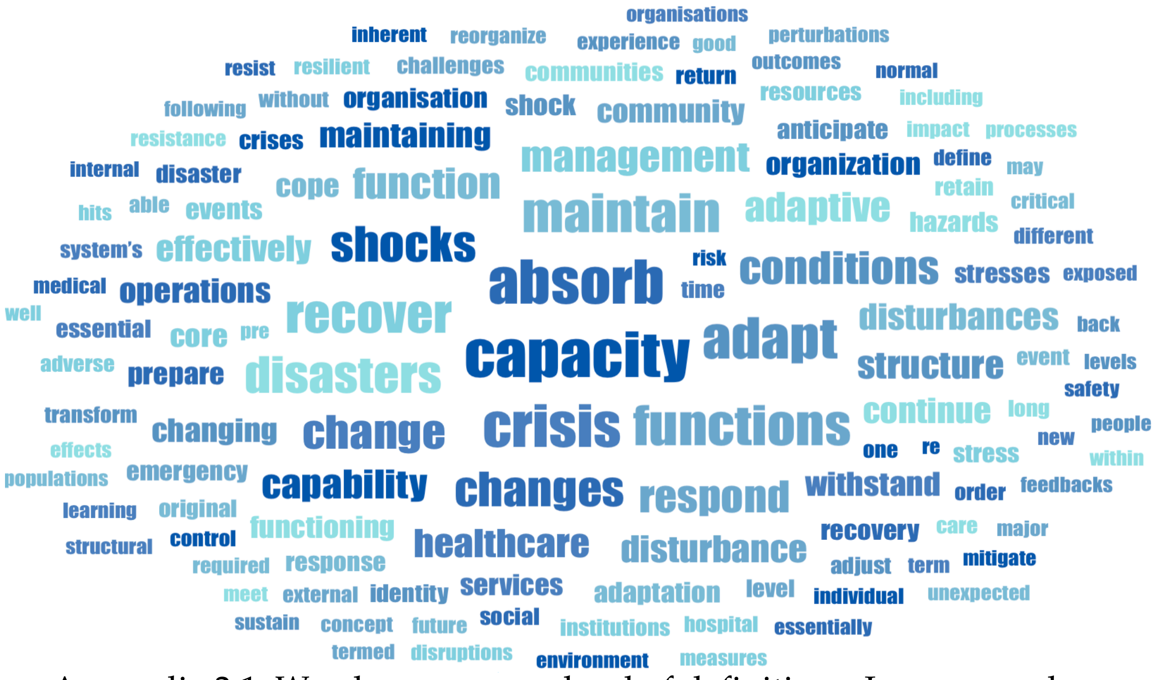

Appendix 2.1: Word occurrence cloud of definitions. Larger words represent increased frequency.

Healthcare Resilience Definition: Word occurrences by timeframe

Pre-event		Intra-event		Post-event		Referent events	
Word/Synonym	Frequency	Word/Synonym	Frequency	Word/Synonym	Frequency	Word/Synonym	Frequency
Prepare	17	Absorb	40	Recovery	42	Crisis	53
Anticipate	10	Adapt	64	Return	15	Shocks/Impacts	41
Expect	5	Capabilities/ Capacities	83	Learning	11	Changes	51
Resource	9	Respond	34	Improvement	9	Disturbance/ Disruption	34
		Maintain	61	Transform	19	Disaster	37
		Essential services	33	Future	7	Events	19
		Essential functions	64			Adversity	12
		Manage	23			Challenges	8
		Mitigate	23			Emergencies	10
		Resist	13			Hazards	11
		Withstand	13				
		Adjust	8				
		Cope	12				

Appendix 2.2: Word occurrence table.

Appendix 3: Data extraction table from reviews

Author, Year	Title of study	Hierarchical level	Research paradigm	Geographic focus & country of 1st author	Study design	Brief summary
**Alami et al., 2021** [13]	How Can Health Systems Better Prepare for the Next Pandemic? Lessons Learned From the Management of COVID-19 in Quebec (Canada)	Macro	Public health	Quebec, Canada	Narrative review and survey	Took a capacity-focused approach to resilience. Concepts include: Insufficient workforce in several settings, need to ensure training, safety and protection, misaligned policies, shortage of equipment and drugs, ethical dilemmas and utilitarianism, importance of high-level communication, managing media communications
**Augustynowicz et al., 2022** [14]	Resilient Health and the Healthcare System. A Few Introductory Remarks in Times of the COVID-19 Pandemic	Macro	Patient safety, theoretical	International, Poland	Narrative review	Describes definition and scope issues. Covers definitions from several paradigms. Lists some of the evolution of thinking in resilience. Includes urban and community resilience which is outside the scope of our review
**Ayanore et al., 2019** [15]	Towards Resilient Health Systems in Sub-Saharan Africa: A Systematic Review of the English Language Literature on Health Workforce, Surveillance, and Health Governance Issues for Health Systems Strengthening	Macro	Global health - HSS	Sub-Saharan Africa, Ghana	Literature review	Assessed 3 out of 6 elements of WHO building blocks of health in Sub-Saharan Africa. Presents lessons learnt from studies focused mainly on infectious diseases outbreaks and epidemics. Key concepts include leadership and physical resource, including equipment, funding and workforce. Some studies highlight the importance of inter-organisational relationships and effective communication
**Banwell et al., 2018** [16]	Towards improved linkage of disaster risk reduction and climate change adaptation in health: A review	Meta	Disaster and Climate change adaptation	International, Australia	Literature review	Increasing links in literature between DRR and climate change adaptation. Refers to SFDRR. Highlights the need for cross-sectoral work between DRR, climate science and healthcare to build future resilience for health systems
**Barasa et al., 2018** [17]	What is resilience and how can it be nurtured? A systematic review of empirical literature on organizational resilience	Meso/Macro	Global health	International, UK	Literature review	Key concepts: Material resources, preparedness & planning, information management, collateral pathways and redundancies, governance processes, leadership, organisational culture, human capital, social networks and collaboration
**Berg et al., 2018** [18]	Methodological strategies in resilient health care studies: An integrative review	Meso	Resilience engineering	International, Norway	Integrative review	All studies included used qualitative methodology to study resilience. Concepts: individual strategies, sense-making, decision-making, performance variability and expertise

For peer review only - http://bmjopen.bmj.com/site/about/guidelines.xhtml

**Biddle et al., 2020** [19]	Health system resilience: A literature review of empirical research	Macro	Global health	International, Germany	Narrative review	Refers to the balance between an outcome-oriented understanding vs a process-oriented understanding of resilience, taken from Blanchet et al. [20]. Public health studies categorised into three groups: (1) Quantitative studies on service delivery, (2) Qualitative studies focusing on HRH, (3) Broader perspectives: building blocks, etc.<br><br>Macro-level Resilience informed by two discourses: disaster management and healthcare quality (patient safety) and resilience engineering.<br>DRR perspectives have shifted after hurricane Catrina in USA toward community resilience
**Blumenstock et al., 2014** [21]	Measuring Preparedness: The National Health Security Preparedness Index	Macro	Public health	USA, USA	Policy document	Presents the National Health Security Preparedness Index:<br>Health surveillance - surveillance and epidemiology investigation, environmental and biological monitoring, laboratory testing<br>Community planning and engagement - cross-sector collaboration, at-risk populations, spontaneous volunteers, social capital and cohesions<br>Incident and information management - multi-agency coordination, emergency public information and warning<br>Surge management - emergency medical services, acute & primary care, mental healthcare<br>Countermeasure management - medical supply chain and distribution, countermeasure utilisation and effectiveness"
**Brand & Jax, 2007** [22]	Focusing the Meaning(s) of Resilience: Resilience as a Descriptive Concept and a Boundary Object	Meso/Macro	Theoretical	International, Germany	Literature review	Focuses on definitions of resilience. 3 categories (descriptive, hybrid and normative). 10 classes and 10 definitions: Original ecological, extended ecological, systematic-heuristic, operational, sociological, ecological-economic, ecosystem-services-related, social-ecological system, metaphoric, sustainability-related.<br><br>Stresses the importance of determining resilience "of what, to what" to operationalise
**Burke et al., 2021** [23]	Building health system resilience through policy development in response to COVID-19 in Ireland: From shock to reform	Macro	Public health - COVID	Ireland, Ireland	Literature review of health policies	States that the Irish government made several changes to increase healthcare access during the COVID19 pandemic. Argues that existing policies/frameworks should not be abandoned in a pandemic, but authorities should focus on those aspects within the used frameworks that contribute to strengthening and dealing with the acute shock. Views resilience as a transformative process in response to crisis
**Carrington et al., 2021** [24]	The impact of disasters on emergency department resources: review against the Sendai framework for disaster risk reduction 2015–2030	Meso	Global health - Disaster	International, Australia	Integrative review	Uses SFDRR to assess emergency departments. Identified points for improvement based on SFDRR framework

For peer review only

**Cartwright et al., 2017** [25]	The changing health priorities of earthquake response and implications for preparedness: a scoping review	Macro	Global health - DRR	LMICs, UK	Scoping review	Mapped the needs in physical health, mental health and health care to disaster cycle. Categorised according to time frame after the event.<br>Preparedness: disconnect between planning and policy, importance of leadership. Acute phase: women, children and elderly disproportionately affected, trauma to extremities, spinal cord injuries, wound debridement, perinatal issues, lack of specialists, physical equipment and lack of workforce, transport systems, coordination difficulties, lack of integration of information systems and international staff. Response: general health needs, respiratory, ocular, GI and dermatology problems, wound infections, late presentations, cold supply chain failure, increased suicides, staff mental health, importance of leadership and coordination, utilitarianism, surveillance. Chronic phase: rehabilitation, unwanted pregnancies, worsening burden of chronic diseases, PTSD and mental health, psychological support for victims and staff<br><br>Mapped lessons onto disaster management cycle. Plan: Policy, address inequalities, surveillance and long-term monitoring, current service gaps, maintenance of essential services, surge capacity, record systems, international staff, logistics, changing health priorities. Organise and equip: medication, equipment and blood, supply points, transportation, donations. Train: disaster mangement, leadership. Exercise: regular exercises to validate plans and arrangements. Evaluate and improve
**Challen et al., 2012** [26]	Where is the evidence for emergency planning: A scoping review	Macro	Emergency management	HICs, UK	Scoping review	Makes use of FEMA emergency cycle. Key aspects: Mitigation, hazard analysis, capability assessment, emergency planning, capability maintenance, emergency response, recovery, development plans, communications/mass media, informatics and intelligence, other organisational issues
**Curtis et al., 2017** [27]	Impact of extreme weather events and climate change for health and social care systems	Macro	Climate	UK, UK	Literature review	Identified need to improve hospital infrastructure, wider social infrastructure and collective responsibility towards resilience approach, cross-sectoral work and integration of planning and preparedness.<br><br>Concepts categorised into three domains: Physical (built infrastructure: health facilities, utilities), Institutional (policies and practices, human resources, professional practice), Social (community groups and informal networks)
**Fallah-Aliabadi et al., 2020** [28]	Towards developing a model for the evaluation of hospital disaster resilience: A systematic review	Meso	Disaster management	International, Iran	Literature review	Assessed several frameworks for disaster resilience: Hospital Safety Index (HSI), Dynamic approach to seismic resilience of hospitals, Measuring framework of the hospital resilience, Indicators for assessing hospital disaster preparedness in Japan.<br><br>Concepts categorised into domains (with 27 sub-domains): Constructive (stability, design, architectural, transportation and transition system<br>Infrastructural: power, water and sewage, communication and IT, Heating, ventilation and air conditioning (HVAC), fuel, medical gas, equipment and furniture, hazardous material, fire system), Administrative (disaster plan, risk assessment and reduction, response, command, coordination, evacuation, need assessment, logistic and supplies, safety committee, continuity of services, volunteers, finance, recover, training)

**Fridell et al., 2020** [29]	Health System Resilience: What Are We Talking About? A Scoping Review Mapping Characteristics and Keywords	Macro	Global health	International, Sweden	Scoping review + expert consultation	Narrative presentation of definitions of resilience.<br><br>Key features of resilience were mapped onto WHO building blocks of health framework: Leadership and governance (ethics, system capacity, operationalisation, engagement, institutional design), information (surveillance, policy making, evaluation), health workforce (diversity and flexibility, limited resources, incentive, community involvement), financing (resource allocation, funding mechanisms, costing), medicines (sustainability), service delivery (response, prevention)
**Grimm et al., 2021** [30]	Enhancing the Understanding of Resilience in Health Systems of Low- and Middle-Income Countries: A Qualitative Evidence Synthesis	Macro	Global health	LMIC, Switzerland	Literature review + interviews	Maps Kruk's Resilience Index [31] onto WHO's building blocks of health [32]. Refined concepts from Resilience Index: Realigned relationships (micro-, meso- and macro-level). Public/private partnerships), foresight and motivation (Investment into slow variables, motivation: political will and individual commitment), change management (integrated change management systems and processes help to confer resilience), emergency preparedness
**Haldane et al., 2020** [33]	National primary care responses to COVID-19: a rapid review of the literature	Macro	Global health	International, Canada	Rapid review	Key lessons from COVID19: Surge capacity should be strengthened. Need to ensure essential services continuity. National guidance needed on biological sampling handling. Most systems increased their telemedicine capabilities
**Hasan et al., 2021** [34]	Integrated health service delivery during COVID-19: A scoping review of published evidence from low-income and lower-middle-income countries	Macro	Global health (UHC)	LMIC, Canada	Scoping review	Assessment of L-LMIC's ability for integrated health service delivery during COVID19 according to WHO building blocks and pandemic continuum.<br><br>Key recommendations: Strengthening of coordination between healthcare bodies at various levels, cross-sectoral collaboration between government, private sector, media, and armed forces. Establishing integrated platforms for testing, health infrastructure to scale-up (redundancies). Empowering communities to engaged in disease prevention and containment. Expansion of digital health technologies. Build resilience by investing in Primary Healthcare and Integrated Health Service Delivery
**Hosseini et al., 2016** [35]	A review of definitions and measures of system resilience	Meso	Engineering	International, USA	Literature review	Presents a narrative of various definitions. Considers differences in organisational, social, economic, and engineering domains. Reliability is often used in engineered systems. There is multidimensionality in some definitions Ongoing issues with definitions include uncertain mechanisms to achieve resilience, the role of recovery, and the need to return to steady-state.<br><br>Presents a summary of how studies measure resilience. Qualitative: conceptual framework, semi-quantitative. Quantitative: generic resilience metrics, structural-based modelling
**Iflaifel et al., 2020** [36]	Resilient Health Care: a systematic review of conceptualisations, study methods and factors that develop resilience	Meso/Macro	Patient safety	International, UK	Literature review	Studies used a mix of qualitative and quantitative methods to study resilience.<br><br>Key concepts: Teamwork, in-situ practical experience, exposure to diverse views and perspectives, trade-offs, protocols & checklists, system design, workarounds

For peer review only

**Jesus et al., 2021** [37]	PREparedness, REsponse and SySTemic transformation (PRE-RE-SyST): a model for disability-inclusive pandemic responses and systemic disparities reduction derived from a scoping review and thematic analysis	Macro	Disability studies	International, India	Scoping review	Focused on inclusive approach to pandemic responses to reduce health inequalities. Key concepts: transform societies' cultural assumptions about disability. Design systems and policies for structural disability-inclusiveness. Prepare inter-sectoral, disability-inclusive pandemic preparedness. Reduce inequalities. Rapid and compassionate responses in residential and long-term care settings. Provide accessibility
**Koeva & Rohova, 2020** [38]	Health System Resilience: Concept Development	Micro/Meso/ Macro	Theoretical	International, Bulgaria	Literature review	Narrative presentation of quotations of definitions. Dictionaries distinguish between physical objects and innate human capacity. Engineering: resist shocks, absorb and hold elastic strain before permanent distortion or failure. Psychology: copes and maintains function in face of adversity, trauma, tragedy or stress. Resilience as an outcome, a process, or even contains both dynamic and static properties (within community resilience).<br><br>Resilience is undergoing evolution from outcome-oriented to process-oriented. There is a dynamically to the scope, breadth and depth of definitions. Conceptualisations are increasingly broad and complex. Existence of dynamism, uncertainty, non-linearity and unpredictability.
**Kruk et al., 2017** [31]	Building resilient health systems: a proposal for a resilience index	Macro	Global health	LMICs, USA	Case studies/ review + framework proposal	Defines resilience as "the capacity of health actors, institutions, and populations to prepare for and effectively respond to crises; maintain core functions when a crisis hits; and, informed by lessons learnt during the crisis, reorganise if conditions require it."<br><br>Key domains: aware (capacity, risks and communication), diverse (meets range of needs, adequate financing), self-regulating (maintain core services and leverage collaborations), integrated (coordination and community engagement), adaptive (distributive decision-making, flexible spending and evaluation for improvement)
**Lapão et al., 2015** [39]	Ebola impact on African health systems entails a quest for more international and local resilience: the case of African Portuguese speaking countries	Meso/Macro	Global health	East Africa, Portugal	Narrative review and case study	Demonstrated fragility of African health systems during Ebola epidemic: increased demand, inequalities and poor access, infrastructure impact, decreased in standards of care of other areas, themes for learning and improvement, critical services need to continue
**Li et al., 2020** [40]	Analysing Healthcare Facility Resilience: Scientometric Review and Knowledge Map	Meso	Global health (UHC)	China, China	Literature review	Measures tend to be indicator based (set of dimensions and indicators), function based (quantitative evaluation of functionality of healthcare facilities), or structure based (modelling of system structures (elements and relationships) and testing failure).<br><br>Concepts: preparedness (risk assessment, strengthen capacities, HRH, HIS, leadership and governance, coordination), monitoring (surveillance, warning systems, monitoring systems), absorb (physical capacities, past experience, flexible approaches, HIS, communication, recovery and learning (safety1 & 2 approaches, organisational quality improvement strategies)

For peer review only

**Luke et al., 2021** [41]	Safer hospital infrastructure assessments for socio-natural disaster - A scoping review	Meso	Disaster management	International, Australia	Scoping review	Uses WHO hospital safety index. Key domains: structural (Previous damage and repair, building integrity, condition of building materials, interaction with other buildings/elements, structural redundancies, architectural design, structure design appropriate to location), non-structural (architectural safety, infrastructure protection, access, and physical security, medical equipment and supplies), critical systems (electrical, telecommunications, water, fire protection, waste management, fuel supplies, medical gases, heating/ventilation)
**Meyer et al., 2020** [42]	A checklist to improve health system resilience to infectious disease outbreaks and natural hazards	Meso/Macro	Global health , Disaster	International, USA	Scoping review + Interview + Checklist workshop	Proposes checklist as a measurement tool or set of indicators for resilience. Items: maintenance of essential health system services and functions, critical infrastructure and transportation, financing, access, communication, collaboration, coordination and partnerships, leadership and command, surge capacity, risk communications, workforce, infection control
**Nuzzo et al., 2019** [43]	What makes health systems resilient against infectious disease outbreaks and natural hazards? Results from a scoping review	Macro	Global health	International, USA	Scoping review	Resilience attributes: maintenance of essential functions, access to healthcare, maintaining critical infrastructure and transport, crisis financing, leadership and command, collaboration, coordination and partnerships, communication, flexible plans and management structures, legal preparations, surge capacity, crisis standards of care, human resources for health, infection prevention and control, quality improvement, post-event recovery (psychological health of staff, revision of plans, rebuilding social cohesion and trust).<br><br>Themes are consistent with the Resilience Index. Additional findings: developing crisis standards of care, plan for post-event recovery, commitment to quality improvement to ensure lessons learned.
**Oppenheim et al., 2019** [44]	Assessing global preparedness for the next pandemic: development and application of an Epidemic Preparedness Index	Macro	Global health	International, USA	Framework proposal and evaluation	Epidemic Preparedness Index: public health infrastructure (surveillance, immunisation, medical workforce, hospital capacity, coordination), physical infrastructure (water and sanitation, roads, phones, internet, logistics), institutional capacity (political stability, corruption, bureaucratic effectiveness, armed conflict, homicide, vital registration), economic resources (government revenue generation, per capita income, gross domestic product, health spending, resource dependency), public health communications (public education, risk communication)
**Penaloza et al., 2020** [45]	A resilience engineering perspective of safety performance measurement systems: A systematic literature review	Meso	Resilience engineering	International, Brazil	Literature review	Safety performance measurement systems should support real-time monitoring and feedback of everyday performance variability, with the ability to analyse adverse events and excellent practices. Measures should be dynamic and subject to re-evaluation

For peer review only - http://bmjopen.bmj.com/site/about/guidelines.xhtml

**Pillay & Morel, 2020** [46]	Measuring Resilience Engineering: An Integrative Review and Framework for Bench-Marking Organisational Safety	Meso	Resilience engineering	International, Australia	Integrative review	"Resilience Engineering is a perspective for organisational safety management which enables organisational members to actively anticipate, respond, monitor and learn; by adapting to operate at the boundary of safe operations by narrowing the gap between work as imagined and work as performed; and manifested in an organisation's culture, cognition and behaviours"<br><br>Argues the need to establish boundaries of safe performance, balance trade-offs between production and safety, and dealing with managed vs prescribed safety. Shifts resilience culture towards resilient climate, acknowledging environmental conditions necessary for resilience.<br><br>Key indicators of resilience: learning culture, flexibility, top management commitment, awareness, preparedness, just culture
**Rameshshanker et al., 2021** [47]	Health system resilience to extreme weather events in Asia-Pacific: a scoping review	Macro/Meta	Disaster, climate change	Asia-Pacific, Canada	Literature review	Explored how well health systems were resilient towards adverse weather events. Categorised efforts according to WHO building blocks checklist.<br>Medical workforce was the most frequently mentioned aspect, while health financing was the least.
**Rangachari & Woods, 2020** [48]	Preserving Organizational Resilience, Patient Safety, and Staff Retention during COVID-19 Requires a Holistic Consideration of the Psychological Safety of Healthcare Workers	Micro/Meso	Psychology, Operational research	International, USA	Narrative review	During COVID19, trust and leadership were key aspects to maintain holistic resiliience of staff, which may improve organisational resilience. Advocates for environment of trst, empowerment and safety to enable patient safety concerns to be brought up
**Ravi et al., 2019** [49]	Establishing a theoretical foundation for measuring global health security: A scoping review	Macro	Global health - HSS	International, USA	Scoping review	Focused on health security, centred around performance of critical health security systems, biosafety and biosecurity, and public health preparedness. Underscored importance of real-time health information systems.<br><br>Key concepts: measuring global health security requires analysis of existing prevention, detection, and response capacities, as well as efforts to build core public health, healthcare, and biosecurity capacities. Assessments of national programs and efforts to mitigate a critical subset of priority threats could serve as useful proxies for measuring global health security. There are measurable enabling factors that facilitate global health security-strengthening efforts. Measuring global health security requires consideration of the risk environment from which infectious disease threats might emerge
**Rezaei et al., 2018** [50]	Hospitals preparedness using WHO guideline: A systematic review and meta-analysis	Meso	Emergency management	International, Iran	Systematic review and meta-analysis	Measured compliance to WHO hospital emergency checklist. Domains include command and control, communication, safety and security, triage, surge capacity, continuity of essential services, human resources for health, logistics and supply management, post-disaster recovery

For peer review only

**Son et al., 2020** [51]	Investigating resilience in emergency management: An integrative review of literature	Meso/Macro	Emergency management	International, USA	Integrative review	Presents a table of collated definitions. Stressed the temporal nature in defining resilience. Some focus on pre-event whilst others focus on intra-event. Expected disruption: some focus on the ability to deal with unexpected events, whilst others do not view the distinction. Means to achieve resilience: allocation of roles and resources, HIS or adjusting procedures.<br><br>Dimensions of resilience in emergency management: collective sense-making (improvisation, honest communication, virtual role systems, attitude of wisdom), team decision making (incident command system, training in generic decision-making protocols), harmonising WAI and WAD, interaction and coordination.<br><br>Tools used to support human operators for resilience: geospatial mapping, event history logs, mobile communication systems, decision support tools
**Tessema et al., 2021** [52]	The COVID-19 pandemic and healthcare systems in Africa: a scoping review of preparedness, impact and response	Macro	Global health, COVID	Africa, Australia	Scoping review	Used Population, Concept Context (PCC) framework for defining eligibility<br>Measured preparedness, impact and response of health systems<br>Framed within WHO building blocks.<br><br>Key findings: preparedness (limited resources, knowledge gaps, lack of training, limited surge capacity and testing ability, limited health services), Impact (reduced patient flow, miss appointments for chronic services, re-orientation of services deter essential services provision, not all services affected), impact on workforce (stigma, mental health illness), response (provision of guidelines, telehealth, segregation of patient streams)
**Tin et al., 2010** [53]	An approach to health system strengthening in the Union of Myanmar	Macro	Public health - HSS	Myanmar, Myanmar	Case study/ national review	Prioritised service delivery, programme coordination and human resource management to strengthen Myanmar's health system. Takes a systems thinking approach. Uses Health Systems Strengthening indicators at measures and index to compare several countries.
**Tippong et al., 2021** [54]	A Review of Applications of Operational Research in Healthcare Coordination in Disaster Management	Meso/Macro	Emergency and Disaster management	International. UK	Literature review	Measured coordination effectiveness in healthcare systems. Various staffing patterns have trade-offs: financial cost vs sustainable pattern. Triage and minimisation of beds needed. Coordination between healthcare-network is important for surge management and adaptive capacity.
**Turenne et al., 2019** [55]	Conceptual analysis of health systems resilience: A scoping review	Macro	Theoretical	International, France	Scoping review	Presented a collated table of definitions and word frequency table. Concepts are dependent on contexts (different groups and populations)<br>In the face of changes. Argues that resilience thinking may be multi-sectoral, but health systems resilience is primarily concerned with the functions of the health system

For peer review only

**Turner et al., 2021** [56]	Systematic review of experiences and perceptions of key actors and organisations at multiple levels within health systems internationally in responding to COVID-19	Micro/Meso/ Macro	Global health - COVID	International. Colombia	Narrative review	Individual level: staff needed to adapt to new working conditions, burnout and psychological distress: work intensity, staff shortages, fear of contagion and transmission, positive adaptation: Telehealth, community healthcare workers, creative approaches<br><br>Organisational level: COVID as catalyst for service change, need to provide psychological support for staff, open management style during crisis<br><br>Local health system level: continuous training to support preparedness for future pandemics, the need to look at working conditions, the need to coordinate diverse actors (community and primary health etc), review and update regulations
**Wiig et al., 2020** [57]	Defining the boundaries and operational concepts of resilience in the resilience in healthcare research programme	Meso	Patient safety	International, Norway	Debate and review	Centred around engineering, psychological and ecological perspectives: Resilience of what, to what, through what. Focused around high quality healthcare, centred around set a capacities, based on events which provide challenges, changes or disruptions. Grounded in multi-level adaptation, enhancement and reorganisation, indicating diversity, coordination and collaboration and is open-ended
**Xin & Xu, 2012** [58]	Hospital emergency management research in China: trends and challenges	Macro	Emergency management	China, China	Narrative review	Defines crisis based on disruptions to hospital systems: patient surge, provision of services, reputation, financial trouble, or major incident. Noted a move from normal vs abnormal crisis to all-hazards. Comprehensive management system may be more useful than temporary command and control, or professional response team. Identified current issues with Chinese medical systems based on WHO building blocks
**Zhong et al., 2014** [59]	Development of hospital disaster resilience: conceptual framework and potential measurement	Macro	Global health - Disaster	International, China	Literature review	Presented a collated list of definitions from different disciplines. Most measures focus on structure, but hardly on process and outcome (based on Donebedian categories of quality of care). Propose measures as a framework: Hospital safety and vulnerability - surveillance, risk & safety. Disaster preparedness and resources - Leadership, cooperation and communication, logistics & stockpiles, workforce, training & drills. Continuity of essential services - surge capacity, emergency medicines. Recovery and adaptation - evaluation, adaptation and recovery capability.<br><br>Factors conferring resilient healthcare: Accessibility, Critical services, Leadership, Integration with community

Legend: WHO = World Health Organisation. DRR = Disaster Risk Reduction. USA = United States of America. HRH = Human Resources for Health. L-LMIC = Low and Low-Middle Income Countries. PTSD = Post-Traumatic Stress Disorder. SFDRR = Sendai Framework for Disaster Risk Reduction. HIS = Health Information Systems

For peer review only

Appendix 4: Summary characteristics of selected resilience frameworks

Framework (reference)	Key components or indicators or principles	Hierarchical focus	Paradigm	Pre-event	Intra-event	Post-event
**Resilience Potentials** [60]	Anticipate,<br>Monitor,<br>Respond,<br>Learn	Meso	Engineering			
**Independent EM concepts** [51]	Collective sense-making,<br>Team decision-making,<br>Harmonising WAI/WAD,<br>Interaction and Coordination	Meso	Emergency Management			
**FEMA adapted emergency management cycle** [26]	Mitigation, hazard and capacity assessment<br>Planning<br>Information systems<br>Continuity of services<br>Emergency response<br>Communications<br>Recovery	Meso	Emergency Management			
**WHO Hospital Safety Index (HSI)** [41]	Structural (e.g. building integrity, redundancies, previous damage)<br>Non-structural (e.g. critical systems, medical equipment and supplies)<br>Disaster management system	Meso	Healthcare			
**WHO hospital disaster preparedness** [46 50]	Command and Control<br>Communication<br>Safety and Security<br>Triage<br>Surge capacity<br>Continuity of essential services<br>HRH<br>Logistics and supply management<br>Post-disaster recovery	Meso	Healthcare			
**Hospital disaster resilience model** [28]	3 domains with 27 sub-domains:<br>Constructive (stability, design, architectural, transportation)<br>Infrastructural (power, water/sewage, communication/IT, HVAC, fuel, medical gas, equipment<br>Administrative (disaster plan, command, coordination, needs assessment, logistics and supplies, safety committee, continuity of services, volunteers, finance, recovery, training)	Meso	Healthcare			
**Independent hospital and healthcare resilience** [59]	4 principles:<br>Robustness<br>Redundancy<br>Rapidity<br>Resourcefulness<br>4 cross cutting domains:<br>Vulnerability and safety<br>Recovery and adaptation<br>Continuity of essential services<br>Preparedness and resources	Meso/Macro	Healthcare			
**Independent disaster preparedness cycle** [25]	5 parts of cycle further divided into components, all underpinned by leadership & coordination:<br>Plan<br>Organise & equip<br>Train<br>Exercise<br>Evaluate & improve	Meso/Macro	Disaster Management			

For peer review only

**Concepts conferring resilience** [17]	Material resources<br>Preparedness & planning<br>Information management<br>Collateral pathways and redundancies<br>Governance processes<br>Leadership<br>Organisational culture<br>Human capital<br>Social networks and collaboration	Meso/Macro	Healthcare			
**Primary Care Framework for pandemics (from H1N1)** [33]	Clinical service delivery,<br>Public health functions,<br>Primary care facility operational level<br>Health system level	Meso/Macro	Healthcare			
**NHS Emergency Preparedness, Resilience and Response (EPRR) framework**	Preparedness & anticipation<br>Continuity<br>Subsidiary: decisions at lowest level<br>Communication<br>Cooperation & integration<br>Direction	Macro	Healthcare			
**SFDRR** [61]	Reduce Disaster Mortality<br>Reduce the Number of Affected People<br>Reduce Direct Disaster Economic Loss<br>Reduce Disaster Damage to Critical Infrastructure and Disruption of Basic Services<br>Increase the Number of Countries with National and Sub-National/Local Disaster Risk Reduction Strategies<br>Enhance International Cooperation<br>Increase the Availability of and Access to Multi-Hazard Early Warning Systems	Macro	Disaster Management			
**Global Health Security Index** [62]	Prevent (pathogens)<br>Detect<br>Respond<br>Healthcare (formal)<br>Norms<br>Risk	Macro	Public health			
**Epidemic Preparedness Index** [44]	Public health infrastructure: surveillance, immunisation, medical workforce, hospital capacity, coordination<br>Physical infrastructure: water and sanitation, roads, phones, internet, logistics<br>Institutional capacity: political stability, corruption, bureaucratic effectiveness, armed conflict, homicide, vital registration<br>Economic resources: government revenue generation, per capita income, gross domestic product, health spending, resource dependency<br>Public health communications: public education, risk communication	Macro	Public health			
**European Primary Care Monitoring** [63]	Structure: Governance, economic conditions, workforce<br>Process: access, comprehensiveness, coordination and continuity<br>Outcome: Quality, efficiency, equity	Macro	Healthcare			
**USA Public Health Infrastructure** [64]	Assessment: monitor health, diagnose/investigate<br>Policy development: Inform/educate/empower, community partnerships, develop policies<br>Assurance: Enforce laws, link care, competent workforce, evaluate	Macro	Public health			
**WHO building blocks** [32]	Leadership & governance,<br>Health Information Systems (HIS),<br>Human Resources for Health (HRH),<br>Financing,<br>Service delivery,<br>Essential medicines and technologies	Macro	Healthcare			

For peer review only

**National Health Security Preparedness Index (NHSPI)** [21]	Health surveillance: surveillance and epidemiology investigation, environmental and biological monitoring, laboratory testing<br>Community planning and engagement: cross-sector collaboration, at-risk populations, spontaneous volunteers, social capital and cohesions<br>Incident and information management: multi-agency coordination, emergency public information and warning<br>Surge management: emergency medical services, acute & primary care, mental healthcare<br>Countermeasure management: medical supply chain and distribution, countermeasure utilisation and effectiveness	Macro	Healthcare			
**Integrated Health Service Delivery** [34]	Clinical,<br>Service,<br>Organisational,<br>Functional	Macro	Healthcare			
**Emerging Infectious Diseases Preparedness** [65]	Hardware: Surveillance, infrastructure & medical supplies, workforce, communication mechanisms<br>Software: Governance, trust	Macro	Healthcare			
**Myanmar Health Systems Strengthening Strategy** [53]	Service delivery<br>Programme coordination<br>Human resources	Macro	Healthcare			
**Stockholm Resilience Centre 7 Principles** [66]	Foster complex adaptive systems thinking,<br>Maintain diversity & redundancies,<br>Broaden Participation,<br>Manage connectivity,<br>Manage slow variables and feedback,<br>Encourage learning,<br>Polycentric governance	Macro/Meta	Socioecologic al systems			
**NZ living standards framework** [67]	Financial/physical capital,<br>Human capital,<br>Natural capital,<br>Social capital	Macro/Meta	Community			
**Refined conceptual models of health system resilience** [30 43]	Foundations of resilience added onto the Resilience Index:<br>Realigned relationships,<br>Foresight and motivation,<br>Change management,<br>Emergency preparedness<br>Crisis standards of care<br>Recovery plans<br>Learning and improvement	Micro/Meso/M acro	Healthcare			
**Health Systems Resilience Index** [31]	Aware: capacity, risks and communication<br>Diverse: meets range of needs, adequate financing<br>Self-regulating: maintain core services and leverage collaborations<br>Integrated: coordination and community engagement<br>Adaptive: distributive decision-making, flexible spending and evaluation for improvement	Meso/Macro/ Meta	Healthcare			

Legend: Frameworks are categorised according to predominant hierarchical focus and paradigm. They are colour-coded according to which time domain they cover.

Abbreviations: EM = Emergency Management. WAI/WAD = Work-As-Imagined vs Work-As-Done. FEMA = Federal Emergency Management Agency. WHO = World Health Organisation. HRH = Human Resources for

Health. IT = Information Technology. HVAC = Heating, Ventilation and Air-Conditioning. NHS = UK's National Health Service. SFDRR = Sendai Framework for Disaster Risk Reduction. USA = United States of America. NZ = New Zealand

For peer review only

References

1. Xiao Y, Watson M. Guidance on Conducting a Systematic Literature Review. *Journal of Planning Education and Research* 2019;39(1):93-112. doi: 10.1177/0739456x17723971
2. Greenhalgh T, Robert G, Macfarlane F, et al. Storylines of research in diffusion of innovation: a meta-narrative approach to systematic review. *Soc Sci Med* 2005;61(2):417-30. doi: 10.1016/j.socscimed.2004.12.001 [published Online First: 2005/05/17]
3. Gough D. Meta-narrative and realist reviews: guidance, rules, publication standards and quality appraisal. *BMC Medicine* 2013;11(1):22. doi: 10.1186/1741-7015-11-22
4. Smith V, Devane D, Begley CM, et al. Methodology in conducting a systematic review of systematic reviews of healthcare interventions. *BMC Med Res Methodol* 2011;11(1):15. doi: 10.1186/1471-2288-11-15 [published Online First: 20110203]
5. Becker L, Oxman A. Overviews of reviews. In: Higgins J, Green S, eds. Cochrane handbook for systematic reviews of interventions. Oxford: The Cochrane Collaboration 2010.
6. Wong G, Greenhalgh T, Westhorp G, et al. RAMESES publication standards: meta-narrative reviews. *BMC Medicine* 2013;11(1):20. doi: 10.1186/1741-7015-11-20
7. Higgins JP TJ, Chandler J, Cumpston M, Li T, Page MJ, Welch VA, editor(s). Cochrane Handbook for Systematic Reviews of Interventions Version 6.2 (updated February 2021): Cochrane; 2021 [Available from: training.cochrane.org/handbook accessed Feb 2022.
8. Hannes K. Supplementary Guidance for Inclusion of Qualitative Research in Cochrane Systematic Reviews of Interventions. Version 1 (updated August 2011). In: Noyes J BA, Hannes K, Harden A, Harris J, Lewin S, Lockwood C ed.: Cochrane Collaboration Qualitative Methods Group 2011.
9. HONG QN, PLUYE P, FÀBREGUES S, et al. MIXED METHODS APPRAISAL TOOL (MMAT): McGill University; 2018 [Available from: http://mixedmethodsappraisaltoolpublic.pbworks.com/w/file/fetch/127916259/MMAT_2018_criteria-manual_2018-08-01_ENG.pdf accessed Jan 2022.
10. CASP. Critical Appraisal Skills Programme - Qualitative Checklist 2018 [Available from: https://casp-uk.net/wp-content/uploads/2018/01/CASP-Qualitative-Checklist-2018.pdf accessed Feb 2022.
11. Harrison R, Jones B, Gardner P, et al. Quality assessment with diverse studies (QuADS): an appraisal tool for methodological and reporting quality in systematic reviews of mixed- or multi-method studies. *BMC Health Services Research* 2021;21(1):144. doi: 10.1186/s12913-021-06122-y
12. Shea BJ, Reeves BC, Wells G, et al. AMSTAR 2: a critical appraisal tool for systematic reviews that include randomised or non-randomised studies of healthcare interventions, or both. *BMJ* 2017;358:j4008. doi: 10.1136/bmj.j4008

For peer review only

13. Alami H, Lehoux P, Fleet R, et al. How Can Health Systems Better Prepare for the Next Pandemic? Lessons Learned From the Management of COVID-19 in Quebec (Canada). *Frontiers in public health* 2021;9:671833. doi: 10.3389/fpubh.2021.671833
14. Augustynowicz A, Opolski J, Waszkiewicz M. Resilient Health and the Healthcare System. A Few Introductory Remarks in Times of the COVID-19 Pandemic. *Int J Environ Res Public Health* 2022;19(6) doi: 10.3390/ijerph19063603
15. Ayanore MA, Amuna N, Aviisah M, et al. Towards Resilient Health Systems in Sub-Saharan Africa: A Systematic Review of the English Language Literature on Health Workforce, Surveillance, and Health Governance Issues for Health Systems Strengthening. *Annals of Global Health* 2019;85(1) doi: 10.5334/aogh.2514
16. Banwell N, Rutherford S, Mackey B, et al. Towards improved linkage of disaster risk reduction and climate change adaptation in health: A review. *Int J Environ Res Public Health* 2018;15(4) doi: 10.3390/ijerph15040793
17. Barasa E, Mbau R, Gilson L. What is resilience and how can it be nurtured? A systematic review of empirical literature on organizational resilience. *International Journal of Health Policy and Management* 2018;7(6):491-503. doi: 10.15171/ijhpm.2018.06
18. Berg SH, Akerjordet K, Ekstedt M, et al. Methodological strategies in resilient health care studies: An integrative review. *Safety Science* 2018;110:300-12. doi: 10.1016/j.ssci.2018.08.025
19. Biddle L, Wahedi K, Bozorgmehr K. Health system resilience: A literature review of empirical research. *Health Policy and Planning* 2020;35(8):1084-109. doi: 10.1093/heapol/czaa032
20. Blanchet K, Nam SL, Ramalingam B, et al. Governance and Capacity to Manage Resilience of Health Systems: Towards a New Conceptual Framework. *International Journal of Health Policy and Management* 2017;6(8):431-35. doi: 10.15171/ijhpm.2017.36
21. Blumenstock J, Bakker G, Jarris PE. Measuring Preparedness: The National Health Security Preparedness Index. *Journal of Public Health Management and Practice* 2014;20(3):361-63. doi: 10.1097/phh.0000000000000073
22. Brand FS, Jax K. Focusing the Meaning(s) of Resilience: Resilience as a Descriptive Concept and a Boundary Object. *Ecology and Society* 2007;12(1)
23. Burke S, Parker S, Fleming P, et al. Building health system resilience through policy development in response to COVID-19 in Ireland: From shock to reform. *Lancet Reg Health Eur* 2021;9:100223. doi: 10.1016/j.lanepe.2021.100223 [published Online First: 20211007]
24. Carrington MA, Ranse J, Hammad K. The impact of disasters on emergency department resources: review against the Sendai framework for disaster risk reduction 2015–2030. *Australas Emerg Care* 2021;24(1):55-60. doi: 10.1016/j.auec.2020.09.003
25. Cartwright C, Hall M, Lee ACK. The changing health priorities of earthquake response and implications for preparedness: a scoping review. *Public Health* 2017;150:60-70. doi: 10.1016/j.puhe.2017.04.024

For peer review only

26. Challen K, Lee AC, Booth A, et al. Where is the evidence for emergency planning: A scoping review. *BMC Public Health* 2012;12(1) doi: 10.1186/1471-2458-12-542
27. Curtis S, Fair A, Wistow J, et al. Impact of extreme weather events and climate change for health and social care systems. *Environmental Health: A Global Access Science Source* 2017;16 doi: 10.1186/s12940-017-0324-3
28. Fallah-Aliabadi S, Ostadtaghizadeh A, Ardalan A, et al. Towards developing a model for the evaluation of hospital disaster resilience: A systematic review. *BMC Health Services Research* 2020;20(1) doi: 10.1186/s12913-020-4915-2
29. Fridell M, Edwin S, Schreeb JV, et al. Health system resilience: what are we talking about? A scoping review mapping characteristics and keywords. *International Journal of Health Policy and Management* 2020;9(1):6-16. doi: 10.15171/ijhpm.2019.71
30. Grimm PY, Oliver S, Merten S, et al. Enhancing the Understanding of Resilience in Health Systems of Low- and Middle-Income Countries: A Qualitative Evidence Synthesis. *International Journal of Health Policy and Management* 2021 doi: 10.34172/ijhpm.2020.261
31. Kruk ME, Ling EJ, Bitton A, et al. Building resilient health systems: a proposal for a resilience index. *BMJ* 2017;357:j2323. doi: 10.1136/bmj.j2323
32. WHO. Key components of a well functioning health system 2010 [Available from: https://www.who.int/healthsystems/EN_HSSkeycomponents.pdf accessed Nov 2019.
33. Haldane V, Zhang Z, Abbas RF, et al. National primary care responses to COVID-19: a rapid review of the literature. *BMJ Open* 2020;10(12):e041622. doi: 10.1136/bmjopen-2020-041622 [published Online First: 20201208]
34. Hasan MZ, Neill R, Das P, et al. Integrated health service delivery during COVID-19: A scoping review of published evidence from low-income and lower-middle-income countries. *BMJ Global Health* 2021;6(6) doi: 10.1136/bmjgh-2021-005667
35. Hosseini S, Barker K, Ramirez-Marquez JE. A review of definitions and measures of system resilience. *Reliability Engineering & System Safety* 2016;145:47-61. doi: https://doi.org/10.1016/j.ress.2015.08.006
36. Iflaifel M, Lim RH, Ryan K, et al. Resilient Health Care: a systematic review of conceptualisations, study methods and factors that develop resilience. *BMC Health Serv Res* 2020;20(1):324. doi: 10.1186/s12913-020-05208-3 [published Online First: 2020/04/19]
37. Jesus TS, Kamalakannan S, Bhattacharjya S, et al. PREparedness, REsponse and SySTemic transformation (PRE-RE-SyST): a model for disability-inclusive pandemic responses and systemic disparities reduction derived from a scoping review and thematic analysis. *Int J Equity Health* 2021;20(1):204. doi: 10.1186/s12939-021-01526-y [published Online First: 20210914]
38. Koeva S, Rohova M. Health system resilience: concept development. *Journal of Imab* 2020;26(3):3251-58. doi: 10.5272/jimab.2020263.3251

For peer review only

39. Lapão LV, Silva A, Pereira N, et al. Ebola impact on African health systems entails a quest for more international and local resilience: the case of African Portuguese speaking countries. *Pan Afr Med J* 2015;22 Suppl 1(Suppl 1):15. doi: 10.11694/pamj.supp.2015.22.1.6653 [published Online First: 20151011]
40. Li X, Krumholz HM, Yip W, et al. Quality of primary health care in China: challenges and recommendations. *The Lancet* 2020;395(10239):1802-12. doi: 10.1016/S0140-6736(20)30122-7
41. Luke J, Franklin R, Aitken P, et al. Safer hospital infrastructure assessments for socio-natural disaster - A scoping review. *Prehosp Disaster Med* 2021;36(5):627-35. doi: 10.1017/S1049023X21000650
42. Meyer D, Bishai D, Ravi SJ, et al. A checklist to improve health system resilience to infectious disease outbreaks and natural hazards. *BMJ Glob Health* 2020;5(8) doi: 10.1136/bmjgh-2020-002429
43. Nuzzo JB, Meyer D, Snyder M, et al. What makes health systems resilient against infectious disease outbreaks and natural hazards? Results from a scoping review. *BMC Public Health* 2019;19(1) doi: 10.1186/s12889-019-7707-z
44. Oppenheim B, Gallivan M, Madhav NK, et al. Assessing global preparedness for the next pandemic: development and application of an Epidemic Preparedness Index. *BMJ Global Health* 2019;4(1):e001157. doi: 10.1136/bmjgh-2018-001157
45. Penaloza GA, Saurin TA, Formoso CT, et al. A resilience engineering perspective of safety performance measurement systems: A systematic literature review. *Safety Science* 2020;130 doi: 10.1016/j.ssci.2020.104864
46. Pillay M, Morel G. Measuring Resilience Engineering: An Integrative Review and Framework for Bench-Marking Organisational Safety. *Safety* 2020;6(3) doi: 10.3390/safety6030037
47. Rameshshanker V, Wyngaarden S, Lau LL, et al. Health system resilience to extreme weather events in Asia-Pacific: a scoping review. *Clim Dev* 2021;13(10):944-58. doi: 10.1080/17565529.2020.1870425
48. Rangachari P, J LW. Preserving Organizational Resilience, Patient Safety, and Staff Retention during COVID-19 Requires a Holistic Consideration of the Psychological Safety of Healthcare Workers. *Int J Environ Res Public Health* 2020;17(12) doi: 10.3390/ijerph17124267 [published Online First: 2020/06/19]
49. Ravi SJ, Meyer D, Cameron E, et al. Establishing a theoretical foundation for measuring global health security: A scoping review. *BMC Public Health* 2019;19(1) doi: 10.1186/s12889-019-7216-0
50. Rezaei F, Maracy MR, Yarmohammadian MH, et al. Hospitals preparedness using WHO guideline: A systematic review and meta-analysis. *Hong Kong Journal of Emergency Medicine* 2018;25(4):211-22. doi: 10.1177/1024907918760123
51. Son C, Sasangohar F, Neville T, et al. Investigating resilience in emergency management: An integrative review of literature. *Appl Ergon* 2020;87 doi: 10.1016/j.apergo.2020.103114
52. Tessema GA, Kinfu Y, Dachew BA, et al. The COVID-19 pandemic and healthcare systems in Africa: a scoping review of preparedness, impact

For peer review only

and response. *Bmj Global Health* 2021;6(12) doi: 10.1136/bmjgh-2021-007179
53. Tin N, Lwin S, Kyaing NN, et al. An approach to health system strengthening in the Union of Myanmar. *Health Policy* 2010;95(2-3):95-102. doi: 10.1016/j.healthpol.2009.11.013
54. Tippong D, Petrovic S, Akbari V. A Review of Applications of Operational Research in Healthcare Coordination in Disaster Management. *Eur J Oper Res* 2021 doi: 10.1016/j.ejor.2021.10.048 [published Online First: 20211028]
55. Turenne CP, Gautier L, Degroote S, et al. Conceptual analysis of health systems resilience: A scoping review. *Soc Sci Med* 2019;232:168-80. doi: 10.1016/j.socscimed.2019.04.020 [published Online First: 20190430]
56. Turner S, Botero-Tovar N, Herrera MA, et al. Systematic review of experiences and perceptions of key actors and organisations at multiple levels within health systems internationally in responding to COVID-19. *Implementation Science* 2021;16(1) doi: 10.1186/s13012-021-01114-2
57. Wiig S, Aase K, Billett S, et al. Defining the boundaries and operational concepts of resilience in the resilience in healthcare research program. *BMC Health Services Research* 2020;20(1) doi: 10.1186/s12913-020-05224-3
58. Xin YT, Xu KY. Hospital emergency management research in China: trends and challenges. *Emerg Med J* 2012;29(5):353-7. doi: 10.1136/emermed-2011-200512 [published Online First: 20111103]
59. Zhong S, Clark M, Hou X-Y, et al. Development of hospital disaster resilience: conceptual framework and potential measurement. *Emergency Medicine Journal* 2014;31(11):930-U8901. doi: 10.1136/emermed-2012-202282
60. How Resilient Is Your Organisation? An Introduction to the Resilience Analysis Grid (RAG). Sustainable Transformation: Building a Resilient Organization; 2010; Toronto, Canada.
61. Calkins J. Moving forward after Sendai: How countries want to use science, evidence and technology for disaster risk reduction. *PLoS Currents* 2015;7(DISASTERS) doi: 10.1371/currents.dis.22247d6293d4109d09794890bcda1878
62. Bell JA, Nuzzo JB. Global Health Security Index: Advancing Collective Action and Accountability Amid Global Crisis: NTI/John Hopkins center for health security; 2021 [Available from: www.GHSIndex.org accessed Mar 2022.
63. Kringos DS, Boerma WGW, Bourgueil Y, et al. The european primary care monitor: structure, process and outcome indicators. *BMC Fam Pract* 2010;11(1):81. doi: 10.1186/1471-2296-11-81
64. Benjamin GC. Ensuring health equity during the COVID-19 pandemic: The role of public health infrastructure. *Revista Panamericana de Salud Publica/Pan American Journal of Public Health* 2020;44 doi: 10.26633/RPSP.2020.70
65. Palagyi A, Marais BJ, Abimbola S, et al. Health system preparedness for emerging infectious diseases: A synthesis of the literature. *Glob Public Health* 2019;14(12):1847-68. doi: 10.1080/17441692.2019.1614645 [published Online First: 20190514]

For peer review only

66. Biggs R, Schlüter M, Schoon ML. Principles for Building Resilience: Cambridge University Press 2015.
67. Campbell-Macdonald G. Living Standards Framework: New Zealand Treasury; 2018 [Available from: https://www.wgtn.ac.nz/__data/assets/pdf_file/0007/1780954/Grace-Campbell-Macdonald-Living-Standards-Framework.pdf accessed Jan 2022.

For peer review only

PRISMA 2020 Checklist

Section and Topic	Item #	Checklist item	Location where item is reported
**TITLE**			
Title	1	Identify the report as a systematic review.	P1, 4
**ABSTRACT**			
Abstract	2	See the PRISMA 2020 for Abstracts checklist.	P1
**INTRODUCTION**			
Rationale	3	Describe the rationale for the review in the context of existing knowledge.	P4
Objectives	4	Provide an explicit statement of the objective(s) or question(s) the review addresses.	P5
**METHODS**			
Eligibility criteria	5	Specify the inclusion and exclusion criteria for the review and how studies were grouped for the syntheses.	P5
Information sources	6	Specify all databases, registers, websites, organisations, reference lists and other sources searched or consulted to identify studies. Specify the date when each source was last searched or consulted.	P5
Search strategy	7	Present the full search strategies for all databases, registers and websites, including any filters and limits used.	P5
Selection process	8	Specify the methods used to decide whether a study met the inclusion criteria of the review, including how many reviewers screened each record and each report retrieved, whether they worked independently, and if applicable, details of automation tools used in the process.	P6
Data collection process	9	Specify the methods used to collect data from reports, including how many reviewers collected data from each report, whether they worked independently, any processes for obtaining or confirming data from study investigators, and if applicable, details of automation tools used in the process.	P6
Data items	10a	List and define all outcomes for which data were sought. Specify whether all results that were compatible with each outcome domain in each study were sought (e.g. for all measures, time points, analyses), and if not, the methods used to decide which results to collect.	P6, Appendices p5-13
	10b	List and define all other variables for which data were sought (e.g. participant and intervention characteristics, funding sources). Describe any assumptions made about any missing or unclear information.	P6
Study risk of bias assessment	11	Specify the methods used to assess risk of bias in the included studies, including details of the tool(s) used, how many reviewers assessed each study and whether they worked independently, and if applicable, details of automation tools used in the process.	P6
Effect measures	12	Specify for each outcome the effect measure(s) (e.g. risk ratio, mean difference) used in the synthesis or presentation of results.	P6
Synthesis methods	13a	Describe the processes used to decide which studies were eligible for each synthesis (e.g. tabulating the study intervention characteristics and comparing against the planned groups for each synthesis (item #5)).	P6
	13b	Describe any methods required to prepare the data for presentation or synthesis, such as handling of missing summary statistics, or data conversions.	P6
	13c	Describe any methods used to tabulate or visually display results of individual studies and syntheses.	P6 – 12 Appendices p5-13
	13d	Describe any methods used to synthesize results and provide a rationale for the choice(s). If meta-analysis was performed, describe the model(s), method(s) to identify the presence and extent of statistical heterogeneity, and software package(s) used.	P6
	13e	Describe any methods used to explore possible causes of heterogeneity among study results (e.g. subgroup analysis, meta-regression).	N/a
	13f	Describe any sensitivity analyses conducted to assess robustness of the synthesized results.	N/a
Reporting bias	14	Describe any methods used to assess risk of bias due to missing results in a synthesis (arising from reporting biases).	P6

For peer review only

PRISMA 2020 Checklist

Section and Topic	Item #	Checklist item	Location where item is reported
assessment			
Certainty assessment	15	Describe any methods used to assess certainty (or confidence) in the body of evidence for an outcome.	P6, Appendices
**RESULTS**			
Study selection	16a	Describe the results of the search and selection process, from the number of records identified in the search to the number of studies included in the review, ideally using a flow diagram.	P7-8
	16b	Cite studies that might appear to meet the inclusion criteria, but which were excluded, and explain why they were excluded.	P7
Study characteristics	17	Cite each included study and present its characteristics.	P7-8, Appendices p5-13
Risk of bias in studies	18	Present assessments of risk of bias for each included study.	Appendices p5-13
Results of individual studies	19	For all outcomes, present, for each study: (a) summary statistics for each group (where appropriate) and (b) an effect estimate and its precision (e.g. confidence/credible interval), ideally using structured tables or plots.	Appendices p5-13
Results of syntheses	20a	For each synthesis, briefly summarise the characteristics and risk of bias among contributing studies.	P7, 12-13 Appendices p5-13
	20b	Present results of all statistical syntheses conducted. If meta-analysis was done, present for each the summary estimate and its precision (e.g. confidence/credible interval) and measures of statistical heterogeneity. If comparing groups, describe the direction of the effect.	N/a
	20c	Present results of all investigations of possible causes of heterogeneity among study results.	P7, 12-13 Appendices p5-13
	20d	Present results of all sensitivity analyses conducted to assess the robustness of the synthesized results.	N/a
Reporting biases	21	Present assessments of risk of bias due to missing results (arising from reporting biases) for each synthesis assessed.	P7, 12-13 Appendices p5-13
Certainty of evidence	22	Present assessments of certainty (or confidence) in the body of evidence for each outcome assessed.	N/a
**DISCUSSION**			
Discussion	23a	Provide a general interpretation of the results in the context of other evidence.	P7-14,
	23b	Discuss any limitations of the evidence included in the review.	P14
	23c	Discuss any limitations of the review processes used.	P14
	23d	Discuss implications of the results for practice, policy, and future research.	P12-14,
**OTHER INFORMATION**			
Registration and protocol	24a	Provide registration information for the review, including register name and registration number, or state that the review was not registered.	P5
	24b	Indicate where the review protocol can be accessed, or state that a protocol was not prepared.	P5

For peer review only

PRISMA 2020 Checklist

Section and Topic	Item #	Checklist item	Location where item is reported
	24c	Describe and explain any amendments to information provided at registration or in the protocol.	P6
Support	25	Describe sources of financial or non-financial support for the review, and the role of the funders or sponsors in the review.	P16
Competing interests	26	Declare any competing interests of review authors.	P16
Availability of data, code and other materials	27	Report which of the following are publicly available and where they can be found: template data collection forms; data extracted from included studies; data used for all analyses; analytic code; any other materials used in the review.	Appendices

From: Page MJ, McKenzie JE, Bossuyt PM, Boutron I, Hoffmann TC, Mulrow CD, et al. The PRISMA 2020 statement: an updated guideline for reporting systematic reviews. BMJ 2021;372:n71. doi: 10.1136/bmj.n71

For more information, visit: http://www.prisma-statement.org/

For peer review only

BMJ Open

Healthcare resilience - A meta-narrative systematic review and synthesis of reviews

Journal:	*BMJ Open*
Manuscript ID	bmjopen-2023-072136.R1
Article Type:	Original research
Date Submitted by the Author:	17-Jul-2023
Complete List of Authors:	Tan, Mark; The University of Manchester,<br>Prager, Gabrielle; Johns Hopkins University<br>McClelland, Andrew; The University of Manchester, Alliance Manchester Business School<br>Dark, Paul; National Institute for Health and Care Research, Clinical Research Network; The University of Manchester, Humanitarian and Conflict Response Institute
**Primary Subject Heading**:	Health services research
Secondary Subject Heading:	Health services research, Qualitative research, Communication, Global health, Health policy
Keywords:	Systematic Review, HEALTH SERVICES ADMINISTRATION & MANAGEMENT, Hospitals, QUALITATIVE RESEARCH, PUBLIC HEALTH

SCHOLARONE™
Manuscripts

I, the Submitting Author has the right to grant and does grant on behalf of all authors of the Work (as defined in the below author licence), an exclusive licence and/or a non-exclusive licence for contributions from authors who are: i) UK Crown employees; ii) where BMJ has agreed a CC-BY licence shall apply, and/or iii) in accordance with the terms applicable for US Federal Government officers or employees acting as part of their official duties; on a worldwide, perpetual, irrevocable, royalty-free basis to BMJ Publishing Group Ltd ("BMJ") its licensees and where the relevant Journal is co-owned by BMJ to the co-owners of the Journal, to publish the Work in this journal and any other BMJ products and to exploit all rights, as set out in our licence.

The Submitting Author accepts and understands that any supply made under these terms is made by BMJ to the Submitting Author unless you are acting as an employee on behalf of your employer or a postgraduate student of an affiliated institution which is paying any applicable article publishing charge ("APC") for Open Access articles. Where the Submitting Author wishes to make the Work available on an Open Access basis (and intends to pay the relevant APC), the terms of reuse of such Open Access shall be governed by a Creative Commons licence – details of these licences and which Creative Commons licence will apply to this Work are set out in our licence referred to above.

Other than as permitted in any relevant BMJ Author's Self Archiving Policies, I confirm this Work has not been accepted for publication elsewhere, is not being considered for publication elsewhere and does not duplicate material already published. I confirm all authors consent to publication of this Work and authorise the granting of this licence.

For peer review only

Healthcare resilience - A meta-narrative systematic review and synthesis of reviews

For peer review only

Abstract

Objectives

The COVID19 pandemic has tested global healthcare resilience. Many countries previously considered "resilient" have performed poorly. Available organisational and system frameworks tend to be context-dependent and focus heavily on physical capacities. This study aims to explore and synthesise evidence about healthcare resilience and present a unified framework for future resilience-building.

Design

Systematic review and synthesis of reviews using a meta-narrative approach.

Setting

Healthcare organisations and systems

Primary and secondary outcome measures

Definitions, concepts, and measures of healthcare resilience. We utilised thematic analysis across included reviews to summarise evidence on healthcare resilience.

Results

The main paradigms within healthcare resilience include global health, disaster risk reduction, emergency management, patient safety and public health. Definitions of healthcare resilience recognise various hierarchical levels: individual (micro), facility or organisation (meso), health system (macro), and planetary or international (meta). There has been a shift from a focus on mainly disasters and crises, to an "all-hazards" approach to resilience. Attempts to measure resilience have met with limited success. We analysed key concepts to build a framework for healthcare resilience containing pre-, intra-, post- and trans-event domains. Alongside, we synthesise a definition which dovetails with our framework.

For peer review only - http://bmjopen.bmj.com/site/about/guidelines.xhtml

Conclusion

Resilience increasingly takes an all-hazards approach and a process-oriented perspective. There is increasing recognition of the relational aspects of resilience. Few frameworks incorporate these, and they are difficult to capture within measurement systems. We need to understand how resilience works across hierarchical levels, and how competing priorities may affect overall resilience. Understanding these will underpin interdisciplinary, cross-sectoral and multi-level approaches to healthcare resilience for the future.

Strengths and limitations of this study

- This is the first systematic review of reviews done on healthcare resilience, at organisational and system levels.
- A meta-narrative approach is utilised, which is particularly suitable for complex topics spanning across disciplines and hierarchical levels.
- Our analysis and synthesis allow for an inter-disciplinary, cross-sectoral and multi-level framework for healthcare resilience.
- Individual (or micro-level) resilience is not included in this review.
- A review of reviews provides a broad overview but does not encompass all details or knowledge about the topic.

For peer review only - http://bmjopen.bmj.com/site/about/guidelines.xhtml

Main Manuscript Title

Healthcare resilience - A meta-narrative systematic review and synthesis of reviews

1. Background

The COVID19 pandemic has tested the resilience of healthcare across the globe. Previously thriving healthcare systems have struggled with multiple aspects; from hospital capacity [1] to infection control measures [2], healthcare worker burnout [3] to degrees of community trust [4], and from international research cooperation [5] to vaccine tribalism and hesitancy [6]. Our previous understanding of healthcare resilience requires re-evaluation.

To practically respond to the whole-of-society challenges in light of COVID19, we need evidence that effectively integrates knowledge across these disciplines. This need has been highlighted by several reviews [7 8]. The meta-narrative approach offers such a method. It has been used to track several disciplines converging within a complex field, and is useful for making sense of such complex, heterogenous, and disparate data across disciplines and sectors [9 10]. It thus forms an interdisciplinary and cross-sectoral approach to healthcare resilience.

Recognition that resilience at individual, organisational and system levels affect each other has resulted in some early multi-level empirical research protocols [11 12]. Yet, many studies tend to focus on single hierarchal levels[13-15], within set paradigms [16 17]. For example, literature on psychological resilience tends to focus on acute interventions during or after crisis [18]. These seldom consider wider determinants at organisational or system level which affect individual resilience [3]. Similarly, some disaster risk reduction (DRR) frameworks have put little emphasis on recovery after crisis [19], which has been highlighted as an important factor to build resilience after COVID19 [20]. Therefore, in addition to interdisciplinary and cross-sectoral evidence, multi-level knowledge must also be consolidated.

Given the sheer volume of empirical literature available on healthcare resilience, it was impractical to review beyond a single hierarchical level. Instead, reviews of reviews are particularly useful for gaining a broad overview of complex topics and systems with many dependencies. For example, it has helped to better understand policy and practice priorities in climate sciences [21], and to unpick COVID19 vaccine hesitancy [22]. Both are highly complex and current topics involving multiple sectors, disciplines, and hierarchical levels.

We therefore undertook a systematic review of reviews using a meta-narrative approach to present an overview and critical look at healthcare resilience. It highlights lessons learnt from the COVID19 pandemic and synthesises evidence towards an

For peer review only - http://bmjopen.bmj.com/site/about/guidelines.xhtml

overarching framework. The research aims to:

1. Explore the definitions, measures, and concepts of healthcare resilience.
2. Synthesise evidence from the exploration and analysis towards a broad overview of the topic.
3. Present an interdisciplinary, cross-sectoral, and multi-level framework from analysed data.

2. Methods

Theoretical approach

We undertook a systematic literature review of reviews using a meta-narrative approach [23 24]. The review was registered with PROSPERO (CRD42022314729)[25]. We utilised RAMESES guidelines for reporting meta-narrative reviews [24]. PRISMA guidelines were followed[26].

The principles of the meta-narrative review are pragmatism, pluralism, historicity, contestation, reflexivity, and peer-review. This approach was adopted for several reasons. Firstly, the field has been informed by many research traditions. Secondly, there exists historical agendas which have shifted the focus of resilience at various hierarchical levels (historicity). Thirdly, there are sometimes contrasting findings about resilience (pluralism and contestation). Fourthly, an interdisciplinary overview may be useful for policymakers, practitioners, and academics (pragmatism). Such an approach helps to extract seemingly heterogenous data (peer review). Finally, the final search strategy was informed by some initial searches and snowball sampling, reflecting a reflexive protocol which was adapted according to findings.

Search strategy

A systematic keyword search was conducted in Scopus (Elsevier), Web of Science (Clarivate), PubMed (NIH), and Global Index Medicus (WHO)(Figure 1). We included alternative spellings and synonyms. Inclusion criteria included reviews related to healthcare organisations and systems. We included records from 2008 to November 2022. The search was performed in January 2022. A repeat search was performed in November 2022, which yielded five further records, four of which did not meet selection criteria. 2008 corresponded to the global financial crisis, which resulted in an increase in resilience literature [27]. Resilience is dynamic [28]. Concepts from earlier events may not possess the same relevance as more recent articles. This is especially the case since COVID19. Reviews which were non-healthcare, focused on individual, psychological or community resilience alone, or concerned with specific diseases outside of epidemic contexts were excluded. We also excluded those focused on infection control measures only [29]. There were many reviews on individual

For peer review only - http://bmjopen.bmj.com/site/about/guidelines.xhtml

psychological resilience, particularly acute interventions to mental health crises. While we recognised the importance of individuals that make up organisations and systems, we wanted to focus on how resilience is understood at other hierarchical levels, so we excluded these reviews.

Study selection

Records were managed with Endnote 20 (Clarivate analytics). Two reviewers independently selected papers. This was done for several reasons. Firstly, there was wide heterogeneity of studies and papers. Secondly, some reviews refer to cross-sectoral interactions but didn't deal with healthcare. Thirdly, the quality of reviews varied significantly. As a result, having independent reviewers (peer-review) minimises personal bias and ensures there is agreement about selection criteria [30 31]. Screening was done using a modified two-stage process. Title screening was insufficient as a stage on its own due to lack of information about the actual review, or only using "resilience" as a tangential keyword. Both reviewers thus performed a title/abstract screening independently, followed by abstract/full-text screening and discussion around inclusion and exclusion criteria. Additional reviewers were agreed *a priori* should disagreements between the two primary reviewers occur. The final list of included papers was supplemented by snowball sampling and hand searching. This was done by searching through relevant references from selected reviews and discussions with other interdisciplinary team members. Because of the volume of results (Figure 2), it was impractical to focus on empirical studies, or we would have had to confine the search to a single hierarchical level. Therefore, we included reviews only. This may reduce details and resolution, but it allows a summary of knowledge across several reviews and disciplines [32].

Quality appraisal

Quality appraisal of articles was conducted using the Joanna Briggs Institute (JBI) checklist for systematic reviews [33]. While there are no specific quality appraisal tools for systematic reviews of reviews, the JBI checklist formed a best fit compared to several other tools (Appendix 1).

Data extraction and analysis

Using an inductive approach, three-tiered coding of the text was conducted around the three foci of definition, measures, and concepts [34](Appendix 1). We used Atlas.ti qualitative software and data extraction tables. We examined prominent definitions and how they have been informed by different research disciplines. We then collated definitions from the reviews and analysed the most frequently occurring words (Appendix 2). These results were categorised and synthesised with the concepts of resilience to produce a definition which acknowledges past research, links current knowledge, and spans hierarchical levels. Concepts were coded from reviews as well as from the frameworks presented in papers (Appendix 3).

For peer review only - http://bmjopen.bmj.com/site/about/guidelines.xhtml

Patient and public involvement

There was no patient or public involvement in this research.

3. Results

Four databases yielded 8845 records, of which 1070 were reviews. First-stage screening yielded 319 records, of which 68 were duplicates. 251 records were re-screened and discussed. The remaining 52 records underwent quality appraisal. This resulted in 33 records from the search which were analysed. 5 reviews were excluded for low quality. They lacked details about search strategy, inclusion criteria, data analyses, or indeed clear research questions. Other reviews were not relevant according to inclusion and exclusion criteria. Snowball sampling yielded 12 records for analysis. These include reviews which discussed resilience frameworks. The PRISMA diagram summarises the selection process (Figure 2).

3.1 Definitions of healthcare resilience

Most reviews took their definition of healthcare resilience from previous papers. Three reviews contained lists of definitions from other studies [17 35 36]. Another three reviews presented narrative collections and analyses of definitions [15 37 38].

Resilience tends to be defined based on paradigms within which it operates [36-38]. For example, within the engineering paradigm it is concerned with the ability of a structure or material to return to its original state [39]. However, within business and ecological systems, it includes the ability for an organisation or system to capitalise or improve after a shock [38 40 41]. A prominent definition of healthcare resilience is from Hollnagel as “the ability of the health care system (a clinic, a ward, a hospital, a county) to adjust its functioning prior to, during, or following events, and thereby sustain required operations under both expected and unexpected conditions” [42 43]. While this definition continues to be refined, it provides a frame to highlight several aspects which emerged from our word occurrence analysis too.

First is the recognition of different hierarchical levels of the healthcare system [43 44](Figure 3). There is consensus of these as micro-level (referring to individual), meso-level (hospital or organisation), macro-level (region or country)[43 44]. Several nuances continue to evade neat classification. For example, the team level does not neatly fit into micro- or meso-levels [45], and a community has been considered both meso- and macro-levels [37 38]. There is also emergence of a meta-level of healthcare resilience. This level focuses mainly on the climate crisis as a determinant of health and how it threatens healthcare resilience and exacerbates inequalities [8 46-48]. This transcends national boundaries and so must be considered on a meta-level.

For peer review only - http://bmjopen.bmj.com/site/about/guidelines.xhtml

Second, resilience spans pre-event, intra-event, and post-event. This appreciates contributions from different paradigms. DRR studies are mostly concerned with pre-event aspects, such as preparing for shocks thereby minimising disturbance to a system [13 49-53]. Emergency Management (EM) studies examine aspects of how organisations adapt during a crisis (intra-event), and how they may learn from it [17 54]. Some definitions do not include recovery, while others emphasise that rapidity of recovery back to steady state is a key feature of resilience [16 37 51 55].

Third, both expected and unexpected events are considered. DRR and EM paradigms are concerned with disasters and unexpected crises, which suggest that resilience is only manifest when a sufficiently large shock is applied to the system [35]. Patient safety paradigms seek to minimise unintended harm to patients, whether in crisis situations, or within normal operational stressors in healthcare [7 36 38 56]. Recently, even DRR and EM reviews have adopted such an "all-hazards" approach to resilience [1 48].

Hollnagel's definition of healthcare resilience, though widely used, does not specifically include recovery or review [42]. Instead, he refers to these concepts as "learn" within his related Resilience Analysis Grid (RAG) [57]. The resilience potentials in RAG are (1) anticipate, (2) respond, (3) monitor, and (4) learn. Many other definitions map well onto Hollnagel's definition and his four potentials. There are benefits in linking up definitions with "potentials" or concepts of resilience. It provides convergence in a complex field, which helps to develop a consistent approach across hierarchical levels. This may promote understanding between policymakers and healthcare practitioners (including leaders and managers). In turn, it may lead to more unified approaches across discipline and hierarchical boundaries.

We performed word occurrence analysis from the included reviews, followed by tiered coding into themes, with iterative refinement based on the analysis of concepts of resilience (Appendix 2). The result is a synthesised definition which complements previous work, spans across disciplines, and dovetails with concepts explored in the next section. Our definition of healthcare resilience is *the ability of healthcare workers, organisations or systems to (a) prepare for and prevent, (b) absorb and adapt to maintain structure and essential functions, (c) recover and review from crises, shocks or stressors* (Figure 4).

3.2 Concepts of healthcare resilience

Concepts may refer to practices or features which promote or hinder resilience, or indicators of resilience. Groups of concepts are often collated in frameworks, or set as best fit into established frameworks (e.g. WHO building blocks of health).

For peer review only - http://bmjopen.bmj.com/site/about/guidelines.xhtml

Frameworks are useful to systematically consider various aspects of healthcare resilience [48 58]. We highlight key concepts at macro- and meso-levels, paying attention to lessons learnt from the COVID19 pandemic. We then present our framework of concepts learnt from this review (Figure 5), which dovetail with our definition (Figure 4).

Physical capacities

Within the DRR and EM paradigms, strengthening of existing capacities feature most prominently. This includes adequate workforce [59], equipment [44 60], financial resources [4], and services [4 59] to ensure maintenance of essential functions during a crisis. From the COVID19 pandemic, appropriate distribution of physical capacities during various stages of a crisis are equally important. For example, changing shift patterns of emergency staff for increased demand intra-event, but redistributing staff to address longer-term care needs post-event [61]. This adopts a longitudinal approach to resilience past the acute crisis and helps to address predicted shortages. As such, it may then prevent mere shifting of crisis from one organisation to another.

Multi-level approaches

Several reviews have highlighted the multi-level approaches required to build resilience [12 43 62-65]. A small number of empirical studies are currently underway to understand how this works in practice [11 66]. Multi-level approaches recognise that while previous boundaries help understand specific details about how individual hierarchical levels pursue resilience, they are insufficient in representing the interconnections between levels. As an example, while improvisation and coping were seen as resilient concepts at the micro-level, healthcare workers' ability to continue working was underpinned by the availability of resources and protective equipment provided by the organisation and their leaders (meso-level), as well as recovery efforts introduced after the crisis at meso- and macro-level [4 44 60]. Further work is needed to better understand the interplay and dynamics between the hierarchical levels.

Recovery and Review

The most appropriate method of quality improvement to ensure learning after a crisis depends on context. It is more important for organisations to demonstrate consistency and commitment to change methodology, rather than any specific tool or method [67]. This is termed "review" in our framework. Within the patient safety paradigm, two prominent and complementary concepts are safety1 and safety2 approaches. The safety1 approach attempts to get to the root cause of adverse events in healthcare, while the safety2 approach recognises that success occurs more frequently than adverse events and therefore lessons can be learnt from such practices too [42].

Recovery is mentioned in DRR/EM literature [16 55 68], but less so in patient safety literature. This could be contextual. In DRR/EM, crises tend to be larger in scale and

For peer review only - http://bmjopen.bmj.com/site/about/guidelines.xhtml

affect more people, organisations, and sectors outside of healthcare. Patient safety tends to focus on teams within clinical emergency scenarios or normal operational circumstances. DRR/EM paradigms therefore require a more concerted recovery effort. Rapidity of recovery has been cited as a feature of resilience, but there is no benchmark. Extent of recovery to previous function has been explored, but this does not appreciate the occasional change in function of a system following a large crisis [14 37]. Several reviews have emphasised the importance of having plans for recovery within resilience frameworks, but do not elaborate on details [44 59 60]. Therefore, there is still much to understand about recovery, and some recent frameworks summarise key lessons learnt from COVID19 [20].

Relational aspects

The COVID19 pandemic has highlighted the importance of relational aspects of resilience. A key aspect is effective leadership [1 13 17 37 48 50 51 54 56 61-63 65 68-73]. Collaborative and authentic styles which were visible and approachable seemed most favoured during crises[1 2 4 12 17 55 56 68 71 72 74 75]. Command-and-control structures have been cited, but these refer more to the flow of information rather than organisation of authority. Consequently, more recent DRR reviews advocate for decisions to be pushed out towards the periphery, enabling more rapid adaptation to change [16 51 54 59].

Networks beyond formal healthcare facilities bolsters resilience through the availability of physical capacities (e.g. financing, staff, equipment and volunteers) [1 13 16 37 61 62 68 73 74 76]. It also facilitates adaptation and planning post-event. It also facilitates post-event concepts. For example, COVID19 clinical and vaccine trials depended on international collaboration between many disciplines and industries [5]. Recognising the importance of this concept, the recent UK Health and Care Act 2022 specifically focuses on inter-organisational relationships towards the provision of integrated care. Further research will therefore be required to better understand how these relationships work in practice [77-79].

An interdisciplinary, cross-sectoral, and multi-level framework for healthcare resilience

These concepts, along with others which emerged from analysis of the reviews, have been compiled into an interdisciplinary, cross-sectoral, and multi-level framework (Figure 4 & 5, Appendix 3). It spans meso- and macro-levels, which few existing frameworks do. It balances the historical focus on physical capacities with relational aspects of resilience. It incorporates lessons learnt from the COVID19 pandemic. The framework may be used as tool to systematically consider various aspects of organisational and systems resilience.

The framework is divided into timeframes (pre-, intra-, post-, and trans-event). Within timeframes are themes (prepare and prevent etc). Themes encompass several

For peer review only - http://bmjopen.bmj.com/site/about/guidelines.xhtml

concepts, and finally, these concepts are further broken down into components (Figure 4 & 5). In Figure 4, the size of faint circles indicates the frequency of concepts extracted from the reviews, which are also represented as n-numbers in Figure 5.

3.3 Measures of resilience

Due to the considerable global interest in healthcare resilience, there have been attempts to develop measures or indicators for resilience. The process of pursuing resilience requires ways to determine progression. Attempts to measure resilience therefore allow comparisons and identification of potential areas for improvement. However, none of the current measures have been validated, and some widely used indices have been shown to be inaccurate during the COVID19 pandemic.

Two examples at the Macro-level are the Global Health Security Index (GHSI) and Epidemic Preparedness Index (EPI). They both focus on infectious diseases outbreaks and contain several domains including public health and healthcare infrastructure (e.g. surveillance capabilities, medical workforce), economic resources, and risk assessment and communication strategies [2 80]. As such, they are focused on the pre-event domain of crises. However, many High-Income Countries (HICs) that scored well on these indices performed very poorly during the COVID19 pandemic [81]. Several reasons have been cited, including lack of consideration of existing health inequalities, globalisation, societal connectedness, and a bias towards physical capacities (e.g. number of hospitals or beds)[81]. Of note, increased globalisation suggests that ”disease control may be only as effective as practices within the poorest performing countries” [81]. This supports the need for a meta-level of resilience where global health inequalities affect the healthcare resilience of other nations.

Some reviews attempt to evaluate a country’s resilience according to one or more domains of the WHO building blocks of health framework [14 59 65 69 82]. However, the framework was designed as a systematic approach to the funding decisions within healthcare, rather than to evaluate resilience [36].

Meso-level measures tend to stem from DRR/EM paradigms, focused on healthcare facilities. Several reviews have collated evidence from empirical papers centred around earthquakes and natural disasters. Quantitative measures include physical capacities (e.g. number of beds, staff, equipment), physical infrastructure (e.g. structural integrity of buildings)[1], and actuality:potentiality ratios (e.g. adherence to operating protocols)[37 83]. Qualitative indicators include checklists containing several components important during disaster situations (e.g. pre-emptive protocols and procedures, command structures, compliance to structural regulations)[16 68]. These qualitative components address the intra-event domain of the resilience framework. By providing measurement tools and checklists, pre-emptive strengthening of intra-event components can help to reduce the impact of crises when they do occur.

For peer review only - http://bmjopen.bmj.com/site/about/guidelines.xhtml

In the patient safety paradigm, the Checklist for Assessing Institutional Resilience (CAIR) contains broad domains to help hospitals consider several concepts of resilience [84]. It was not designed as a set of measures but may be considered a tool for systematic thinking in patient safety. Similarly, while some have suggested that the work-as-imagined vs work-as-done (WAI/WAD) ratio (or the more general actuality:potentiality ratio) may be used as a way to improve patient safety (by minimising performance variability)[17], others highlighted that this approach may work for some simple processes, but is unlikely to result in favourable patient outcomes in unexpected or complex scenarios [43]. For example, adherence to sepsis guidelines (WAI) is audited and associated with overall better outcomes [85], but depends on accurate diagnosis, which is itself difficult. Sepsis is frequently misdiagnosed (WAD), leading to wrong treatments, antimicrobial resistance, and poor outcomes [86 87]. Therefore, WAI/WAD is not an adequate approach to measure resilience. Instead, WAI/WAD should be used as means to understand the balance struck within systems, and a concept with which to frame observations about adaptations in healthcare. This reframes resilience from an outcome-oriented approach to a process-oriented one.

Some multi-level assessments of the resilience of critical infrastructures exist. The Tiered Approach by Linkov et al. highlights three tiers of assessments focusing on relationships between components of a system [88]. A key feature of this is the use of multiple tools for assessment at different levels and for different goals. For example, the Functional Resonance Analysis Method (FRAM)[89] is useful for mapping relationships between components, while indices may help to align proposed changes with the overarching goals of the system. Checklists may be useful during a crisis. Modelling can help to predict consequences, audit progress, and address slow variables. As such, our framework (Figure 4 & 5, Appendix 3) and the related appendix 4 provide examples of resilience frameworks mapped onto the time domain of crises. There are contemporary studies which seek to better understand multi-level influences specific to healthcare resilience [78 79].

There seems to be limited focus on the measurement of post-event and trans-event domains. While quality improvement models tend to be embedded within healthcare organisations and systems, assessment of speed of recovery and robustness of interorganisational relationships rarely feature in assessment tools. Similarly, assessment of culture and leadership is challenging [90].

4. Discussion

We have employed a relatively new review methodology well-suited for complex topics informed by various research traditions. There is increasing acknowledgement

For peer review only - http://bmjopen.bmj.com/site/about/guidelines.xhtml

of the need for interdisciplinary, cross-sectoral, and multi-level approaches to healthcare resilience. Yet, its history has been one of multiple research traditions operating within specific paradigms. Global health paradigms dominate the macro-level, while disaster risk reduction and patient safety frame the meso-level.

Definitions of healthcare resilience are increasingly broad. They have evolved from highly contextualised situations towards appreciation of different hierarchical levels and from an outcome-oriented understanding towards one which is more process-oriented. With this evolution, it has become more difficult to characterise failure of resilience. Even though healthcare systems and organisations are affected by a range of stressors, they are inherently resilient as essential societal services. Recognising the interplay between hierarchical levels is vital for building resilience. For example, the Institute for Healthcare Improvement framework for improving joy in work considers meso- and macro-level aspects (e.g. participative management and an environment of psychological safety), that affect micro-level resilience, and thus, explicitly links previously distinct hierarchical levels [91]. Such inter-level frameworks rarely feature at the meso- and macro-levels, despite healthcare workers being arguably the most important resources of healthcare systems. Several recent study protocols have been published to better understand multi-level influences in resilience, particularly in the patient safety paradigm[11 66].

Put another way, resilience concepts at macro-level have tended to focus on physical capacities prior to COVID19. Yet, there is increasing recognition of the importance of relational aspects of resilience, particularly across macro- and meso-level boundaries. Some examples of these within the COVID19 pandemic include the adoption of more open leadership at meso- and macro-levels [60], interorganisational relationships and their realignment [4 62], cultivating an environment of trust [44 61], and open communication channels at micro-, meso- and macro-levels [92]. Open leadership styles gained more trust and enabled better understanding of WAI/WAD [44]. Alongside, the open channels of communication between healthcare workers, managers and in turn national bodies helped with rapid adaptation and identification of acute needs, from equipment and supply issues to psychological support services [60].

While a range of tools and indicators have been developed for physical capacities and infrastructure, it is still not known how best to assess the effectiveness of relational aspects of resilience. In particular, understanding the nature of interorganisational relationships seems to stand out as a research priority.

Our framework for healthcare resilience fulfils several needs in this field. First, it brings together knowledge and observations gained from a range of disciplines, across hierarchical levels, and integrates it with lessons learnt from the COVID19 pandemic. Second, it emphasises both physical and relational aspects of resilience, the latter of

For peer review only - http://bmjopen.bmj.com/site/about/guidelines.xhtml

which has received less attention in the past. Finally, it dovetails with our definition and achieves some convergence in a highly heterogenous field.

There are several strengths to this study. Firstly, a review of reviews provides a broad overview of the topic across several geopolitical contexts. Reviews included countries and hospitals with different health financing models, health inequalities, geographies, and range of services (Appendix 3). There is consolidation of knowledge across a wide range of disciplines and paradigms, enabling wider appreciation of the complexities of the topic and the many ways it has been approached. Secondly, a meta-narrative approach helps to explore historical trends and ongoing debates within a complex field. In addition to the consolidation of knowledge in the previous point, this approach also acknowledges the differences between paradigms and broadens the understanding of resilience (Figure 3, Appendix 4). Thirdly, we have taken a multi-level approach at meso- and macro-levels, which has hitherto been rare. These three points provide a map for healthcare workers and health systems managers to better understand the contexts within which each party operates [3]. Fourthly, we have highlighted several lessons learnt from the COVID19 pandemic, which was a shock to entire healthcare systems across the world. Because of the range of geopolitical contexts covered, extracted lessons may be applied across different healthcare systems and organisations. Our framework increases the focus on relational aspects of resilience, particularly in the trans-event domains (Figures 4 & 5). The importance of which is also reflected within the recently renewed interest into integrated care in the UK.

Several limitations persist. We do not focus on the micro-level, even though humans are undoubtedly the most important components of healthcare systems, both as staff and service-users. Empirical studies are excluded. This means that while we were able to maintain breadth, we inadvertently lose resolution. In addition, knowledge continues to be discovered and some will not have made it into reviews yet. Therefore, while this review of reviews is timely, we acknowledge that resilience is a dynamic process [28]. Several important empirical studies are underway to unpick key relational factors between hierarchical levels and how they may support or hinder adaptive capacity [11 78]. These studies should help to deepen understanding into healthcare resilience. We chose not to include grey literature due to volume of work, but this potentially excludes some important information from this review. This is also the case for the language restriction. There is a risk that language selection bias present in other reviews is amplified in this review. Nevertheless, many reviews included studies from a range of countries. This partially mitigates the above issues.

Frameworks can be considered tools for systematically approaching resilience, but require adaptation towards other more contextualised situations (Appendix 4). We

For peer review only - http://bmjopen.bmj.com/site/about/guidelines.xhtml

have provided only limited guidance in individual aspects such as health financing, infrastructure, or leadership styles. There is a wealth of literature for these purposes. We instead focus on a broad overview across disciplines and hierarchical levels.

Future reviews should state clear research questions, adopt systematic search strategies, and use robust data analyses techniques. These would maximise the quality of evidence generated. Future priorities arising from this research include the need to examine multi-level resilience with empirical studies [11], exploring how each level's resilience contributes to other levels [3], and gaining deeper understanding of the nature and extent of interorganisational relationships and how they affect resilience.

5. Conclusion

Our review of reviews provides a broad overview of healthcare resilience. The meta-narrative approach adopted recognises the contribution from several disciplines and paradigms within this field. We have highlighted the tendency for studies to focus on individual hierarchical levels and limited timescales, but also acknowledge the need and current desire to develop more interdisciplinary, cross-sectoral, and multi-level approaches to healthcare resilience. Our framework for healthcare resilience crosses hierarchical and time boundaries, providing a broad and reflexive overview of healthcare resilience. Understanding the balances between sometimes opposing concepts, and contrasting priorities of different professional groups or hierarchies, is key to building resilience for the future.

For peer review only

Contributorship statement

Concept – MT, AMcC, PD. Planning and design – MT. Screening – MT, GP. Additional reviewers – AMcC, PD. Data collection, analysis, and interpretation – MT, GP. Writing – MT. Editing – all authors

Competing interests

None declared.

Funding

No funding.

Data sharing statement

All data relevant to the study are included in the article or uploaded as supplementary information.

Ethics Approval

This study does not involve human participants and ethical approval was not required.

Patient and Public Involvement

There was no patient or public involvement in this research.

For peer review only

References

1. Barasa E, Mbau R, Gilson L. What Is Resilience and How Can It Be Nurtured? A Systematic Review of Empirical Literature on Organizational Resilience. *International Journal of Health Policy and Management* 2018;7(6):491-503. doi: 10.15171/ijhpm.2018.06
2. Oppenheim B, Gallivan M, Madhav NK, et al. Assessing global preparedness for the next pandemic: development and application of an Epidemic Preparedness Index. *BMJ Global Health* 2019;4(1):e001157. doi: 10.1136/bmjgh-2018-001157
3. Tan MZY. Resilience is a dirty word: misunderstood, and how we can truly build it. *Critical Care* 2022;26(1):168. doi: 10.1186/s13054-022-04040-x
4. Meyer D, Bishai D, Ravi SJ, et al. A checklist to improve health system resilience to infectious disease outbreaks and natural hazards. *BMJ Glob Health* 2020;5(8) doi: 10.1136/bmjgh-2020-002429
5. Park JJH, Mogg R, Smith GE, et al. How COVID-19 has fundamentally changed clinical research in global health. *The Lancet Global Health* 2021;9(5):e711-e20. doi: https://doi.org/10.1016/S2214-109X(20)30542-8
6. Razai MS, Chaudhry UAR, Doerholt K, et al. Covid-19 vaccination hesitancy. *BMJ* 2021;373:n1138. doi: 10.1136/bmj.n1138
7. Pillay M, Morel G. Measuring Resilience Engineering: An Integrative Review and Framework for Bench-Marking Organisational Safety. *Safety* 2020;6(3) doi: 10.3390/safety6030037
8. Curtis S, Fair A, Wistow J, et al. Impact of extreme weather events and climate change for health and social care systems. *Environmental Health: A Global Access Science Source* 2017;16 doi: 10.1186/s12940-017-0324-3
9. Greenhalgh T, Robert G, Macfarlane F, et al. Storylines of research in diffusion of innovation: a meta-narrative approach to systematic review. *Soc Sci Med* 2005;61(2):417-30. doi: 10.1016/j.socscimed.2004.12.001 [published Online First: 2005/05/17]
10. Mondal S, Van Belle S, Maioni A. Learning from intersectoral action beyond health: a meta-narrative review. *Health Policy Plan* 2021;36(4):552-71. doi: 10.1093/heapol/czaa163
11. Aase K, Guise V, Billett S, et al. Resilience in Healthcare (RiH): A longitudinal research programme protocol. *BMJ Open* 2020;10(10) doi: 10.1136/bmjopen-2020-038779
12. Wiig S, Aase K, Billett S, et al. Defining the boundaries and operational concepts of resilience in the resilience in healthcare research program. *BMC Health Services Research* 2020;20(1) doi: 10.1186/s12913-020-05224-3
13. Li L, Liao S, Yuan J, et al. Analyzing Healthcare Facility Resilience: Scientometric Review and Knowledge Map. *Frontiers in Public Health* 2021;9 doi: 10.3389/fpubh.2021.764069
14. Biddle L, Wahedi K, Bozorgmehr K. Health system resilience: A literature review of empirical research. *Health Policy and Planning* 2020;35(8):1084-109. doi:

For peer review only - http://bmjopen.bmj.com/site/about/guidelines.xhtml

10.1093/heapol/czaa032
15. Koeva S, Rohova M. Health system resilience: concept development. *Journal of Imab* 2020;26(3):3251-58. doi: 10.5272/jimab.2020263.3251
16. Zhong S, Clark M, Hou X-Y, et al. Development of hospital disaster resilience: conceptual framework and potential measurement. *Emergency Medicine Journal* 2014;31(11):930-U8901. doi: 10.1136/emermed-2012-202282
17. Son C, Sasangohar F, Neville T, et al. Investigating resilience in emergency management: An integrative review of literature. *Appl Ergon* 2020;87 doi: 10.1016/j.apergo.2020.103114
18. Almedom AM. Resilience research and policy/practice discourse in health, social, behavioral, and environmental sciences over the last ten years. *African health sciences* 2008;8 Suppl 1:S5-13.
19. Calkins J. Moving forward after Sendai: How countries want to use science, evidence and technology for disaster risk reduction. *PLoS Currents* 2015;7(DISASTERS) doi: 10.1371/currents.dis.22247d6293d4109d09794890bcda1878
20. McClelland AG, Shaw D, O'Grady N, et al. Recovery for Development: A Multi-Dimensional, Practice-Oriented Framework for Transformative Change Post-Disaster. *The Journal of Development Studies* 2022:1-20. doi: 10.1080/00220388.2022.2130055
21. Conti A, Valente M, Paganini M, et al. Knowledge Gaps and Research Priorities on the Health Effects of Heatwaves: A Systematic Review of Reviews. *Int J Environ Res Public Health* 2022;19(10) doi: 10.3390/ijerph19105887 [published Online First: 20220512]
22. Ries M. The COVID-19 Infodemic: Mechanism, Impact, and Counter-Measures—A Review of Reviews. *Sustainability* 2022;14(5):2605.
23. Gough D. Meta-narrative and realist reviews: guidance, rules, publication standards and quality appraisal. *BMC Medicine* 2013;11(1):22. doi: 10.1186/1741-7015-11-22
24. Wong G, Greenhalgh T, Westhorp G, et al. RAMESES publication standards: meta-narrative reviews. *BMC Medicine* 2013;11(1):20. doi: 10.1186/1741-7015-11-20
25. Tan M, Prager G. Resilience in healthcare: a meta-narrative review of reviews. CRD42022314729: PROSPERO; 2022 [Available from: https://www.crd.york.ac.uk/prospero/display_record.php?ID=CRD42022314729 accessed Mar 2022.
26. Page MJ, McKenzie JE, Bossuyt PM, et al. The PRISMA 2020 statement: an updated guideline for reporting systematic reviews. *BMJ* 2021;372:n71. doi: 10.1136/bmj.n71
27. Gurney A. Resilience in the UK and other OECD economies: Treasury Economic Working Paper No.2 London: HM Treasury; 2008 [Available from: https://webarchive.nationalarchives.gov.uk/ukgwa/20081112160625/http:/www.hm-treasury.gov.uk/d/bud08_workingpaper2_557_.pdf.
28. Bealt J, Powell D, Shaw D. Benefits of involving public involvement in emergency planning Manchester: University of Manchester; 2021 [Available from: https://committees.parliament.uk/writtenevidence/21957/pdf/ accessed

For peer review only - http://bmjopen.bmj.com/site/about/guidelines.xhtml

Aug 2021.
29. Abdullah MA, Shaikh BT. Confusion and denial: need for systems thinking to understand the HIV epidemic in Pakistan. *J Ayub Med Coll Abbottabad* 2014;26(3):396-400.
30. Butler A, Hall H, Copnell B. A Guide to Writing a Qualitative Systematic Review Protocol to Enhance Evidence-Based Practice in Nursing and Health Care. *Worldviews Evid Based Nurs* 2016;13(3):241-9. doi: 10.1111/wvn.12134 [published Online First: 20160120]
31. Smith V, Devane D, Begley CM, et al. Methodology in conducting a systematic review of systematic reviews of healthcare interventions. *BMC Med Res Methodol* 2011;11(1):15. doi: 10.1186/1471-2288-11-15 [published Online First: 20110203]
32. Smith V, Devane D, Begley CM, et al. Methodology in conducting a systematic review of systematic reviews of healthcare interventions. *BMC Medical Research Methodology* 2011;11(1):15. doi: 10.1186/1471-2288-11-15
33. Aromataris E, Fernandez R, Godfrey C, et al. Summarizing systenatic reviews: methodological development, conduct and reporting of an Umbrella review approach. . *Int J Evid Based Healthc* 2015;13:132-40.
34. Noble H, Mitchell G. What is grounded theory? *Evidence Based Nursing* 2016;19(2):34-35. doi: 10.1136/eb-2016-102306
35. Brand FS, Jax K. Focusing the Meaning(s) of Resilience: Resilience as a Descriptive Concept and a Boundary Object. *Ecology and Society* 2007;12(1)
36. Turenne CP, Gautier L, Degroote S, et al. Conceptual analysis of health systems resilience: A scoping review. *Soc Sci Med* 2019;232:168-80. doi: 10.1016/j.socscimed.2019.04.020 [published Online First: 20190430]
37. Hosseini S, Barker K, Ramirez-Marquez JE. A review of definitions and measures of system resilience. *Reliability Engineering & System Safety* 2016;145:47-61. doi: https://doi.org/10.1016/j.ress.2015.08.006
38. Augustynowicz A, Opolski J, Waszkiewicz M. Resilient Health and the Healthcare System. A Few Introductory Remarks in Times of the COVID-19 Pandemic. *Int J Environ Res Public Health* 2022;19(6) doi: 10.3390/ijerph19063603
39. Zhong S, Clark M, Hou X-Y, et al. Proposing and developing a definition and conceptual framework for health care resilience to cope with disasters. *Emergencias* 2014;26(1):69-77.
40. Biggs R, Schlüter M, Schoon ML. Principles for Building Resilience: Cambridge University Press 2015.
41. Florez-Jimenez M, Muñoz-Villamizar A, Lleo A. Exploring the relationship between sustainability, resilience, and purpose in the context of corporations: a comprehensive literature review SSRN; 2021 [Available from: https://papers.ssrn.com/sol3/papers.cfm?abstract_id=3944148 accessed Nov 2021.
42. Hollnagel E, Wears R, Braithwaite J. From Safety-I to Safety-II: A White Paper. 2015.
43. Berg SH, Akerjordet K, Ekstedt M, et al. Methodological strategies in resilient health care studies: An integrative review. *Safety Science* 2018;110:300-12.

For peer review only - http://bmjopen.bmj.com/site/about/guidelines.xhtml

doi: 10.1016/j.ssci.2018.08.025

44. Rangachari P, J LW. Preserving Organizational Resilience, Patient Safety, and Staff Retention during COVID-19 Requires a Holistic Consideration of the Psychological Safety of Healthcare Workers. *Int J Environ Res Public Health* 2020;17(12) doi: 10.3390/ijerph17124267 [published Online First: 2020/06/19]
45. Stoverink AC, Kirkman BL, Mistry S, et al. Bouncing Back Together: Toward a Theoretical Model of Work Team Resilience. *Academy of Management Review* 2020;45(2):395-422. doi: 10.5465/amr.2017.0005
46. Ayuningtyas D, Windiarti S, Sapoan Hadi M, et al. Disaster preparedness and mitigation in indonesia: A narrative review. *Iranian Journal of Public Health* 2021;50(8):1536-46.
47. Romanello M, McGushin A, Di Napoli C, et al. The 2021 report of the Lancet Countdown on health and climate change: code red for a healthy future. *The Lancet* 2021;398(10311):1619-62. doi: 10.1016/S0140-6736(21)01787-6
48. Kruk ME, Ling EJ, Bitton A, et al. Building resilient health systems: a proposal for a resilience index. *BMJ* 2017;357:j2323. doi: 10.1136/bmj.j2323
49. Carrington MA, Ranse J, Hammad K. The impact of disasters on emergency department resources: review against the Sendai framework for disaster risk reduction 2015–2030. *Australas Emerg Care* 2021;24(1):55-60. doi: 10.1016/j.auec.2020.09.003
50. Cartwright C, Hall M, Lee ACK. The changing health priorities of earthquake response and implications for preparedness: a scoping review. *Public Health* 2017;150:60-70. doi: 10.1016/j.puhe.2017.04.024
51. Challen K, Lee AC, Booth A, et al. Where is the evidence for emergency planning: A scoping review. *BMC Public Health* 2012;12(1) doi: 10.1186/1471-2458-12-542
52. Fallah-Aliabadi S, Ostadtaghizadeh A, Ardalan A, et al. Towards developing a model for the evaluation of hospital disaster resilience: A systematic review. *BMC Health Services Research* 2020;20(1) doi: 10.1186/s12913-020-4915-2
53. Luke J, Franklin R, Aitken P, et al. Safer hospital infrastructure assessments for socio-natural disaster - A scoping review. *Prehosp Disaster Med* 2021;36(5):627-35. doi: 10.1017/S1049023X21000650
54. Xin YT, Xu KY. Hospital emergency management research in China: trends and challenges. *Emerg Med J* 2012;29(5):353-7. doi: 10.1136/emermed-2011-200512 [published Online First: 20111103]
55. Nuzzo JB, Meyer D, Snyder M, et al. What makes health systems resilient against infectious disease outbreaks and natural hazards? Results from a scoping review. *BMC Public Health* 2019;19(1) doi: 10.1186/s12889-019-7707-z
56. Iflaifel M, Lim RH, Ryan K, et al. Resilient Health Care: a systematic review of conceptualisations, study methods and factors that develop resilience. *BMC Health Serv Res* 2020;20(1):324. doi: 10.1186/s12913-020-05208-3 [published Online First: 2020/04/19]
57. Hollnagel E. Epilogue: RAG–the resilience analysis grid. Resilience engineering in practice: CRC Press 2017:275-96.
58. WHO. Monitoring the building blocks of health systems 2010 [accessed Nov 2019.

For peer review only - http://bmjopen.bmj.com/site/about/guidelines.xhtml

59. Tessema GA, Kinfu Y, Dachew BA, et al. The COVID-19 pandemic and healthcare systems in Africa: a scoping review of preparedness, impact and response. *Bmj Global Health* 2021;6(12) doi: 10.1136/bmjgh-2021-007179
60. Turner S, Botero-Tovar N, Herrera MA, et al. Systematic review of experiences and perceptions of key actors and organisations at multiple levels within health systems internationally in responding to COVID-19. *Implementation Science* 2021;16(1) doi: 10.1186/s13012-021-01114-2
61. Alami H, Lehoux P, Fleet R, et al. How Can Health Systems Better Prepare for the Next Pandemic? Lessons Learned From the Management of COVID-19 in Quebec (Canada). *Frontiers in public health* 2021;9:671833. doi: 10.3389/fpubh.2021.671833
62. Grimm PY, Oliver S, Merten S, et al. Enhancing the Understanding of Resilience in Health Systems of Low- and Middle-Income Countries: A Qualitative Evidence Synthesis. *International Journal of Health Policy and Management* 2021 doi: 10.34172/ijhpm.2020.261
63. Kamara JK, Akombi BJ, Agho K, et al. Resilience to climate-induced disasters and its overall relationship to well-being in southern africa: A mixed-methods systematic review. *Int J Environ Res Public Health* 2018;15(11) doi: 10.3390/ijerph15112375
64. Tippong D, Petrovic S, Akbari V. A Review of Applications of Operational Research in Healthcare Coordination in Disaster Management. *Eur J Oper Res* 2021 doi: 10.1016/j.ejor.2021.10.048 [published Online First: 20211028]
65. Van Ryneveld M, Schneider H, Lehmann U. Looking back to look forward: a review of human resources for health governance in South Africa from 1994 to 2018. *Human Resources for Health* 2020;18(1) doi: 10.1186/s12960-020-00536-1
66. Anderson JE, Aase K, Bal R, et al. Multilevel influences on resilient healthcare in six countries: An international comparative study protocol. *BMJ Open* 2020;10(12) doi: 10.1136/bmjopen-2020-039158
67. HealthFoundation. Quality improvement made simple: What everyone should know about health care quality improvement: The Health Foundation; 2021 [Available from: https://www.health.org.uk/sites/default/files/QualityImprovementMadeSimple.pdfOct 2022.
68. Rezaei F, Maracy MR, Yarmohammadian MH, et al. Hospitals preparedness using WHO guideline: A systematic review and meta-analysis. *Hong Kong Journal of Emergency Medicine* 2018;25(4):211-22. doi: 10.1177/1024907918760123
69. Ayanore MA, Amuna N, Aviisah M, et al. Towards Resilient Health Systems in Sub-Saharan Africa: A Systematic Review of the English Language Literature on Health Workforce, Surveillance, and Health Governance Issues for Health Systems Strengthening. *Annals of Global Health* 2019;85(1) doi: 10.5334/aogh.2514
70. Hasan MZ, Neill R, Das P, et al. Integrated health service delivery during COVID-19: A scoping review of published evidence from low-income and lower-middle-income countries. *BMJ Global Health* 2021;6(6) doi: 10.1136/bmjgh-2021-005667

For peer review only - http://bmjopen.bmj.com/site/about/guidelines.xhtml

71. Ravi SJ, Meyer D, Cameron E, et al. Establishing a theoretical foundation for measuring global health security: A scoping review. *BMC Public Health* 2019;19(1) doi: 10.1186/s12889-019-7216-0
72. Fridell M, Edwin S, Schreeb JV, et al. Health system resilience: what are we talking about? A scoping review mapping characteristics and keywords. *International Journal of Health Policy and Management* 2020;9(1):6-16. doi: 10.15171/ijhpm.2019.71
73. Blumenstock J, Bakker G, Jarris PE. Measuring Preparedness: The National Health Security Preparedness Index. *Journal of Public Health Management and Practice* 2014;20(3):361-63. doi: 10.1097/phh.0000000000000073
74. Haldane V, Zhang Z, Abbas RF, et al. National primary care responses to COVID-19: a rapid review of the literature. *BMJ Open* 2020;10(12):e041622. doi: 10.1136/bmjopen-2020-041622 [published Online First: 20201208]
75. Lapão LV, Silva A, Pereira N, et al. Ebola impact on African health systems entails a quest for more international and local resilience: the case of African Portuguese speaking countries. *Pan Afr Med J* 2015;22 Suppl 1(Suppl 1):15. doi: 10.11694/pamj.supp.2015.22.1.6653 [published Online First: 20151011]
76. Tin N, Lwin S, Kyaing NN, et al. An approach to health system strengthening in the Union of Myanmar. *Health Policy* 2010;95(2-3):95-102. doi: 10.1016/j.healthpol.2009.11.013
77. Sanderson M, Allen P, Osipovic D, et al. The Developing Architecture of System Management: Integrated Care Systems and Sustainability and Transformation Partnerships - Interim report: PRUComm; 2021 [Available from: https://prucomm.ac.uk/assets/uploads/PRUComm_ICS_study_interim_report_feb_2021.pdf accessed Jul 2022.
78. Anderson JE, Aase K, Bal R, et al. Multilevel influences on resilient healthcare in six countries: an international comparative study protocol. *BMJ Open* 2020;10(12):e039158. doi: 10.1136/bmjopen-2020-039158
79. Petersen EE, Lyng HB, Ree E, et al. Relationship between management and resilience in healthcare: A study protocol for a systematic review. *BMJ Open* 2021;11(7) doi: 10.1136/bmjopen-2020-047855
80. Bell JA, Nuzzo JB. Global Health Security Index: Advancing Collective Action and Accountability Amid Global Crisis: NTI/John Hopkins center for health security; 2021 [Available from: www.GHSIndex.org accessed Mar 2022.
81. Baum F, Freeman T, Musolino C, et al. Explaining covid-19 performance: what factors might predict national responses? *BMJ* 2021;372:n91. doi: 10.1136/bmj.n91
82. Rameshshanker V, Wyngaarden S, Lau LL, et al. Health system resilience to extreme weather events in Asia-Pacific: a scoping review. *Clim Dev* 2021;13(10):944-58. doi: 10.1080/17565529.2020.1870425
83. Penaloza GA, Saurin TA, Formoso CT, et al. A resilience engineering perspective of safety performance measurement systems: A systematic literature review. *Safety Science* 2020;130 doi: 10.1016/j.ssci.2020.104864
84. Carthey J, de Leval MR, Reason JT. Institutional resilience in healthcare systems. *Quality in Health Care* 2001;10(1):29-32. doi: 10.1136/qhc.10.1.29
85. Evans L, Rhodes A, Alhazzani W, et al. Surviving sepsis campaign: international

For peer review only - http://bmjopen.bmj.com/site/about/guidelines.xhtml

guidelines for management of sepsis and septic shock 2021. *Intensive Care Med* 2021;47(11):1181-247. doi: 10.1007/s00134-021-06506-y [published Online First: 20211002]

86. Fitzpatrick F, Tarrant C, Hamilton V, et al. Sepsis and antimicrobial stewardship: two sides of the same coin. *BMJ Quality & Safety* 2019;28(9):758-61. doi: 10.1136/bmjqs-2019-009445

87. Vincent JL. The Clinical Challenge of Sepsis Identification and Monitoring. *PLoS Med* 2016;13(5):e1002022. doi: 10.1371/journal.pmed.1002022 [published Online First: 20160517]

88. Linkov I, Trump BD, Trump J, et al. Resilience stress testing for critical infrastructure. *Int J Disaster Risk Reduct* 2022;82:103323. doi: https://doi.org/10.1016/j.ijdrr.2022.103323

89. Patriarca R, Di Gravio G, Woltjer R, et al. Framing the FRAM: A literature review on the functional resonance analysis method. *Safety Science* 2020;129:104827. doi: https://doi.org/10.1016/j.ssci.2020.104827

90. Njah J, Hansoti B, Adeyami A, et al. Measuring for Success: Evaluating Leadership Training Programs for Sustainable Impact. *Ann Glob Health* 2021;87(1):63. doi: 10.5334/aogh.3221 [published Online First: 20210712]

91. Smith AF, Plunkett E. People, systems and safety: resilience and excellence in healthcare practice. *Anaesthesia* 2019;74(4):508-17. doi: 10.1111/anae.14519

92. Raymond CB, Ward PR. Community-level experiences, understandings, and responses to COVID-19 in low-and middle-income countries: A systematic review of qualitative and ethnographic studies. *Int J Environ Res Public Health* 2021;18(22) doi: 10.3390/ijerph182212063

For peer review only

For peer review only - http://bmjopen.bmj.com/site/about/guidelines.xhtml

Figure captions

Figure 1: Search strategy

Figure 2: PRISMA diagram

Figure 3: Healthcare resilience: hierarchical levels and selected paradigm timelines with historical highlights. Legend: WHO = World Health Organisation. DRR: Disaster Risk Reduction. FEMA: Federal Emergency Management Agency. GHSI: Global Health Security Index.

Figure 4: An interdisciplinary, cross-sectoral, and multi-level definition and conceptual framework for healthcare resilience. *Legend: Defining features are listed as headings within the pre-, intra-, post- and trans-event time domains.*

Figure 5: Details of the interdisciplinary, cross-sectoral, and multi-level framework for healthcare resilience. Concepts are presented with frequency of occurrences as n-numbers. They are further divided into components, along with example considerations extracted from reviews.

For peer review only

For peer review only - http://bmjopen.bmj.com/site/about/guidelines.xhtml

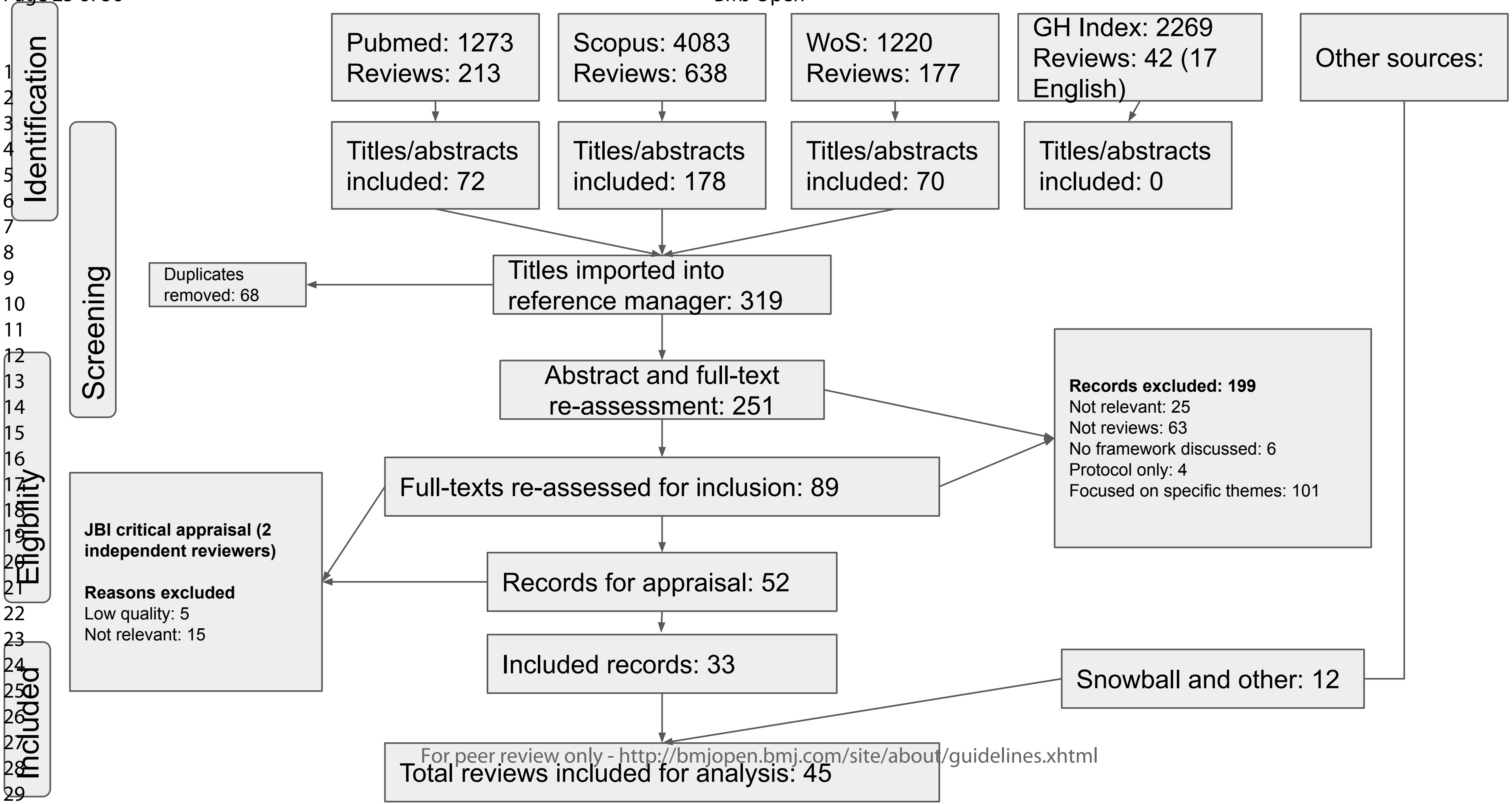
Identification
Screening
Eligibility
Included
Pubmed: 1273
Reviews: 213
Scopus: 4083
Reviews: 638
WoS: 1220
Reviews: 177
GH Index: 2269
Reviews: 42 (17 English)
Other sources:
Titles/abstracts included: 72
Titles/abstracts included: 178
Titles/abstracts included: 70
Titles/abstracts included: 0
Duplicates removed: 68
Titles imported into reference manager: 319
Abstract and full-text re-assessment: 251
Records excluded: 199
Not relevant: 25
Not reviews: 63
No framework discussed: 6
Protocol only: 4
Focused on specific themes: 101
Full-texts re-assessed for inclusion: 89
JBI critical appraisal (2 independent reviewers)
Reasons excluded
Low quality: 5
Not relevant: 15
Records for appraisal: 52
Included records: 33
Snowball and other: 12
Total reviews included for analysis: 45

Search Strategy

Scopus (Elsevier)

(TITLE-ABS-KEY ("resilien*"OR"strengthening") AND TITLE-ABS-KEY ("system"OR"organi*ation") AND TITLE-ABS-KEY ("health"OR"hospital"OR"healthcare") AND TITLE-ABS-KEY ("crisis"OR"disaster"OR“epidemic”OR“pandemic”))

Web of Science (Clarivate)

("resilience" OR "resilient" OR "strengthen") AND ("health") AND ("organisation" OR "systems") AND ("crisis" OR "disaster" OR "epidemic" OR "pandemic”)

PubMed (NIH)

((("resilien*"OR"strengthening") AND ("system"OR"organisation")) AND (health)) AND ("crisis"OR"disaster"OR"epidemic"OR"pandemic”)

Global Index Medicus (WHO)

(tw:(Resilien* OR strengthening)) AND (tw:(System OR Organisation))

Inclusion Criteria

- 2008 - 2022
- Scholarly articles (reviews) published in peer-reviewed academic databases
- Related to healthcare organisations and systems

Exclusion Criteria

- Non-healthcare
- Focused on individual, psychological or community resilience
- Concerned with a particular disease or health condition outside of epidemic/pandemic context
- Non-English

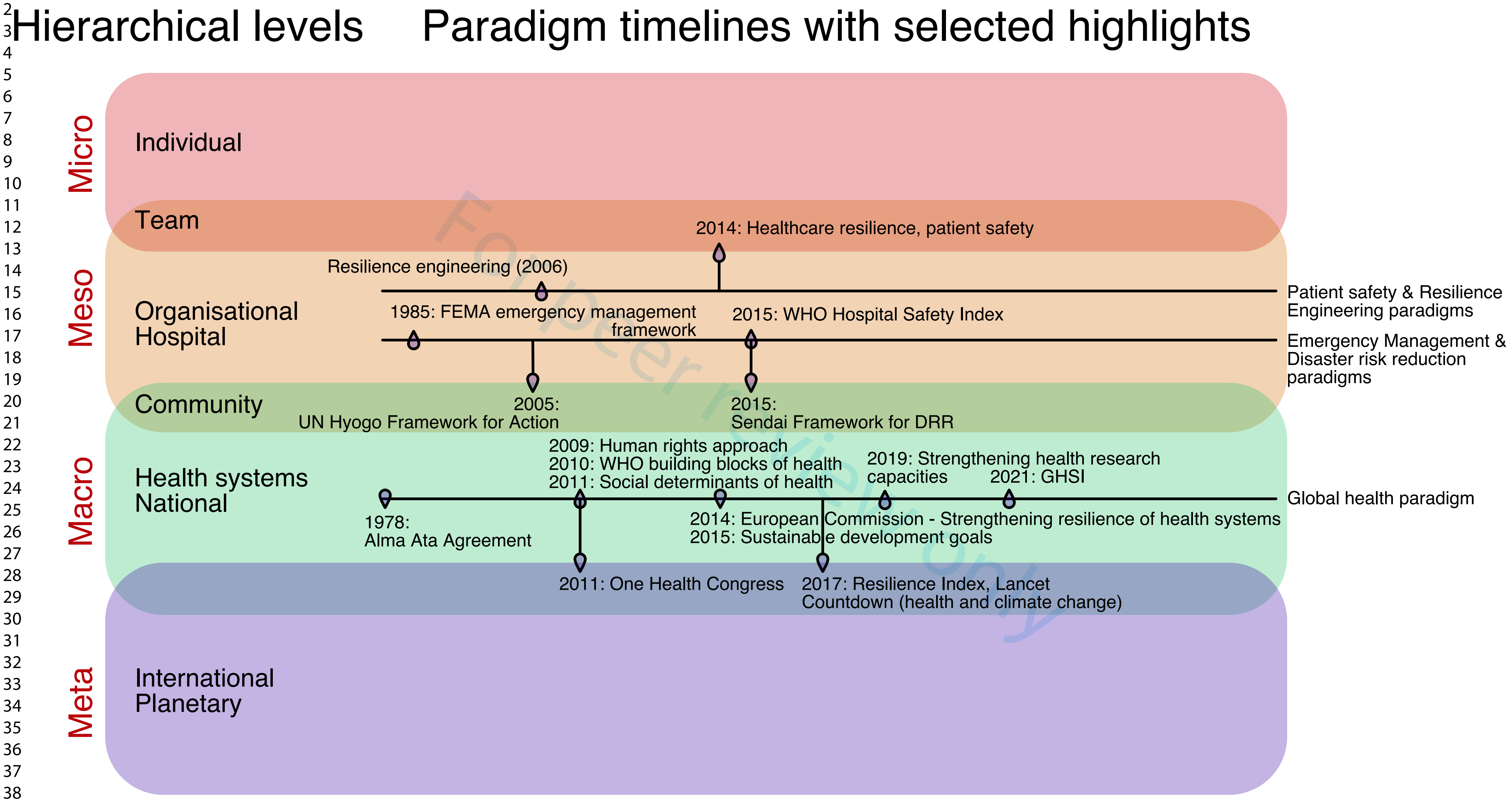

For peer review only

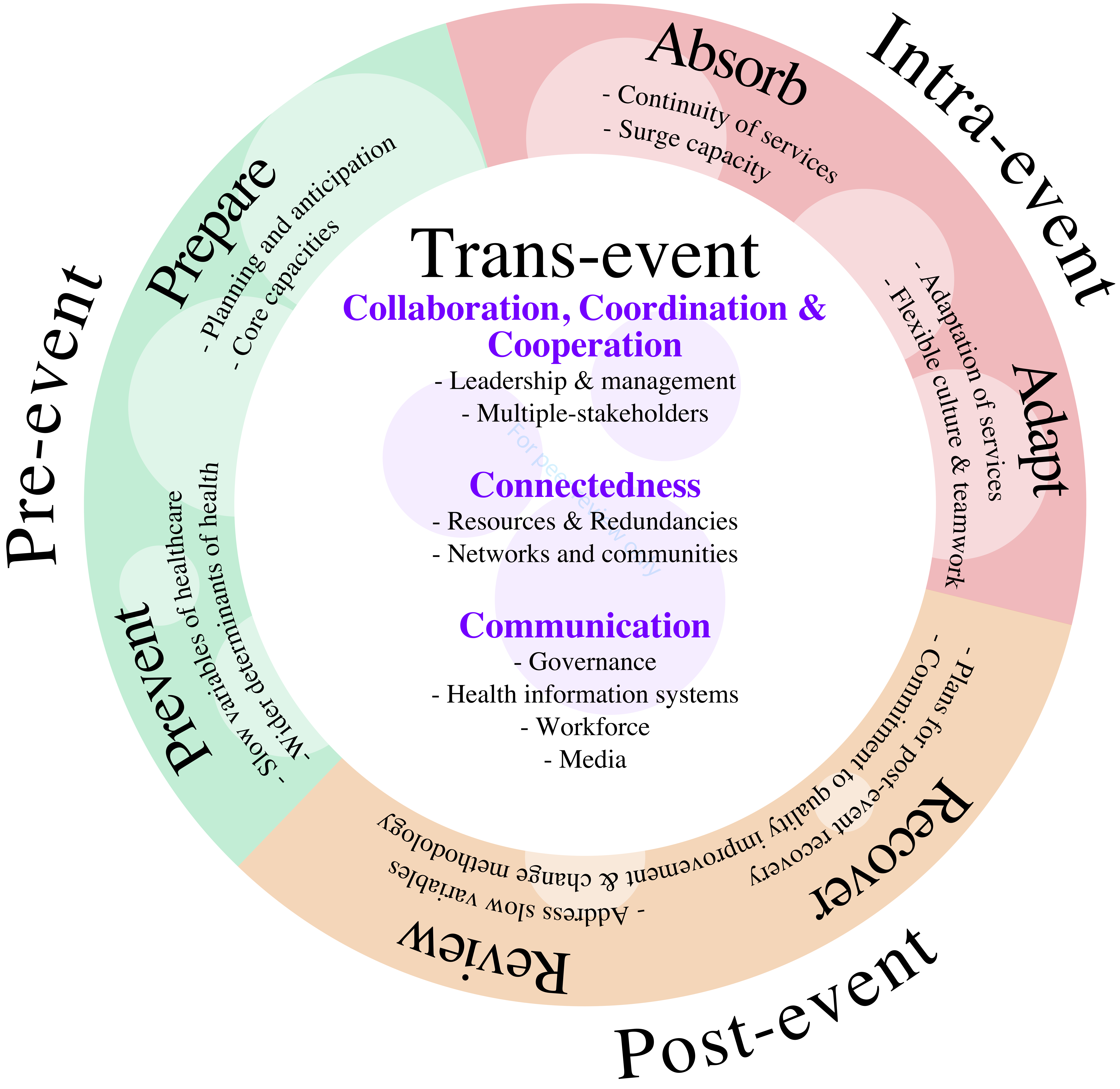
Pre-event
Prepare
- Planning and anticipation
- Core capacities
Prevent
- Slow variables of healthcare
- Wider determinants of health
Intra-event
Absorb
- Continuity of services
- Surge capacity
Adapt
- Adaptation of services
- Flexible culture & teamwork
Post-event
Recover
- Plans for post-event recovery
- Commitment to quality improvement & change methodology
Review
- Address slow variables
Trans-event
Collaboration, Coordination & Cooperation
- Leadership & management
- Multiple-stakeholders
Connectedness
- Resources & Redundancies
- Networks and communities
Communication
- Governance
- Health information systems
- Workforce
- Media

Interdisciplinary, Cross-sectoral, Multi-level framework for Healthcare Resilience

	Themes	Concepts (frequency)	Component	Example considerations
Pre-event	Prevent	Slow variables (n=8)	Healthcare access and range of services	Universal healthcare, geographical access factors, equality in access
			Acting on risks identified	Health inequalities, at risk populations
			Strengthening healthcare infrastructure	Ventilation and air-conditioning systems, integrity of buildings, additional cliincal spaces
			Healthcare expenditure and funding channels	WHO Building Blocks of Health
			Staff retention and education	From COVID19: burnout, wellbeing, development opportunities. Plan for attrition.
		Wider determinants (n=10)	Social determinants of health	Water, sanitation, education, employment
			Health inequalities	From COVID19: disproportionate health outcomes for ethnic minorities and lower socioeconomic groups
			Physical and critical infrastructure	Road and access, buildings and public services
			Environmental considerations	One Health movement, planetary health
	Prepare	Planning & anticipation (n=12)	Disaster or crisis protocols and policies	Sendai Framework for Disaster Risk Reduction, FEMA emergency management cycle
			Strengthen community and long-term care	From COVID19: lack of workers in care settings after initial surge
			Modelling disasters	
			Strengthen interorganisational networks	Transport and supply chains, voluntary sectors, faith-based organisations
			Training and maintenance of skills	Disaster and major incident training, simulation and drills.
		Core capacities (n=11)	Beds, staff, equipment, medicines	Existing resources and emergency stockpiles
			Plans for utilising surge capacities	Equipment, spaces, alternative facilities
Intra-event	Absorb	Continuity of services (n=19)	Maintain essential services	Maternity, paediatrics, emergency medicine, intensive care
			Planning and involving spontaneous volunteers	ISO 22319:2017
			Mass-casualty triage protocols	Royal College of Emergency Medicine guidance
			Ensure access to healthcare services	Consider hard-to-reach populations
		Surge capacity (n=16)	Mobilising core and surge capacities	
			Activating policies and processes for major incidents	
			Donation standards and distribution	
	Adapt	Adaptation of services (n=17)	Temporary reduction in elective services	From COVID19: reduction in volume of services (e.g. USA's Crisis Standards of Care)
			Temporary incident command and communication system	
			Adopting new technologies	From COVID19: telemedicine and videoconferencing
		Flexible culture and teamwork (n=7)	Collective sensemaking and fast variables in healthcare	From COVID19: improvisations and workarounds characterised resilient adaptation.
			Trade-offs, workarounds and improvisation	WAI/WAD. Actuality:Potentiality ratios
Post-event	Recover	Plans for post-event recovery (n=8)	Acute psychological interventions for staff	
			Staff for increased long-term care	Predicted late surge in community, complex and long-term care
			Strategies for community recovery	Recovery for Development Framework. National Consortium for Societal Resilience
	Review	Commitment to quality improvement and change methodology (n=16)	Quality improvement method or framework	Evaluation reports, vulnerability analysis, risk assessments. Safety 1 & 2
			Evaluation and data collection systems	
		Address slow variables (n=8)	Organisational or system adaptation based on lessons learnt	
Trans-event	Collaboration, Coordination, Cooperation	Leadership & management (n=20, 12 respectively)	Effective leadership and trust	Open style leadership during crisis
			Crisis leadership training	
		Multiple stakeholders (n= 3)	Links with surveillance organisations	Meteorological institutes, public services, public health authorities
			Cross-sector collaboration	Patient groups, manufacturers, volunteer and faith-based sector, private, non-profit
			Diverse approaches to support creativiity and innovation	Kruk's Resilience Index, Principles of Socio-ecological resilience, Empirical studies of Uganda's public corporations
	Connectedness	Resources and redundancies (n=22)	Supply and stock management processes	
			Plans for pooling resources	From COVID19: Capacity transfers in intensive care
		Networks and communities (n=16)	Integrated care	UK Health and Care Act 2022
			Dialogue with community leaders & volunteers	
			Regional and international links and diplomatic ties	International emergency response for earthquakes and natural disasters
	Communication	Governance (n=5)	Ensure multiple feedback loops	
			Civil registration and statistics systems	
		Health information systems (n=7)	Multi-level surveillance strategies	Public health functions, routine data collection and dissemination, data sharing and integration, early warning systems
		Workforce (n=17)	Feedback & communication mechanisms across hierarchical levels	Staff forums, incident reporting
			Staff recognition	
		Media (n=3)	Accuracy of information disssemination	From COVID19: false news and vaccine hesitancy
			Population trust in health system	

Supplementary file

Healthcare resilience - A meta-narrative systematic review and synthesis of reviews

For peer review only

List of supplementary materials

Appendix 1: Additional details of methodology
Appendix 2: Definition word cloud and word occurrence table
Appendix 3: Data extraction table from reviews
Appendix 4: Healthcare resilience framework summary

Appendix 1: Additional methodological details

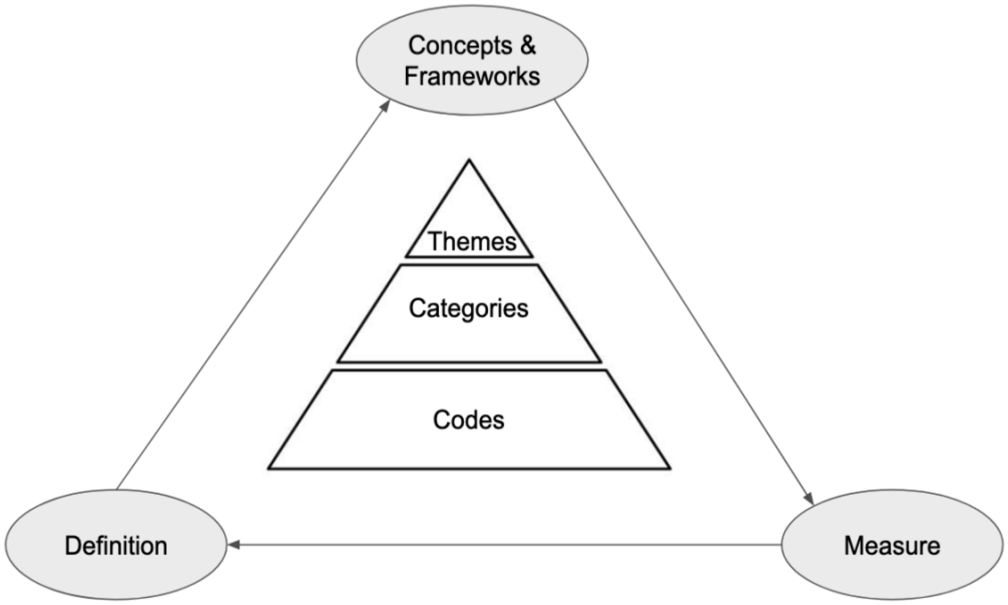

Appendix 1.1: Theoretical framework describing three tiers of analysis within three foci.

In choosing our approach, we referred to Xiao et al.'s typology of reviews [1]. We sought to describe and extend the current understanding of healthcare resilience. Since resilience has been studied within a range of academic disciplines, across multiple sectors and a numerous hierarchies and levels, a meta-narrative was the most appropriate form of review to perform. This aims to consolidate the various research traditions involved in the study of a broad topic, thereby forming an overarching synthesis of the landscape of said topic [2]. A related method is the realist review, which seeks to distil circumstantial conditions conferring success of various interventions [3]. Traditional systematic reviews or meta-analyses were unsuitable methodologies for this study due to several reasons. In particular, studies into resilience include multiple methodologies, both quantitative and qualitative, over a span of disciplines which include evolving terminology and nomenclature. The homogeneity required for a traditional systematic review or quantitative measures which form the basis of a meta-analysis do not feature in resilience studies [4 5].

The key principles of meta-narrative reviews are pragmatism, pluralism,

historicity, contestation, reflexivity and peer review [6]. This review of reviews provides a multidisciplinary and multi-level view of healthcare resilience. It maps current understanding and presents various conceptualisations and frameworks (pluralism). This will thus be useful for a multitude of actors including healthcare leaders, policymakers and academics (pragmatism). Examining the various research traditions which converge in this topic, it will both chart its evolution and explore various perspectives (historicity). Differences in definitions, measures and conceptualisations will be highlighted and discussed (contestation). The protocol is updated according to the findings, and future agendas are proposed in line with emergent data and insights (reflexivity). Finally, independent reviewers are involved throughout the process, while results are shared with a broader research team focusing on whole-of-society resilience (peer review).

Tool or method	Reason for unsuitability
Cochrane protocol [7]	Requires established quantitative metrics
Cochrane qualitative tool [8]	Only applicable for empirical studies. Unsuitable for mixed or multi-method studies
Mixed Methods Assessment Tool (MMAT)[9]	Heavily weighted towards quantitative studies. Many studies in healthcare resilience are qualitative in nature. Sections on randomisation are irrelevant for this topic.
Critical Appraisal Skills Programme (CASP) checklist [10]	Deals with systematic reviews mainly. This precludes many of the reviews within healthcare resilience.
Quality Assessment of Diverse Studies (QuADS)[11]	Targeted at empirical studies. Many components are difficult to apply to reviews.
AMSTAR2 tool [12]	Focused on healthcare interventions, centred around (population, intervention, control group and outcome) PICO components

Appendix 1.2: Table of critical appraisal tool examined and why they were deemed unsuitable for this study.

For peer review only

Appendix 2: Definitions - word cloud and word occurrence table

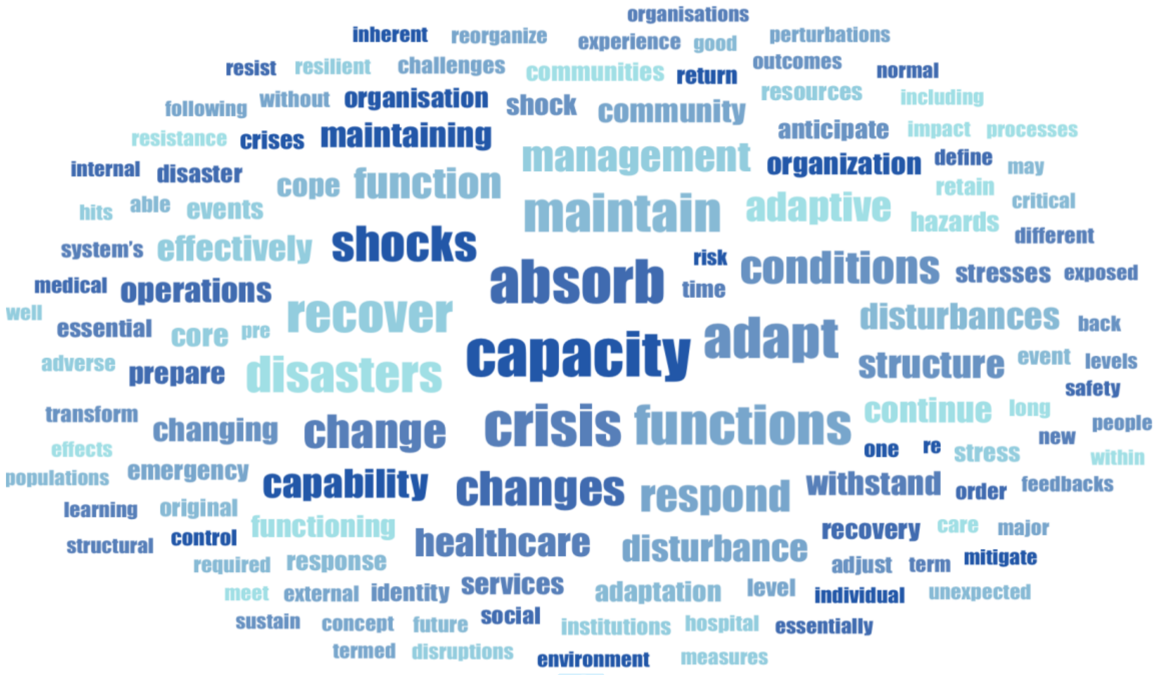

Appendix 2.1: Word occurrence cloud of definitions. Larger words represent increased frequency.

Healthcare Resilience Definition: Word occurrences by timeframe

Pre-event		Intra-event		Post-event		Referent events	
Word/Synonym	Frequency	Word/Synonym	Frequency	Word/Synonym	Frequency	Word/Synonym	Frequency
Prepare	17	Absorb	40	Recovery	42	Crisis	53
Anticipate	10	Adapt	64	Return	15	Shocks/Impacts	41
Expect	5	Capabilities/ Capacities	83	Learning	11	Changes	51
Resource	9	Respond	34	Improvement	9	Disturbance/ Disruption	34
		Maintain	61	Transform	19	Disaster	37
		Essential services	33	Future	7	Events	19
		Essential functions	64			Adversity	12
		Manage	23			Challenges	8
		Mitigate	23			Emergencies	10
		Resist	13			Hazards	11
		Withstand	13				
		Adjust	8				
		Cope	12				

Appendix 2.2: Word occurrence table.

Appendix 3: Data extraction table from reviews

Author, Year Country of 1st author	Title of study	Hierarchical level	Research paradigm	Geographic focus	Study design	Brief summary
**Alami et al., 2021 [13] Canada**	How Can Health Systems Better Prepare for the Next Pandemic? Lessons Learned From the Management of COVID-19 in Quebec (Canada)	Macro	Public health	Quebec, Canada	Narrative review and survey	Took a capacity-focused approach to resilience. Concepts include: Insufficient workforce in several settings, need to ensure training, safety and protection, misaligned policies, shortage of equipment and drugs, ethical dilemmas and utilitarianism, importance of high-level communication, managing media communications
**Augustynowicz et al., 2022 [14] Poland**	Resilient Health and the Healthcare System. A Few Introductory Remarks in Times of the COVID-19 Pandemic	Macro	Patient safety, theoretical	International	Narrative review	Describes definition and scope issues. Covers definitions from several paradigms. Lists some of the evolution of thinking in resilience. Includes urban and community resilience which is outside the scope of our review
**Ayanore et al., 2019 [15] Ghana**	Towards Resilient Health Systems in Sub-Saharan Africa: A Systematic Review of the English Language Literature on Health Workforce, Surveillance, and Health Governance Issues for Health Systems Strengthening	Macro	Global health - HSS	Sub-Saharan Africa	Literature review	Assessed 3 out of 6 elements of WHO building blocks of health in Sub-Saharan Africa. Presents lessons learnt from studies focused mainly on infectious diseases outbreaks and epidemics. Key concepts include leadership and physical resource, including equipment, funding and workforce. Some studies highlight the importance of inter-organisational relationships and effective communication
**Banwell et al., 2018 [16] Australia**	Towards improved linkage of disaster risk reduction and climate change adaptation in health: A review	Meta	Disaster and Climate change adaptation	International	Literature review	Increasing links in literature between DRR and climate change adaptation. Refers to SFDRR. Highlights the need for cross-sectoral work between DRR, climate science and healthcare to build future resilience for health systems
**Barasa et al., 2018 [17] UK**	What is resilience and how can it be nurtured? A systematic review of empirical literature on organizational resilience	Meso/Macro	Global health	International	Literature review	Key concepts: Material resources, preparedness & planning, information management, collateral pathways and redundancies, governance processes, leadership, organisational culture, human capital, social networks and collaboration
**Berg et al., 2018 [18] Norway**	Methodological strategies in resilient health care studies: An integrative review	Meso	Resilience engineering	International	Integrative review	All studies included used qualitative methodology to study resilience. Concepts: individual strategies, sense-making, decision-making, performance variability and expertise

For peer review only

**Biddle et al., 2020 [19] Germany**	Health system resilience: A literature review of empirical research	Macro	Global health	International	Narrative review	Refers to the balance between an outcome-oriented understanding vs a process-oriented understanding of resilience, taken from Blanchet et al. [20]. Public health studies categorised into three groups: (1) Quantitative studies on service delivery, (2) Qualitative studies focusing on HRH, (3) Broader perspectives: building blocks, etc.<br><br>Macro-level Resilience informed by two discourses: disaster management and healthcare quality (patient safety) and resilience engineering.<br>DRR perspectives have shifted after hurricane Catrina in USA toward community resilience
**Blumenstock et al., 2014 [21] USA**	Measuring Preparedness: The National Health Security Preparedness Index	Macro	Public health	USA	Policy document	Presents the National Health Security Preparedness Index:<br>Health surveillance - surveillance and epidemiology investigation, environmental and biological monitoring, laboratory testing<br>Community planning and engagement - cross-sector collaboration, at-risk populations, spontaneous volunteers, social capital and cohesions<br>Incident and information management - multi-agency coordination, emergency public information and warning<br>Surge management - emergency medical services, acute & primary care, mental healthcare<br>Countermeasure management - medical supply chain and distribution, countermeasure utilisation and effectiveness”
**Brand & Jax, 2007 [22] Germany**	Focusing the Meaning(s) of Resilience: Resilience as a Descriptive Concept and a Boundary Object	Meso/Macro	Theoretical	International	Literature review	Focuses on definitions of resilience. 3 categories (descriptive, hybrid and normative). 10 classes and 10 definitions: Original ecological, extended ecological, systematic-heuristic, operational, sociological, ecological-economic, ecosystem-services-related, social-ecological system, metaphoric, sustainability-related.<br><br>Stresses the importance of determining resilience “of what, to what” to operationalise
**Burke et al., 2021 [23] Ireland**	Building health system resilience through policy development in response to COVID-19 in Ireland: From shock to reform	Macro	Public health - COVID	Ireland	Literature review of health policies	States that the Irish government made several changes to increase healthcare access during the COVID19 pandemic. Argues that existing policies/frameworks should not be abandoned in a pandemic, but authorities should focus on those aspects within the used frameworks that contribute to strengthening and dealing with the acute shock. Views resilience as a transformative process in response to crisis
**Carrington et al., 2021 [24] Australia**	The impact of disasters on emergency department resources: review against the Sendai framework for disaster risk reduction 2015–2030	Meso	Global health - Disaster	International	Integrative review	Uses SFDRR to assess emergency departments. Identified points for improvement based on SFDRR framework

For peer review only

**Cartwright et al., 2017** [25] **UK**	The changing health priorities of earthquake response and implications for preparedness: a scoping review	Macro	Global health - DRR	LMICs	Scoping review	Mapped the needs in physical health, mental health and health care to disaster cycle. Categorised according to time frame after the event.<br>Preparedness: disconnect between planning and policy, importance of leadership. Acute phase: women, children and elderly disproportionately affected, trauma to extremities, spinal cord injuries, wound debridement, perinatal issues, lack of specialists, physical equipment and lack of workforce, transport systems, coordination difficulties, lack of integration of information systems and international staff. Response: general health needs, respiratory, ocular, GI and dermatology problems, wound infections, late presentations, cold supply chain failure, increased suicides, staff mental health, importance of leadership and coordination, utilitarianism, surveillance. Chronic phase: rehabilitation, unwanted pregnancies, worsening burden of chronic diseases, PTSD and mental health, psychological support for victims and staff<br><br>Mapped lessons onto disaster management cycle. Plan: Policy, address inequalities, surveillance and long-term monitoring, current service gaps, maintenance of essential services, surge capacity, record systems, international staff, logistics, changing health priorities. Organise and equip: medication, equipment and blood, supply points, transportation, donations. Train: disaster mangement, leadership. Exercise: regular exercises to validate plans and arrangements. Evaluate and improve
**Challen et al., 2012** [26] **UK**	Where is the evidence for emergency planning: A scoping review	Macro	Emergency management	HICs	Scoping review	Makes use of FEMA emergency cycle. Key aspects: Mitigation, hazard analysis, capability assessment, emergency planning, capability maintenance, emergency response, recovery, development plans, communications/mass media, informatics and intelligence, other organisational issues
**Curtis et al., 2017** [27] **UK**	Impact of extreme weather events and climate change for health and social care systems	Macro	Climate	UK	Literature review	Identified need to improve hospital infrastructure, wider social infrastructure and collective responsibility towards resilience approach, cross-sectoral work and integration of planning and preparedness.<br><br>Concepts categorised into three domains: Physical (built infrastructure: health facilities, utilities), Institutional (policies and practices, human resources, professional practice), Social (community groups and informal networks)
**Fallah-Aliabadi et al., 2020** [28] **Iran**	Towards developing a model for the evaluation of hospital disaster resilience: A systematic review	Meso	Disaster management	International	Literature review	Assessed several frameworks for disaster resilience: Hospital Safety Index (HSI), Dynamic approach to seismic resilience of hospitals, Measuring framework of the hospital resilience, Indicators for assessing hospital disaster preparedness in Japan.<br><br>Concepts categorised into domains (with 27 sub-domains): Constructive (stability, design, architectural, transportation and transition system<br>Infrastructural: power, water and sewage, communication and IT, Heating, ventilation and air conditioning (HVAC), fuel, medical gas, equipment and furniture, hazardous material, fire system), Administrative (disaster plan, risk assessment and reduction, response, command, coordination, evacuation, need assessment, logistic and supplies, safety committee, continuity of services, volunteers, finance, recover, training)

For peer review only

**Fridell et al., 2020 [29]<br>Sweden**	Health System Resilience: What Are We Talking About? A Scoping Review Mapping Characteristics and Keywords	Macro	Global health	International	Scoping review + expert consultatio n	Narrative presentation of definitions of resilience.<br><br>Key features of resilience were mapped onto WHO building blocks of health framework: Leadership and governance (ethics, system capacity, operationalisation, engagement, institutional design), information (surveillance, policy making, evaluation), health workforce (diversity and flexibility, limited resources, incentive, community involvement), financing (resource allocation, funding mechanisms, costing), medicines (sustainability), service delivery (response, prevention)
**Grimm et al., 2021 [30]<br>Switzerland**	Enhancing the Understanding of Resilience in Health Systems of Low- and Middle-Income Countries: A Qualitative Evidence Synthesis	Macro	Global health	LMIC	Literature review + interviews	Maps Kruk's Resilience Index [31] onto WHO's building blocks of health [32]. Refined concepts from Resilience Index: Realigned relationships (micro-, meso- and macro-level). Public/private partnerships), foresight and motivation (Investment into slow variables, motivation: political will and individual commitment), change management (integrated change management systems and processes help to confer resilience), emergency preparedness
**Haldane et al., 2020 [33]<br>Canada**	National primary care responses to COVID-19: a rapid review of the literature	Macro	Global health	International	Rapid review	Key lessons from COVID19: Surge capacity should be strengthened. Need to ensure essential services continuity. National guidance needed on biological sampling handling. Most systems increased their telemedicine capabilities
**Hasan et al., 2021 [34]<br>Canada**	Integrated health service delivery during COVID-19: A scoping review of published evidence from low-income and lower-middle-income countries	Macro	Global health (UHC)	LMIC	Scoping review	Assessment of L-LMIC's ability for integrated health service delivery during COVID19 according to WHO building blocks and pandemic continuum.<br><br>Key recommendations: Strengthening of coordination between healthcare bodies at various levels, cross-sectoral collaboration between government, private sector, media, and armed forces. Establishing integrated platforms for testing, health infrastructure to scale-up (redundancies). Empowering communities to engaged in disease prevention and containment. Expansion of digital health technologies. Build resilience by investing in Primary Healthcare and Integrated Health Service Delivery
**Hosseini et al., 2016 [35]<br>USA**	A review of definitions and measures of system resilience	Meso	Engineering	International	Literature review	Presents a narrative of various definitions. Considers differences in organisational, social, economic, and engineering domains. Reliability is often used in engineered systems. There is multidimensionality in some definitions Ongoing issues with definitions include uncertain mechanisms to achieve resilience, the role of recovery, and the need to return to steady-state.<br><br>Presents a summary of how studies measure resilience. Qualitative: conceptual framework, semi-quantitative. Quantitative: generic resilience metrics, structural-based modelling
**Iflaifel et al., 2020 [36]<br>UK**	Resilient Health Care: a systematic review of conceptualisations, study methods and factors that develop resilience	Meso/Macro	Patient safety	International	Literature review	Studies used a mix of qualitative and quantitative methods to study resilience.<br><br>Key concepts: Teamwork, in-situ practical experience, exposure to diverse views and perspectives, trade-offs, protocols & checklists, system design, workarounds

For peer review only

**Jesus et al., 2021** [37] **Inda**	PREparedness, REsponse and SySTemic transformation (PRE-RE-SyST): a model for disability-inclusive pandemic responses and systemic disparities reduction derived from a scoping review and thematic analysis	Macro	Disability studies	International	Scoping review	Focused on inclusive approach to pandemic responses to reduce health inequalities. Key concepts: transform societies' cultural assumptions about disability. Design systems and policies for structural disability-inclusiveness. Prepare inter-sectoral, disability-inclusive pandemic preparedness. Reduce inequalities. Rapid and compassionate responses in residential and long-term care settings. Provide accessibility
**Koeva & Rohova, 2020** [38] **Bulgaria**	Health System Resilience: Concept Development	Micro/Meso/ Macro	Theoretical	International	Literature review	Narrative presentation of quotations of definitions. Dictionaries distinguish between physical objects and innate human capacity. Engineering: resist shocks, absorb and hold elastic strain before permanent distortion or failure. Psychology: copes and maintains function in face of adversity, trauma, tragedy or stress. Resilience as an outcome, a process, or even contains both dynamic and static properties (within community resilience).<br><br>Resilience is undergoing evolution from outcome-oriented to process-oriented. There is a dynamically to the scope, breadth and depth of definitions. Conceptualisations are increasingly broad and complex. Existence of dynamism, uncertainty, non-linearity and unpredictability.
**Kruk et al., 2017** [31] **USA**	Building resilient health systems: a proposal for a resilience index	Macro	Global health	LMICs	Case studies/ review + framework proposal	Defines resilience as "the capacity of health actors, institutions, and populations to prepare for and effectively respond to crises; maintain core functions when a crisis hits; and, informed by lessons learnt during the crisis, reorganise if conditions require it."<br><br>Key domains: aware (capacity, risks and communication), diverse (meets range of needs, adequate financing), self-regulating (maintain core services and leverage collaborations), integrated (coordination and community engagement), adaptive (distributive decision-making, flexible spending and evaluation for improvement)
**Lapão et al., 2015** [39] **Portugal**	Ebola impact on African health systems entails a quest for more international and local resilience: the case of African Portuguese speaking countries	Meso/Macro	Global health	East Africa	Narrative review and case study	Demonstrated fragility of African health systems during Ebola epidemic: increased demand, inequalities and poor access, infrastructure impact, decreased in standards of care of other areas, themes for learning and improvement, critical services need to continue
**Li et al., 2020** [40] **China**	Analysing Healthcare Facility Resilience: Scientometric Review and Knowledge Map	Meso	Global health (UHC)	China	Literature review	Measures tend to be indicator based (set of dimensions and indicators), function based (quantitative evaluation of functionality of healthcare facilities), or structure based (modelling of system structures (elements and relationships) and testing failure).<br><br>Concepts: preparedness (risk assessment, strengthen capacities, HRH, HIS, leadership and governance, coordination), monitoring (surveillance, warning systems, monitoring systems), absorb (physical capacities, past experience, flexible approaches, HIS, communication, recovery and learning (safety1 & 2 approaches, organisational quality improvement strategies)

**Luke et al., 2021** [41] **Australia**	Safer hospital infrastructure assessments for socio-natural disaster - A scoping review	Meso	Disaster management	International	Scoping review	Uses WHO hospital safety index. Key domains: structural (Previous damage and repair, building integrity, condition of building materials, interaction with other buildings/elements, structural redundancies, architectural design, structure design appropriate to location), non-structural (architectural safety, infrastructure protection, access, and physical security, medical equipment and supplies), critical systems (electrical, telecommunications, water, fire protection, waste management, fuel supplies, medical gases, heating/ventilation)
**Meyer et al., 2020** [42] **USA**	A checklist to improve health system resilience to infectious disease outbreaks and natural hazards	Meso/Macro	Global health , Disaster	International	Scoping review + Interview + Checklist workshop	Proposes checklist as a measurement tool or set of indicators for resilience. Items: maintenance of essential health system services and functions, critical infrastructure and transportation, financing, access, communication, collaboration, coordination and partnerships, leadership and command, surge capacity, risk communications, workforce, infection control
**Nuzzo et al., 2019** [43] **USA**	What makes health systems resilient against infectious disease outbreaks and natural hazards? Results from a scoping review	Macro	Global health	International	Scoping review	Resilience attributes: maintenance of essential functions, access to healthcare, maintaining critical infrastructure and transport, crisis financing, leadership and command, collaboration, coordination and partnerships, communication, flexible plans and management structures, legal preparations, surge capacity, crisis standards of care, human resources for health, infection prevention and control, quality improvement, post-event recovery (psychological health of staff, revision of plans, rebuilding social cohesion and trust).<br><br>Themes are consistent with the Resilience Index. Additional findings: developing crisis standards of care, plan for post-event recovery, commitment to quality improvement to ensure lessons learned.
**Oppenheim et al., 2019** [44] **USA**	Assessing global preparedness for the next pandemic: development and application of an Epidemic Preparedness Index	Macro	Global health	International	Framework proposal and evaluation	Epidemic Preparedness Index: public health infrastructure (surveillance, immunisation, medical workforce, hospital capacity, coordination), physical infrastructure (water and sanitation, roads, phones, internet, logistics), institutional capacity (political stability, corruption, bureaucratic effectiveness, armed conflict, homicide, vital registration), economic resources (government revenue generation, per capita income, gross domestic product, health spending, resource dependency), public health communications (public education, risk communication)
**Penaloza et al., 2020** [45] **Brazil**	A resilience engineering perspective of safety performance measurement systems: A systematic literature review	Meso	Resilience engineering	International	Literature review	Safety performance measurement systems should support real-time monitoring and feedback of everyday performance variability, with the ability to analyse adverse events and excellent practices. Measures should be dynamic and subject to re-evaluation

For peer review only

**Pillay & Morel, 2020** [46] **Australia**	Measuring Resilience Engineering: An Integrative Review and Framework for Bench-Marking Organisational Safety	Meso	Resilience engineering	International	Integrative review	"Resilience Engineering is a perspective for organisational safety management which enables organisational members to actively anticipate, respond, monitor and learn; by adapting to operate at the boundary of safe operations by narrowing the gap between work as imagined and work as performed; and manifested in an organisation's culture, cognition and behaviours"<br><br>Argues the need to establish boundaries of safe performance, balance trade-offs between production and safety, and dealing with managed vs prescribed safety. Shifts resilience culture towards resilient climate, acknowledging environmental conditions necessary for resilience.<br><br>Key indicators of resilience: learning culture, flexibility, top management commitment, awareness, preparedness, just culture
**Rameshshanker et al., 2021** [47] **Canada**	Health system resilience to extreme weather events in Asia-Pacific: a scoping review	Macro/Meta	Disaster, climate change	Asia-Pacific	Literature review	Explored how well health systems were resilient towards adverse weather events. Categorised efforts according to WHO building blocks checklist.<br>Medical workforce was the most frequently mentioned aspect, while health financing was the least.
**Rangachari & Woods, 2020** [48] **USA**	Preserving Organizational Resilience, Patient Safety, and Staff Retention during COVID-19 Requires a Holistic Consideration of the Psychological Safety of Healthcare Workers	Micro/Meso	Psychology, Operational research	International	Narrative review	During COVID19, trust and leadership were key aspects to maintain holistic resiliience of staff, which may improve organisational resilience. Advocates for environment of trst, empowerment and safety to enable patient safety concerns to be brought up
**Ravi et al., 2019** [49] **USA**	Establishing a theoretical foundation for measuring global health security: A scoping review	Macro	Global health - HSS	International	Scoping review	Focused on health security, centred around performance of critical health security systems, biosafety and biosecurity, and public health preparedness. Underscored importance of real-time health information systems.<br><br>Key concepts: measuring global health security requires analysis of existing prevention, detection, and response capacities, as well as efforts to build core public health, healthcare, and biosecurity capacities. Assessments of national programs and efforts to mitigate a critical subset of priority threats could serve as useful proxies for measuring global health security. There are measurable enabling factors that facilitate global health security-strengthening efforts. Measuring global health security requires consideration of the risk environment from which infectious disease threats might emerge
**Rezaei et al., 2018** [50] **Iran**	Hospitals preparedness using WHO guideline: A systematic review and meta-analysis	Meso	Emergency management	International	Systematic review and meta-analysis	Measured compliance to WHO hospital emergency checklist. Domains include command and control, communication, safety and security, triage, surge capacity, continuity of essential services, human resources for health, logistics and supply management, post-disaster recovery

For peer review only

**Son et al., 2020** [51]<br>**USA**	Investigating resilience in emergency management: An integrative review of literature	Meso/Macro	Emergency management	International	Integrative review	Presents a table of collated definitions. Stressed the temporal nature in defining resilience. Some focus on pre-event whilst others focus on intra-event. Expected disruption: some focus on the ability to deal with unexpected events, whilst others do not view the distinction. Means to achieve resilience: allocation of roles and resources, HIS or adjusting procedures.<br><br>Dimensions of resilience in emergency management: collective sense-making (improvisation, honest communication, virtual role systems, attitude of wisdom), team decision making (incident command system, training in generic decision-making protocols), harmonising WAI and WAD, interaction and coordination.<br><br>Tools used to support human operators for resilience: geospatial mapping, event history logs, mobile communication systems, decision support tools
**Tessema et al., 2021** [52]<br>**Australia**	The COVID-19 pandemic and healthcare systems in Africa: a scoping review of preparedness, impact and response	Macro	Global health, COVID	Africa	Scoping review	Used Population, Concept Context (PCC) framework for defining eligibility<br>Measured preparedness, impact and response of health systems<br>Framed within WHO building blocks.<br><br>Key findings: preparedness (limited resources, knowledge gaps, lack of training, limited surge capacity and testing ability, limited health services), Impact (reduced patient flow, miss appointments for chronic services, re-orientation of services deter essential services provision, not all services affected), impact on workforce (stigma, mental health illness), response (provision of guidelines, telehealth, segregation of patient streams)
**Tin et al., 2010** [53]<br>**Myanmar**	An approach to health system strengthening in the Union of Myanmar	Macro	Public health - HSS	Myanmar	Case study/ national review	Prioritised service delivery, programme coordination and human resource management to strengthen Myanmar's health system. Takes a systems thinking approach. Uses Health Systems Strengthening indicators at measures and index to compare several countries.
**Tippong et al., 2021** [54]<br>**UK**	A Review of Applications of Operational Research in Healthcare Coordination in Disaster Management	Meso/Macro	Emergency and Disaster management	International	Literature review	Measured coordination effectiveness in healthcare systems. Various staffing patterns have trade-offs: financial cost vs sustainable pattern. Triage and minimisation of beds needed. Coordination between healthcare-network is important for surge management and adaptive capacity.
**Turenne et al., 2019** [55]<br>**France**	Conceptual analysis of health systems resilience: A scoping review	Macro	Theoretical	International	Scoping review	Presented a collated table of definitions and word frequency table. Concepts are dependent on contexts (different groups and populations)<br>In the face of changes. Argues that resilience thinking may be multi-sectoral, but health systems resilience is primarily concerned with the functions of the health system

For peer review only

**Turner et al., 2021 [56] Colombia**	Systematic review of experiences and perceptions of key actors and organisations at multiple levels within health systems internationally in responding to COVID-19	Micro/Meso/ Macro	Global health - COVID	International	Narrative review	Individual level: staff needed to adapt to new working conditions, burnout and psychological distress: work intensity, staff shortages, fear of contagion and transmission, positive adaptation: Telehealth, community healthcare workers, creative approaches<br><br>Organisational level: COVID as catalyst for service change, need to provide psychological support for staff, open management style during crisis<br><br>Local health system level: continuous training to support preparedness for future pandemics, the need to look at working conditions, the need to coordinate diverse actors (community and primary health etc), review and update regulations
**Wiig et al., 2020 [57] Norway**	Defining the boundaries and operational concepts of resilience in the resilience in healthcare research programme	Meso	Patient safety	International	Debate and review	Centred around engineering, psychological and ecological perspectives: Resilience of what, to what, through what. Focused around high quality healthcare, centred around set a capacities, based on events which provide challenges, changes or disruptions. Grounded in multi-level adaptation, enhancement and reorganisation, indicating diversity, coordination and collaboration and is open-ended
**Xin & Xu, 2012 [58] China**	Hospital emergency management research in China: trends and challenges	Macro	Emergency management	China	Narrative review	Defines crisis based on disruptions to hospital systems: patient surge, provision of services, reputation, financial trouble, or major incident. Noted a move from normal vs abnormal crisis to all-hazards. Comprehensive management system may be more useful than temporary command and control, or professional response team. Identified current issues with Chinese medical systems based on WHO building blocks
**Zhong et al., 2014 [59] China**	Development of hospital disaster resilience: conceptual framework and potential measurement	Macro	Global health - Disaster	International	Literature review	Presented a collated list of definitions from different disciplines. Most measures focus on structure, but hardly on process and outcome (based on Donebedian categories of quality of care). Propose measures as a framework: Hospital safety and vulnerability - surveillance, risk & safety. Disaster preparedness and resources - Leadership, cooperation and communication, logistics & stockpiles, workforce, training & drills. Continuity of essential services - surge capacity, emergency medicines. Recovery and adaptation - evaluation, adaptation and recovery capability.<br><br>Factors conferring resilient healthcare: Accessibility, Critical services, Leadership, Integration with community

Legend: WHO = World Health Organisation. DRR = Disaster Risk Reduction. USA = United States of America. HRH = Human Resources for Health. L-LMIC = Low and Low-Middle Income Countries. PTSD = Post-Traumatic Stress Disorder. SFDRR = Sendai Framework for Disaster Risk Reduction. HIS = Health Information Systems

For peer review only

For peer review only

Appendix 4: Summary characteristics of selected resilience frameworks

Framework (reference)	Key components or indicators or principles	Hierarchical focus	Paradigm	Pre-event	Intra-event	Post-event
Resilience Potentials [60]	Anticipate,<br>Monitor,<br>Respond,<br>Learn	Meso	Engineering			
Independent EM concepts [51]	Collective sense-making,<br>Team decision-making,<br>Harmonising WAI/WAD,<br>Interaction and Coordination	Meso	Emergency Management			
FEMA adapted emergency management cycle [26]	Mitigation, hazard and capacity assessment<br>Planning<br>Information systems<br>Continuity of services<br>Emergency response<br>Communications<br>Recovery	Meso	Emergency Management			
WHO Hospital Safety Index (HSI) [41]	Structural (e.g. building integrity, redundancies, previous damage)<br>Non-structural (e.g. critical systems, medical equipment and supplies)<br>Disaster management system	Meso	Healthcare			
WHO hospital disaster preparedness [46,50]	Command and Control<br>Communication<br>Safety and Security<br>Triage<br>Surge capacity<br>Continuity of essential services<br>HRH<br>Logistics and supply management<br>Post-disaster recovery	Meso	Healthcare			
Hospital disaster resilience model [28]	3 domains with 27 sub-domains:<br>Constructive (stability, design, architectural, transportation)<br>Infrastructural (power, water/sewage, communication/IT, HVAC, fuel, medical gas, equipment<br>Administrative (disaster plan, command, coordination, needs assessment, logistics and supplies, safety committee, continuity of services, volunteers, finance, recovery, training)	Meso	Healthcare			
Independent hospital and healthcare resilience [59]	4 principles:<br>Robustness<br>Redundancy<br>Rapidity<br>Resourcefulness<br>4 cross cutting domains:<br>Vulnerability and safety<br>Recovery and adaptation<br>Continuity of essential services<br>Preparedness and resources	Meso/Macro	Healthcare			
Independent disaster preparedness cycle [25]	5 parts of cycle further divided into components, all underpinned by leadership & coordination:<br>Plan<br>Organise & equip<br>Train<br>Exercise	Meso/Macro	Disaster Management			

For peer review only

	Evaluate & improve					
**Concepts conferring resilience** [17]	Material resources<br>Preparedness & planning<br>Information management<br>Collateral pathways and redundancies<br>Governance processes<br>Leadership<br>Organisational culture<br>Human capital<br>Social networks and collaboration	Meso/Macro	Healthcare			
**Primary Care Framework for pandemics (from H1N1)** [33]	Clinical service delivery,<br>Public health functions,<br>Primary care facility operational level<br>Health system level	Meso/Macro	Healthcare			
**NHS Emergency Preparedness, Resilience and Response (EPRR) framework**	Preparedness & anticipation<br>Continuity<br>Subsidiary: decisions at lowest level<br>Communication<br>Cooperation & integration<br>Direction	Macro	Healthcare			
**SFDRR** [61]	Reduce Disaster Mortality<br>Reduce the Number of Affected People<br>Reduce Direct Disaster Economic Loss<br>Reduce Disaster Damage to Critical Infrastructure and Disruption of Basic Services<br>Increase the Number of Countries with National and Sub-National/Local Disaster Risk Reduction Strategies<br>Enhance International Cooperation<br>Increase the Availability of and Access to Multi-Hazard Early Warning Systems	Macro	Disaster Management			
**Global Health Security Index** [62]	Prevent (pathogens)<br>Detect<br>Respond<br>Healthcare (formal)<br>Norms<br>Risk	Macro	Public health			
**Epidemic Preparedness Index** [44]	Public health infrastructure: surveillance, immunisation, medical workforce, hospital capacity, coordination<br>Physical infrastructure: water and sanitation, roads, phones, internet, logistics<br>Institutional capacity: political stability, corruption, bureaucratic effectiveness, armed conflict, homicide, vital registration<br>Economic resources: government revenue generation, per capita income, gross domestic product, health spending, resource dependency<br>Public health communications: public education, risk communication	Macro	Public health			
**European Primary Care Monitoring** [63]	Structure: Governance, economic conditions, workforce<br>Process: access, comprehensiveness, coordination and continuity<br>Outcome: Quality, efficiency, equity	Macro	Healthcare			

For peer review only

**USA Public Health Infrastructure** [64]	Assessment: monitor health, diagnose/investigate<br>Policy development: Inform/educate/empower, community partnerships, develop policies<br>Assurance: Enforce laws, link care, competent workforce, evaluate	Macro	Public health			
**WHO building blocks** [32]	Leadership & governance,<br>Health Information Systems (HIS),<br>Human Resources for Health (HRH),<br>Financing,<br>Service delivery,<br>Essential medicines and technologies	Macro	Healthcare			
**National Health Security Preparedness Index (NHSPI)** [21]	Health surveillance: surveillance and epidemiology investigation, environmental and biological monitoring, laboratory testing<br>Community planning and engagement: cross-sector collaboration, at-risk populations, spontaneous volunteers, social capital and cohesions<br>Incident and information management: multi-agency coordination, emergency public information and warning<br>Surge management: emergency medical services, acute & primary care, mental healthcare<br>Countermeasure management: medical supply chain and distribution, countermeasure utilisation and effectiveness	Macro	Healthcare			
**Integrated Health Service Delivery** [34]	Clinical,<br>Service,<br>Organisational,<br>Functional	Macro	Healthcare			
**Emerging Infectious Diseases Preparedness** [65]	Hardware: Surveillance, infrastructure & medical supplies, workforce, communication mechanisms<br>Software: Governance, trust	Macro	Healthcare			
**Myanmar Health Systems Strengthening Strategy** [53]	Service delivery<br>Programme coordination<br>Human resources	Macro	Healthcare			
**Stockholm Resilience Centre 7 Principles** [66]	Foster complex adaptive systems thinking,<br>Maintain diversity & redundancies,<br>Broaden Participation,<br>Manage connectivity,<br>Manage slow variables and feedback,<br>Encourage learning,<br>Polycentric governance	Macro/Meta	Socioecological systems			
**Refined conceptual models of health system resilience** [30 43]	Foundations of resilience added onto the Resilience Index:<br>Realigned relationships,<br>Foresight and motivation,<br>Change management,<br>Emergency preparedness<br>Crisis standards of care<br>Recovery plans<br>Learning and improvement	Micro/Meso/Macro	Healthcare			
**Health Systems Resilience Index** [31]	Aware: capacity, risks and communication<br>Diverse: meets range of needs, adequate financing<br>Self-regulating: maintain core services and leverage collaborations<br>Integrated: coordination and community engagement<br>Adaptive: distributive decision-making, flexible spending and evaluation for improvement	Meso/Macro/Meta	Healthcare			

Legend: Frameworks are categorised according to predominant hierarchical

focus and paradigm. They are colour-coded according to which time domain they cover.

Abbreviations: EM = Emergency Management. WAI/WAD = Work-As-Imagined vs Work-As-Done. FEMA = Federal Emergency Management Agency. WHO = World Health Organisation. HRH = Human Resources for Health. IT = Information Technology. HVAC = Heating, Ventilation and Air-Conditioning. NHS = UK's National Health Service. SFDRR = Sendai Framework for Disaster Risk Reduction. USA = United States of America.

For peer review only

References

1. Xiao Y, Watson M. Guidance on Conducting a Systematic Literature Review. *Journal of Planning Education and Research* 2019;39(1):93-112. doi: 10.1177/0739456x17723971
2. Greenhalgh T, Robert G, Macfarlane F, et al. Storylines of research in diffusion of innovation: a meta-narrative approach to systematic review. *Soc Sci Med* 2005;61(2):417-30. doi: 10.1016/j.socscimed.2004.12.001 [published Online First: 2005/05/17]
3. Gough D. Meta-narrative and realist reviews: guidance, rules, publication standards and quality appraisal. *BMC Medicine* 2013;11(1):22. doi: 10.1186/1741-7015-11-22
4. Smith V, Devane D, Begley CM, et al. Methodology in conducting a systematic review of systematic reviews of healthcare interventions. *BMC Med Res Methodol* 2011;11(1):15. doi: 10.1186/1471-2288-11-15 [published Online First: 20110203]
5. Becker L, Oxman A. Overviews of reviews. In: Higgins J, Green S, eds. Cochrane handbook for systematic reviews of interventions. Oxford: The Cochrane Collaboration 2010.
6. Wong G, Greenhalgh T, Westhorp G, et al. RAMESES publication standards: meta-narrative reviews. *BMC Medicine* 2013;11(1):20. doi: 10.1186/1741-7015-11-20
7. Higgins JP TJ, Chandler J, Cumpston M, Li T, Page MJ, Welch VA, editor(s). Cochrane Handbook for Systematic Reviews of Interventions Version 6.2 (updated February 2021): Cochrane; 2021 [Available from: training.cochrane.org/handbook accessed Feb 2022.
8. Hannes K. Supplementary Guidance for Inclusion of Qualitative Research in Cochrane Systematic Reviews of Interventions. Version 1 (updated August 2011). In: Noyes J BA, Hannes K, Harden A, Harris J, Lewin S, Lockwood C ed.: Cochrane Collaboration Qualitative Methods Group 2011.
9. HONG QN, PLUYE P, FÀBREGUES S, et al. MIXED METHODS APPRAISAL TOOL (MMAT): McGill University; 2018 [Available from: http://mixedmethodsappraisaltoolpublic.pbworks.com/w/file/fetch/127916259/MMAT_2018_criteria-manual_2018-08-01_ENG.pdf accessed Jan 2022.
10. CASP. Critical Appraisal Skills Programme - Qualitative Checklist 2018 [Available from: https://casp-uk.net/wp-content/uploads/2018/01/CASP-Qualitative-Checklist-2018.pdf accessed

For peer review only

Feb 2022.
11. Harrison R, Jones B, Gardner P, et al. Quality assessment with diverse studies (QuADS): an appraisal tool for methodological and reporting quality in systematic reviews of mixed- or multi-method studies. *BMC Health Services Research* 2021;21(1):144. doi: 10.1186/s12913-021-06122-y
12. Shea BJ, Reeves BC, Wells G, et al. AMSTAR 2: a critical appraisal tool for systematic reviews that include randomised or non-randomised studies of healthcare interventions, or both. *BMJ* 2017;358:j4008. doi: 10.1136/bmj.j4008
13. Alami H, Lehoux P, Fleet R, et al. How Can Health Systems Better Prepare for the Next Pandemic? Lessons Learned From the Management of COVID-19 in Quebec (Canada). *Frontiers in public health* 2021;9:671833. doi: 10.3389/fpubh.2021.671833
14. Augustynowicz A, Opolski J, Waszkiewicz M. Resilient Health and the Healthcare System. A Few Introductory Remarks in Times of the COVID-19 Pandemic. *Int J Environ Res Public Health* 2022;19(6) doi: 10.3390/ijerph19063603
15. Ayanore MA, Amuna N, Aviisah M, et al. Towards Resilient Health Systems in Sub-Saharan Africa: A Systematic Review of the English Language Literature on Health Workforce, Surveillance, and Health Governance Issues for Health Systems Strengthening. *Annals of Global Health* 2019;85(1) doi: 10.5334/aogh.2514
16. Banwell N, Rutherford S, Mackey B, et al. Towards improved linkage of disaster risk reduction and climate change adaptation in health: A review. *Int J Environ Res Public Health* 2018;15(4) doi: 10.3390/ijerph15040793
17. Barasa E, Mbau R, Gilson L. What is resilience and how can it be nurtured? A systematic review of empirical literature on organizational resilience. *International Journal of Health Policy and Management* 2018;7(6):491-503. doi: 10.15171/ijhpm.2018.06
18. Berg SH, Akerjordet K, Ekstedt M, et al. Methodological strategies in resilient health care studies: An integrative review. *Safety Science* 2018;110:300-12. doi: 10.1016/j.ssci.2018.08.025
19. Biddle L, Wahedi K, Bozorgmehr K. Health system resilience: A literature review of empirical research. *Health Policy and Planning* 2020;35(8):1084-109. doi: 10.1093/heapol/czaa032
20. Blanchet K, Nam SL, Ramalingam B, et al. Governance and Capacity to Manage Resilience of Health Systems: Towards a New Conceptual Framework. *International Journal of Health Policy and Management*

For peer review only

2017;6(8):431-35. doi: 10.15171/ijhpm.2017.36

21. Blumenstock J, Bakker G, Jarris PE. Measuring Preparedness: The National Health Security Preparedness Index. *Journal of Public Health Management and Practice* 2014;20(3):361-63. doi: 10.1097/phh.0000000000000073
22. Brand FS, Jax K. Focusing the Meaning(s) of Resilience: Resilience as a Descriptive Concept and a Boundary Object. *Ecology and Society* 2007;12(1)
23. Burke S, Parker S, Fleming P, et al. Building health system resilience through policy development in response to COVID-19 in Ireland: From shock to reform. *Lancet Reg Health Eur* 2021;9:100223. doi: 10.1016/j.lanepe.2021.100223 [published Online First: 20211007]
24. Carrington MA, Ranse J, Hammad K. The impact of disasters on emergency department resources: review against the Sendai framework for disaster risk reduction 2015–2030. *Australas Emerg Care* 2021;24(1):55-60. doi: 10.1016/j.auec.2020.09.003
25. Cartwright C, Hall M, Lee ACK. The changing health priorities of earthquake response and implications for preparedness: a scoping review. *Public Health* 2017;150:60-70. doi: 10.1016/j.puhe.2017.04.024
26. Challen K, Lee AC, Booth A, et al. Where is the evidence for emergency planning: A scoping review. *BMC Public Health* 2012;12(1) doi: 10.1186/1471-2458-12-542
27. Curtis S, Fair A, Wistow J, et al. Impact of extreme weather events and climate change for health and social care systems. *Environmental Health: A Global Access Science Source* 2017;16 doi: 10.1186/s12940-017-0324-3
28. Fallah-Aliabadi S, Ostadtaghizadeh A, Ardalan A, et al. Towards developing a model for the evaluation of hospital disaster resilience: A systematic review. *BMC Health Services Research* 2020;20(1) doi: 10.1186/s12913-020-4915-2
29. Fridell M, Edwin S, Schreeb JV, et al. Health system resilience: what are we talking about? A scoping review mapping characteristics and keywords. *International Journal of Health Policy and Management* 2020;9(1):6-16. doi: 10.15171/ijhpm.2019.71
30. Grimm PY, Oliver S, Merten S, et al. Enhancing the Understanding of Resilience in Health Systems of Low- and Middle-Income Countries: A Qualitative Evidence Synthesis. *International Journal of Health Policy and Management* 2021 doi: 10.34172/ijhpm.2020.261
31. Kruk ME, Ling EJ, Bitton A, et al. Building resilient health systems: a proposal for a resilience index. *BMJ* 2017;357:j2323. doi:

For peer review only - http://bmjopen.bmj.com/site/about/guidelines.xhtml

10.1136/bmj.j2323
32. WHO. Key components of a well functioning health system 2010 [Available from: https://www.who.int/healthsystems/EN_HSSkeycomponents.pdf accessed Nov 2019.
33. Haldane V, Zhang Z, Abbas RF, et al. National primary care responses to COVID-19: a rapid review of the literature. *BMJ Open* 2020;10(12):e041622. doi: 10.1136/bmjopen-2020-041622 [published Online First: 20201208]
34. Hasan MZ, Neill R, Das P, et al. Integrated health service delivery during COVID-19: A scoping review of published evidence from low-income and lower-middle-income countries. *BMJ Global Health* 2021;6(6) doi: 10.1136/bmjgh-2021-005667
35. Hosseini S, Barker K, Ramirez-Marquez JE. A review of definitions and measures of system resilience. *Reliability Engineering & System Safety* 2016;145:47-61. doi: https://doi.org/10.1016/j.ress.2015.08.006
36. Iflaifel M, Lim RH, Ryan K, et al. Resilient Health Care: a systematic review of conceptualisations, study methods and factors that develop resilience. *BMC Health Serv Res* 2020;20(1):324. doi: 10.1186/s12913-020-05208-3 [published Online First: 2020/04/19]
37. Jesus TS, Kamalakannan S, Bhattacharjya S, et al. PREparedness, REsponse and SySTemic transformation (PRE-RE-SyST): a model for disability-inclusive pandemic responses and systemic disparities reduction derived from a scoping review and thematic analysis. *Int J Equity Health* 2021;20(1):204. doi: 10.1186/s12939-021-01526-y [published Online First: 20210914]
38. Koeva S, Rohova M. Health system resilience: concept development. *Journal of Imab* 2020;26(3):3251-58. doi: 10.5272/jimab.2020263.3251
39. Lapão LV, Silva A, Pereira N, et al. Ebola impact on African health systems entails a quest for more international and local resilience: the case of African Portuguese speaking countries. *Pan Afr Med J* 2015;22 Suppl 1(Suppl 1):15. doi: 10.11694/pamj.supp.2015.22.1.6653 [published Online First: 20151011]
40. Li X, Krumholz HM, Yip W, et al. Quality of primary health care in China: challenges and recommendations. *The Lancet* 2020;395(10239):1802-12. doi: 10.1016/S0140-6736(20)30122-7
41. Luke J, Franklin R, Aitken P, et al. Safer hospital infrastructure assessments for socio-natural disaster - A scoping review. *Prehosp Disaster Med* 2021;36(5):627-35. doi: 10.1017/S1049023X21000650
42. Meyer D, Bishai D, Ravi SJ, et al. A checklist to improve health system resilience to infectious disease outbreaks and natural hazards. *BMJ Glob*

For peer review only - http://bmjopen.bmj.com/site/about/guidelines.xhtml

Health 2020;5(8) doi: 10.1136/bmjgh-2020-002429
43. Nuzzo JB, Meyer D, Snyder M, et al. What makes health systems resilient against infectious disease outbreaks and natural hazards? Results from a scoping review. *BMC Public Health* 2019;19(1) doi: 10.1186/s12889-019-7707-z
44. Oppenheim B, Gallivan M, Madhav NK, et al. Assessing global preparedness for the next pandemic: development and application of an Epidemic Preparedness Index. *BMJ Global Health* 2019;4(1):e001157. doi: 10.1136/bmjgh-2018-001157
45. Penaloza GA, Saurin TA, Formoso CT, et al. A resilience engineering perspective of safety performance measurement systems: A systematic literature review. *Safety Science* 2020;130 doi: 10.1016/j.ssci.2020.104864
46. Pillay M, Morel G. Measuring Resilience Engineering: An Integrative Review and Framework for Bench-Marking Organisational Safety. *Safety* 2020;6(3) doi: 10.3390/safety6030037
47. Rameshshanker V, Wyngaarden S, Lau LL, et al. Health system resilience to extreme weather events in Asia-Pacific: a scoping review. *Clim Dev* 2021;13(10):944-58. doi: 10.1080/17565529.2020.1870425
48. Rangachari P, J LW. Preserving Organizational Resilience, Patient Safety, and Staff Retention during COVID-19 Requires a Holistic Consideration of the Psychological Safety of Healthcare Workers. *Int J Environ Res Public Health* 2020;17(12) doi: 10.3390/ijerph17124267 [published Online First: 2020/06/19]
49. Ravi SJ, Meyer D, Cameron E, et al. Establishing a theoretical foundation for measuring global health security: A scoping review. *BMC Public Health* 2019;19(1) doi: 10.1186/s12889-019-7216-0
50. Rezaei F, Maracy MR, Yarmohammadian MH, et al. Hospitals preparedness using WHO guideline: A systematic review and meta-analysis. *Hong Kong Journal of Emergency Medicine* 2018;25(4):211-22. doi: 10.1177/1024907918760123
51. Son C, Sasangohar F, Neville T, et al. Investigating resilience in emergency management: An integrative review of literature. *Appl Ergon* 2020;87 doi: 10.1016/j.apergo.2020.103114
52. Tessema GA, Kinfu Y, Dachew BA, et al. The COVID-19 pandemic and healthcare systems in Africa: a scoping review of preparedness, impact and response. *Bmj Global Health* 2021;6(12) doi: 10.1136/bmjgh-2021-007179
53. Tin N, Lwin S, Kyaing NN, et al. An approach to health system strengthening in the Union of Myanmar. *Health Policy* 2010;95(2-3):95-

For peer review only

102. doi: 10.1016/j.healthpol.2009.11.013

54. Tippong D, Petrovic S, Akbari V. A Review of Applications of Operational Research in Healthcare Coordination in Disaster Management. *Eur J Oper Res* 2021 doi: 10.1016/j.ejor.2021.10.048 [published Online First: 20211028]

55. Turenne CP, Gautier L, Degroote S, et al. Conceptual analysis of health systems resilience: A scoping review. *Soc Sci Med* 2019;232:168-80. doi: 10.1016/j.socscimed.2019.04.020 [published Online First: 20190430]

56. Turner S, Botero-Tovar N, Herrera MA, et al. Systematic review of experiences and perceptions of key actors and organisations at multiple levels within health systems internationally in responding to COVID-19. *Implementation Science* 2021;16(1) doi: 10.1186/s13012-021-01114-2

57. Wiig S, Aase K, Billett S, et al. Defining the boundaries and operational concepts of resilience in the resilience in healthcare research program. *BMC Health Services Research* 2020;20(1) doi: 10.1186/s12913-020-05224-3

58. Xin YT, Xu KY. Hospital emergency management research in China: trends and challenges. *Emerg Med J* 2012;29(5):353-7. doi: 10.1136/emermed-2011-200512 [published Online First: 20111103]

59. Zhong S, Clark M, Hou X-Y, et al. Development of hospital disaster resilience: conceptual framework and potential measurement. *Emergency Medicine Journal* 2014;31(11):930-U8901. doi: 10.1136/emermed-2012-202282

60. Hollnagel E. Epilogue: RAG–the resilience analysis grid. Resilience engineering in practice: CRC Press 2017:275-96.

61. Calkins J. Moving forward after Sendai: How countries want to use science, evidence and technology for disaster risk reduction. *PLoS Currents* 2015;7(DISASTERS) doi: 10.1371/currents.dis.22247d6293d4109d09794890bcda1878

62. Bell JA, Nuzzo JB. Global Health Security Index: Advancing Collective Action and Accountability Amid Global Crisis: NTI/John Hopkins center for health security; 2021 [Available from: www.GHSIndex.org accessed Mar 2022.

63. Kringos DS, Boerma WGW, Bourgueil Y, et al. The european primary care monitor: structure, process and outcome indicators. *BMC Fam Pract* 2010;11(1):81. doi: 10.1186/1471-2296-11-81

64. Benjamin GC. Ensuring health equity during the COVID-19 pandemic: The role of public health infrastructure. *Revista Panamericana de Salud Publica/Pan American Journal of Public Health* 2020;44 doi: 10.26633/RPSP.2020.70

For peer review only

65. Palagyi A, Marais BJ, Abimbola S, et al. Health system preparedness for emerging infectious diseases: A synthesis of the literature. *Glob Public Health* 2019;14(12):1847-68. doi: 10.1080/17441692.2019.1614645 [published Online First: 20190514]

66. Biggs R, Schlüter M, Schoon ML. Principles for Building Resilience: Cambridge University Press 2015.

For peer review only

PRISMA 2020 Checklist

Section and Topic	Item #	Checklist item	Location where item is reported
**TITLE**			
Title	1	Identify the report as a systematic review.	P1, 4
**ABSTRACT**			
Abstract	2	See the PRISMA 2020 for Abstracts checklist.	P1
**INTRODUCTION**			
Rationale	3	Describe the rationale for the review in the context of existing knowledge.	P4
Objectives	4	Provide an explicit statement of the objective(s) or question(s) the review addresses.	P5
**METHODS**			
Eligibility criteria	5	Specify the inclusion and exclusion criteria for the review and how studies were grouped for the syntheses.	P5
Information sources	6	Specify all databases, registers, websites, organisations, reference lists and other sources searched or consulted to identify studies. Specify the date when each source was last searched or consulted.	P5
Search strategy	7	Present the full search strategies for all databases, registers and websites, including any filters and limits used.	P5
Selection process	8	Specify the methods used to decide whether a study met the inclusion criteria of the review, including how many reviewers screened each record and each report retrieved, whether they worked independently, and if applicable, details of automation tools used in the process.	P6
Data collection process	9	Specify the methods used to collect data from reports, including how many reviewers collected data from each report, whether they worked independently, any processes for obtaining or confirming data from study investigators, and if applicable, details of automation tools used in the process.	P6
Data items	10a	List and define all outcomes for which data were sought. Specify whether all results that were compatible with each outcome domain in each study were sought (e.g. for all measures, time points, analyses), and if not, the methods used to decide which results to collect.	P6, Appendices p5-13
	10b	List and define all other variables for which data were sought (e.g. participant and intervention characteristics, funding sources). Describe any assumptions made about any missing or unclear information.	P6
Study risk of bias assessment	11	Specify the methods used to assess risk of bias in the included studies, including details of the tool(s) used, how many reviewers assessed each study and whether they worked independently, and if applicable, details of automation tools used in the process.	P6
Effect measures	12	Specify for each outcome the effect measure(s) (e.g. risk ratio, mean difference) used in the synthesis or presentation of results.	P6
Synthesis methods	13a	Describe the processes used to decide which studies were eligible for each synthesis (e.g. tabulating the study intervention characteristics and comparing against the planned groups for each synthesis (item #5)).	P6
	13b	Describe any methods required to prepare the data for presentation or synthesis, such as handling of missing summary statistics, or data conversions.	P6
	13c	Describe any methods used to tabulate or visually display results of individual studies and syntheses.	P6 – 12 Appendices p5-13
	13d	Describe any methods used to synthesize results and provide a rationale for the choice(s). If meta-analysis was performed, describe the model(s), method(s) to identify the presence and extent of statistical heterogeneity, and software package(s) used.	P6
	13e	Describe any methods used to explore possible causes of heterogeneity among study results (e.g. subgroup analysis, meta-regression).	N/a
	13f	Describe any sensitivity analyses conducted to assess robustness of the synthesized results.	N/a
Reporting bias	14	Describe any methods used to assess risk of bias due to missing results in a synthesis (arising from reporting biases).	P6

For peer review only - http://bmjopen.bmj.com/site/about/guidelines.xhtml

PRISMA 2020 Checklist

Section and Topic	Item #	Checklist item	Location where item is reported
assessment			
Certainty assessment	15	Describe any methods used to assess certainty (or confidence) in the body of evidence for an outcome.	P6, Appendices
**RESULTS**			
Study selection	16a	Describe the results of the search and selection process, from the number of records identified in the search to the number of studies included in the review, ideally using a flow diagram.	P7-8
	16b	Cite studies that might appear to meet the inclusion criteria, but which were excluded, and explain why they were excluded.	P7
Study characteristics	17	Cite each included study and present its characteristics.	P7-8, Appendices p5-13
Risk of bias in studies	18	Present assessments of risk of bias for each included study.	Appendices p5-13
Results of individual studies	19	For all outcomes, present, for each study: (a) summary statistics for each group (where appropriate) and (b) an effect estimate and its precision (e.g. confidence/credible interval), ideally using structured tables or plots.	Appendices p5-13
Results of syntheses	20a	For each synthesis, briefly summarise the characteristics and risk of bias among contributing studies.	P7, 12-13 Appendices p5-13
	20b	Present results of all statistical syntheses conducted. If meta-analysis was done, present for each the summary estimate and its precision (e.g. confidence/credible interval) and measures of statistical heterogeneity. If comparing groups, describe the direction of the effect.	N/a
	20c	Present results of all investigations of possible causes of heterogeneity among study results.	P7, 12-13 Appendices p5-13
	20d	Present results of all sensitivity analyses conducted to assess the robustness of the synthesized results.	N/a
Reporting biases	21	Present assessments of risk of bias due to missing results (arising from reporting biases) for each synthesis assessed.	P7, 12-13 Appendices p5-13
Certainty of evidence	22	Present assessments of certainty (or confidence) in the body of evidence for each outcome assessed.	N/a
**DISCUSSION**			
Discussion	23a	Provide a general interpretation of the results in the context of other evidence.	P7-14,
	23b	Discuss any limitations of the evidence included in the review.	P14
	23c	Discuss any limitations of the review processes used.	P14
	23d	Discuss implications of the results for practice, policy, and future research.	P12-14,
**OTHER INFORMATION**			
Registration and protocol	24a	Provide registration information for the review, including register name and registration number, or state that the review was not registered.	P5
	24b	Indicate where the review protocol can be accessed, or state that a protocol was not prepared.	P5

For peer review only - http://bmjopen.bmj.com/site/about/guidelines.xhtml

PRISMA 2020 Checklist

Section and Topic	Item #	Checklist item	Location where item is reported
	24c	Describe and explain any amendments to information provided at registration or in the protocol.	P6
Support	25	Describe sources of financial or non-financial support for the review, and the role of the funders or sponsors in the review.	P16
Competing interests	26	Declare any competing interests of review authors.	P16
Availability of data, code and other materials	27	Report which of the following are publicly available and where they can be found: template data collection forms; data extracted from included studies; data used for all analyses; analytic code; any other materials used in the review.	Appendices

From: Page MJ, McKenzie JE, Bossuyt PM, Boutron I, Hoffmann TC, Mulrow CD, et al. The PRISMA 2020 statement: an updated guideline for reporting systematic reviews. BMJ 2021;372:n71. doi: 10.1136/bmj.n71

For more information, visit: http://www.prisma-statement.org/

For peer review only

BMJ Open

Healthcare resilience - A meta-narrative systematic review and synthesis of reviews

Journal:	*BMJ Open*
Manuscript ID	bmjopen-2023-072136.R2
Article Type:	Original research
Date Submitted by the Author:	23-Aug-2023
Complete List of Authors:	Tan, Mark; The University of Manchester,<br>Prager, Gabrielle; Johns Hopkins University<br>McClelland, Andrew; The University of Manchester, Alliance Manchester Business School<br>Dark, Paul; National Institute for Health and Care Research, Clinical Research Network; The University of Manchester, Humanitarian and Conflict Response Institute
**Primary Subject Heading**:	Health services research
Secondary Subject Heading:	Health services research, Qualitative research, Communication, Global health, Health policy
Keywords:	Systematic Review, HEALTH SERVICES ADMINISTRATION & MANAGEMENT, Hospitals, QUALITATIVE RESEARCH, PUBLIC HEALTH

SCHOLARONE™
Manuscripts

I, the Submitting Author has the right to grant and does grant on behalf of all authors of the Work (as defined in the below author licence), an exclusive licence and/or a non-exclusive licence for contributions from authors who are: i) UK Crown employees; ii) where BMJ has agreed a CC-BY licence shall apply, and/or iii) in accordance with the terms applicable for US Federal Government officers or employees acting as part of their official duties; on a worldwide, perpetual, irrevocable, royalty-free basis to BMJ Publishing Group Ltd ("BMJ") its licensees and where the relevant Journal is co-owned by BMJ to the co-owners of the Journal, to publish the Work in this journal and any other BMJ products and to exploit all rights, as set out in our licence.

The Submitting Author accepts and understands that any supply made under these terms is made by BMJ to the Submitting Author unless you are acting as an employee on behalf of your employer or a postgraduate student of an affiliated institution which is paying any applicable article publishing charge ("APC") for Open Access articles. Where the Submitting Author wishes to make the Work available on an Open Access basis (and intends to pay the relevant APC), the terms of reuse of such Open Access shall be governed by a Creative Commons licence – details of these licences and which Creative Commons licence will apply to this Work are set out in our licence referred to above.

Other than as permitted in any relevant BMJ Author's Self Archiving Policies, I confirm this Work has not been accepted for publication elsewhere, is not being considered for publication elsewhere and does not duplicate material already published. I confirm all authors consent to publication of this Work and authorise the granting of this licence.

For peer review only

Healthcare resilience - A meta-narrative systematic review and synthesis of reviews

Abstract

Objectives

The COVID19 pandemic has tested global healthcare resilience. Many countries previously considered "resilient" have performed poorly. Available organisational and system frameworks tend to be context-dependent and focus heavily on physical capacities. This study aims to explore and synthesise evidence about healthcare resilience and present a unified framework for future resilience-building.

Design

Systematic review and synthesis of reviews using a meta-narrative approach.

Setting

Healthcare organisations and systems

Primary and secondary outcome measures

Definitions, concepts, and measures of healthcare resilience. We utilised thematic analysis across included reviews to summarise evidence on healthcare resilience.

Results

The main paradigms within healthcare resilience include global health, disaster risk reduction, emergency management, patient safety and public health. Definitions of healthcare resilience recognise various hierarchical levels: individual (micro), facility or organisation (meso), health system (macro), and planetary or international (meta). There has been a shift from a focus on mainly disasters and crises, to an "all-hazards" approach to resilience. Attempts to measure resilience have met with limited success. We analysed key concepts to build a framework for healthcare resilience containing pre-, intra-, post- and trans-event domains. Alongside, we synthesise a definition which dovetails with our framework.

For peer review only - http://bmjopen.bmj.com/site/about/guidelines.xhtml

Conclusion

Resilience increasingly takes an all-hazards approach and a process-oriented perspective. There is increasing recognition of the relational aspects of resilience. Few frameworks incorporate these, and they are difficult to capture within measurement systems. We need to understand how resilience works across hierarchical levels, and how competing priorities may affect overall resilience. Understanding these will underpin interdisciplinary, cross-sectoral, and multi-level approaches to healthcare resilience for the future.

Strengths and limitations of this study

- This is the first systematic review of reviews done on healthcare resilience, at organisational and system levels.
- A meta-narrative approach is utilised, which is particularly suitable for complex topics spanning across disciplines and hierarchical levels.
- Our analysis and synthesis allow for an inter-disciplinary, cross-sectoral and multi-level framework for healthcare resilience.
- Individual (or micro-level) resilience is not included in this review.
- A review of reviews provides a broad overview but does not encompass all details or knowledge about the topic.

For peer review only

For peer review only - http://bmjopen.bmj.com/site/about/guidelines.xhtml

Main Manuscript Title

Healthcare resilience - A meta-narrative systematic review and synthesis of reviews

1. Background

The COVID19 pandemic has tested the resilience of healthcare across the globe. Previously thriving healthcare systems have struggled with multiple aspects; from hospital capacity (1) to infection control measures (2), healthcare worker burnout (3) to degrees of community trust (4), and from international research cooperation (5) to vaccine tribalism and hesitancy (6). Our previous understanding of healthcare resilience requires re-evaluation.

To practically respond to the whole-of-society challenges in light of COVID19, we need evidence that effectively integrates knowledge across these disciplines. This need has been highlighted by several reviews (7, 8). The meta-narrative approach offers such a method. It has been used to track several disciplines converging within a complex field, and is useful for making sense of such complex, heterogenous, and disparate data across disciplines and sectors (9, 10). It thus forms an interdisciplinary and cross-sectoral approach to healthcare resilience.

Recognition that resilience at individual, organisational and system levels affect each other has resulted in some early multi-level empirical research protocols (11, 12). Yet, many studies tend to focus on single hierarchal levels(13-15), within set paradigms (16, 17). For example, literature on psychological resilience tends to focus on acute interventions during or after crisis (18). These seldom consider wider determinants at organisational or system level which affect individual resilience (3). Similarly, some disaster risk reduction (DRR) frameworks have put little emphasis on recovery after crisis (19), which has been highlighted as an important factor to build resilience after COVID19 (20). Therefore, in addition to interdisciplinary and cross-sectoral evidence, multi-level knowledge must also be consolidated.

Given the sheer volume of empirical literature available on healthcare resilience, it was impractical to review beyond a single hierarchical level. Instead, reviews of reviews are particularly useful for gaining a broad overview of complex topics and systems with many dependencies. For example, it has helped to better understand policy and practice priorities in climate sciences (21), and to unpick COVID19 vaccine hesitancy (22). Both are highly complex and current topics involving multiple sectors, disciplines, and hierarchical levels.

We therefore undertook a systematic review of reviews using a meta-narrative approach to present an overview and critical look at healthcare resilience. It highlights lessons learnt from the COVID19 pandemic and synthesises evidence towards an

For peer review only - http://bmjopen.bmj.com/site/about/guidelines.xhtml

overarching framework. The research aims to:

1. Explore the definitions, measures, and concepts of healthcare resilience.
2. Synthesise evidence from the exploration and analysis towards a broad overview of the topic.
3. Present an interdisciplinary, cross-sectoral, and multi-level framework from analysed data.

2. Methods

Theoretical approach

We undertook a systematic literature review of reviews using a meta-narrative approach (23, 24). The review was registered with PROSPERO (CRD42022314729)(25). We utilised RAMESES guidelines for reporting meta-narrative reviews (24). PRISMA guidelines were followed(26).

The principles of the meta-narrative review are pragmatism, pluralism, historicity, contestation, reflexivity, and peer-review. This approach was adopted for several reasons. Firstly, the field has been informed by many research traditions. Secondly, there exists historical agendas which have shifted the focus of resilience at various hierarchical levels (historicity). Thirdly, there are sometimes contrasting findings about resilience (pluralism and contestation). Fourthly, an interdisciplinary overview may be useful for policymakers, practitioners, and academics (pragmatism). Such an approach helps to extract seemingly heterogenous data (peer review). Finally, the final search strategy was informed by some initial searches and snowball sampling, reflecting a reflexive protocol which was adapted according to findings.

Search strategy

A systematic keyword search was conducted in Scopus (Elsevier), Web of Science (Clarivate), PubMed (NIH), and Global Index Medicus (WHO)(Figure 1). We included alternative spellings and synonyms. Inclusion criteria included reviews related to healthcare organisations and systems. We included records from 2008 to November 2022. The search was performed in January 2022 and repeated in November 2022. 2008 corresponded to the global financial crisis, which resulted in an increase in resilience literature (27). Resilience is dynamic (28). Concepts from earlier events may not possess the same relevance as more recent articles. This is especially the case since COVID19. Reviews which were non-healthcare, focused on individual, psychological or community resilience alone, or concerned with specific diseases outside of epidemic contexts were excluded. We also excluded those focused on infection control measures only (29). There were many reviews on individual psychological resilience, particularly acute interventions to mental health crises.

For peer review only - http://bmjopen.bmj.com/site/about/guidelines.xhtml

While we recognised the importance of individuals that make up organisations and systems, we wanted to focus on how resilience is understood at other hierarchical levels, so we excluded these reviews.

Study selection

Records were managed with Endnote 20 (Clarivate analytics). Two reviewers independently selected papers. This was done for several reasons. Firstly, there was wide heterogeneity of studies and papers. Secondly, some reviews refer to cross-sectoral interactions but didn't deal with healthcare. Thirdly, the quality of reviews varied significantly. As a result, having independent reviewers (peer-review) minimises personal bias and ensures there is agreement about selection criteria (30, 31). Screening was done using a modified two-stage process. Title screening was insufficient as a stage on its own due to lack of information about the actual review, or only using "resilience" as a tangential keyword. Both reviewers thus performed a title/abstract screening independently, followed by abstract/full-text screening and discussion around inclusion and exclusion criteria. Additional reviewers were agreed *a priori* should disagreements between the two primary reviewers occur. The final list of included papers was supplemented by snowball sampling and hand searching. This was done by searching through relevant references from selected reviews and discussions with other interdisciplinary team members. Because of the volume of results (Figure 2), it was impractical to focus on empirical studies, or we would have had to confine the search to a single hierarchical level. Therefore, we included reviews only. This may reduce details and resolution, but it allows a summary of knowledge across several reviews and disciplines (32).

Quality appraisal

Quality appraisal of articles was conducted using the Joanna Briggs Institute (JBI) checklist for systematic reviews (33). While there are no specific quality appraisal tools for systematic reviews of reviews, the JBI checklist formed a best fit compared to several other tools (Appendix 1).

Data extraction and analysis

Using an inductive approach, three-tiered coding of the text was conducted around the three foci of definition, measures, and concepts (34)(Appendix 1). We used Atlas.ti qualitative software and data extraction tables. We examined prominent definitions and how they have been informed by different research disciplines. We then collated definitions from the reviews and analysed the most frequently occurring words (Appendix 2). These results were categorised and synthesised with the concepts of resilience to produce a definition which acknowledges past research, links current knowledge, and spans hierarchical levels. Concepts were coded from reviews as well as from the frameworks presented in papers (Appendix 3).

For peer review only - http://bmjopen.bmj.com/site/about/guidelines.xhtml

Patient and public involvement

There was no patient or public involvement in this research.

3. Results

Four databases yielded 8845 records, of which 1070 were reviews. First-stage screening yielded 319 records, of which 68 were duplicates. 251 records were re-screened and discussed. The remaining 52 records underwent quality appraisal. This resulted in 33 records from the search which were analysed. 5 reviews were excluded for low quality. They lacked details about search strategy, inclusion criteria, data analyses, or indeed clear research questions. Other reviews were not relevant according to inclusion and exclusion criteria. Snowball sampling yielded 12 records for analysis. These include reviews which discussed resilience frameworks. The PRISMA diagram summarises the selection process (Figure 2).

3.1 Definitions of healthcare resilience

Most reviews took their definition of healthcare resilience from previous papers. Three reviews contained lists of definitions from other studies (17, 35, 36). Another three reviews presented narrative collections and analyses of definitions (15, 37, 38).

Resilience tends to be defined based on paradigms within which it operates (36-38). For example, within the engineering paradigm it is concerned with the ability of a structure or material to return to its original state (39). However, within business and ecological systems, it includes the ability for an organisation or system to capitalise or improve after a shock (38, 40, 41). A prominent definition of healthcare resilience is from Hollnagel as "the ability of the health care system (a clinic, a ward, a hospital, a county) to adjust its functioning prior to, during, or following events, and thereby sustain required operations under both expected and unexpected conditions" (42, 43). While this definition continues to be refined, it provides a frame to highlight several aspects which emerged from our word occurrence analysis too.

First is the recognition of different hierarchical levels of the healthcare system (43, 44)(Figure 3). There is consensus of these as micro-level (referring to individual), meso-level (hospital or organisation), macro-level (region or country)(43, 44). Several nuances continue to evade neat classification. For example, the team level does not neatly fit into micro- or meso-levels (45), and a community has been considered both meso- and macro-levels (37, 38). There is also emergence of a meta-level of healthcare resilience. This level focuses mainly on the climate crisis as a determinant of health and how it threatens healthcare resilience and exacerbates inequalities (8, 46-48). This transcends national boundaries and so must be considered on a meta-level.

For peer review only - http://bmjopen.bmj.com/site/about/guidelines.xhtml

Second, resilience spans pre-event, intra-event, and post-event. This appreciates contributions from different paradigms. DRR studies are mostly concerned with pre-event aspects, such as preparing for shocks thereby minimising disturbance to a system (13, 49-53). Emergency Management (EM) studies examine aspects of how organisations adapt during a crisis (intra-event), and how they may learn from it (17, 54). Some definitions do not include recovery, while others emphasise that rapidity of recovery back to steady state is a key feature of resilience (16, 37, 51, 55).

Third, both expected and unexpected events are considered. DRR and EM paradigms are concerned with disasters and unexpected crises, which suggest that resilience is only manifest when a sufficiently large shock is applied to the system (35). Patient safety paradigms seek to minimise unintended harm to patients, whether in crisis situations, or within normal operational stressors in healthcare (7, 36, 38, 56). Recently, even DRR and EM reviews have adopted such an "all-hazards" approach to resilience (1, 48).

Hollnagel's definition of healthcare resilience, though widely used, does not specifically include recovery or review (42). Instead, he refers to these concepts as "learn" within his related Resilience Analysis Grid (RAG) (57). The resilience potentials in RAG are anticipate, respond, monitor, and learn. Many other definitions map well onto Hollnagel's definition and his four potentials. There are benefits in linking up definitions with "potentials" or concepts of resilience. It provides convergence in a complex field, which helps to develop a consistent approach across hierarchical levels. This may promote understanding between policymakers and healthcare practitioners (including leaders and managers). In turn, it may lead to more unified approaches across discipline and hierarchical boundaries.

We performed word occurrence analysis from the included reviews, followed by tiered coding into themes, with iterative refinement based on the analysis of concepts of resilience (Appendix 2). The result is a synthesised definition which complements previous work, spans across disciplines, and dovetails with concepts explored in the next section. Our definition of healthcare resilience is *the ability of healthcare workers, organisations or systems to (a) prepare for and prevent, (b) absorb and adapt to maintain structure and essential functions, (c) recover and review from crises, shocks or stressors* (Figure 4).

3.2 Concepts of healthcare resilience

Concepts may refer to practices or features which promote or hinder resilience, or indicators of resilience. Groups of concepts are often collated in frameworks, or set as best fit into established frameworks (e.g. WHO building blocks of health).

For peer review only - http://bmjopen.bmj.com/site/about/guidelines.xhtml

Frameworks are useful to systematically consider various aspects of healthcare resilience (48, 58). We highlight key concepts at macro- and meso-levels, paying attention to lessons learnt from the COVID19 pandemic. We then present our framework of concepts learnt from this review (Figure 5), which dovetail with our definition (Figure 4).

Physical capacities

Within the DRR and EM paradigms, strengthening of existing capacities feature most prominently. This includes adequate workforce (59), equipment (44, 60), financial resources (4), and services (4, 59) to ensure maintenance of essential functions during a crisis. From the COVID19 pandemic, appropriate distribution of physical capacities during various stages of a crisis are equally important. For example, changing shift patterns of emergency staff for increased demand intra-event, but redistributing staff to address longer-term care needs post-event (61). This adopts a longitudinal approach to resilience past the acute crisis and helps to address predicted shortages. As such, it may then prevent mere shifting of crisis from one organisation to another.

Multi-level approaches

Several reviews have highlighted the multi-level approaches required to build resilience (12, 43, 62-65). A small number of empirical studies are currently underway to understand how this works in practice (11, 66). Multi-level approaches recognise that while previous boundaries help understand specific details about how individual hierarchical levels pursue resilience, they are insufficient in representing the interconnections between levels. As an example, while improvisation and coping were seen as resilient concepts at the micro-level, healthcare workers' ability to continue working was underpinned by the availability of resources and protective equipment provided by the organisation and their leaders (meso-level), as well as recovery efforts introduced after the crisis at meso- and macro-level (4, 44, 60). Further work is needed to better understand the interplay and dynamics between the hierarchical levels.

Recovery and Review

The most appropriate method of quality improvement to ensure learning after a crisis depends on context. It is more important for organisations to demonstrate consistency and commitment to change methodology, rather than any specific tool or method (67). This is termed "review" in our framework. Within the patient safety paradigm, two prominent and complementary concepts are safety1 and safety2 approaches. The safety1 approach attempts to get to the root cause of adverse events in healthcare, while the safety2 approach recognises that success occurs more frequently than adverse events and therefore lessons can be learnt from such practices too (42).

For peer review only - http://bmjopen.bmj.com/site/about/guidelines.xhtml

Recovery is mentioned in DRR/EM literature (16, 55, 68), but less so in patient safety literature. This could be contextual. In DRR/EM, crises tend to be larger in scale and affect more people, organisations, and sectors outside of healthcare. Patient safety tends to focus on teams within clinical emergency scenarios or normal operational circumstances. DRR/EM paradigms therefore require a more concerted recovery effort. Rapidity of recovery has been cited as a feature of resilience, but there is no benchmark. Extent of recovery to previous function has been explored, but this does not appreciate the occasional change in function of a system following a large crisis (14, 37). Several reviews have emphasised the importance of having plans for recovery within resilience frameworks, but do not elaborate on details (44, 59, 60). Therefore, there is still much to understand about recovery, and some recent frameworks summarise key lessons learnt from COVID19 (20).

Relational aspects

The COVID19 pandemic has highlighted the importance of relational aspects of resilience. A key aspect is effective leadership (1, 13, 17, 37, 48, 50, 51, 54, 56, 61-63, 65, 68-73). Collaborative and authentic styles which were visible and approachable seemed most favoured during crises(1, 2, 4, 12, 17, 55, 56, 68, 71, 72, 74, 75). Command-and-control structures have been cited, but these refer more to the flow of information rather than organisation of authority. Consequently, more recent DRR reviews advocate for decisions to be pushed out towards the periphery, enabling more rapid adaptation to change (16, 51, 54, 59).

Networks beyond formal healthcare facilities bolsters resilience through the availability of physical capacities (e.g. financing, staff, equipment and volunteers) (1, 13, 16, 37, 61, 62, 68, 73, 74, 76). It also facilitates adaptation and planning post-event. It also facilitates post-event concepts. For example, COVID19 clinical and vaccine trials depended on international collaboration between many disciplines and industries (5). Recognising the importance of this concept, the recent UK Health and Care Act 2022 specifically focuses on inter-organisational relationships towards the provision of integrated care. Further research will therefore be required to better understand how these relationships work in practice (77-79).

An interdisciplinary, cross-sectoral, and multi-level framework for healthcare resilience

These concepts, along with others which emerged from analysis of the reviews, have been compiled into an interdisciplinary, cross-sectoral, and multi-level framework (Figure 4 & 5, Appendix 3). It spans meso- and macro-levels, which few existing frameworks do. It balances the historical focus on physical capacities with relational aspects of resilience. It incorporates lessons learnt from the COVID19 pandemic. The framework may be used as tool to systematically consider various aspects of organisational and systems resilience.

For peer review only - http://bmjopen.bmj.com/site/about/guidelines.xhtml

The framework is divided into timeframes (pre-, intra-, post-, and trans-event). Within timeframes are themes (prepare and prevent etc). Themes encompass several concepts, and finally, these concepts are further broken down into components (Figure 4 & 5). In Figure 4, the size of faint circles indicates the frequency of concepts extracted from the reviews, which are also represented as n-numbers in Figure 5.

3.3 Measures of resilience

Due to the considerable global interest in healthcare resilience, there have been attempts to develop measures or indicators for resilience. The process of pursuing resilience requires ways to determine progression. Attempts to measure resilience therefore allow comparisons and identification of potential areas for improvement. However, none of the current measures have been validated, and some widely used indices have been shown to be inaccurate during the COVID19 pandemic.

Two examples at the Macro-level are the Global Health Security Index (GHSI) and Epidemic Preparedness Index (EPI). They both focus on infectious diseases outbreaks and contain several domains including public health and healthcare infrastructure (e.g. surveillance capabilities, medical workforce), economic resources, and risk assessment and communication strategies (2, 80). As such, they are focused on the pre-event domain of crises. However, many High-Income Countries (HICs) that scored well on these indices performed very poorly during the COVID19 pandemic (81). Several reasons have been cited, including lack of consideration of existing health inequalities, globalisation, societal connectedness, and a bias towards physical capacities (e.g. number of hospitals or beds)(81). Of note, increased globalisation suggests that "disease control may be only as effective as practices within the poorest performing countries" (81). This supports the need for a meta-level of resilience where global health inequalities affect the healthcare resilience of other nations.

Some reviews attempt to evaluate a country's resilience according to one or more domains of the WHO building blocks of health framework (14, 59, 65, 69, 82). However, the framework was designed as a systematic approach to the funding decisions within healthcare, rather than to evaluate resilience (36).

Meso-level measures tend to stem from DRR/EM paradigms, focused on healthcare facilities. Several reviews have collated evidence from empirical papers centred around earthquakes and natural disasters. Quantitative measures include physical capacities (e.g. number of beds, staff, equipment), physical infrastructure (e.g. structural integrity of buildings)(1), and actuality:potentiality ratios (e.g. adherence to operating protocols)(37, 83). Qualitative indicators include checklists containing several components important during disaster situations (e.g. pre-emptive protocols and procedures, command structures, compliance to structural

For peer review only - http://bmjopen.bmj.com/site/about/guidelines.xhtml

regulations)(16, 68). These qualitative components address the intra-event domain of the resilience framework. By providing measurement tools and checklists, pre-emptive strengthening of intra-event components can help to reduce the impact of crises when they do occur.

In the patient safety paradigm, the Checklist for Assessing Institutional Resilience (CAIR) contains broad domains to help hospitals consider several concepts of resilience (84). It was not designed as a set of measures but may be considered a tool for systematic thinking in patient safety. Similarly, while some have suggested that the work-as-imagined vs work-as-done (WAI/WAD) ratio (or the more general actuality:potentiality ratio) may be used as a way to improve patient safety (by minimising performance variability)(17), others highlighted that this approach may work for some simple processes, but is unlikely to result in favourable patient outcomes in unexpected or complex scenarios (43). For example, adherence to sepsis guidelines (WAI) is audited and associated with overall better outcomes (85), but depends on accurate diagnosis, which is itself difficult. Sepsis is frequently misdiagnosed (WAD), leading to wrong treatments, antimicrobial resistance, and poor outcomes (86, 87). Therefore, WAI/WAD is not an adequate approach to measure resilience. Instead, WAI/WAD should be used as means to understand the balance struck within systems, and a concept with which to frame observations about adaptations in healthcare. This reframes resilience from an outcome-oriented approach to a process-oriented one.

Some multi-level assessments of the resilience of critical infrastructures exist. The Tiered Approach by Linkov et al. highlights three tiers of assessments focusing on relationships between components of a system (88). A key feature of this is the use of multiple tools for assessment at different levels and for different goals. For example, the Functional Resonance Analysis Method (FRAM)(89) is useful for mapping relationships between components, while indices may help to align proposed changes with the overarching goals of the system. Checklists may be useful during a crisis. Modelling can help to predict consequences, audit progress, and address slow variables. As such, our framework (Figure 4 & 5, Appendix 3) and the related appendix 4 provide examples of resilience frameworks mapped onto the time domain of crises. There are contemporary studies which seek to better understand multi-level influences specific to healthcare resilience (78, 79).

There seems to be limited focus on the measurement of post-event and trans-event domains. While quality improvement models tend to be embedded within healthcare organisations and systems, assessment of speed of recovery and robustness of interorganisational relationships rarely feature in assessment tools. Similarly, assessment of culture and leadership is challenging (90).

For peer review only - http://bmjopen.bmj.com/site/about/guidelines.xhtml

4. Discussion

We have employed a relatively new review methodology well-suited for complex topics informed by various research traditions. There is increasing acknowledgement of the need for interdisciplinary, cross-sectoral, and multi-level approaches to healthcare resilience. Yet, its history has been one of multiple research traditions operating within specific paradigms. Global health paradigms dominate the macro-level, while disaster risk reduction and patient safety frame the meso-level.

Definitions of healthcare resilience are increasingly broad. They have evolved from highly contextualised situations towards appreciation of different hierarchical levels and from an outcome-oriented understanding towards one which is more process-oriented. With this evolution, it has become more difficult to characterise failure of resilience. Even though healthcare systems and organisations are affected by a range of stressors, they are inherently resilient as essential societal services. Recognising the interplay between hierarchical levels is vital for building resilience. For example, the Institute for Healthcare Improvement framework for improving joy in work considers meso- and macro-level aspects (e.g. participative management and an environment of psychological safety), that affect micro-level resilience, and thus, explicitly links previously distinct hierarchical levels (91). Such inter-level frameworks rarely feature at the meso- and macro-levels, despite healthcare workers being arguably the most important resources of healthcare systems. Several recent study protocols have been published to better understand multi-level influences in resilience, particularly in the patient safety paradigm(11, 66).

Put another way, resilience concepts at macro-level have tended to focus on physical capacities prior to COVID19. Yet, there is increasing recognition of the importance of relational aspects of resilience, particularly across macro- and meso-level boundaries. Some examples of these within the COVID19 pandemic include the adoption of more open leadership at meso- and macro-levels (60), interorganisational relationships and their realignment (4, 62), cultivating an environment of trust (44, 61), and open communication channels at micro-, meso- and macro-levels (92). Open leadership styles gained more trust and enabled better understanding of WAI/WAD (44). Alongside, the open channels of communication between healthcare workers, managers and in turn national bodies helped with rapid adaptation and identification of acute needs, from equipment and supply issues to psychological support services (60).

While a range of tools and indicators have been developed for physical capacities and infrastructure, it is still not known how best to assess the effectiveness of relational aspects of resilience. In particular, understanding the nature of

For peer review only - http://bmjopen.bmj.com/site/about/guidelines.xhtml

interorganisational relationships seems to stand out as a research priority.

Our framework for healthcare resilience fulfils several needs in this field. First, it brings together knowledge and observations gained from a range of disciplines, across hierarchical levels, and integrates it with lessons learnt from the COVID19 pandemic. It acknowledges and builds upon historical health agendas across geopolitical and financial contexts (Figure 3). Many of these have been centred in low-middle income countries including WHO's building blocks of health, health systems strengthening, and disaster risk reduction agendas (Appendix 3). As such, our framework may be applied to a wide range of health organisations and systems. It suggests that resilience requires simultaneous consideration of a diverse set of components but does not assert a particular component as most important. Second, it emphasises both physical and relational aspects of resilience (Figures 4 & 5). Relational aspects such as cross-sector networks and collaborations feature in global health agendas developed primarily for low-middle income countries but have hitherto received less attention in the indices applied in high-income countries. Thus, different components of the framework may take priority for different systems at different points of time. Finally, it dovetails with our definition and achieves some convergence in a highly heterogenous field.

There are several strengths to this study. Firstly, a review of reviews provides a broad overview of the topic across several geopolitical contexts. There is consolidation of knowledge across a wide range of disciplines and paradigms, enabling wider appreciation of the complexities of the topic and the many ways it has been approached. Secondly, a meta-narrative approach helps to explore historical trends and ongoing debates within a complex field. In addition to the consolidation of knowledge in the previous point, this approach also acknowledges the differences between paradigms and broadens the understanding of resilience (Figure 3, Appendix 4). Thirdly, we have taken a multi-level approach at meso- and macro-levels, which has hitherto been rare. These three points provide a map for healthcare workers and health systems managers to better understand the contexts within which each party operates (3). Fourthly, we have highlighted several lessons learnt from the COVID19 pandemic, which was a shock to entire healthcare systems across the world.

Several limitations persist. We do not focus on the micro-level, even though humans are undoubtedly the most important components of healthcare systems, both as staff and service-users. Empirical studies are excluded. This means that while we were able to maintain breadth, we inadvertently lose resolution. In addition, knowledge continues to be discovered and some will not have made it into reviews yet. Therefore, while this review of reviews is timely, we acknowledge that resilience is a dynamic process (28). Several important empirical studies are underway to unpick key relational factors between hierarchical levels and how they may support or hinder

For peer review only - http://bmjopen.bmj.com/site/about/guidelines.xhtml

adaptive capacity (11, 78). These studies should help to deepen understanding into healthcare resilience. We chose not to include grey literature due to volume of work, but this potentially excludes some important information from this review. Similarly, while the language restriction potentially imposes a selection bias, a significant proportion of included reviewed specifically focused on non-English speaking and/or low-middle income countries. Frameworks can be considered tools for systematically approaching resilience but require adaptation towards individual situations (Appendix 4). Our framework does not provide guidance on which components should be prioritised by which health system at which point of time. This requires contextualised analyses and aligns with the related realist approach seeking to understand "what works, for whom, under what circumstances and how" (24).

Future reviews should state clear research questions, adopt systematic search strategies, and use robust data analyses techniques. These would maximise the quality of evidence generated. Future priorities arising from this research include the need to examine multi-level resilience with empirical studies (11), exploring how each level's resilience contributes to other levels (3), and gaining deeper understanding of the nature and extent of interorganisational relationships and how they affect resilience.

5. Conclusion

Our review of reviews provides a broad overview of healthcare resilience. The meta-narrative approach adopted recognises the contribution from several disciplines and paradigms within this field. We have highlighted the tendency for studies to focus on individual hierarchical levels and limited timescales, but also acknowledge the need and current desire to develop more interdisciplinary, cross-sectoral, and multi-level approaches to healthcare resilience. Our framework for healthcare resilience crosses hierarchical and time boundaries, providing a broad and reflexive overview of healthcare resilience. Understanding the balances between sometimes opposing concepts, and contrasting priorities of different professional groups or hierarchies, is key to building resilience for the future.

For peer review only - http://bmjopen.bmj.com/site/about/guidelines.xhtml

Contributorship statement

Concept – MT, AMcC, PD. Planning and design – MT. Screening – MT, GP. Additional reviewers – AMcC, PD. Data collection, analysis, and interpretation – MT, GP. Writing – MT. Editing – all authors

Competing interests

None declared.

Funding

No funding.

Data sharing statement

All data relevant to the study are included in the article or uploaded as supplementary information.

Ethics Approval

This study does not involve human participants and ethical approval was not required.

For peer review only

For peer review only - http://bmjopen.bmj.com/site/about/guidelines.xhtml

References

1. Barasa E, Mbau R, Gilson L. What Is Resilience and How Can It Be Nurtured? A Systematic Review of Empirical Literature on Organizational Resilience. International Journal of Health Policy and Management. 2018;7(6):491-503.
2. Oppenheim B, Gallivan M, Madhav NK, Brown N, Serhiyenko V, Wolfe ND, et al. Assessing global preparedness for the next pandemic: development and application of an Epidemic Preparedness Index. BMJ Global Health. 2019;4(1):e001157.
3. Tan MZY. Resilience is a dirty word: misunderstood, and how we can truly build it. Critical Care. 2022;26(1):168.
4. Meyer D, Bishai D, Ravi SJ, Rashid H, Mahmood SS, Toner E, et al. A checklist to improve health system resilience to infectious disease outbreaks and natural hazards. BMJ Glob Health. 2020;5(8).
5. Park JJH, Mogg R, Smith GE, Nakimuli-Mpungu E, Jehan F, Rayner CR, et al. How COVID-19 has fundamentally changed clinical research in global health. The Lancet Global Health. 2021;9(5):e711-e20.
6. Razai MS, Chaudhry UAR, Doerholt K, Bauld L, Majeed A. Covid-19 vaccination hesitancy. BMJ. 2021;373:n1138.
7. Pillay M, Morel G. Measuring Resilience Engineering: An Integrative Review and Framework for Bench-Marking Organisational Safety. Safety. 2020;6(3).
8. Curtis S, Fair A, Wistow J, Val DV, Oven K. Impact of extreme weather events and climate change for health and social care systems. Environmental Health: A Global Access Science Source. 2017;16.
9. Greenhalgh T, Robert G, Macfarlane F, Bate P, Kyriakidou O, Peacock R. Storylines of research in diffusion of innovation: a meta-narrative approach to systematic review. Soc Sci Med. 2005;61(2):417-30.
10. Mondal S, Van Belle S, Maioni A. Learning from intersectoral action beyond health: a meta-narrative review. Health Policy Plan. 2021;36(4):552-71.
11. Aase K, Guise V, Billett S, Sollid SJM, Njå O, Røise O, et al. Resilience in Healthcare (RiH): A longitudinal research programme protocol. BMJ Open. 2020;10(10).
12. Wiig S, Aase K, Billett S, Canfield C, Røise O, Njå O, et al. Defining the boundaries and operational concepts of resilience in the resilience in healthcare research program. BMC Health Services Research. 2020;20(1).
13. Li L, Liao S, Yuan J, Wang E, She J. Analyzing Healthcare Facility Resilience: Scientometric Review and Knowledge Map. Frontiers in Public Health. 2021;9.
14. Biddle L, Wahedi K, Bozorgmehr K. Health system resilience: A literature review of empirical research. Health Policy and Planning. 2020;35(8):1084-109.
15. Koeva S, Rohova M. Health system resilience: concept development. Journal of Imab. 2020;26(3):3251-8.
16. Zhong S, Clark M, Hou X-Y, Zang Y-L, Fitzgerald G. Development of hospital disaster resilience: conceptual framework and potential measurement. Emergency Medicine Journal. 2014;31(11):930-U8901.
17. Son C, Sasangohar F, Neville T, Peres SC, Moon J. Investigating resilience in

For peer review only

For peer review only - http://bmjopen.bmj.com/site/about/guidelines.xhtml

emergency management: An integrative review of literature. Appl Ergon. 2020;87.
18. Almedom AM. Resilience research and policy/practice discourse in health, social, behavioral, and environmental sciences over the last ten years. African health sciences. 2008;8 Suppl 1:S5-13.
19. Calkins J. Moving forward after Sendai: How countries want to use science, evidence and technology for disaster risk reduction. PLoS Currents. 2015;7(DISASTERS).
20. McClelland AG, Shaw D, O'Grady N, Fattoum A. Recovery for Development: A Multi-Dimensional, Practice-Oriented Framework for Transformative Change Post-Disaster. The Journal of Development Studies. 2022:1-20.
21. Conti A, Valente M, Paganini M, Farsoni M, Ragazzoni L, Barone-Adesi F. Knowledge Gaps and Research Priorities on the Health Effects of Heatwaves: A Systematic Review of Reviews. Int J Environ Res Public Health. 2022;19(10).
22. Ries M. The COVID-19 Infodemic: Mechanism, Impact, and Counter-Measures—A Review of Reviews. Sustainability. 2022;14(5):2605.
23. Gough D. Meta-narrative and realist reviews: guidance, rules, publication standards and quality appraisal. BMC Medicine. 2013;11(1):22.
24. Wong G, Greenhalgh T, Westhorp G, Buckingham J, Pawson R. RAMESES publication standards: meta-narrative reviews. BMC Medicine. 2013;11(1):20.
25. Tan M, Prager G. Resilience in healthcare: a meta-narrative review of reviews. CRD42022314729: PROSPERO; 2022 [Available from: https://www.crd.york.ac.uk/prospero/display_record.php?ID=CRD42022314729.
26. Page MJ, McKenzie JE, Bossuyt PM, Boutron I, Hoffmann TC, Mulrow CD, et al. The PRISMA 2020 statement: an updated guideline for reporting systematic reviews. BMJ. 2021;372:n71.
27. Gurney A. Resilience in the UK and other OECD economies: Treasury Economic Working Paper No.2 London: HM Treasury; 2008 [Available from: https://webarchive.nationalarchives.gov.uk/ukgwa/20081112160625/http:/www.hm-treasury.gov.uk/d/bud08_workingpaper2_557_.pdf.
28. Bealt J, Powell D, Shaw D. Benefits of involving public involvement in emergency planning Manchester: University of Manchester; 2021 [Available from: https://committees.parliament.uk/writtenevidence/21957/pdf/.
29. Abdullah MA, Shaikh BT. Confusion and denial: need for systems thinking to understand the HIV epidemic in Pakistan. J Ayub Med Coll Abbottabad. 2014;26(3):396-400.
30. Butler A, Hall H, Copnell B. A Guide to Writing a Qualitative Systematic Review Protocol to Enhance Evidence-Based Practice in Nursing and Health Care. Worldviews Evid Based Nurs. 2016;13(3):241-9.
31. Smith V, Devane D, Begley CM, Clarke M. Methodology in conducting a systematic review of systematic reviews of healthcare interventions. BMC Med Res Methodol. 2011;11(1):15.
32. Smith V, Devane D, Begley CM, Clarke M. Methodology in conducting a systematic review of systematic reviews of healthcare interventions. BMC Medical Research Methodology. 2011;11(1):15.
33. Aromataris E, Fernandez R, Godfrey C, Holly C, Kahlil H, Tungpunkom P. Summarizing systenatic reviews: methodological development, conduct and reporting

For peer review only - http://bmjopen.bmj.com/site/about/guidelines.xhtml

of an Umbrella review approach. . Int J Evid Based Healthc. 2015;13:132-40.
34. Noble H, Mitchell G. What is grounded theory? Evidence Based Nursing. 2016;19(2):34-5.
35. Brand FS, Jax K. Focusing the Meaning(s) of Resilience: Resilience as a Descriptive Concept and a Boundary Object. Ecology and Society. 2007;12(1).
36. Turenne CP, Gautier L, Degroote S, Guillard E, Chabrol F, Ridde V. Conceptual analysis of health systems resilience: A scoping review. Soc Sci Med. 2019;232:168-80.
37. Hosseini S, Barker K, Ramirez-Marquez JE. A review of definitions and measures of system resilience. Reliability Engineering & System Safety. 2016;145:47-61.
38. Augustynowicz A, Opolski J, Waszkiewicz M. Resilient Health and the Healthcare System. A Few Introductory Remarks in Times of the COVID-19 Pandemic. Int J Environ Res Public Health. 2022;19(6).
39. Zhong S, Clark M, Hou X-Y, Zang Y-L, Fitzgerald G. Proposing and developing a definition and conceptual framework for health care resilience to cope with disasters. Emergencias. 2014;26(1):69-77.
40. Biggs R, Schlüter M, Schoon ML. Principles for Building Resilience: Cambridge University Press; 2015.
41. Florez-Jimenez M, Muñoz-Villamizar A, Lleo A. Exploring the relationship between sustainability, resilience, and purpose in the context of corporations: a comprehensive literature review SSRN; 2021 [Available from: https://papers.ssrn.com/sol3/papers.cfm?abstract_id=3944148.
42. Hollnagel E, Wears R, Braithwaite J. From Safety-I to Safety-II: A White Paper. 2015.
43. Berg SH, Akerjordet K, Ekstedt M, Aase K. Methodological strategies in resilient health care studies: An integrative review. Safety Science. 2018;110:300-12.
44. Rangachari P, J LW. Preserving Organizational Resilience, Patient Safety, and Staff Retention during COVID-19 Requires a Holistic Consideration of the Psychological Safety of Healthcare Workers. Int J Environ Res Public Health. 2020;17(12).
45. Stoverink AC, Kirkman BL, Mistry S, Rosen B. Bouncing Back Together: Toward a Theoretical Model of Work Team Resilience. Academy of Management Review. 2020;45(2):395-422.
46. Ayuningtyas D, Windiarti S, Sapoan Hadi M, Fasrini UU, Barinda S. Disaster preparedness and mitigation in indonesia: A narrative review. Iranian Journal of Public Health. 2021;50(8):1536-46.
47. Romanello M, McGushin A, Di Napoli C, Drummond P, Hughes N, Jamart L, et al. The 2021 report of the Lancet Countdown on health and climate change: code red for a healthy future. The Lancet. 2021;398(10311):1619-62.
48. Kruk ME, Ling EJ, Bitton A, Cammett M, Cavanaugh K, Chopra M, et al. Building resilient health systems: a proposal for a resilience index. BMJ. 2017;357:j2323.
49. Carrington MA, Ranse J, Hammad K. The impact of disasters on emergency department resources: review against the Sendai framework for disaster risk reduction 2015–2030. Australas Emerg Care. 2021;24(1):55-60.
50. Cartwright C, Hall M, Lee ACK. The changing health priorities of earthquake

For peer review only - http://bmjopen.bmj.com/site/about/guidelines.xhtml

response and implications for preparedness: a scoping review. Public Health. 2017;150:60-70.
51. Challen K, Lee AC, Booth A, Gardois P, Woods HB, Goodacre SW. Where is the evidence for emergency planning: A scoping review. BMC Public Health. 2012;12(1).
52. Fallah-Aliabadi S, Ostadtaghizadeh A, Ardalan A, Fatemi F, Khazai B, Mirjalili MR. Towards developing a model for the evaluation of hospital disaster resilience: A systematic review. BMC Health Services Research. 2020;20(1).
53. Luke J, Franklin R, Aitken P, Dyson J. Safer hospital infrastructure assessments for socio-natural disaster - A scoping review. Prehosp Disaster Med. 2021;36(5):627-35.
54. Xin YT, Xu KY. Hospital emergency management research in China: trends and challenges. Emerg Med J. 2012;29(5):353-7.
55. Nuzzo JB, Meyer D, Snyder M, Ravi SJ, Lapascu A, Souleles J, et al. What makes health systems resilient against infectious disease outbreaks and natural hazards? Results from a scoping review. BMC Public Health. 2019;19(1).
56. Iflaifel M, Lim RH, Ryan K, Crowley C. Resilient Health Care: a systematic review of conceptualisations, study methods and factors that develop resilience. BMC Health Serv Res. 2020;20(1):324.
57. Hollnagel E. Epilogue: RAG–the resilience analysis grid. Resilience engineering in practice: CRC Press; 2017. p. 275-96.
58. WHO. Monitoring the building blocks of health systems 2010 [
59. Tessema GA, Kinfu Y, Dachew BA, Tesema AG, Assefa Y, Alene KA, et al. The COVID-19 pandemic and healthcare systems in Africa: a scoping review of preparedness, impact and response. Bmj Global Health. 2021;6(12).
60. Turner S, Botero-Tovar N, Herrera MA, Borda Kuhlmann JP, Ortiz F, Ramirez JC, et al. Systematic review of experiences and perceptions of key actors and organisations at multiple levels within health systems internationally in responding to COVID-19. Implementation Science. 2021;16(1).
61. Alami H, Lehoux P, Fleet R, Fortin JP, Liu J, Attieh R, et al. How Can Health Systems Better Prepare for the Next Pandemic? Lessons Learned From the Management of COVID-19 in Quebec (Canada). Frontiers in public health. 2021;9:671833.
62. Grimm PY, Oliver S, Merten S, Han WW, Wyss K. Enhancing the Understanding of Resilience in Health Systems of Low- and Middle-Income Countries: A Qualitative Evidence Synthesis. International Journal of Health Policy and Management. 2021.
63. Kamara JK, Akombi BJ, Agho K, Renzaho AMN. Resilience to climate-induced disasters and its overall relationship to well-being in southern africa: A mixed-methods systematic review. Int J Environ Res Public Health. 2018;15(11).
64. Tippong D, Petrovic S, Akbari V. A Review of Applications of Operational Research in Healthcare Coordination in Disaster Management. Eur J Oper Res. 2021.
65. Van Ryneveld M, Schneider H, Lehmann U. Looking back to look forward: a review of human resources for health governance in South Africa from 1994 to 2018. Human Resources for Health. 2020;18(1).
66. Anderson JE, Aase K, Bal R, Bourrier M, Braithwaite J, Nakajima K, et al. Multilevel influences on resilient healthcare in six countries: An international comparative study protocol. BMJ Open. 2020;10(12).

For peer review only - http://bmjopen.bmj.com/site/about/guidelines.xhtml

67. HealthFoundation. Quality improvement made simple: What everyone should know about health care quality improvement: The Health Foundation; 2021 [Available from: https://www.health.org.uk/sites/default/files/QualityImprovementMadeSimple.pdf.
68. Rezaei F, Maracy MR, Yarmohammadian MH, Sheikhbardsiri H. Hospitals preparedness using WHO guideline: A systematic review and meta-analysis. Hong Kong Journal of Emergency Medicine. 2018;25(4):211-22.
69. Ayanore MA, Amuna N, Aviisah M, Awolu A, Kipo-Sunyehzi DD, Mogre V, et al. Towards Resilient Health Systems in Sub-Saharan Africa: A Systematic Review of the English Language Literature on Health Workforce, Surveillance, and Health Governance Issues for Health Systems Strengthening. Annals of Global Health. 2019;85(1).
70. Hasan MZ, Neill R, Das P, Venugopal V, Arora D, Bishai D, et al. Integrated health service delivery during COVID-19: A scoping review of published evidence from low-income and lower-middle-income countries. BMJ Global Health. 2021;6(6).
71. Ravi SJ, Meyer D, Cameron E, Nalabandian M, Pervaiz B, Nuzzo JB. Establishing a theoretical foundation for measuring global health security: A scoping review. BMC Public Health. 2019;19(1).
72. Fridell M, Edwin S, Schreeb JV, Saulnier DD. Health system resilience: what are we talking about? A scoping review mapping characteristics and keywords. International Journal of Health Policy and Management. 2020;9(1):6-16.
73. Blumenstock J, Bakker G, Jarris PE. Measuring Preparedness: The National Health Security Preparedness Index. Journal of Public Health Management and Practice. 2014;20(3):361-3.
74. Haldane V, Zhang Z, Abbas RF, Dodd W, Lau LL, Kidd MR, et al. National primary care responses to COVID-19: a rapid review of the literature. BMJ Open. 2020;10(12):e041622.
75. Lapão LV, Silva A, Pereira N, Vasconcelos P, Conceição C. Ebola impact on African health systems entails a quest for more international and local resilience: the case of African Portuguese speaking countries. Pan Afr Med J. 2015;22 Suppl 1(Suppl 1):15.
76. Tin N, Lwin S, Kyaing NN, Htay TT, Grundy J, Skold M, et al. An approach to health system strengthening in the Union of Myanmar. Health Policy. 2010;95(2-3):95-102.
77. Sanderson M, Allen P, Osipovic D, Boiko O, Lorne C. The Developing Architecture of System Management: Integrated Care Systems and Sustainability and Transformation Partnerships - Interim report: PRUComm; 2021 [Available from: https://prucomm.ac.uk/assets/uploads/PRUComm_ICS_study_interim_report_feb_2021.pdf.
78. Anderson JE, Aase K, Bal R, Bourrier M, Braithwaite J, Nakajima K, et al. Multilevel influences on resilient healthcare in six countries: an international comparative study protocol. BMJ Open. 2020;10(12):e039158.
79. Petersen EE, Lyng HB, Ree E, Wiig S. Relationship between management and resilience in healthcare: A study protocol for a systematic review. BMJ Open. 2021;11(7).
80. Bell JA, Nuzzo JB. Global Health Security Index: Advancing Collective Action

For peer review only - http://bmjopen.bmj.com/site/about/guidelines.xhtml

and Accountability Amid Global Crisis: NTI/John Hopkins center for health security; 2021 [Available from: www.GHSIndex.org.
81. Baum F, Freeman T, Musolino C, Abramovitz M, De Ceukelaire W, Flavel J, et al. Explaining covid-19 performance: what factors might predict national responses? BMJ. 2021;372:n91.
82. Rameshshanker V, Wyngaarden S, Lau LL, Dodd W. Health system resilience to extreme weather events in Asia-Pacific: a scoping review. Clim Dev. 2021;13(10):944-58.
83. Penaloza GA, Saurin TA, Formoso CT, Herrera IA. A resilience engineering perspective of safety performance measurement systems: A systematic literature review. Safety Science. 2020;130.
84. Carthey J, de Leval MR, Reason JT. Institutional resilience in healthcare systems. Quality in Health Care. 2001;10(1):29-32.
85. Evans L, Rhodes A, Alhazzani W, Antonelli M, Coopersmith CM, French C, et al. Surviving sepsis campaign: international guidelines for management of sepsis and septic shock 2021. Intensive Care Med. 2021;47(11):1181-247.
86. Fitzpatrick F, Tarrant C, Hamilton V, Kiernan FM, Jenkins D, Krockow EM. Sepsis and antimicrobial stewardship: two sides of the same coin. BMJ Quality & Safety. 2019;28(9):758-61.
87. Vincent JL. The Clinical Challenge of Sepsis Identification and Monitoring. PLoS Med. 2016;13(5):e1002022.
88. Linkov I, Trump BD, Trump J, Pescaroli G, Hynes W, Mavrodieva A, et al. Resilience stress testing for critical infrastructure. Int J Disaster Risk Reduct. 2022;82:103323.
89. Patriarca R, Di Gravio G, Woltjer R, Costantino F, Praetorius G, Ferreira P, et al. Framing the FRAM: A literature review on the functional resonance analysis method. Safety Science. 2020;129:104827.
90. Njah J, Hansoti B, Adeyami A, Bruce K, O'Malley G, Gugerty MK, et al. Measuring for Success: Evaluating Leadership Training Programs for Sustainable Impact. Ann Glob Health. 2021;87(1):63.
91. Smith AF, Plunkett E. People, systems and safety: resilience and excellence in healthcare practice. Anaesthesia. 2019;74(4):508-17.
92. Raymond CB, Ward PR. Community-level experiences, understandings, and responses to COVID-19 in low-and middle-income countries: A systematic review of qualitative and ethnographic studies. Int J Environ Res Public Health. 2021;18(22).

For peer review only - http://bmjopen.bmj.com/site/about/guidelines.xhtml

Figure captions

Figure 1: Search strategy

Figure 2: PRISMA diagram

Figure 3: Healthcare resilience: hierarchical levels and selected paradigm timelines with historical highlights. Legend: WHO = World Health Organisation. DRR: Disaster Risk Reduction. FEMA: Federal Emergency Management Agency. GHSI: Global Health Security Index.

Figure 4: An interdisciplinary, cross-sectoral, and multi-level definition and conceptual framework for healthcare resilience. *Legend: Defining features are listed as headings within the pre-, intra-, post- and trans-event time domains.*

Figure 5: Details of the interdisciplinary, cross-sectoral, and multi-level framework for healthcare resilience. Concepts are presented with frequency of occurrences as n-numbers. They are further divided into components, along with example considerations extracted from reviews.

For peer review only

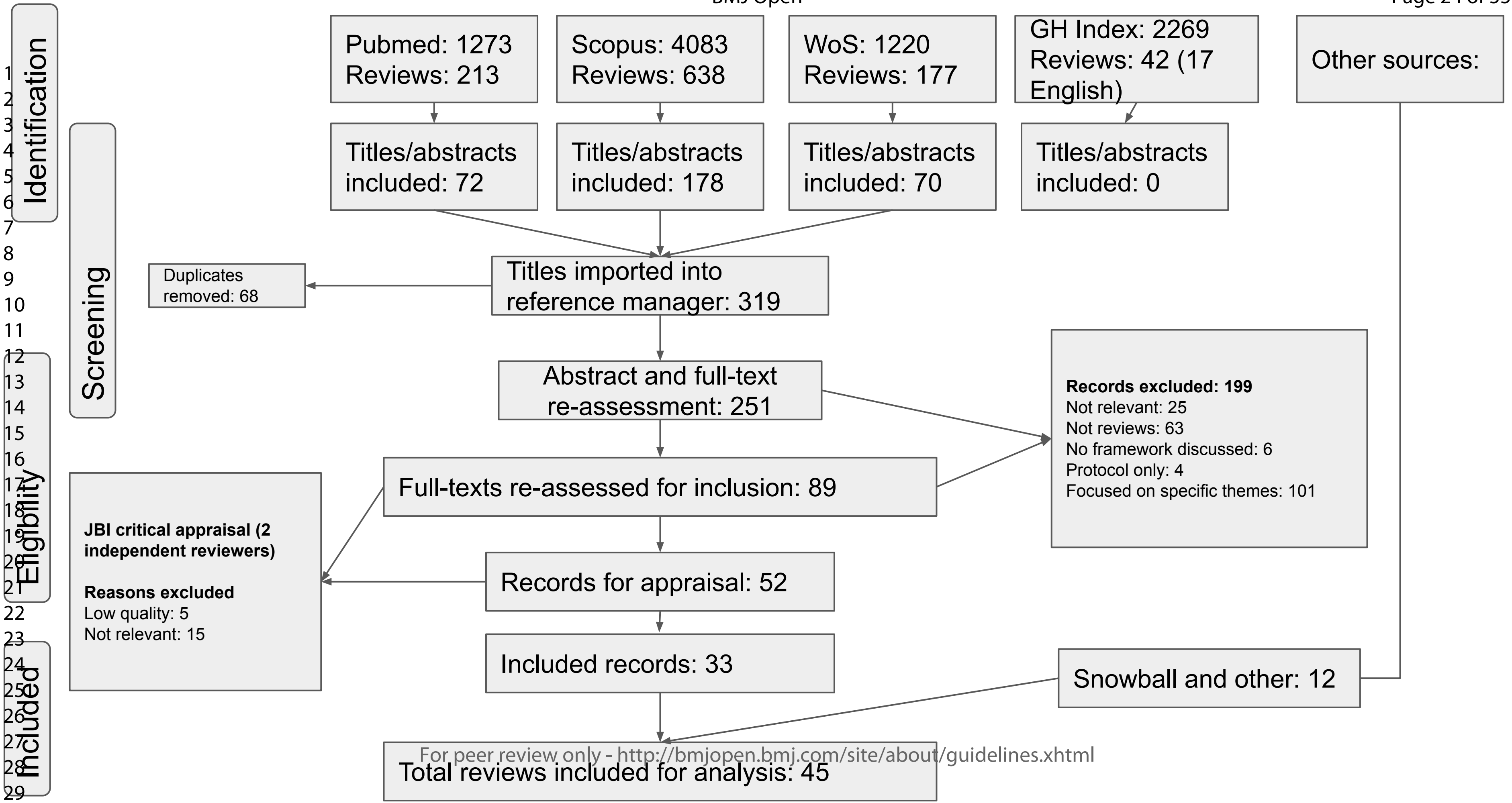
Identification
Screening
Eligibility
Included
Pubmed: 1273
Reviews: 213
Scopus: 4083
Reviews: 638
WoS: 1220
Reviews: 177
GH Index: 2269
Reviews: 42 (17 English)
Other sources:
Titles/abstracts included: 72
Titles/abstracts included: 178
Titles/abstracts included: 70
Titles/abstracts included: 0
Duplicates removed: 68
Titles imported into reference manager: 319
Abstract and full-text re-assessment: 251
Records excluded: 199
Not relevant: 25
Not reviews: 63
No framework discussed: 6
Protocol only: 4
Focused on specific themes: 101
Full-texts re-assessed for inclusion: 89
JBI critical appraisal (2 independent reviewers)
Reasons excluded
Low quality: 5
Not relevant: 15
Records for appraisal: 52
Included records: 33
Snowball and other: 12
Total reviews included for analysis: 45

Search Strategy

Scopus (Elsevier)

(TITLE-ABS-KEY ("resilien*"OR"strengthening") AND TITLE-ABS-KEY ("system"OR"organi*ation") AND TITLE-ABS-KEY ("health"OR"hospital"OR"healthcare") AND TITLE-ABS-KEY ("crisis"OR"disaster"OR“epidemic”OR“pandemic”))

Web of Science (Clarivate)

("resilience" OR "resilient" OR "strengthen") AND ("health") AND ("organisation" OR "systems") AND ("crisis" OR "disaster" OR "epidemic" OR "pandemic”)

PubMed (NIH)

((("resilien*"OR"strengthening") AND ("system"OR"organisation")) AND (health)) AND ("crisis"OR"disaster"OR"epidemic"OR"pandemic”)

Global Index Medicus (WHO)

(tw:(Resilien* OR strengthening)) AND (tw:(System OR Organisation))

Inclusion Criteria

- 2008 - 2022
- Scholarly articles (reviews) published in peer-reviewed academic databases
- Related to healthcare organisations and systems

Exclusion Criteria

- Non-healthcare
- Focused on individual, psychological or community resilience
- Concerned with a particular disease or health condition outside of epidemic/pandemic context
- Non-English

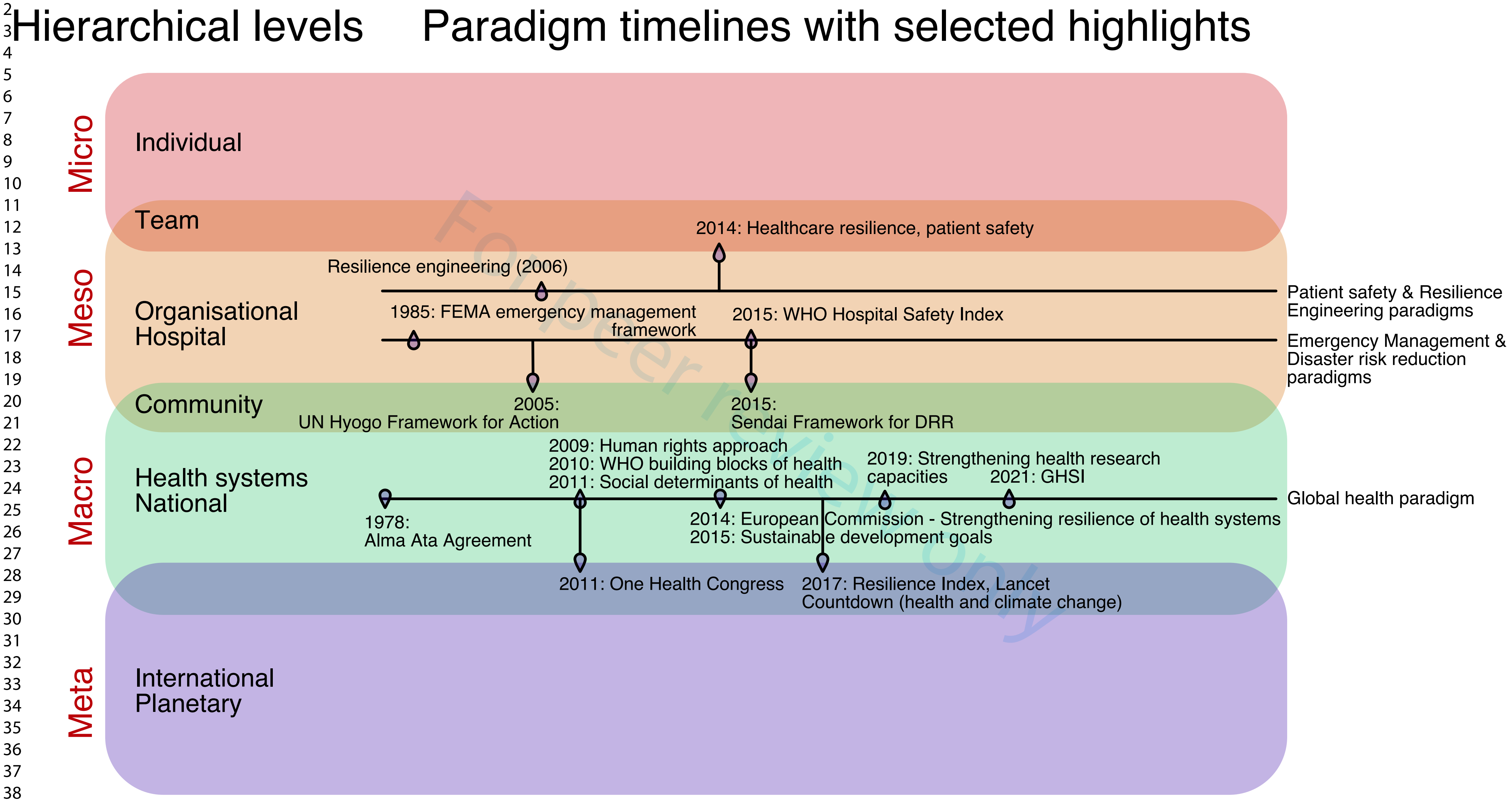
Hierarchical levels
Paradigm timelines with selected highlights
Micro
Individual
Team
Meso
Organisational
Hospital
Community
Macro
Health systems
National
Meta
International
Planetary
2014: Healthcare resilience, patient safety
Resilience engineering (2006)
Patient safety & Resilience Engineering paradigms
1985: FEMA emergency management framework
2015: WHO Hospital Safety Index
Emergency Management & Disaster risk reduction paradigms
2005:
UN Hyogo Framework for Action
2015:
Sendai Framework for DRR
2009: Human rights approach
2010: WHO building blocks of health
2011: Social determinants of health
2019: Strengthening health research capacities
2021: GHSI
Global health paradigm
1978:
Alma Ata Agreement
2014: European Commission - Strengthening resilience of health systems
2015: Sustainable development goals
2011: One Health Congress
2017: Resilience Index, Lancet Countdown (health and climate change)

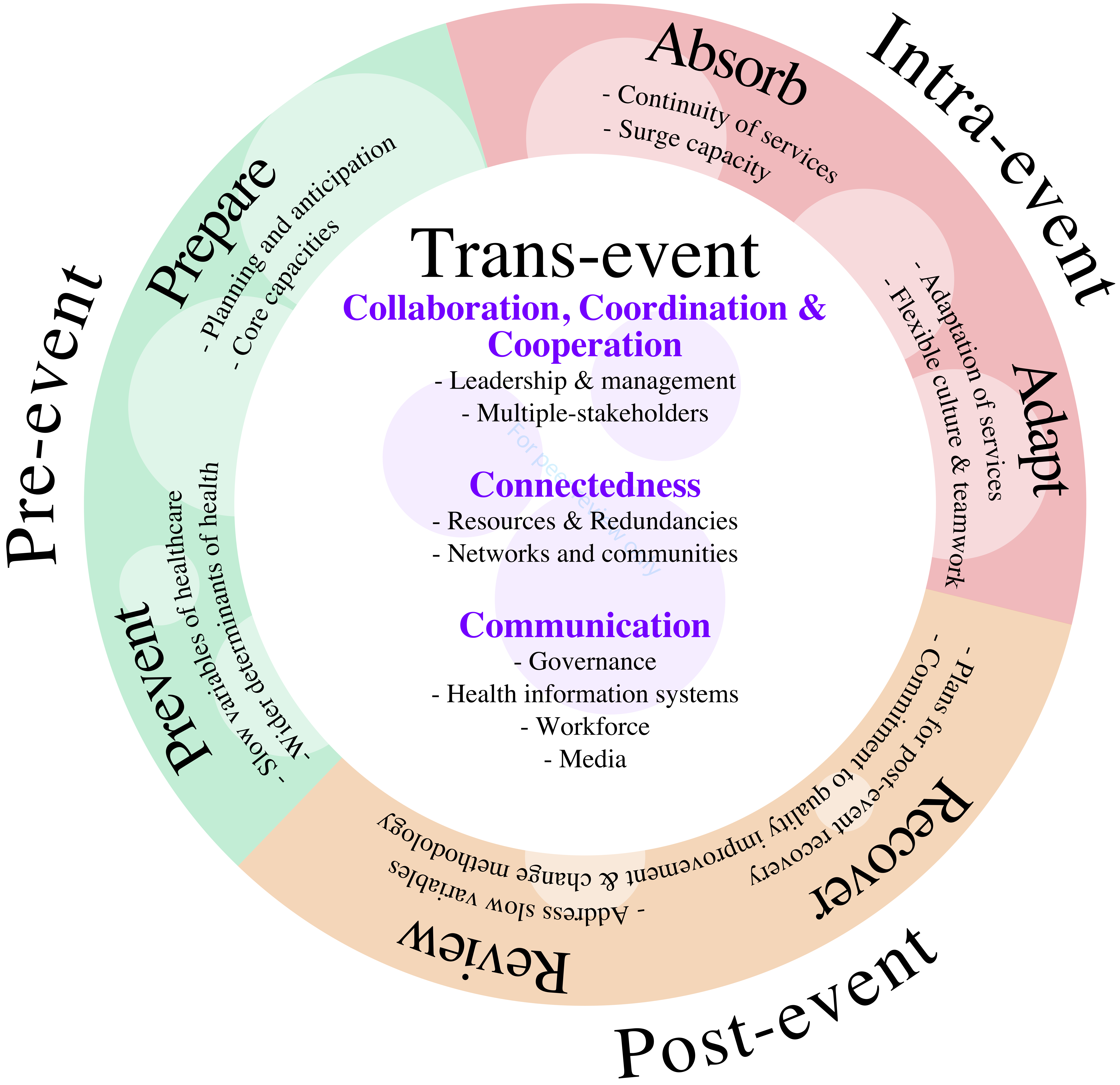

For peer review only

Interdisciplinary, Cross-sectoral, Multi-level framework for Healthcare Resilience

	Themes	Concepts (frequency)	Component	Example considerations
Pre-event	Prevent	Slow variables (n=8)	Healthcare access and range of services	Universal healthcare, geographical access factors, equality in access
			Acting on risks identified	Health inequalities, at risk populations
			Strengthening healthcare infrastructure	Ventilation and air-conditioning systems, integrity of buildings, additional cliincal spaces
			Healthcare expenditure and funding channels	WHO Building Blocks of Health
			Staff retention and education	From COVID19: burnout, wellbeing, development opportunities. Plan for attrition.
		Wider determinants (n=10)	Social determinants of health	Water, sanitation, education, employment
			Health inequalities	From COVID19: disproportionate health outcomes for ethnic minorities and lower socioeconomic groups
			Physical and critical infrastructure	Road and access, buildings and public services
			Environmental considerations	One Health movement, planetary health
	Prepare	Planning & anticipation (n=12)	Disaster or crisis protocols and policies	Sendai Framework for Disaster Risk Reduction, FEMA emergency management cycle
			Strengthen community and long-term care	From COVID19: lack of workers in care settings after initial surge
			Modelling disasters	
			Strengthen interorganisational networks	Transport and supply chains, voluntary sectors, faith-based organisations
			Training and maintenance of skills	Disaster and major incident training, simulation and drills.
		Core capacities (n=11)	Beds, staff, equipment, medicines	Existing resources and emergency stockpiles
			Plans for utilising surge capacities	Equipment, spaces, alternative facilities
Intra-event	Absorb	Continuity of services (n=19)	Maintain essential services	Maternity, paediatrics, emergency medicine, intensive care
			Planning and involving spontaneous volunteers	ISO 22319:2017
			Mass-casualty triage protocols	Royal College of Emergency Medicine guidance
			Ensure access to healthcare services	Consider hard-to-reach populations
		Surge capacity (n=16)	Mobilising core and surge capacities	
			Activating policies and processes for major incidents	
			Donation standards and distribution	
	Adapt	Adaptation of services (n=17)	Temporary reduction in elective services	From COVID19: reduction in volume of services (e.g. USA's Crisis Standards of Care)
			Temporary incident command and communication system	
			Adopting new technologies	From COVID19: telemedicine and videoconferencing
		Flexible culture and teamwork (n=7)	Collective sensemaking and fast variables in healthcare	From COVID19: improvisations and workarounds characterised resilient adaptation.
			Trade-offs, workarounds and improvisation	WAI/WAD. Actuality:Potentiality ratios
Post-event	Recover	Plans for post-event recovery (n=8)	Acute psychological interventions for staff	
			Staff for increased long-term care	Predicted late surge in community, complex and long-term care
			Strategies for community recovery	Recovery for Development Framework. National Consortium for Societal Resilience
	Review	Commitment to quality improvement and change methodology (n=16)	Quality improvement method or framework	Evaluation reports, vulnerability analysis, risk assessments. Safety 1 & 2
			Evaluation and data collection systems	
		Address slow variables (n=8)	Organisational or system adaptation based on lessons learnt	
Trans-event	Collaboration, Coordination, Cooperation	Leadership & management (n=20, 12 respectively)	Effective leadership and trust	Open style leadership during crisis
			Crisis leadership training	
		Multiple stakeholders (n= 3)	Links with surveillance organisations	Meteorological institutes, public services, public health authorities
			Cross-sector collaboration	Patient groups, manufacturers, volunteer and faith-based sector, private, non-profit
			Diverse approaches to support creativiity and innovation	Kruk's Resilience Index, Principles of Socio-ecological resilience, Empirical studies of Uganda's public corporations
	Connectedness	Resources and redundancies (n=22)	Supply and stock management processes	
			Plans for pooling resources	From COVID19: Capacity transfers in intensive care
		Networks and communities (n=16)	Integrated care	UK Health and Care Act 2022
			Dialogue with community leaders & volunteers	
			Regional and international links and diplomatic ties	International emergency response for earthquakes and natural disasters
	Communication	Governance (n=5)	Ensure multiple feedback loops	
			Civil registration and statistics systems	
		Health information systems (n=7)	Multi-level surveillance strategies	Public health functions, routine data collection and dissemination, data sharing and integration, early warning systems
		Workforce (n=17)	Feedback & communication mechanisms across hierarchical levels	Staff forums, incident reporting
			Staff recognition	
		Media (n=3)	Accuracy of information disssemination	From COVID19: false news and vaccine hesitancy
			Population trust in health system	

Supplementary file

Healthcare resilience - A meta-narrative systematic review and synthesis of reviews

For peer review only

List of supplementary materials

Appendix 1: Additional details of methodology
Appendix 2: Definition word cloud and word occurrence table
Appendix 3: Data extraction table from reviews
Appendix 4: Healthcare resilience framework summary

Appendix 1: Additional methodological details

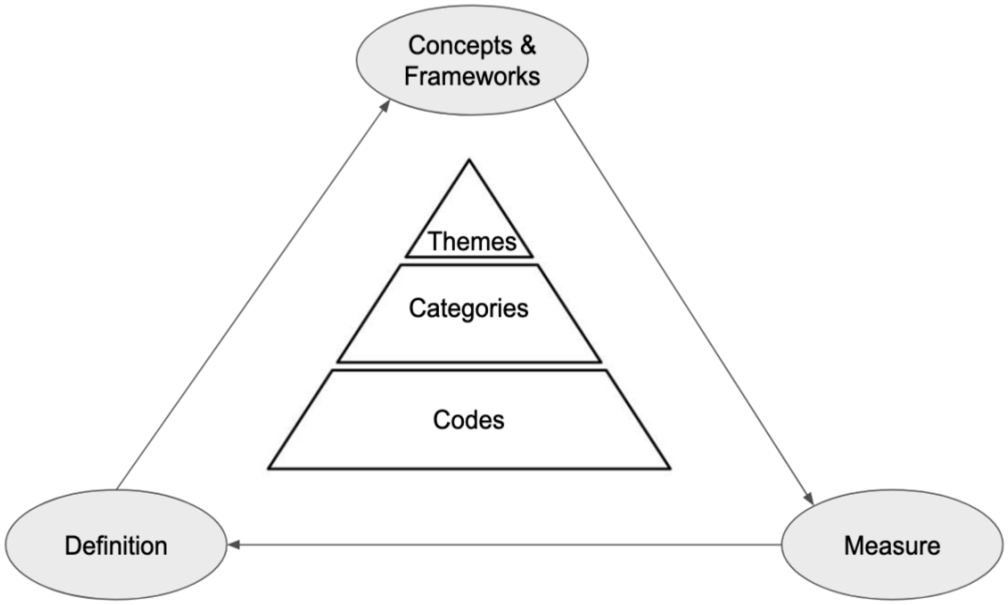

Appendix 1.1: Theoretical framework describing three tiers of analysis within three foci.

In choosing our approach, we referred to Xiao et al.'s typology of reviews [1]. We sought to describe and extend the current understanding of healthcare resilience. Since resilience has been studied within a range of academic disciplines, across multiple sectors and a numerous hierarchies and levels, a meta-narrative was the most appropriate form of review to perform. This aims to consolidate the various research traditions involved in the study of a broad topic, thereby forming an overarching synthesis of the landscape of said topic [2]. A related method is the realist review, which seeks to distil circumstantial conditions conferring success of various interventions [3]. Traditional systematic reviews or meta-analyses were unsuitable methodologies for this study due to several reasons. In particular, studies into resilience include multiple methodologies, both quantitative and qualitative, over a span of disciplines which include evolving terminology and nomenclature. The homogeneity required for a traditional systematic review or quantitative measures which form the basis of a meta-analysis do not feature in resilience studies [4 5].

The key principles of meta-narrative reviews are pragmatism, pluralism,

historicity, contestation, reflexivity and peer review [6]. This review of reviews provides a multidisciplinary and multi-level view of healthcare resilience. It maps current understanding and presents various conceptualisations and frameworks (pluralism). This will thus be useful for a multitude of actors including healthcare leaders, policymakers and academics (pragmatism). Examining the various research traditions which converge in this topic, it will both chart its evolution and explore various perspectives (historicity). Differences in definitions, measures and conceptualisations will be highlighted and discussed (contestation). The protocol is updated according to the findings, and future agendas are proposed in line with emergent data and insights (reflexivity). Finally, independent reviewers are involved throughout the process, while results are shared with a broader research team focusing on whole-of-society resilience (peer review).

Tool or method	Reason for unsuitability
Cochrane protocol [7]	Requires established quantitative metrics
Cochrane qualitative tool [8]	Only applicable for empirical studies. Unsuitable for mixed or multi-method studies
Mixed Methods Assessment Tool (MMAT)[9]	Heavily weighted towards quantitative studies. Many studies in healthcare resilience are qualitative in nature. Sections on randomisation are irrelevant for this topic.
Critical Appraisal Skills Programme (CASP) checklist [10]	Deals with systematic reviews mainly. This precludes many of the reviews within healthcare resilience.
Quality Assessment of Diverse Studies (QuADS)[11]	Targeted at empirical studies. Many components are difficult to apply to reviews.
AMSTAR2 tool [12]	Focused on healthcare interventions, centred around (population, intervention, control group and outcome) PICO components

Appendix 1.2: Table of critical appraisal tool examined and why they were deemed unsuitable for this study.

For peer review only

Appendix 2: Definitions - word cloud and word occurrence table

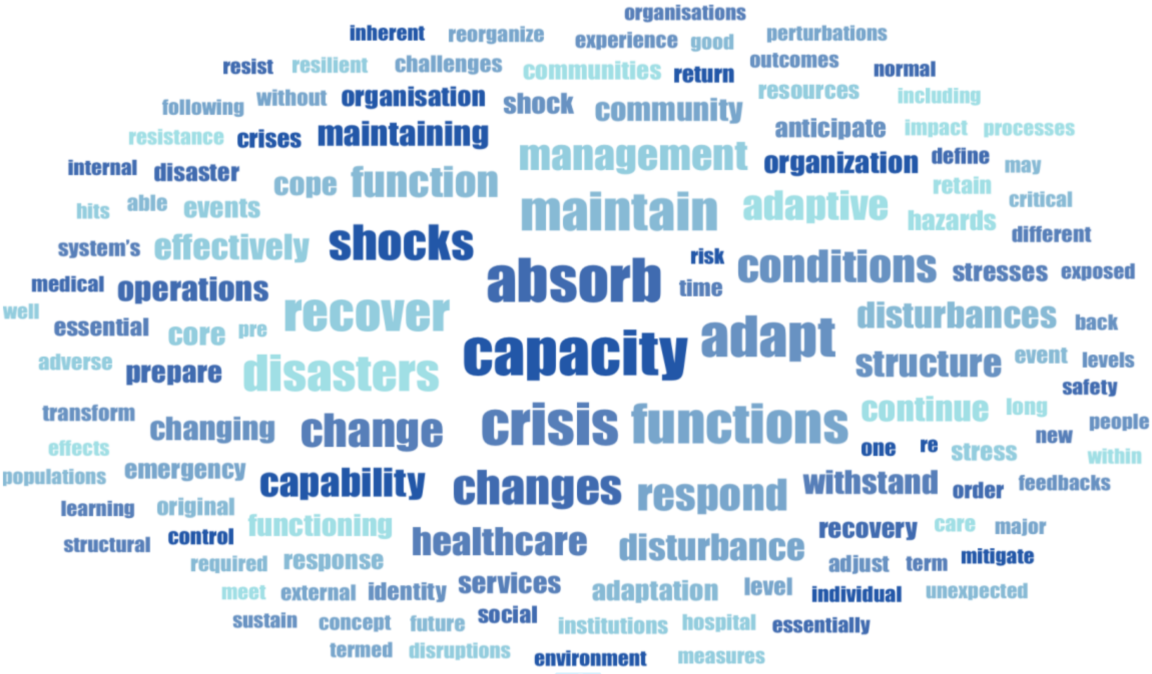

Appendix 2.1: Word occurrence cloud of definitions. Larger words represent increased frequency.

Healthcare Resilience Definition: Word occurrences by timeframe

Pre-event		Intra-event		Post-event		Referent events	
Word/Synonym	Frequency	Word/Synonym	Frequency	Word/Synonym	Frequency	Word/Synonym	Frequency
Prepare	17	Absorb	40	Recovery	42	Crisis	53
Anticipate	10	Adapt	64	Return	15	Shocks/Impacts	41
Expect	5	Capabilities/ Capacities	83	Learning	11	Changes	51
Resource	9	Respond	34	Improvement	9	Disturbance/ Disruption	34
		Maintain	61	Transform	19	Disaster	37
		Essential services	33	Future	7	Events	19
		Essential functions	64			Adversity	12
		Manage	23			Challenges	8
		Mitigate	23			Emergencies	10
		Resist	13			Hazards	11
		Withstand	13				
		Adjust	8				
		Cope	12				

Appendix 2.2: Word occurrence table.

Appendix 3: Data extraction table from reviews

Author, Year Country of 1st author	Title of study	Hierarchical level	Research paradigm	Geographic focus	Study design	Brief summary
**Alami et al., 2021 [13] Canada**	How Can Health Systems Better Prepare for the Next Pandemic? Lessons Learned From the Management of COVID-19 in Quebec (Canada)	Macro	Public health	Quebec, Canada	Narrative review and survey	Took a capacity-focused approach to resilience. Concepts include: Insufficient workforce in several settings, need to ensure training, safety and protection, misaligned policies, shortage of equipment and drugs, ethical dilemmas and utilitarianism, importance of high-level communication, managing media communications
**Augustynowicz et al., 2022 [14] Poland**	Resilient Health and the Healthcare System. A Few Introductory Remarks in Times of the COVID-19 Pandemic	Macro	Patient safety, theoretical	International	Narrative review	Describes definition and scope issues. Covers definitions from several paradigms. Lists some of the evolution of thinking in resilience. Includes urban and community resilience which is outside the scope of our review
**Ayanore et al., 2019 [15] Ghana**	Towards Resilient Health Systems in Sub-Saharan Africa: A Systematic Review of the English Language Literature on Health Workforce, Surveillance, and Health Governance Issues for Health Systems Strengthening	Macro	Global health - HSS	Sub-Saharan Africa	Literature review	Assessed 3 out of 6 elements of WHO building blocks of health in Sub-Saharan Africa. Presents lessons learnt from studies focused mainly on infectious diseases outbreaks and epidemics. Key concepts include leadership and physical resource, including equipment, funding and workforce. Some studies highlight the importance of inter-organisational relationships and effective communication
**Banwell et al., 2018 [16] Australia**	Towards improved linkage of disaster risk reduction and climate change adaptation in health: A review	Meta	Disaster and Climate change adaptation	International	Literature review	Increasing links in literature between DRR and climate change adaptation. Refers to SFDRR. Highlights the need for cross-sectoral work between DRR, climate science and healthcare to build future resilience for health systems
**Barasa et al., 2018 [17] UK**	What is resilience and how can it be nurtured? A systematic review of empirical literature on organizational resilience	Meso/Macro	Global health	International	Literature review	Key concepts: Material resources, preparedness & planning, information management, collateral pathways and redundancies, governance processes, leadership, organisational culture, human capital, social networks and collaboration
**Berg et al., 2018 [18] Norway**	Methodological strategies in resilient health care studies: An integrative review	Meso	Resilience engineering	International	Integrative review	All studies included used qualitative methodology to study resilience. Concepts: individual strategies, sense-making, decision-making, performance variability and expertise

For peer review only

**Biddle et al., 2020** [19] **Germany**	Health system resilience: A literature review of empirical research	Macro	Global health	International	Narrative review	Refers to the balance between an outcome-oriented understanding vs a process-oriented understanding of resilience, taken from Blanchet et al. [20]. Public health studies categorised into three groups: (1) Quantitative studies on service delivery, (2) Qualitative studies focusing on HRH, (3) Broader perspectives: building blocks, etc.<br><br>Macro-level Resilience informed by two discourses: disaster management and healthcare quality (patient safety) and resilience engineering.<br>DRR perspectives have shifted after hurricane Catrina in USA toward community resilience
**Blumenstock et al., 2014** [21] **USA**	Measuring Preparedness: The National Health Security Preparedness Index	Macro	Public health	USA	Policy document	Presents the National Health Security Preparedness Index:<br>Health surveillance - surveillance and epidemiology investigation, environmental and biological monitoring, laboratory testing<br>Community planning and engagement - cross-sector collaboration, at-risk populations, spontaneous volunteers, social capital and cohesions<br>Incident and information management - multi-agency coordination, emergency public information and warning<br>Surge management - emergency medical services, acute & primary care, mental healthcare<br>Countermeasure management - medical supply chain and distribution, countermeasure utilisation and effectiveness"
**Brand & Jax, 2007** [22] **Germany**	Focusing the Meaning(s) of Resilience: Resilience as a Descriptive Concept and a Boundary Object	Meso/Macro	Theoretical	International	Literature review	Focuses on definitions of resilience. 3 categories (descriptive, hybrid and normative). 10 classes and 10 definitions: Original ecological, extended ecological, systematic-heuristic, operational, sociological, ecological-economic, ecosystem-services-related, social-ecological system, metaphoric, sustainability-related.<br><br>Stresses the importance of determining resilience "of what, to what" to operationalise
**Burke et al., 2021** [23] **Ireland**	Building health system resilience through policy development in response to COVID-19 in Ireland: From shock to reform	Macro	Public health - COVID	Ireland	Literature review of health policies	States that the Irish government made several changes to increase healthcare access during the COVID19 pandemic. Argues that existing policies/frameworks should not be abandoned in a pandemic, but authorities should focus on those aspects within the used frameworks that contribute to strengthening and dealing with the acute shock. Views resilience as a transformative process in response to crisis
**Carrington et al., 2021** [24] **Australia**	The impact of disasters on emergency department resources: review against the Sendai framework for disaster risk reduction 2015–2030	Meso	Global health - Disaster	International	Integrative review	Uses SFDRR to assess emergency departments. Identified points for improvement based on SFDRR framework

**Cartwright et al., 2017** [25] **UK**	The changing health priorities of earthquake response and implications for preparedness: a scoping review	Macro	Global health - DRR	LMICs	Scoping review	Mapped the needs in physical health, mental health and health care to disaster cycle. Categorised according to time frame after the event.<br>Preparedness: disconnect between planning and policy, importance of leadership. Acute phase: women, children and elderly disproportionately affected, trauma to extremities, spinal cord injuries, wound debridement, perinatal issues, lack of specialists, physical equipment and lack of workforce, transport systems, coordination difficulties, lack of integration of information systems and international staff. Response: general health needs, respiratory, ocular, GI and dermatology problems, wound infections, late presentations, cold supply chain failure, increased suicides, staff mental health, importance of leadership and coordination, utilitarianism, surveillance. Chronic phase: rehabilitation, unwanted pregnancies, worsening burden of chronic diseases, PTSD and mental health, psychological support for victims and staff<br><br>Mapped lessons onto disaster management cycle. Plan: Policy, address inequalities, surveillance and long-term monitoring, current service gaps, maintenance of essential services, surge capacity, record systems, international staff, logistics, changing health priorities. Organise and equip: medication, equipment and blood, supply points, transportation, donations. Train: disaster mangement, leadership. Exercise: regular exercises to validate plans and arrangements. Evaluate and improve
**Challen et al., 2012** [26] **UK**	Where is the evidence for emergency planning: A scoping review	Macro	Emergency management	HICs	Scoping review	Makes use of FEMA emergency cycle. Key aspects: Mitigation, hazard analysis, capability assessment, emergency planning, capability maintenance, emergency response, recovery, development plans, communications/mass media, informatics and intelligence, other organisational issues
**Curtis et al., 2017** [27] **UK**	Impact of extreme weather events and climate change for health and social care systems	Macro	Climate	UK	Literature review	Identified need to improve hospital infrastructure, wider social infrastructure and collective responsibility towards resilience approach, cross-sectoral work and integration of planning and preparedness.<br><br>Concepts categorised into three domains: Physical (built infrastructure: health facilities, utilities), Institutional (policies and practices, human resources, professional practice), Social (community groups and informal networks)
**Fallah-Aliabadi et al., 2020** [28] **Iran**	Towards developing a model for the evaluation of hospital disaster resilience: A systematic review	Meso	Disaster management	International	Literature review	Assessed several frameworks for disaster resilience: Hospital Safety Index (HSI), Dynamic approach to seismic resilience of hospitals, Measuring framework of the hospital resilience, Indicators for assessing hospital disaster preparedness in Japan.<br><br>Concepts categorised into domains (with 27 sub-domains): Constructive (stability, design, architectural, transportation and transition system<br>Infrastructural: power, water and sewage, communication and IT, Heating, ventilation and air conditioning (HVAC), fuel, medical gas, equipment and furniture, hazardous material, fire system), Administrative (disaster plan, risk assessment and reduction, response, command, coordination, evacuation, need assessment, logistic and supplies, safety committee, continuity of services, volunteers, finance, recover, training)

For peer review only

**Fridell et al., 2020** [29] **Sweden**	Health System Resilience: What Are We Talking About? A Scoping Review Mapping Characteristics and Keywords	Macro	Global health	International	Scoping review + expert consultation	Narrative presentation of definitions of resilience.<br><br>Key features of resilience were mapped onto WHO building blocks of health framework: Leadership and governance (ethics, system capacity, operationalisation, engagement, institutional design), information (surveillance, policy making, evaluation), health workforce (diversity and flexibility, limited resources, incentive, community involvement), financing (resource allocation, funding mechanisms, costing), medicines (sustainability), service delivery (response, prevention)
**Grimm et al., 2021** [30] **Switzerland**	Enhancing the Understanding of Resilience in Health Systems of Low- and Middle-Income Countries: A Qualitative Evidence Synthesis	Macro	Global health	LMIC	Literature review + interviews	Maps Kruk's Resilience Index [31] onto WHO's building blocks of health [32]. Refined concepts from Resilience Index: Realigned relationships (micro-, meso- and macro-level). Public/private partnerships), foresight and motivation (Investment into slow variables, motivation: political will and individual commitment), change management (integrated change management systems and processes help to confer resilience), emergency preparedness
**Haldane et al., 2020** [33] **Canada**	National primary care responses to COVID-19: a rapid review of the literature	Macro	Global health	International	Rapid review	Key lessons from COVID19: Surge capacity should be strengthened. Need to ensure essential services continuity. National guidance needed on biological sampling handling. Most systems increased their telemedicine capabilities
**Hasan et al., 2021** [34] **Canada**	Integrated health service delivery during COVID-19: A scoping review of published evidence from low-income and lower-middle-income countries	Macro	Global health (UHC)	LMIC	Scoping review	Assessment of L-LMIC's ability for integrated health service delivery during COVID19 according to WHO building blocks and pandemic continuum.<br><br>Key recommendations: Strengthening of coordination between healthcare bodies at various levels, cross-sectoral collaboration between government, private sector, media, and armed forces. Establishing integrated platforms for testing, health infrastructure to scale-up (redundancies). Empowering communities to engaged in disease prevention and containment. Expansion of digital health technologies. Build resilience by investing in Primary Healthcare and Integrated Health Service Delivery
**Hosseini et al., 2016** [35] **USA**	A review of definitions and measures of system resilience	Meso	Engineering	International	Literature review	Presents a narrative of various definitions. Considers differences in organisational, social, economic, and engineering domains. Reliability is often used in engineered systems. There is multidimensionality in some definitions Ongoing issues with definitions include uncertain mechanisms to achieve resilience, the role of recovery, and the need to return to steady-state.<br><br>Presents a summary of how studies measure resilience. Qualitative: conceptual framework, semi-quantitative. Quantitative: generic resilience metrics, structural-based modelling
**Iflaifel et al., 2020** [36] **UK**	Resilient Health Care: a systematic review of conceptualisations, study methods and factors that develop resilience	Meso/Macro	Patient safety	International	Literature review	Studies used a mix of qualitative and quantitative methods to study resilience.<br><br>Key concepts: Teamwork, in-situ practical experience, exposure to diverse views and perspectives, trade-offs, protocols & checklists, system design, workarounds

For peer review only - http://bmjopen.bmj.com/site/about/guidelines.xhtml

**Jesus et al., 2021** [37] **Inda**	PREparedness, REsponse and SySTemic transformation (PRE-RE-SyST): a model for disability-inclusive pandemic responses and systemic disparities reduction derived from a scoping review and thematic analysis	Macro	Disability studies	International	Scoping review	Focused on inclusive approach to pandemic responses to reduce health inequalities. Key concepts: transform societies' cultural assumptions about disability. Design systems and policies for structural disability-inclusiveness. Prepare inter-sectoral, disability-inclusive pandemic preparedness. Reduce inequalities. Rapid and compassionate responses in residential and long-term care settings. Provide accessibility
**Koeva & Rohova, 2020** [38] **Bulgaria**	Health System Resilience: Concept Development	Micro/Meso/ Macro	Theoretical	International	Literature review	Narrative presentation of quotations of definitions. Dictionaries distinguish between physical objects and innate human capacity. Engineering: resist shocks, absorb and hold elastic strain before permanent distortion or failure. Psychology: copes and maintains function in face of adversity, trauma, tragedy or stress. Resilience as an outcome, a process, or even contains both dynamic and static properties (within community resilience).<br><br>Resilience is undergoing evolution from outcome-oriented to process-oriented. There is a dynamically to the scope, breadth and depth of definitions. Conceptualisations are increasingly broad and complex. Existence of dynamism, uncertainty, non-linearity and unpredictability.
**Kruk et al., 2017** [31] **USA**	Building resilient health systems: a proposal for a resilience index	Macro	Global health	LMICs	Case studies/ review + framework proposal	Defines resilience as "the capacity of health actors, institutions, and populations to prepare for and effectively respond to crises; maintain core functions when a crisis hits; and, informed by lessons learnt during the crisis, reorganise if conditions require it."<br><br>Key domains: aware (capacity, risks and communication), diverse (meets range of needs, adequate financing), self-regulating (maintain core services and leverage collaborations), integrated (coordination and community engagement), adaptive (distributive decision-making, flexible spending and evaluation for improvement)
**Lapão et al., 2015** [39] **Portugal**	Ebola impact on African health systems entails a quest for more international and local resilience: the case of African Portuguese speaking countries	Meso/Macro	Global health	East Africa	Narrative review and case study	Demonstrated fragility of African health systems during Ebola epidemic: increased demand, inequalities and poor access, infrastructure impact, decreased in standards of care of other areas, themes for learning and improvement, critical services need to continue
**Li et al., 2020** [40] **China**	Analysing Healthcare Facility Resilience: Scientometric Review and Knowledge Map	Meso	Global health (UHC)	China	Literature review	Measures tend to be indicator based (set of dimensions and indicators), function based (quantitative evaluation of functionality of healthcare facilities), or structure based (modelling of system structures (elements and relationships) and testing failure).<br><br>Concepts: preparedness (risk assessment, strengthen capacities, HRH, HIS, leadership and governance, coordination), monitoring (surveillance, warning systems, monitoring systems), absorb (physical capacities, past experience, flexible approaches, HIS, communication, recovery and learning (safety1 & 2 approaches, organisational quality improvement strategies)

**Luke et al., 2021** [41] **Australia**	Safer hospital infrastructure assessments for socio-natural disaster - A scoping review	Meso	Disaster management	International	Scoping review	Uses WHO hospital safety index. Key domains: structural (Previous damage and repair, building integrity, condition of building materials, interaction with other buildings/elements, structural redundancies, architectural design, structure design appropriate to location), non-structural (architectural safety, infrastructure protection, access, and physical security, medical equipment and supplies), critical systems (electrical, telecommunications, water, fire protection, waste management, fuel supplies, medical gases, heating/ventilation)
**Meyer et al., 2020** [42] **USA**	A checklist to improve health system resilience to infectious disease outbreaks and natural hazards	Meso/Macro	Global health , Disaster	International	Scoping review + Interview + Checklist workshop	Proposes checklist as a measurement tool or set of indicators for resilience. Items: maintenance of essential health system services and functions, critical infrastructure and transportation, financing, access, communication, collaboration, coordination and partnerships, leadership and command, surge capacity, risk communications, workforce, infection control
**Nuzzo et al., 2019** [43] **USA**	What makes health systems resilient against infectious disease outbreaks and natural hazards? Results from a scoping review	Macro	Global health	International	Scoping review	Resilience attributes: maintenance of essential functions, access to healthcare, maintaining critical infrastructure and transport, crisis financing, leadership and command, collaboration, coordination and partnerships, communication, flexible plans and management structures, legal preparations, surge capacity, crisis standards of care, human resources for health, infection prevention and control, quality improvement, post-event recovery (psychological health of staff, revision of plans, rebuilding social cohesion and trust).<br><br>Themes are consistent with the Resilience Index. Additional findings: developing crisis standards of care, plan for post-event recovery, commitment to quality improvement to ensure lessons learned.
**Oppenheim et al., 2019** [44] **USA**	Assessing global preparedness for the next pandemic: development and application of an Epidemic Preparedness Index	Macro	Global health	International	Framework proposal and evaluation	Epidemic Preparedness Index: public health infrastructure (surveillance, immunisation, medical workforce, hospital capacity, coordination), physical infrastructure (water and sanitation, roads, phones, internet, logistics), institutional capacity (political stability, corruption, bureaucratic effectiveness, armed conflict, homicide, vital registration), economic resources (government revenue generation, per capita income, gross domestic product, health spending, resource dependency), public health communications (public education, risk communication)
**Penaloza et al., 2020** [45] **Brazil**	A resilience engineering perspective of safety performance measurement systems: A systematic literature review	Meso	Resilience engineering	International	Literature review	Safety performance measurement systems should support real-time monitoring and feedback of everyday performance variability, with the ability to analyse adverse events and excellent practices. Measures should be dynamic and subject to re-evaluation

For peer review only

**Pillay & Morel, 2020 [46] Australia**	Measuring Resilience Engineering: An Integrative Review and Framework for Bench-Marking Organisational Safety	Meso	Resilience engineering	International	Integrative review	"Resilience Engineering is a perspective for organisational safety management which enables organisational members to actively anticipate, respond, monitor and learn; by adapting to operate at the boundary of safe operations by narrowing the gap between work as imagined and work as performed; and manifested in an organisation's culture, cognition and behaviours"<br><br>Argues the need to establish boundaries of safe performance, balance trade-offs between production and safety, and dealing with managed vs prescribed safety. Shifts resilience culture towards resilient climate, acknowledging environmental conditions necessary for resilience.<br><br>Key indicators of resilience: learning culture, flexibility, top management commitment, awareness, preparedness, just culture
**Rameshshanker et al., 2021 [47] Canada**	Health system resilience to extreme weather events in Asia-Pacific: a scoping review	Macro/Meta	Disaster, climate change	Asia-Pacific	Literature review	Explored how well health systems were resilient towards adverse weather events. Categorised efforts according to WHO building blocks checklist.<br>Medical workforce was the most frequently mentioned aspect, while health financing was the least.
**Rangachari & Woods, 2020 [48] USA**	Preserving Organizational Resilience, Patient Safety, and Staff Retention during COVID-19 Requires a Holistic Consideration of the Psychological Safety of Healthcare Workers	Micro/Meso	Psychology, Operational research	International	Narrative review	During COVID19, trust and leadership were key aspects to maintain holistic resiliience of staff, which may improve organisational resilience. Advocates for environment of trst, empowerment and safety to enable patient safety concerns to be brought up
**Ravi et al., 2019 [49] USA**	Establishing a theoretical foundation for measuring global health security: A scoping review	Macro	Global health - HSS	International	Scoping review	Focused on health security, centred around performance of critical health security systems, biosafety and biosecurity, and public health preparedness. Underscored importance of real-time health information systems.<br><br>Key concepts: measuring global health security requires analysis of existing prevention, detection, and response capacities, as well as efforts to build core public health, healthcare, and biosecurity capacities. Assessments of national programs and efforts to mitigate a critical subset of priority threats could serve as useful proxies for measuring global health security. There are measurable enabling factors that facilitate global health security-strengthening efforts. Measuring global health security requires consideration of the risk environment from which infectious disease threats might emerge
**Rezaei et al., 2018 [50] Iran**	Hospitals preparedness using WHO guideline: A systematic review and meta-analysis	Meso	Emergency management	International	Systematic review and meta-analysis	Measured compliance to WHO hospital emergency checklist. Domains include command and control, communication, safety and security, triage, surge capacity, continuity of essential services, human resources for health, logistics and supply management, post-disaster recovery

For peer review only

**Son et al., 2020 [51]** **USA**	Investigating resilience in emergency management: An integrative review of literature	Meso/Macro	Emergency management	International	Integrative review	Presents a table of collated definitions. Stressed the temporal nature in defining resilience. Some focus on pre-event whilst others focus on intra-event. Expected disruption: some focus on the ability to deal with unexpected events, whilst others do not view the distinction. Means to achieve resilience: allocation of roles and resources, HIS or adjusting procedures.<br><br>Dimensions of resilience in emergency management: collective sense-making (improvisation, honest communication, virtual role systems, attitude of wisdom), team decision making (incident command system, training in generic decision-making protocols), harmonising WAI and WAD, interaction and coordination.<br><br>Tools used to support human operators for resilience: geospatial mapping, event history logs, mobile communication systems, decision support tools
**Tessema et al., 2021 [52]** **Australia**	The COVID-19 pandemic and healthcare systems in Africa: a scoping review of preparedness, impact and response	Macro	Global health, COVID	Africa	Scoping review	Used Population, Concept Context (PCC) framework for defining eligibility<br>Measured preparedness, impact and response of health systems<br>Framed within WHO building blocks.<br><br>Key findings: preparedness (limited resources, knowledge gaps, lack of training, limited surge capacity and testing ability, limited health services), Impact (reduced patient flow, miss appointments for chronic services, re-orientation of services deter essential services provision, not all services affected), impact on workforce (stigma, mental health illness), response (provision of guidelines, telehealth, segregation of patient streams)
**Tin et al., 2010 [53]** **Myanmar**	An approach to health system strengthening in the Union of Myanmar	Macro	Public health - HSS	Myanmar	Case study/ national review	Prioritised service delivery, programme coordination and human resource management to strengthen Myanmar's health system. Takes a systems thinking approach. Uses Health Systems Strengthening indicators at measures and index to compare several countries.
**Tippong et al., 2021 [54]** **UK**	A Review of Applications of Operational Research in Healthcare Coordination in Disaster Management	Meso/Macro	Emergency and Disaster management	International	Literature review	Measured coordination effectiveness in healthcare systems. Various staffing patterns have trade-offs: financial cost vs sustainable pattern. Triage and minimisation of beds needed. Coordination between healthcare-network is important for surge management and adaptive capacity.
**Turenne et al., 2019 [55]** **France**	Conceptual analysis of health systems resilience: A scoping review	Macro	Theoretical	International	Scoping review	Presented a collated table of definitions and word frequency table. Concepts are dependent on contexts (different groups and populations)<br>In the face of changes. Argues that resilience thinking may be multi-sectoral, but health systems resilience is primarily concerned with the functions of the health system

For peer review only

**Turner et al., 2021** [56] **Colombia**	Systematic review of experiences and perceptions of key actors and organisations at multiple levels within health systems internationally in responding to COVID-19	Micro/Meso/ Macro	Global health - COVID	International	Narrative review	Individual level: staff needed to adapt to new working conditions, burnout and psychological distress: work intensity, staff shortages, fear of contagion and transmission, positive adaptation: Telehealth, community healthcare workers, creative approaches<br><br>Organisational level: COVID as catalyst for service change, need to provide psychological support for staff, open management style during crisis<br><br>Local health system level: continuous training to support preparedness for future pandemics, the need to look at working conditions, the need to coordinate diverse actors (community and primary health etc), review and update regulations
**Wiig et al., 2020** [57] **Norway**	Defining the boundaries and operational concepts of resilience in the resilience in healthcare research programme	Meso	Patient safety	International	Debate and review	Centred around engineering, psychological and ecological perspectives: Resilience of what, to what, through what. Focused around high quality healthcare, centred around set a capacities, based on events which provide challenges, changes or disruptions. Grounded in multi-level adaptation, enhancement and reorganisation, indicating diversity, coordination and collaboration and is open-ended
**Xin & Xu, 2012** [58] **China**	Hospital emergency management research in China: trends and challenges	Macro	Emergency management	China	Narrative review	Defines crisis based on disruptions to hospital systems: patient surge, provision of services, reputation, financial trouble, or major incident. Noted a move from normal vs abnormal crisis to all-hazards. Comprehensive management system may be more useful than temporary command and control, or professional response team. Identified current issues with Chinese medical systems based on WHO building blocks
**Zhong et al., 2014** [59] **China**	Development of hospital disaster resilience: conceptual framework and potential measurement	Macro	Global health - Disaster	International	Literature review	Presented a collated list of definitions from different disciplines. Most measures focus on structure, but hardly on process and outcome (based on Donebedian categories of quality of care). Propose measures as a framework: Hospital safety and vulnerability - surveillance, risk & safety. Disaster preparedness and resources - Leadership, cooperation and communication, logistics & stockpiles, workforce, training & drills. Continuity of essential services - surge capacity, emergency medicines. Recovery and adaptation - evaluation, adaptation and recovery capability.<br><br>Factors conferring resilient healthcare: Accessibility, Critical services, Leadership, Integration with community

Legend: WHO = World Health Organisation. DRR = Disaster Risk Reduction. USA = United States of America. HRH = Human Resources for Health. L-LMIC = Low and Low-Middle Income Countries. PTSD = Post-Traumatic Stress Disorder. SFDRR = Sendai Framework for Disaster Risk Reduction. HIS = Health Information Systems

For peer review only

For peer review only

Appendix 4: Summary characteristics of selected resilience frameworks

Framework (reference)	Key components or indicators or principles	Hierarchical focus	Paradigm	Pre-event	Intra-event	Post-event
**Resilience Potentials** [60]	Anticipate,<br>Monitor,<br>Respond,<br>Learn	Meso	Engineering			
**Independent EM concepts** [51]	Collective sense-making,<br>Team decision-making,<br>Harmonising WAI/WAD,<br>Interaction and Coordination	Meso	Emergency Management			
**FEMA adapted emergency management cycle** [26]	Mitigation, hazard and capacity assessment<br>Planning<br>Information systems<br>Continuity of services<br>Emergency response<br>Communications<br>Recovery	Meso	Emergency Management			
**WHO Hospital Safety Index (HSI)** [41]	Structural (e.g. building integrity, redundancies, previous damage)<br>Non-structural (e.g. critical systems, medical equipment and supplies)<br>Disaster management system	Meso	Healthcare			
**WHO hospital disaster preparedness** [46,50]	Command and Control<br>Communication<br>Safety and Security<br>Triage<br>Surge capacity<br>Continuity of essential services<br>HRH<br>Logistics and supply management<br>Post-disaster recovery	Meso	Healthcare			
**Hospital disaster resilience model** [28]	3 domains with 27 sub-domains:<br>Constructive (stability, design, architectural, transportation)<br>Infrastructural (power, water/sewage, communication/IT, HVAC, fuel, medical gas, equipment<br>Administrative (disaster plan, command, coordination, needs assessment, logistics and supplies, safety committee, continuity of services, volunteers, finance, recovery, training)	Meso	Healthcare			
**Independent hospital and healthcare resilience** [59]	4 principles:<br>Robustness<br>Redundancy<br>Rapidity<br>Resourcefulness<br>4 cross cutting domains:<br>Vulnerability and safety<br>Recovery and adaptation<br>Continuity of essential services<br>Preparedness and resources	Meso/Macro	Healthcare			
**Independent disaster preparedness cycle** [25]	5 parts of cycle further divided into components, all underpinned by leadership & coordination:<br>Plan<br>Organise & equip<br>Train<br>Exercise	Meso/Macro	Disaster Management			

For peer review only

	Evaluate & improve					
**Concepts conferring resilience** [17]	Material resources<br>Preparedness & planning<br>Information management<br>Collateral pathways and redundancies<br>Governance processes<br>Leadership<br>Organisational culture<br>Human capital<br>Social networks and collaboration	Meso/Macro	Healthcare			
**Primary Care Framework for pandemics (from H1N1)** [33]	Clinical service delivery,<br>Public health functions,<br>Primary care facility operational level<br>Health system level	Meso/Macro	Healthcare			
**NHS Emergency Preparedness, Resilience and Response (EPRR) framework**	Preparedness & anticipation<br>Continuity<br>Subsidiary: decisions at lowest level<br>Communication<br>Cooperation & integration<br>Direction	Macro	Healthcare			
**SFDRR** [61]	Reduce Disaster Mortality<br>Reduce the Number of Affected People<br>Reduce Direct Disaster Economic Loss<br>Reduce Disaster Damage to Critical Infrastructure and Disruption of Basic Services<br>Increase the Number of Countries with National and Sub-National/Local Disaster Risk Reduction Strategies<br>Enhance International Cooperation<br>Increase the Availability of and Access to Multi-Hazard Early Warning Systems	Macro	Disaster Management			
**Global Health Security Index** [62]	Prevent (pathogens)<br>Detect<br>Respond<br>Healthcare (formal)<br>Norms<br>Risk	Macro	Public health			
**Epidemic Preparedness Index** [44]	Public health infrastructure: surveillance, immunisation, medical workforce, hospital capacity, coordination<br>Physical infrastructure: water and sanitation, roads, phones, internet, logistics<br>Institutional capacity: political stability, corruption, bureaucratic effectiveness, armed conflict, homicide, vital registration<br>Economic resources: government revenue generation, per capita income, gross domestic product, health spending, resource dependency<br>Public health communications: public education, risk communication	Macro	Public health			
**European Primary Care Monitoring** [63]	Structure: Governance, economic conditions, workforce<br>Process: access, comprehensiveness, coordination and continuity<br>Outcome: Quality, efficiency, equity	Macro	Healthcare			

For peer review only

**USA Public Health Infrastructure** [64]	Assessment: monitor health, diagnose/investigate<br>Policy development: Inform/educate/empower, community partnerships, develop policies<br>Assurance: Enforce laws, link care, competent workforce, evaluate	Macro	Public health			
**WHO building blocks** [32]	Leadership & governance,<br>Health Information Systems (HIS),<br>Human Resources for Health (HRH),<br>Financing,<br>Service delivery,<br>Essential medicines and technologies	Macro	Healthcare			
**National Health Security Preparedness Index (NHSPI)** [21]	Health surveillance: surveillance and epidemiology investigation, environmental and biological monitoring, laboratory testing<br>Community planning and engagement: cross-sector collaboration, at-risk populations, spontaneous volunteers, social capital and cohesions<br>Incident and information management: multi-agency coordination, emergency public information and warning<br>Surge management: emergency medical services, acute & primary care, mental healthcare<br>Countermeasure management: medical supply chain and distribution, countermeasure utilisation and effectiveness	Macro	Healthcare			
**Integrated Health Service Delivery** [34]	Clinical,<br>Service,<br>Organisational,<br>Functional	Macro	Healthcare			
**Emerging Infectious Diseases Preparedness** [65]	Hardware: Surveillance, infrastructure & medical supplies, workforce, communication mechanisms<br>Software: Governance, trust	Macro	Healthcare			
**Myanmar Health Systems Strengthening Strategy** [53]	Service delivery<br>Programme coordination<br>Human resources	Macro	Healthcare			
**Stockholm Resilience Centre 7 Principles** [66]	Foster complex adaptive systems thinking,<br>Maintain diversity & redundancies,<br>Broaden Participation,<br>Manage connectivity,<br>Manage slow variables and feedback,<br>Encourage learning,<br>Polycentric governance	Macro/Meta	Socioecological systems			
**Refined conceptual models of health system resilience** [30 43]	Foundations of resilience added onto the Resilience Index:<br>Realigned relationships,<br>Foresight and motivation,<br>Change management,<br>Emergency preparedness<br>Crisis standards of care<br>Recovery plans<br>Learning and improvement	Micro/Meso/Macro	Healthcare			
**Health Systems Resilience Index** [31]	Aware: capacity, risks and communication<br>Diverse: meets range of needs, adequate financing<br>Self-regulating: maintain core services and leverage collaborations<br>Integrated: coordination and community engagement<br>Adaptive: distributive decision-making, flexible spending and evaluation for improvement	Meso/Macro/Meta	Healthcare			

Legend: Frameworks are categorised according to predominant hierarchical

For peer review only

focus and paradigm. They are colour-coded according to which time domain they cover.

Abbreviations: EM = Emergency Management. WAI/WAD = Work-As-Imagined vs Work-As-Done. FEMA = Federal Emergency Management Agency. WHO = World Health Organisation. HRH = Human Resources for Health. IT = Information Technology. HVAC = Heating, Ventilation and Air-Conditioning. NHS = UK's National Health Service. SFDRR = Sendai Framework for Disaster Risk Reduction. USA = United States of America.

For peer review only

References

1. Xiao Y, Watson M. Guidance on Conducting a Systematic Literature Review. *Journal of Planning Education and Research* 2019;39(1):93-112. doi: 10.1177/0739456x17723971
2. Greenhalgh T, Robert G, Macfarlane F, et al. Storylines of research in diffusion of innovation: a meta-narrative approach to systematic review. *Soc Sci Med* 2005;61(2):417-30. doi: 10.1016/j.socscimed.2004.12.001 [published Online First: 2005/05/17]
3. Gough D. Meta-narrative and realist reviews: guidance, rules, publication standards and quality appraisal. *BMC Medicine* 2013;11(1):22. doi: 10.1186/1741-7015-11-22
4. Smith V, Devane D, Begley CM, et al. Methodology in conducting a systematic review of systematic reviews of healthcare interventions. *BMC Med Res Methodol* 2011;11(1):15. doi: 10.1186/1471-2288-11-15 [published Online First: 20110203]
5. Becker L, Oxman A. Overviews of reviews. In: Higgins J, Green S, eds. Cochrane handbook for systematic reviews of interventions. Oxford: The Cochrane Collaboration 2010.
6. Wong G, Greenhalgh T, Westhorp G, et al. RAMESES publication standards: meta-narrative reviews. *BMC Medicine* 2013;11(1):20. doi: 10.1186/1741-7015-11-20
7. Higgins JP TJ, Chandler J, Cumpston M, Li T, Page MJ, Welch VA, editor(s). Cochrane Handbook for Systematic Reviews of Interventions Version 6.2 (updated February 2021): Cochrane; 2021 [Available from: training.cochrane.org/handbook accessed Feb 2022.
8. Hannes K. Supplementary Guidance for Inclusion of Qualitative Research in Cochrane Systematic Reviews of Interventions. Version 1 (updated August 2011). In: Noyes J BA, Hannes K, Harden A, Harris J, Lewin S, Lockwood C ed.: Cochrane Collaboration Qualitative Methods Group 2011.
9. HONG QN, PLUYE P, FÀBREGUES S, et al. MIXED METHODS APPRAISAL TOOL (MMAT): McGill University; 2018 [Available from: http://mixedmethodsappraisaltoolpublic.pbworks.com/w/file/fetch/127916259/MMAT_2018_criteria-manual_2018-08-01_ENG.pdf accessed Jan 2022.
10. CASP. Critical Appraisal Skills Programme - Qualitative Checklist 2018 [Available from: https://casp-uk.net/wp-content/uploads/2018/01/CASP-Qualitative-Checklist-2018.pdf accessed

For peer review only - http://bmjopen.bmj.com/site/about/guidelines.xhtml

Feb 2022.

11. Harrison R, Jones B, Gardner P, et al. Quality assessment with diverse studies (QuADS): an appraisal tool for methodological and reporting quality in systematic reviews of mixed- or multi-method studies. *BMC Health Services Research* 2021;21(1):144. doi: 10.1186/s12913-021-06122-y
12. Shea BJ, Reeves BC, Wells G, et al. AMSTAR 2: a critical appraisal tool for systematic reviews that include randomised or non-randomised studies of healthcare interventions, or both. *BMJ* 2017;358:j4008. doi: 10.1136/bmj.j4008
13. Alami H, Lehoux P, Fleet R, et al. How Can Health Systems Better Prepare for the Next Pandemic? Lessons Learned From the Management of COVID-19 in Quebec (Canada). *Frontiers in public health* 2021;9:671833. doi: 10.3389/fpubh.2021.671833
14. Augustynowicz A, Opolski J, Waszkiewicz M. Resilient Health and the Healthcare System. A Few Introductory Remarks in Times of the COVID-19 Pandemic. *Int J Environ Res Public Health* 2022;19(6) doi: 10.3390/ijerph19063603
15. Ayanore MA, Amuna N, Aviisah M, et al. Towards Resilient Health Systems in Sub-Saharan Africa: A Systematic Review of the English Language Literature on Health Workforce, Surveillance, and Health Governance Issues for Health Systems Strengthening. *Annals of Global Health* 2019;85(1) doi: 10.5334/aogh.2514
16. Banwell N, Rutherford S, Mackey B, et al. Towards improved linkage of disaster risk reduction and climate change adaptation in health: A review. *Int J Environ Res Public Health* 2018;15(4) doi: 10.3390/ijerph15040793
17. Barasa E, Mbau R, Gilson L. What is resilience and how can it be nurtured? A systematic review of empirical literature on organizational resilience. *International Journal of Health Policy and Management* 2018;7(6):491-503. doi: 10.15171/ijhpm.2018.06
18. Berg SH, Akerjordet K, Ekstedt M, et al. Methodological strategies in resilient health care studies: An integrative review. *Safety Science* 2018;110:300-12. doi: 10.1016/j.ssci.2018.08.025
19. Biddle L, Wahedi K, Bozorgmehr K. Health system resilience: A literature review of empirical research. *Health Policy and Planning* 2020;35(8):1084-109. doi: 10.1093/heapol/czaa032
20. Blanchet K, Nam SL, Ramalingam B, et al. Governance and Capacity to Manage Resilience of Health Systems: Towards a New Conceptual Framework. *International Journal of Health Policy and Management*

For peer review only

2017;6(8):431-35. doi: 10.15171/ijhpm.2017.36
21. Blumenstock J, Bakker G, Jarris PE. Measuring Preparedness: The National Health Security Preparedness Index. *Journal of Public Health Management and Practice* 2014;20(3):361-63. doi: 10.1097/phh.0000000000000073
22. Brand FS, Jax K. Focusing the Meaning(s) of Resilience: Resilience as a Descriptive Concept and a Boundary Object. *Ecology and Society* 2007;12(1)
23. Burke S, Parker S, Fleming P, et al. Building health system resilience through policy development in response to COVID-19 in Ireland: From shock to reform. *Lancet Reg Health Eur* 2021;9:100223. doi: 10.1016/j.lanepe.2021.100223 [published Online First: 20211007]
24. Carrington MA, Ranse J, Hammad K. The impact of disasters on emergency department resources: review against the Sendai framework for disaster risk reduction 2015–2030. *Australas Emerg Care* 2021;24(1):55-60. doi: 10.1016/j.auec.2020.09.003
25. Cartwright C, Hall M, Lee ACK. The changing health priorities of earthquake response and implications for preparedness: a scoping review. *Public Health* 2017;150:60-70. doi: 10.1016/j.puhe.2017.04.024
26. Challen K, Lee AC, Booth A, et al. Where is the evidence for emergency planning: A scoping review. *BMC Public Health* 2012;12(1) doi: 10.1186/1471-2458-12-542
27. Curtis S, Fair A, Wistow J, et al. Impact of extreme weather events and climate change for health and social care systems. *Environmental Health: A Global Access Science Source* 2017;16 doi: 10.1186/s12940-017-0324-3
28. Fallah-Aliabadi S, Ostadtaghizadeh A, Ardalan A, et al. Towards developing a model for the evaluation of hospital disaster resilience: A systematic review. *BMC Health Services Research* 2020;20(1) doi: 10.1186/s12913-020-4915-2
29. Fridell M, Edwin S, Schreeb JV, et al. Health system resilience: what are we talking about? A scoping review mapping characteristics and keywords. *International Journal of Health Policy and Management* 2020;9(1):6-16. doi: 10.15171/ijhpm.2019.71
30. Grimm PY, Oliver S, Merten S, et al. Enhancing the Understanding of Resilience in Health Systems of Low- and Middle-Income Countries: A Qualitative Evidence Synthesis. *International Journal of Health Policy and Management* 2021 doi: 10.34172/ijhpm.2020.261
31. Kruk ME, Ling EJ, Bitton A, et al. Building resilient health systems: a proposal for a resilience index. *BMJ* 2017;357:j2323. doi:

For peer review only

10.1136/bmj.j2323
32. WHO. Key components of a well functioning health system 2010 [Available from: https://www.who.int/healthsystems/EN_HSSkeycomponents.pdf accessed Nov 2019.
33. Haldane V, Zhang Z, Abbas RF, et al. National primary care responses to COVID-19: a rapid review of the literature. *BMJ Open* 2020;10(12):e041622. doi: 10.1136/bmjopen-2020-041622 [published Online First: 20201208]
34. Hasan MZ, Neill R, Das P, et al. Integrated health service delivery during COVID-19: A scoping review of published evidence from low-income and lower-middle-income countries. *BMJ Global Health* 2021;6(6) doi: 10.1136/bmjgh-2021-005667
35. Hosseini S, Barker K, Ramirez-Marquez JE. A review of definitions and measures of system resilience. *Reliability Engineering & System Safety* 2016;145:47-61. doi: https://doi.org/10.1016/j.ress.2015.08.006
36. Iflaifel M, Lim RH, Ryan K, et al. Resilient Health Care: a systematic review of conceptualisations, study methods and factors that develop resilience. *BMC Health Serv Res* 2020;20(1):324. doi: 10.1186/s12913-020-05208-3 [published Online First: 2020/04/19]
37. Jesus TS, Kamalakannan S, Bhattacharjya S, et al. PREparedness, REsponse and SySTemic transformation (PRE-RE-SyST): a model for disability-inclusive pandemic responses and systemic disparities reduction derived from a scoping review and thematic analysis. *Int J Equity Health* 2021;20(1):204. doi: 10.1186/s12939-021-01526-y [published Online First: 20210914]
38. Koeva S, Rohova M. Health system resilience: concept development. *Journal of Imab* 2020;26(3):3251-58. doi: 10.5272/jimab.2020263.3251
39. Lapão LV, Silva A, Pereira N, et al. Ebola impact on African health systems entails a quest for more international and local resilience: the case of African Portuguese speaking countries. *Pan Afr Med J* 2015;22 Suppl 1(Suppl 1):15. doi: 10.11694/pamj.supp.2015.22.1.6653 [published Online First: 20151011]
40. Li X, Krumholz HM, Yip W, et al. Quality of primary health care in China: challenges and recommendations. *The Lancet* 2020;395(10239):1802-12. doi: 10.1016/S0140-6736(20)30122-7
41. Luke J, Franklin R, Aitken P, et al. Safer hospital infrastructure assessments for socio-natural disaster - A scoping review. *Prehosp Disaster Med* 2021;36(5):627-35. doi: 10.1017/S1049023X21000650
42. Meyer D, Bishai D, Ravi SJ, et al. A checklist to improve health system resilience to infectious disease outbreaks and natural hazards. *BMJ Glob*

For peer review only - http://bmjopen.bmj.com/site/about/guidelines.xhtml

Health 2020;5(8) doi: 10.1136/bmjgh-2020-002429
43. Nuzzo JB, Meyer D, Snyder M, et al. What makes health systems resilient against infectious disease outbreaks and natural hazards? Results from a scoping review. *BMC Public Health* 2019;19(1) doi: 10.1186/s12889-019-7707-z
44. Oppenheim B, Gallivan M, Madhav NK, et al. Assessing global preparedness for the next pandemic: development and application of an Epidemic Preparedness Index. *BMJ Global Health* 2019;4(1):e001157. doi: 10.1136/bmjgh-2018-001157
45. Penaloza GA, Saurin TA, Formoso CT, et al. A resilience engineering perspective of safety performance measurement systems: A systematic literature review. *Safety Science* 2020;130 doi: 10.1016/j.ssci.2020.104864
46. Pillay M, Morel G. Measuring Resilience Engineering: An Integrative Review and Framework for Bench-Marking Organisational Safety. *Safety* 2020;6(3) doi: 10.3390/safety6030037
47. Rameshshanker V, Wyngaarden S, Lau LL, et al. Health system resilience to extreme weather events in Asia-Pacific: a scoping review. *Clim Dev* 2021;13(10):944-58. doi: 10.1080/17565529.2020.1870425
48. Rangachari P, J LW. Preserving Organizational Resilience, Patient Safety, and Staff Retention during COVID-19 Requires a Holistic Consideration of the Psychological Safety of Healthcare Workers. *Int J Environ Res Public Health* 2020;17(12) doi: 10.3390/ijerph17124267 [published Online First: 2020/06/19]
49. Ravi SJ, Meyer D, Cameron E, et al. Establishing a theoretical foundation for measuring global health security: A scoping review. *BMC Public Health* 2019;19(1) doi: 10.1186/s12889-019-7216-0
50. Rezaei F, Maracy MR, Yarmohammadian MH, et al. Hospitals preparedness using WHO guideline: A systematic review and meta-analysis. *Hong Kong Journal of Emergency Medicine* 2018;25(4):211-22. doi: 10.1177/1024907918760123
51. Son C, Sasangohar F, Neville T, et al. Investigating resilience in emergency management: An integrative review of literature. *Appl Ergon* 2020;87 doi: 10.1016/j.apergo.2020.103114
52. Tessema GA, Kinfu Y, Dachew BA, et al. The COVID-19 pandemic and healthcare systems in Africa: a scoping review of preparedness, impact and response. *Bmj Global Health* 2021;6(12) doi: 10.1136/bmjgh-2021-007179
53. Tin N, Lwin S, Kyaing NN, et al. An approach to health system strengthening in the Union of Myanmar. *Health Policy* 2010;95(2-3):95-

For peer review only

102. doi: 10.1016/j.healthpol.2009.11.013
54. Tippong D, Petrovic S, Akbari V. A Review of Applications of Operational Research in Healthcare Coordination in Disaster Management. *Eur J Oper Res* 2021 doi: 10.1016/j.ejor.2021.10.048 [published Online First: 20211028]
55. Turenne CP, Gautier L, Degroote S, et al. Conceptual analysis of health systems resilience: A scoping review. *Soc Sci Med* 2019;232:168-80. doi: 10.1016/j.socscimed.2019.04.020 [published Online First: 20190430]
56. Turner S, Botero-Tovar N, Herrera MA, et al. Systematic review of experiences and perceptions of key actors and organisations at multiple levels within health systems internationally in responding to COVID-19. *Implementation Science* 2021;16(1) doi: 10.1186/s13012-021-01114-2
57. Wiig S, Aase K, Billett S, et al. Defining the boundaries and operational concepts of resilience in the resilience in healthcare research program. *BMC Health Services Research* 2020;20(1) doi: 10.1186/s12913-020-05224-3
58. Xin YT, Xu KY. Hospital emergency management research in China: trends and challenges. *Emerg Med J* 2012;29(5):353-7. doi: 10.1136/emermed-2011-200512 [published Online First: 20111103]
59. Zhong S, Clark M, Hou X-Y, et al. Development of hospital disaster resilience: conceptual framework and potential measurement. *Emergency Medicine Journal* 2014;31(11):930-U8901. doi: 10.1136/emermed-2012-202282
60. Hollnagel E. Epilogue: RAG–the resilience analysis grid. Resilience engineering in practice: CRC Press 2017:275-96.
61. Calkins J. Moving forward after Sendai: How countries want to use science, evidence and technology for disaster risk reduction. *PLoS Currents* 2015;7(DISASTERS) doi: 10.1371/currents.dis.22247d6293d4109d09794890bcda1878
62. Bell JA, Nuzzo JB. Global Health Security Index: Advancing Collective Action and Accountability Amid Global Crisis: NTI/John Hopkins center for health security; 2021 [Available from: www.GHSIndex.org accessed Mar 2022.
63. Kringos DS, Boerma WGW, Bourgueil Y, et al. The european primary care monitor: structure, process and outcome indicators. *BMC Fam Pract* 2010;11(1):81. doi: 10.1186/1471-2296-11-81
64. Benjamin GC. Ensuring health equity during the COVID-19 pandemic: The role of public health infrastructure. *Revista Panamericana de Salud Publica/Pan American Journal of Public Health* 2020;44 doi: 10.26633/RPSP.2020.70

For peer review only

65. Palagyi A, Marais BJ, Abimbola S, et al. Health system preparedness for emerging infectious diseases: A synthesis of the literature. *Glob Public Health* 2019;14(12):1847-68. doi: 10.1080/17441692.2019.1614645 [published Online First: 20190514]
66. Biggs R, Schlüter M, Schoon ML. Principles for Building Resilience: Cambridge University Press 2015.

For peer review only

PRISMA 2020 Checklist

Section and Topic	Item #	Checklist item	Location where item is reported
**TITLE**			
Title	1	Identify the report as a systematic review.	P1, 4
**ABSTRACT**			
Abstract	2	See the PRISMA 2020 for Abstracts checklist.	P1
**INTRODUCTION**			
Rationale	3	Describe the rationale for the review in the context of existing knowledge.	P4
Objectives	4	Provide an explicit statement of the objective(s) or question(s) the review addresses.	P5
**METHODS**			
Eligibility criteria	5	Specify the inclusion and exclusion criteria for the review and how studies were grouped for the syntheses.	P5
Information sources	6	Specify all databases, registers, websites, organisations, reference lists and other sources searched or consulted to identify studies. Specify the date when each source was last searched or consulted.	P5
Search strategy	7	Present the full search strategies for all databases, registers and websites, including any filters and limits used.	P5
Selection process	8	Specify the methods used to decide whether a study met the inclusion criteria of the review, including how many reviewers screened each record and each report retrieved, whether they worked independently, and if applicable, details of automation tools used in the process.	P6
Data collection process	9	Specify the methods used to collect data from reports, including how many reviewers collected data from each report, whether they worked independently, any processes for obtaining or confirming data from study investigators, and if applicable, details of automation tools used in the process.	P6
Data items	10a	List and define all outcomes for which data were sought. Specify whether all results that were compatible with each outcome domain in each study were sought (e.g. for all measures, time points, analyses), and if not, the methods used to decide which results to collect.	P6, Appendices p5-13
	10b	List and define all other variables for which data were sought (e.g. participant and intervention characteristics, funding sources). Describe any assumptions made about any missing or unclear information.	P6
Study risk of bias assessment	11	Specify the methods used to assess risk of bias in the included studies, including details of the tool(s) used, how many reviewers assessed each study and whether they worked independently, and if applicable, details of automation tools used in the process.	P6
Effect measures	12	Specify for each outcome the effect measure(s) (e.g. risk ratio, mean difference) used in the synthesis or presentation of results.	P6
Synthesis methods	13a	Describe the processes used to decide which studies were eligible for each synthesis (e.g. tabulating the study intervention characteristics and comparing against the planned groups for each synthesis (item #5)).	P6
	13b	Describe any methods required to prepare the data for presentation or synthesis, such as handling of missing summary statistics, or data conversions.	P6
	13c	Describe any methods used to tabulate or visually display results of individual studies and syntheses.	P6 – 12 Appendices p5-13
	13d	Describe any methods used to synthesize results and provide a rationale for the choice(s). If meta-analysis was performed, describe the model(s), method(s) to identify the presence and extent of statistical heterogeneity, and software package(s) used.	P6
	13e	Describe any methods used to explore possible causes of heterogeneity among study results (e.g. subgroup analysis, meta-regression).	N/a
	13f	Describe any sensitivity analyses conducted to assess robustness of the synthesized results.	N/a
Reporting bias	14	Describe any methods used to assess risk of bias due to missing results in a synthesis (arising from reporting biases).	P6

For peer review only - http://bmjopen.bmj.com/site/about/guidelines.xhtml

PRISMA 2020 Checklist

Section and Topic	Item #	Checklist item	Location where item is reported
assessment			
Certainty assessment	15	Describe any methods used to assess certainty (or confidence) in the body of evidence for an outcome.	P6, Appendices
**RESULTS**			
Study selection	16a	Describe the results of the search and selection process, from the number of records identified in the search to the number of studies included in the review, ideally using a flow diagram.	P7-8
	16b	Cite studies that might appear to meet the inclusion criteria, but which were excluded, and explain why they were excluded.	P7
Study characteristics	17	Cite each included study and present its characteristics.	P7-8, Appendices p5-13
Risk of bias in studies	18	Present assessments of risk of bias for each included study.	Appendices p5-13
Results of individual studies	19	For all outcomes, present, for each study: (a) summary statistics for each group (where appropriate) and (b) an effect estimate and its precision (e.g. confidence/credible interval), ideally using structured tables or plots.	Appendices p5-13
Results of syntheses	20a	For each synthesis, briefly summarise the characteristics and risk of bias among contributing studies.	P7, 12-13 Appendices p5-13
	20b	Present results of all statistical syntheses conducted. If meta-analysis was done, present for each the summary estimate and its precision (e.g. confidence/credible interval) and measures of statistical heterogeneity. If comparing groups, describe the direction of the effect.	N/a
	20c	Present results of all investigations of possible causes of heterogeneity among study results.	P7, 12-13 Appendices p5-13
	20d	Present results of all sensitivity analyses conducted to assess the robustness of the synthesized results.	N/a
Reporting biases	21	Present assessments of risk of bias due to missing results (arising from reporting biases) for each synthesis assessed.	P7, 12-13 Appendices p5-13
Certainty of evidence	22	Present assessments of certainty (or confidence) in the body of evidence for each outcome assessed.	N/a
**DISCUSSION**			
Discussion	23a	Provide a general interpretation of the results in the context of other evidence.	P7-14,
	23b	Discuss any limitations of the evidence included in the review.	P14
	23c	Discuss any limitations of the review processes used.	P14
	23d	Discuss implications of the results for practice, policy, and future research.	P12-14,
**OTHER INFORMATION**			
Registration and protocol	24a	Provide registration information for the review, including register name and registration number, or state that the review was not registered.	P5
	24b	Indicate where the review protocol can be accessed, or state that a protocol was not prepared.	P5

For peer review only

PRISMA 2020 Checklist

Section and Topic	Item #	Checklist item	Location where item is reported
	24c	Describe and explain any amendments to information provided at registration or in the protocol.	P6
Support	25	Describe sources of financial or non-financial support for the review, and the role of the funders or sponsors in the review.	P16
Competing interests	26	Declare any competing interests of review authors.	P16
Availability of data, code and other materials	27	Report which of the following are publicly available and where they can be found: template data collection forms; data extracted from included studies; data used for all analyses; analytic code; any other materials used in the review.	Appendices

From: Page MJ, McKenzie JE, Bossuyt PM, Boutron I, Hoffmann TC, Mulrow CD, et al. The PRISMA 2020 statement: an updated guideline for reporting systematic reviews. BMJ 2021;372:n71. doi: 10.1136/bmj.n71

For more information, visit: http://www.prisma-statement.org/

For peer review only
